# Adjuvant nivolumab and relatlimab in stage III/IV melanoma: the randomized phase 3 RELATIVITY-098 trial

**DOI:** 10.1038/s41591-025-04032-8

**Published:** 2025-10-18

**Authors:** Georgina V. Long, Charlie Garnett-Benson, Sonia Dolfi, Paolo A. Ascierto, Jun Guo, Ahmad A. Tarhini, Sunandana Chandra, Eva Muñoz-Couselo, Michele Del Vecchio, Andreia Cristina de Melo, Margaret Callahan, Helen Gogas, Reinhard Dummer, Dirk Schadendorf, Peter Koelblinger, Gaelle Quereux, Ioannis Thomas, Jia Xin Yu, Andrew Fisher, Bonnie Wang, Patrick Djidel, Armand Chouzy, Mark Semaan, Bohang Chen, Alicia M. Y. Cheong, Hussein A. Tawbi

**Affiliations:** 1 Melanoma Institute Australia, The University of Sydney, Royal North Shore and Mater Hospitals, Sydney, New South Wales Australia; 2 Bristol Myers Squibb, Princeton, NJ USA; 3 Istituto Nazionale dei Tumori IRCCS ‘Fondazione G. Pascale’, Napoli, Italy; 4 Peking University Cancer Hospital & Institute, Beijing, China; 5 Moffitt Cancer Center & Research Institute, Tampa, FL USA; 6 Northwestern University, Chicago, IL USA; 7 Vall d’Hebron Hospital and Vall d’Hebron Institute of Oncology (VHIO), Barcelona, Spain; 8 Fondazione IRCCS Istituto Nazionale dei Tumori, Milan, Italy; 9 Brazilian National Cancer Institute, Rio de Janeiro, Brazil; 10 Memorial Sloan Kettering Cancer Center, New York, NY USA; 11 National and Kapodistrian University of Athens, Athens, Greece; 12 University of Zurich, Zurich, Switzerland; 13 University Hospital Essen, National Center for Tumor Diseases (NCT-West), Campus Essen, German Cancer Consortium, Campus Essen, and University Alliance Ruhr, Research Center One Health, University Duisburg-Essen, Essen, Germany; 14 Paracelsus Medical University, Salzburg, Austria; 15 CHU Nantes, Nantes University and Immunology and New Concepts in ImmunoTherapy (INCIT), Nantes, France; 16 EDOEAP, Athens, Greece; 17 Bristol Myers Squibb, Uxbridge, UK; 18 The University of Texas MD Anderson Cancer Center, Houston, TX USA

**Keywords:** Melanoma, Randomized controlled trials, Translational research

## Abstract

Based on RELATIVITY-047, nivolumab plus relatlimab is approved for advanced melanoma. Here, to address a current unmet need for more efficacious adjuvant regimens for completely resected melanoma, the phase 3, double-blind RELATIVITY-098 trial compared adjuvant nivolumab plus relatlimab to nivolumab after complete resection of stage III/IV melanoma. Patients were randomized 1:1 to receive nivolumab 480 mg plus relatlimab 160 mg (*n* = 547) or nivolumab 480 mg (*n* = 546) intravenously every 4 weeks for ≤1 year; safety populations totaled 543 and 545 patients, respectively. The primary endpoint was recurrence-free survival (RFS), and the key secondary was overall survival; translational endpoints were exploratory. There was no difference in RFS for nivolumab plus relatlimab versus nivolumab (hazard ratio = 1.01; 95% confidence interval: 0.83–1.22; *P* = 0.928); therefore, overall survival was not tested. Translational data across trials showed lower circulating LAG-3^+^ T cells in the adjuvant setting (RELATIVITY-098) versus advanced melanoma (RELATIVITY-047), where LAG-3^+^ T cells were enriched in tumor versus blood. The absence of macroscopic tumor and reduced peripheral LAG-3^+^ T cells may explain the lack of added benefit of nivolumab plus relatlimab over nivolumab in resected versus metastatic melanoma. ClinicalTrials.gov identifier: NCT05002569.

## Abstract (web summary)

As presented at the ASCO 2025 Annual Meeting and the ESMO Congress 2025: In RELATIVITY-098, treatment of patients with stage III/IV resected melanoma with nivolumab and relatlimab compared to nivolumab alone did not significantly change recurrence-free survival, with correlative data pointing to the absence of tumor-infiltrating LAG3^+^ T cells as a potential reason.

## Main

The programmed death 1 (PD-1) immune checkpoint inhibitors nivolumab and pembrolizumab, both administered as monotherapy, and the targeted therapy combination of dabrafenib plus trametinib are standard-of-care adjuvant therapy options for patients with completely resected stage III/IV melanoma^1–5^. However, approximately 50% of patients have disease recurrence, with 5-year RFS rates of 50% for nivolumab^6^, 55% for pembrolizumab^4^ and 52% for dabrafenib plus trametinib^5^. These outcomes highlight the ongoing need to develop treatments that improve RFS while maintaining a manageable safety profile.

Nivolumab plus relatlimab fixed-dose combination (FDC; 480-mg nivolumab plus 160-mg relatlimab intravenously every 4 weeks) was approved for treatment of advanced melanoma based on the phase 2/3 RELATIVITY-047 trial^7^. In the primary analysis, there was a significant improvement with nivolumab plus relatlimab compared to nivolumab in median progression-free survival (PFS; 10.1 months versus 4.6 months; hazard ratio = 0.75; 95% confidence interval: 0.62–0.92; *P* = 0.006) at a median follow-up of 13.2 months, with a tolerable safety profile^7^. In updated analyses, blinded independent confirmed objective response rates were 43.1% with nivolumab plus relatlimab and 32.6% with nivolumab (odds ratio = 1.6; 95% confidence interval: 1.2–2.2) at a median follow-up of 19.3 months^8^, and median overall survival was 53.3 months and 33.2 months, respectively (hazard ratio = 0.77; 95% confidence interval: 0.64–0.94) at a median follow-up of 34.9 months^9^.

Given the unmet need for more efficacious adjuvant regimens for completely resected melanoma, the phase 3 RELATIVITY-098 trial compared adjuvant treatment with nivolumab plus relatlimab FDC to nivolumab in patients after complete resection of stage III/IV melanoma. Here we present the primary results of RELATIVITY-098, which demonstrate no added benefit of the combination versus nivolumab in the resected setting, and translational evidence from RELATIVITY-098 and RELATIVITY-047 to better understand the differential efficacy of this combination in the resected versus advanced melanoma settings.

## Results

### Patients

**Fig. 1:**
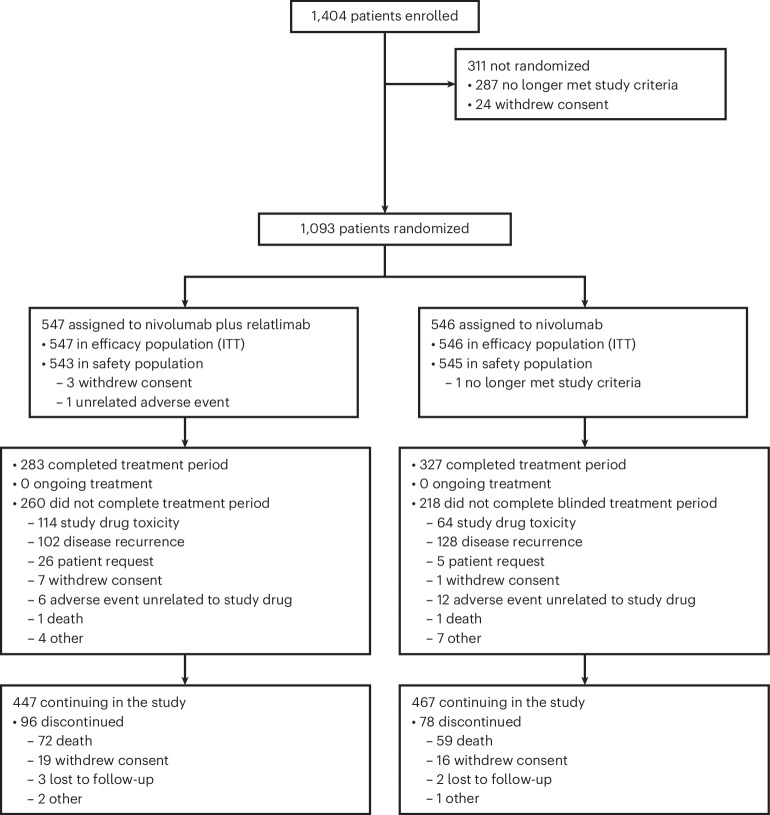
Flow diagram depicting the patient numbers in each arm throughout the study. CONSORT diagram. ITT, intention-to-treat.

From October 2021 to November 2022, a total of 1,093 patients were randomly assigned to receive nivolumab plus relatlimab (547 patients) or nivolumab (546 patients) (Fig. 1) at 163 hospitals and cancer centers worldwide (list provided in the Supplementary Information) in 24 countries worldwide. Patients were recruited by investigators through enrollment based on prespecified inclusion/exclusion criteria. Eligibility criteria and screening procedures minimized the potential of self-selection bias. Baseline characteristics were generally balanced across the treatment arms (Table 1).

**Table 1:** Characteristics of the patients at baseline

Characteristic	Nivolumab plus relatlimab (*n* =547)	Nivolumab (*n* = 546)
Median age (range) — years	59 (18‒89)	59 (19‒92)
Sex — no. (%)		
Male	327 (59.8)	315 (57.7)
Female	220 (40.2)	231 (42.3)
Geographic region — no. (%)		
United States/Canada	54 (9.9)	63 (11.5)
Australia	67 (12.2)	57 (10.4)
Europe	318 (58.1)	319 (58.4)
Latin America	76 (13.9)	72 (13.2)
China	32 (5.9)	35 (6.4)
ECOG performance status — no. (%)		
0	494 (90.3)	498 (91.2)
1	53 (9.7)	48 (8.8)
AJCC stage — no. (%)		
IIIA−IIIB	209 (38.2)	199 (36.4)
IIIC	270 (49.4)	271 (49.6)
IIID−IV	68 (12.4)	76 (13.9)
Melanoma subtype — no. (%)		
Cutaneous non-acral	436 (79.7)	454 (83.2)
Cutaneous acral	61 (11.2)	52 (9.5)
Mucosal	10 (1.8)	8 (1.5)
Unknown primary	38 (6.9)	32 (5.9)
Other	2 (0.4)	0
LDH — no. (%)		
≤ULN	496 (90.7)	486 (89.0)
>ULN	49 (9.0)	57 (10.4)
Not reported	2 (0.4)	3 (0.5)
*BRAF* status — no. (%)		
Mutant	220 (40.2)	217 (39.7)
Wild-type	195 (35.6)	200 (36.6)
PD-L1 status — no. (%)		
Positive (≥1%)	136 (24.9)	123 (22.5)
Negative (<1%)	315 (57.6)	340 (62.3)
Indeterminate/not evaluable/not reported	96 (17.6)	83 (15.2)
LAG-3 status — no. (%)		
Positive (≥1%)	352 (64.4)	352 (64.5)
Negative (<1%)	126 (23.0)	145 (26.6)
Indeterminate/not evaluable/not reported	69 (12.6)	49 (9.0)

ULN, upper limit of normal.

At the 16 December 2024 clinical cutoff date, patients had a minimum follow-up (time from last patient randomized to the cutoff date) of 23.4 months and a median follow-up (median time between randomization and the last known alive date) of 26.7 months. No patient was continuing treatment, and 96 patients (17.7%) in the nivolumab plus relatlimab arm and 78 patients (14.3%) in the nivolumab arm discontinued the study, mainly due to death (72 (13.3%) and 59 (10.8%) patients, respectively) (Fig. 1). Subsequent systemic therapy was received by 27.1% of patients in the nivolumab plus relatlimab arm and by 28.4% of patients in the nivolumab arm (Extended Data Table 1).

### Efficacy

**Fig. 2:**
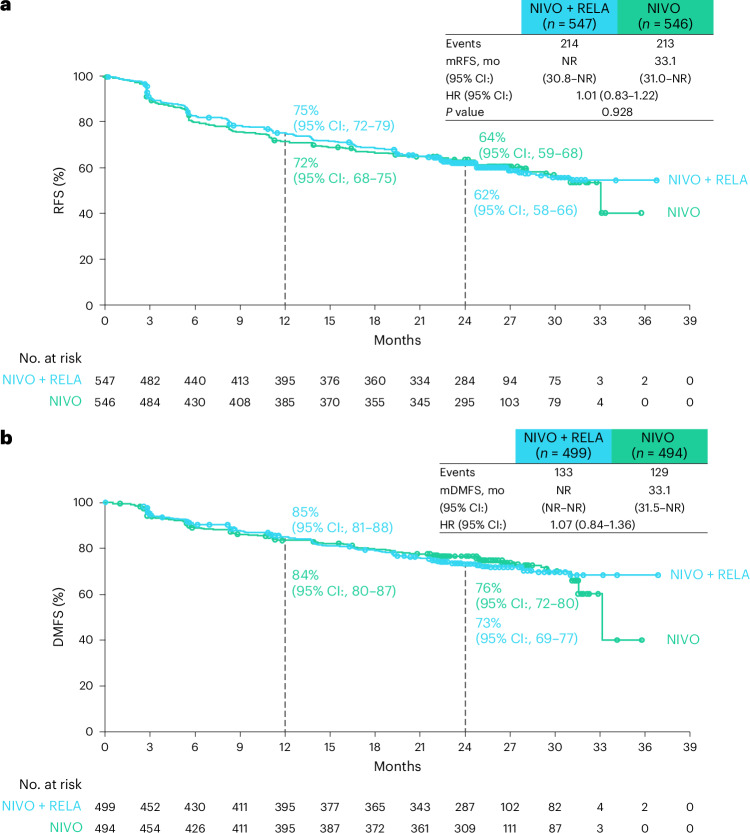
RFS and DMFS. **a**, Kaplan–Meier estimates of RFS defined as the time between randomization and the first date of documented recurrence (local, regional or distant recurrence or new primary melanomas (excluding in situ)) or death due to any cause in all randomized patients. **b**, DMFS defined as the time between randomization and first distant metastasis or death due to any cause in a subset of intention-to-treat patients, defined as those with resected stage III−IVA−IVB melanoma (those without presence of baseline metastasis prior to surgical resection—that is, stage III cutaneous and stage IVA−IVB mucosal melanoma). RFS statistical analysis was two-sided log-rank test, with no multiple comparisons. Symbols (circles) indicate censored data. Dashed lines indicate landmark analyses. CI, confidence interval; HR, hazard ratio; mDMFS, median DMFS; mRFS, median RFS; mo, months; NIVO, nivolumab; NR, not reached; RELA, relatlimab.

**Extended Data Fig. 1:**
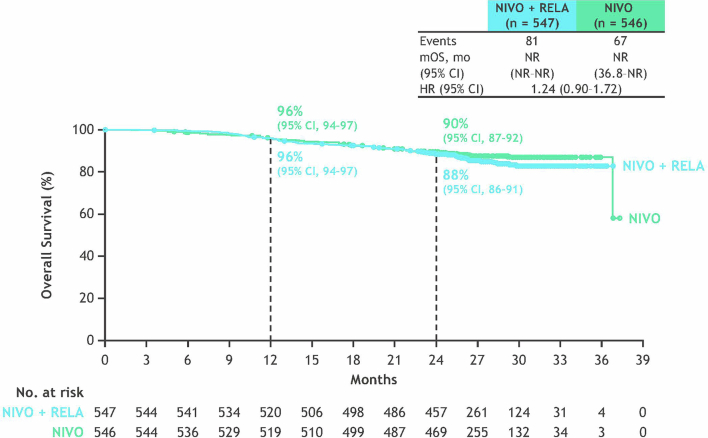
OS. Kaplan–Meier estimates of OS defined as the time between randomization and death. Symbols (circles) indicate censored data. Dashed lines indicate landmark analyses. mOS, median OS; NIVO, nivolumab; NR, not reached; RELA, relatlimab.

**Extended Data Fig. 2:**
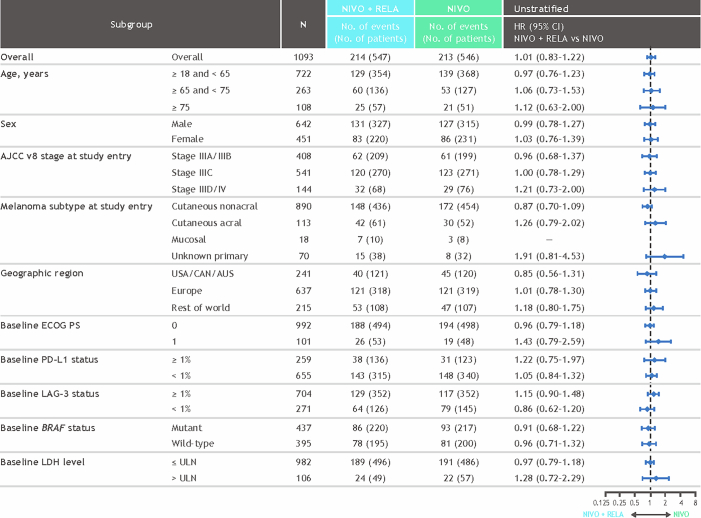
RFS according to prespecified subgroups. Shown is a forest plot of hazard ratios for disease recurrence or death among prespecified subgroups of patients in the nivolumab plus relatlimab group and nivolumab group. The hazard ratios were not stratified according to the stratification factors. The horizontal lines indicate 95% confidence intervals with the hazard ratio represented by the center dot. AJCC-8, American Joint Committee on Cancer; *Cancer Staging Manual*, Eighth Edition; ECOG PS, Eastern Cooperative Oncology Group performance status; LAG-3, lymphocyte activation gene 3; LDH, lactate dehydrogenase; NIVO, nivolumab; RELA, relatlimab.

The primary endpoint of RFS was not statistically significantly associated in the intention-to-treat population, with a hazard ratio for nivolumab plus relatlimab versus nivolumab of 1.01 (95% confidence interval: 0.83–1.22; *P* = 0.928) (Fig. 2a) and a similar incidence of recurrence events across the two arms (36.7% (*n* = 201) and 37.7% (*n* = 206), respectively) (Extended Data Table 2). Although overall survival data are immature and not tested for significance, they showed a hazard ratio for nivolumab plus relatlimab versus nivolumab of 1.24 (95% confidence interval: 0.90–1.72) (Extended Data Fig. 1). The secondary endpoint of distant metastasis-free survival (DMFS) showed a hazard ratio for nivolumab plus relatlimab versus nivolumab of 1.07 (95% confidence interval: 0.84–1.36) (Fig. 2b). RFS results for patients across prespecified subgroups were generally similar to the overall population (Extended Data Fig. 2). Additional planned secondary endpoint of PFS through next-line therapy is not reported in this paper as the analyses were not completed due to the primary endpoint not reaching statistical significance.

### Exposure and safety

The median (range) duration of therapy was similar for nivolumab plus relatlimab (11.0 months (0.03–12.06)) and nivolumab (11.0 months (0.03–12.06)). Overall, 89.0% of patients treated with nivolumab plus relatlimab and 80.4% of patients treated with nivolumab had any-grade treatment-related adverse events (TRAEs); grade 3 or 4 TRAEs were reported in 19.0% and 8.4% of patients, respectively (Table 2). The most frequent (≥10%) any-grade TRAEs in the nivolumab plus relatlimab arm were hypothyroidism (25.2%), fatigue (24.3%), pruritus (18.0%), hyperthyroidism (17.5%), rash (15.3%), arthralgia (13.3%) and diarrhea (11.2%); those in the nivolumab arm were fatigue (25.3%), pruritus (18.7%), hypothyroidism (13.6%), rash (13.4%) and arthralgia (12.7%) (Table 2). Any-grade TRAEs led to treatment discontinuation in 17.5% of patients in the nivolumab plus relatlimab arm and in 8.8% of patients in the nivolumab arm; grade 3 or 4 TRAEs led to treatment discontinuation in 9.6% and 3.1%, respectively (Table 2). Treatment-related deaths occurred in two patients (0.4%; gastrointestinal hemorrhage and sinusoidal obstructive syndrome (*n* = 1) and myocarditis (*n* = 1)) in the nivolumab plus relatlimab arm and in one patient (0.2%; immune-mediated myocarditis and immune-mediated myositis) in the nivolumab arm. Immune-mediated adverse events (Extended Data Table 3) and adverse events of special interest (Extended Data Table 4) are presented.

**Table 2:** Safety summary^a^

Event	Nivolumab plus relatlimab (*n* = 543)		Nivolumab (*n* = 545)	
	Any grade	Grade 3 or 4	Any grade	Grade 3 or 4
Any adverse event, *n* (%)	522 (96.1)	155 (28.5)	520 (95.4)	100 (18.3)
Any adverse event leading to discontinuation, *n* (%)	102 (18.8)	55 (10.1)	65 (11.9)	28 (5.1)
TRAEs, *n* (%)	483 (89.0)	103 (19.0)	438 (80.4)	46 (8.4)
TRAEs leading to discontinuation, *n* (%)	95 (17.5)	52 (9.6)	48 (8.8)	17 (3.1)
TRAEs in ≥5% patients, *n* (%)				
Hypothyroidism	137 (25.2)	3 (0.6)	74 (13.6)	0
Fatigue	132 (24.3)	1 (0.2)	138 (25.3)	1 (0.2)
Pruritus	98 (18.0)	0	102 (18.7)	1 (0.2)
Hyperthyroidism	95 (17.5)	3 (0.6)	53 (9.7)	0
Rash	83 (15.3)	0	73 (13.4)	4 (0.7)
Arthralgia	72 (13.3)	2 (0.4)	69 (12.7)	3 (0.6)
Diarrhea	61 (11.2)	6 (1.1)	54 (9.9)	2 (0.4)
Increased alanine aminotransferase	50 (9.2)	8 (1.5)	34 (6.2)	0
Asthenia	46 (8.5)	3 (0.6)	44 (8.1)	1 (0.2)
Dry mouth	46 (8.5)	0	29 (5.3)	0
Increased aspartate aminotransferase	41 (7.6)	5 (0.9)	27 (5.0)	0
Hypophysitis	40 (7.4)	11 (2.0)	7 (1.3)	2 (0.4)
Nausea	39 (7.2)	0	28 (5.1)	0
Myalgia	34 (6.3)	2 (0.4)	30 (5.5)	0
Infusion-related reaction	30 (5.5)	1 (0.2)	18 (3.3)	0
Treatment-related deaths, *n* (%)	2 (0.4)^b^		1 (0.2)^c^	

^a^The safety population included all patients who had received at least one dose of a trial drug. Listed are events that were reported between the first dose and 30 days after the last dose. The severity of adverse events was graded according to the National Cancer Institute Common Terminology Criteria for Adverse Events version 5.0. ^b^Causes of deaths were gastrointestinal hemorrhage and sinusoidal obstructive syndrome (*n* = 1) and myocarditis (*n* = 1). ^c^Cause of death was immune-mediated myocarditis and immune-mediated myositis (*n* = 1).

### Translational data

Clinical results presented in this report show that there was no added benefit in the resected setting for nivolumab plus relatlimab over nivolumab, although benefit was demonstrated in advanced melanoma in RELATIVITY-047 (refs. ^7–9^). Therefore, as post hoc analyses, the pharmacodynamic effects of nivolumab plus relatlimab and nivolumab were compared between patients in the adjuvant treatment setting from RELATIVITY-098 and patients in the advanced melanoma treatment setting from RELATIVITY-047.

**Fig. 3:**
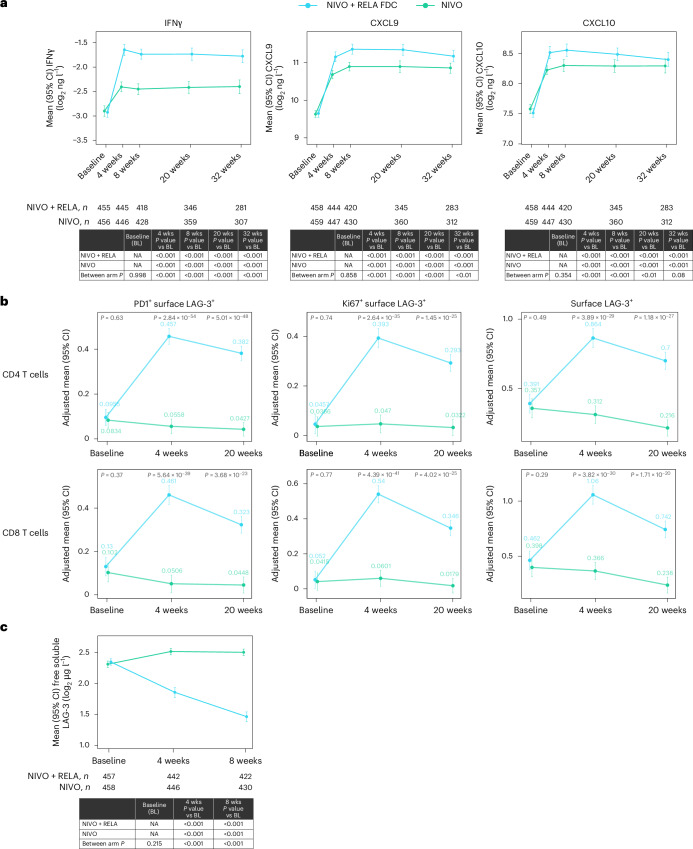
Pharmacodynamic and target engagement in the periphery for adjuvant treatment of melanoma. **a**, Expression of serum cytokines. **b**, Expression of surface LAG-3^+^ CD4 and CD8 T cells. **c**, Soluble LAG-3. **a**, Linear mixed-effect models per cytokine. Comparisons were between baseline and each on-treatment mean and of the mean of each arm for each timepoint. **b**, Linear mixed-effect models per flow populations. Comparisons of mean of each arm for each timepoint. **c**, Linear mixed-effect models for free soluble LAG-3. Comparisons were between baseline and each on-treatment mean and of the mean of each arm for each timepoint. Tests were two-sided *t*-tests for pairwise comparisons of estimated marginal means with 95% confidence interval (CI). No adjustment was made for multiple comparisons because each biomarker/timepoint was analyzed one at a time. NA, not applicable; NIVO, nivolumab; RELA relatlimab; wks, weeks.

**Extended Data Fig. 3:**
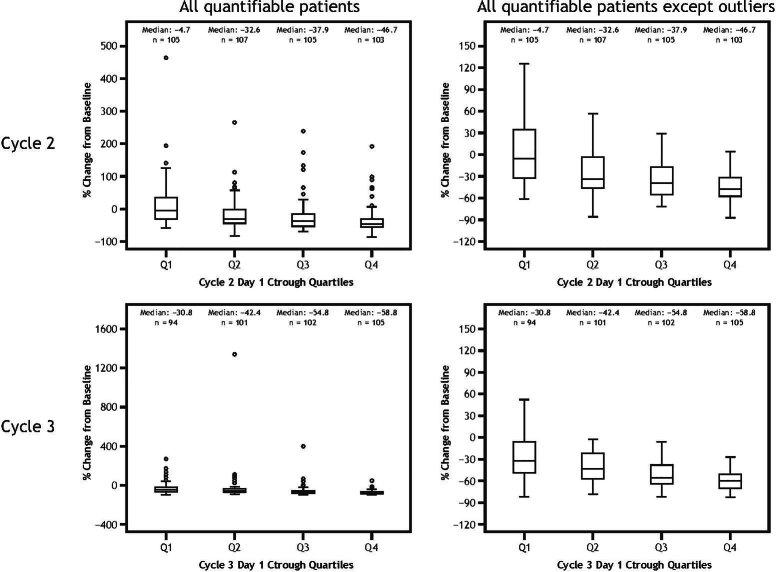
Decrease in Soluble LAG-3 Levels Driven by Relatlimab Trough Concentration (Ctrough) Exposure. Center lines represent medians, the bounds of the boxes indicate the 25th and 75th percentiles, the whiskers represent the minima and maxima, and dots represent outliers; baseline = last non-missing result with a collection date-time less than or equal to the date-time of the first active dose of study medication; Q1 = [0.0892-8.7000); Q2 = [8.7000-13.2790); Q3 = [13.2790-19.5397); Q4 = [19.5397-50.5000].

**Extended Data Fig. 4:**
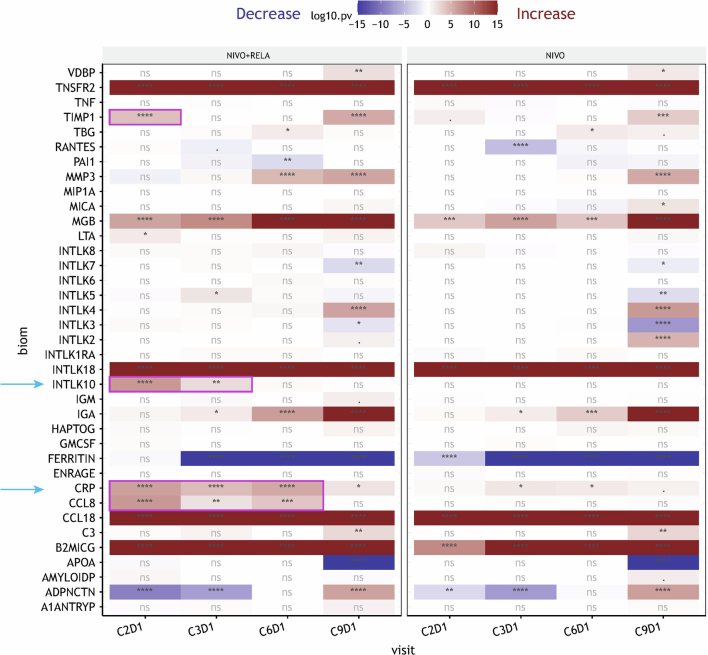
Pharmacodynamics Heat Map for Serum Factors in Patients with Melanoma treated with Adjuvant Nivolumab plus Relatlimab versus Nivolumab Alone in RELATIVITY-098. The figure presents a heatmap summarizing linear mixed effect models for a cytokine panel separated by treatment arm. Each individual box represents the statistical comparison between the on-treatment time point on the x-axis with the baseline time point, colored by the difference in means, in which blue is lower on treatment and red is higher on-treatment. C, cycle; D, day; ns, not significant/*P* > 0.1. •*P* ≤ 0.1(trending), *P ≤ 0.05, **P ≤ 0.01, ***P ≤ 0.001, and ****P ≤ 0.0001.

Similar to results observed in patients with advanced melanoma from RELATIVITY-047 (ref. ^10^), pharmacodynamic and target engagement evaluation in patients in RELATIVITY-098 demonstrated that inflammatory cytokines (interferon gamma (IFNγ), C-X-C motif chemokine ligand 9 and C-X-C motif chemokine ligand 10 (CXCL9 and CXCL10)) were significantly increased by nivolumab plus relatlimab and nivolumab treatment, with greater increases in the nivolumab plus relatlimab arm than in the nivolumab arm (Fig. 3a). In a longitudinal blood analysis, nivolumab plus relatlimab promoted greater expansion of LAG-3^+^ T cells than nivolumab (Fig. 3b). Additionally, a significant decrease in free soluble LAG-3 was observed with nivolumab plus relatlimab compared to increases with nivolumab, supporting relatlimab target engagement (Fig. 3c). An inverse relationship was observed between free soluble LAG-3 levels in serum and relatlimab exposures, with free soluble LAG-3 levels decreasing as relatlimab trough concentration increased (Extended Data Fig. 3). Immunosuppressive cytokines were significantly increased in the nivolumab plus relatlimab arm but not in the nivolumab arm (for example, IL-10 and C-reactive protein) (Extended Data Fig. 4).

**Fig. 4:**
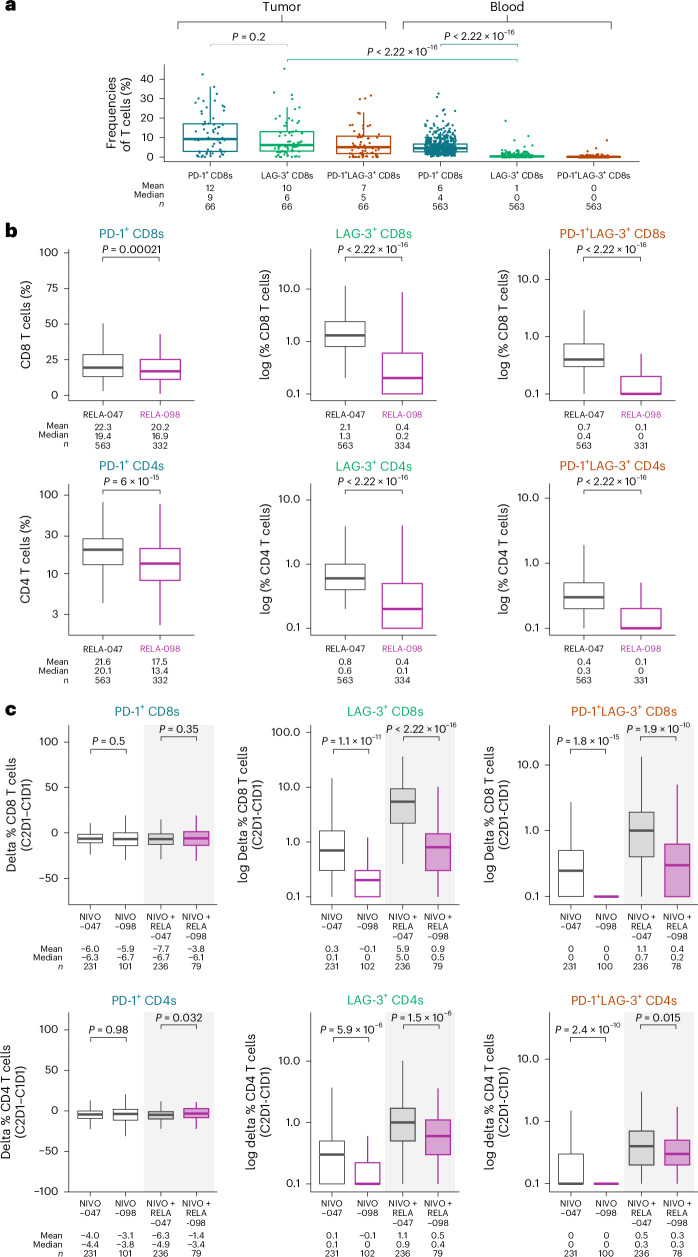
Comparative analysis of LAG-3^+^ and PD-1^+^ CD8 and CD4 T cells in metastatic and adjuvant treatment of melanoma. **a**, Baseline PD-1^+^, LAG-3^+^ and PD-1^+^LAG3^+^ CD8 cells in tumor and blood in advanced melanoma (RELATIVITY-047). **b**, Baseline expression of PD-1^+^, LAG-3^+^ and PD-1^+^LAG-3^+^ CD8 and CD4 cells in blood in advanced melanoma (RELATIVITY-047) and adjuvant-treated melanoma (RELATIVITY-098). **c**, Delta change of baseline to on-treatment expression of PD-1^+^, LAG-3^+^ and PD-1^+^LAG-3^+^ CD8 and CD4 cells in blood in advanced and adjuvant-treated melanoma. **a**, Tumor CD8 frequencies from CODEX/PhenoCycler. Blood frequencies from multiparametric flow cytometry. **b**, Figure is non-log mean and non-log median, and *n* is the number of patients with flow cytometry. Figure is the log-transformed frequencies of each population. **c**, Figure is non-log mean of delta and non-log median of delta, and *n* is the number of patients with flow cytometry. LAG-3^+^ and LAG-3^+^PD-1^+^ figures represent the log-transformed frequencies of each population. Statistical tests were two-sided Wilcoxon tests with no multiple comparisons. Error bars are s.e. of the estimated marginal means.

Immune checkpoint expression on T cells in the blood and tumor was investigated to understand whether tumor (which was surgically removed in the adjuvant treatment setting) may be necessary for relatlimab benefit. In samples from patients with advanced melanoma from RELATIVITY-047 the levels of PD-1^+^ CD8 T cells in the tumor and blood were similar; however, LAG-3^+^ and LAG-3^+^PD-1^+^ CD8 T cells were substantially higher in tumor (multiplex CODEX analysis) versus blood (flow cytometry) (Fig. 4a). The similar multiplex CODEX analyses were not available for the RELATIVITY-098 study. Comparing baseline blood samples of advanced melanoma (RELATIVITY-047) to those of resected melanoma (RELATIVITY-098) demonstrated similar PD-1^+^ T cell levels (Fig. 4b). By contrast, among patients in the advanced disease setting, there were higher levels of LAG-3-expressing T cells in the blood at baseline (Fig. 4b) and a greater magnitude of increase in LAG-3^+^ T cells upon treatment with nivolumab plus relatlimab than in patients treated in the adjuvant disease setting after tumor resection (Fig. 4c).

**Extended Data Fig. 5:**
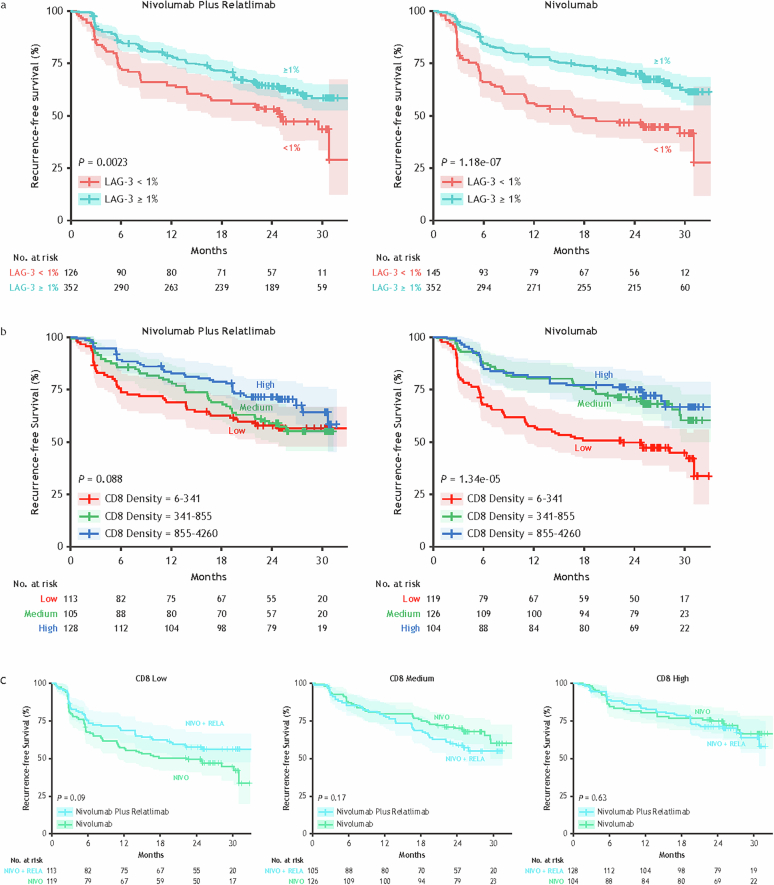
RFS Association with Baseline TME Biomarkers. **a**, Association with LAG-3 within treatment arms. **b**, Association with CD8 within treatment arms. **c**, Association with CD8 between treatment arms. CD8 density is defined as CD8 positive cells/mm^2^. Statistical testing is two-sided log-rank test with no multiple comparisons and error bars are 95% confidence interval. CD, cluster of differentiation; LAG-3, lymphocyte activation gene 3; TME, tumor microenvironment.

**Extended Data Fig. 6:**
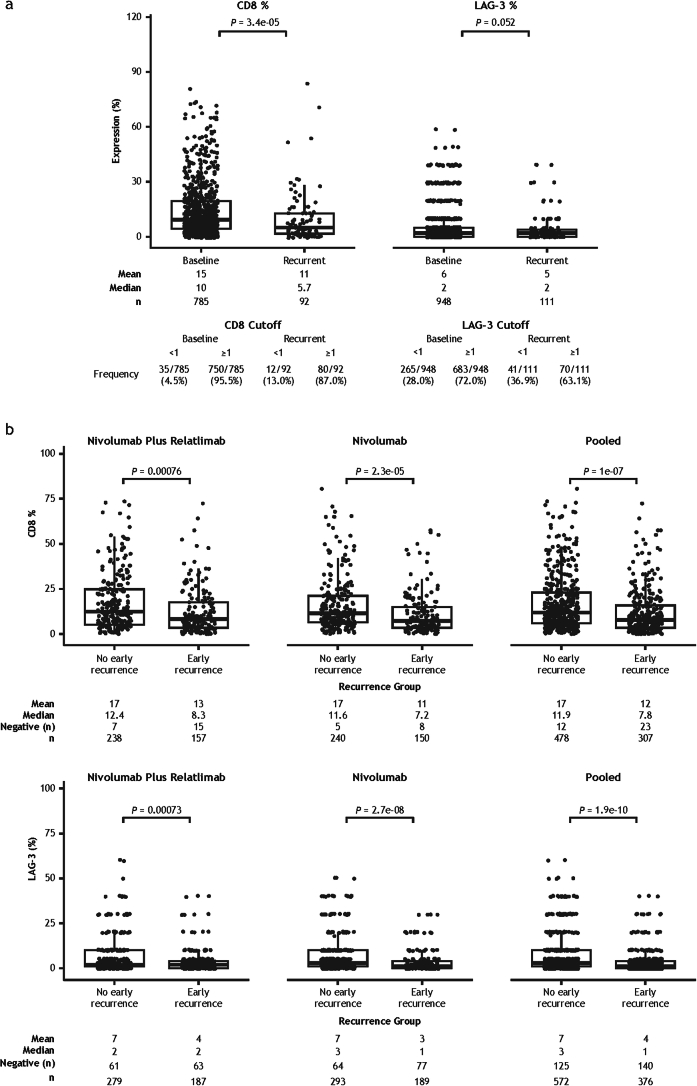
Evaluation of CD8 and LAG-3 Biomarkers in Baseline and Recurrent Tumors. **a**, CD8 and LAG-3 expression overall in baseline vs early recurrent tumors. **b**, Baseline expression of CD8 and LAG-3 in patients who did not have a recurrence vs those who had an early recurrence (prior to the minimum follow-up of 23.4 months). **a**, Box plot of screening/baseline versus recurrent CD8% and LAG3% in pooled treatment arms. The figure shows each biomarker, visit, and frequency of patients below and above the 1% cutoff. **b**, Negative(n) indicates number of patients with <1% immunohistochemistry and N indicates number of patients with immunohistochemistry. Box plot shows 25%, 50% and 75% quartiles and statistical tests are two-sided Wilcoxon with no multiple comparisons.CD, cluster of differentiation; LAG-3, lymphocyte activation gene 3.

When correlating tumor expression with RFS, higher LAG-3 and CD8 expression in resected tumors enriched for RFS benefit in both the nivolumab plus relatlimab and nivolumab arms (Extended Data Fig. 5a,b). In addition, there was a trend toward RFS benefit for nivolumab plus relatlimab compared to nivolumab in CD8 low inflamed tumors (Extended Data Fig. 5c). Overall, CD8 expression was higher in baseline resected tumors compared to recurrent tumors (Extended Data Fig. 6a), and there was a higher proportion of LAG-3^−^ (<1%) tumors in recurrent tumor samples compared to baseline resected tumors (Extended Data Fig. 6a). Expression of CD8 and LAG-3 at baseline was lower in patients who had a recurrence than in those who did not in both arms (Extended Data Fig. 6b).

## Discussion

In RELATIVITY-098, nivolumab plus relatlimab did not significantly improve RFS over nivolumab as adjuvant treatment for patients after complete resection of stage III−IV melanoma. However, in RELATIVITY-047, nivolumab plus relatlimab was shown to be superior to nivolumab in terms of PFS in patients with advanced melanoma^7^. Comparing biomarker results in resected melanoma in RELATIVITY-098 and advanced melanoma in RELATIVITY-047 suggested that the presence of tumor and associated higher levels of LAG-3^+^ T cells in the blood may be necessary to derive additional clinical benefit with nivolumab plus relatlimab over nivolumab.

There was a similar recurrence pattern in the nivolumab plus relatlimab and nivolumab arms of RELATIVITY-098, and RFS results in prespecified subgroups were generally similar to those in the intention-to-treat population. Overall survival and DMFS were also similar across the treatment arms. The safety profile of nivolumab plus relatlimab in RELATIVITY-098 was similar to that in RELATIVITY-047, with no new safety signals; similar to the metastatic setting, an increased frequency of TRAEs for nivolumab plus relatlimab compared to nivolumab alone was reported^7^. Furthermore, similar proportions of patients receiving nivolumab plus relatlimab discontinued treatment because of TRAEs in RELATIVITY-098 and RELATIVITY-047 (17.5% and 14.6%, respectively)^7^.

Results from RELATIVITY-047 established the superiority of nivolumab plus relatlimab over nivolumab in metastatic melanoma^7–9^. At a follow-up of 4 years, nivolumab plus relatlimab demonstrated a sustained PFS benefit (hazard ratio = 0.78; 95% confidence interval: 0.65–0.93) and a clinically meaningful overall survival benefit (hazard ratio = 0.77; 95% confidence interval: 0.64–0.94) compared to nivolumab^9^ while maintaining a tolerable safety profile. Although there was no added efficacy benefit for nivolumab plus relatlimab in resected melanoma in RELATIVITY-098, pharmacodynamic modulation of IFNγ and target engagement via a reduction in free soluble LAG-3 were similar to those observed in patients with advanced disease in RELATIVITY-047 (ref. ^10^). In addition, higher levels of biomarkers associated with inflammation (LAG-3 and CD8 expression) in baseline tumors enriched for RFS benefit in both treatment arms, similar to trends observed for PFS in patients with advanced melanoma in RELATIVITY-047 (ref. ^9^).

Comparative biomarker results from RELATIVITY-098 and RELATIVITY-047 explored why clinical activity differed between resected and advanced disease. In patients with advanced melanoma, there was a higher percentage of LAG-3^+^ and LAG-3^+^PD-1^+^ CD8 T cells in tumor samples than in blood samples. In the blood, PD-1^+^ CD8 T cells were much higher than LAG-3^+^ CD8 T cells, whereas LAG-3^+^ T cell levels and PD-1^+^ CD8 T cell levels in the tumor were similar. The higher level of LAG-3^+^ T cells in the blood of patients with advanced melanoma compared to patients with resected melanoma tumors suggested that there were more T cells with a relatlimab ‘target’ in the advanced setting than in the resected disease setting, which may have been driven by the presence of tumor. Previous analysis in advanced melanoma revealed that response to nivolumab plus relatlimab, but not nivolumab, was associated with an on-treatment increase in LAG-3^+^ T cells^10^. When comparing biomarker results from RELATIVITY-047 and RELATIVITY-098, there was a greater magnitude of on-treatment increase of LAG-3^+^ T cells in the advanced disease setting than in the resected disease setting. In comparison, there were substantial levels of PD-1^+^ T cells in the blood, and the on-treatment changes were similar between the two settings. Although informative, these analyses have inherent limitations preventing adequate conclusions to be drawn, including that multiplex CODEX analyses were not available for the RELATIVITY-098 study. This limitation prevented direct assessment of LAG-3^+^ T cells within earlier-stage tumors, leading to insight inferred from the data generated in the advanced setting. The inferences drawn here need to be tested directly comparing resected tumor with blood in patients with adjuvant setting disease.

Published studies support the requirement of tumor presence for increased benefit with the addition of relatlimab to nivolumab, specifically during neoadjuvant therapy administered prior to surgery^11–13^. Nivolumab plus relatlimab showed impressive activity as neoadjuvant therapy in a single-arm study in melanoma, with a major pathologic response rate of 63%^11^, and in MMR-deficient colon cancer, with a major pathologic response rate of 92%^12^. In addition, encouraging initial neoadjuvant activity was observed in a randomized phase 2 study with non-small cell lung cancer, with a major pathologic response rate of 30%^13^. Moreover, in advanced melanoma (RELATIVITY-047), responders to nivolumab plus relatlimab were associated with CD8^+^CD103^+^ gene expression signatures indicative of tissue-resident T cells^10^.

Along with nivolumab plus relatlimab, other immune checkpoint inhibitor combinations have proven unsuccessful as melanoma adjuvant therapy. The KeyVibe-010 trial, which investigated adjuvant treatment with pembrolizumab plus the anti-TIGIT antibody vibostolimab versus pembrolizumab monotherapy, met the prespecified futility criteria for RFS and was terminated, with a higher rate of discontinuation in the combination arm due to immune-mediated adverse events^14,15^. Moreover, treatment with nivolumab plus ipilimumab did not meet the primary endpoint (RFS) of adjuvant therapy for melanoma (CheckMate-915)^16^, whereas it was shown to be effective in neoadjuvant therapy (NADINA)^17^. New modalities, such as mRNA vaccines, may overcome some of the challenges associated with the lack of tumor in the resected setting by mimicking the neoadjuvant state and eliciting an immune response against preselected or personalized tumor neoantigens^18^.

As mentioned, higher LAG-3 and CD8 expression in baseline tumors in patients with resected melanoma (RELATIVITY-098) enriched for RFS benefit in both the nivolumab plus relatlimab and nivolumab arms. Interestingly, there were lower levels of these biomarkers in recurrent tumors than in baseline tumors (Supplementary Fig. 5a), suggesting that recurring tumors were less inflamed. Moreover, patients with recurrent disease demonstrated lower levels of these inflammatory biomarkers in their baseline resected tumors (Supplementary Fig. 5b).

In conclusion, nivolumab plus relatlimab did not significantly improve RFS compared to nivolumab in patients with resected stage III/IV melanoma in RELATVITY-098, despite nivolumab plus relatlimab having been proven to provide benefit in patients with advanced melanoma. Translational evidence suggests that the presence of tumor may be required for the benefit of nivolumab plus relatlimab compared to nivolumab in melanoma. Nivolumab plus relatlimab remains a standard-of-care option for patients with advanced melanoma.

## Methods

### Trial oversight and ethics approval

The protocol and amendments for this trial (available in the Supplementary Information) were reviewed by the institutional review board or independent ethics committee for each trial site, and all patients provided written informed consent before enrollment. The trial was conducted in accordance with International Council for Harmonization Good Clinical Practice guidelines and was designed by the trial steering committee and the sponsor. Data were collected by the sponsor and analyzed in collaboration with the authors who vouch for the accuracy and completeness of the data and for the fidelity of the trial to the protocol. An independent data monitoring committee was established to provide oversight to assess the efficacy and safety profile of nivolumab plus relatlimab and nivolumab. All authors contributed to drafting the manuscript, provided critical review and gave final approval to submit the manuscript for publication. Professional medical writing and editorial assistance were funded by the sponsor.

### Patients

Eligible patients were at least 12 years of age with an Eastern Cooperative Oncology Group (ECOG) performance status of 0–1 and stage IIIA (>1-mm tumor in lymph node), stage IIIB/C/D or stage IV (no evidence of disease) melanoma (per the American Joint Committee on Cancer (AJCC) Cancer Staging Manual (eighth edition) (AJCC-8))^19^ completely resected <90 days from randomization. Patient exclusions included prior treatment for melanoma other than surgery; adjuvant brain radiotherapy or adjuvant interferon; prior immunotherapy for any malignancy; concurrent prior malignancy within 2 years of randomization; ocular melanoma, except conjunctival melanoma; or any condition requiring treatment with a systemic corticosteroid within 14 days, or other immunosuppressive medications within 30 days, of randomization.

### Trial design, treatment and endpoints

RELATIVITY-098 was a double-blind phase 3 trial with patients randomly assigned in a 1:1 ratio to receive 480 mg of nivolumab plus 160 mg of relatlimab as FDC or 480 mg of nivolumab administered via intravenous infusion every 4 weeks for a maximum of 1 year (or ≤13 doses) or until recurrence of disease, unacceptable adverse events or withdrawal of consent. Patients were stratified according to AJCC-8 stage (IIIA/IIIB or IIIC or IIID/IV (including mucosal melanoma)) and geographic region (United States/Canada/Australia or Europe or rest of the world). Randomization was carried out via permutated blocks within each stratum. The sponsor, participants, investigator and site staff were blinded to the study therapy administered.

The primary endpoint was investigator-assessed RFS, with computed tomography scans performed every 12 weeks for 2 years and then twice a year; patients who did not undergo complete lymph node dissection required ultrasound surveillance. Overall survival was the key secondary endpoint to be hierarchically tested if the primary endpoint was met. Other secondary endpoints included investigator-assessed DMFS, PFS through next-line therapy and safety. The severity of adverse events was graded according to National Cancer Institute Common Terminology Criteria for Adverse Events version 5.0. Included TRAES are those reported between the first dose and 30 days after the last dose. In addition, immune-mediated adverse events and adverse events of special interest reported between the first dose and 135 days after the last dose are reported per category.

### Exploratory translational endpoints

Biomarker analyses were exploratory and were performed on patient blood samples from RELATIVITY-098 and RELATIVITY-047 at baseline and on treatment to assess expression or expansion of biomarker-specific immune cell populations and soluble proteins. The RELATIVITY-047 study (NCT03470922) was previously published^7–9^ and can be referred to for additional information on the RELATIVITY-047 study, including study compliance and a link to the protocol. In brief, eligible patients were ≥12 years of age with previously untreated, unresectable stage III or IV melanoma and had tumor tissue that was evaluable for LAG-3 and programmed death ligand 1 (PD-L1) expression. Enrolled patients were randomly assigned 1:1 to receive 480 mg of nivolumab and 160 mg of relatlimab as an FDC or 480 mg of nivolumab alone, administered intravenously every 4 weeks until disease progression, unacceptable adverse effects or withdrawal of consent. Random assignment was stratified according to LAG-3 expression (≥1% or <1%), PD-L1 expression (≥1% or <1%), *BRAF V600* mutation status and metastasis stage (M0 or M1 with normal lactate dehydrogenase (LDH) levels versus M1 with elevated LDH levels).

Cytokines and free soluble LAG-3 were measured in whole blood serum samples from RELATIVITY-098 at baseline and on treatment to assess pharmacodynamic activity and target engagement. On-treatment samples in general were taken at cycle 2, day 1 (C2D1), with samples also taken for select analyses on day 1 of cycles 3, 6 and 9.

Analyses of patient tumor samples from RELATIVITY-047 were performed by CODEX/PhenoCycler system to assess expression of marker-specific CD8 T cells. Images were analyzed using HALO AI to segment cells and measure mean marker intensities per cell (Indica Labs). Cell segmentation used a deep learning model trained on DAPI-stained images to segment nuclei, and the cytoplasm compartment was approximated as a 3-µm radius from segmented nuclei. Segmented cells were classified into broad lineages (that is, tumor, immune and other) using a secondary deep learning image classifier model trained on SOX10, CD45, PCNA, Collagen IV and DAPI channels. For each cell, the mean intensities per cellular compartment (that is, nuclear and cytoplasmic) were measured and exported into a tabular format for subsequent phenotyping in R. Cell phenotyping was computed in R by thresholding marker intensities (for example, CD3, CD4 and CD8) and deriving cell classes through logical combinations of binarized markers with the broad cell lineages (tumor, immune and other), resolving conflicts by prioritizing markers with cleaner segmentation profiles.

LAG-3 and PD-1 expression on CD8 and CD4 T cells in the peripheral blood was analyzed on a five-laser Cytek Aurora system (Cytek Biosciences) using a 25-color LAG-3-specific immunophenotyping panel. Flow cytometric analysis for RELATIVITY-047 and RELATIVITY-098 was performed in an exploratory manner. Pre-treatment and on-treatment frozen peripheral blood mononuclear cell (PBMC) samples (RELATIVITY-047) or whole blood (RELATIVITY-098) from patients were sent for immunophenotypic analysis at Q2 Solutions in Atlanta, Georgia. In brief, samples were thawed, stained using a custom antibody panel and subsequently acquired on a five-laser Aurora spectral cytometer from Cytek Biosciences. Data were analyzed in the OMIQ platform from Dotmatics using a hierarchical gating strategy to remove non-cellular debris, spectral artifacts, dead cells and non-lymphocytes before T cell (CD3^+^CD56^−^CD4^+^CD8^−^ and CD3^+^CD56^−^CD4^−^CD8^+^) subsets of interest were identified using specific markers and reported out as relative frequencies. A PBMC sample from the same presumed normal healthy volunteer was included in all experiments to ensure inter-assay consistency.

Peripheral biomarker levels (for example, cytokines and peripheral flow cytometry) were analyzed using linear mixed-effect models for pharmacodynamic changes. Separate models were fitted for each treatment arm to evaluate changes over time, with biomarker concentration/frequency as the dependent variable, study timepoints (for example, screening, C2D1, etc.) as fixed effects and participant-specific random intercepts to account for repeated measures. Within each arm, pairwise contrasts were used to estimate the difference between the screening and each on-treatment timepoint. In addition, differences between treatment arms were assessed at each timepoint.

Tumor biomarker expression was assessed at baseline and at recurrence via immunohistochemistry. Monoplex immunohistochemistry staining was used to evaluate PD-L1, LAG-3 and CD8 expression in resected and recurrent tumor samples from RELATIVITY-098.

### Statistical analysis

An approximate sample size of 1,050 patients was planned to achieve the required 410 RFS events and show a significant difference in investigator-assessed RFS with a two-sided *α* of 0.05 using a stratified log-rank test with at least 90% statistical power when the average hazard ratio of nivolumab and relatlimab FDC versus nivolumab was 0.72 and an assumed cure rate of 0.52 in the nivolumab arm. The actual events were 427, with a critical hazard ratio of 0.815 and cumulative power of 91.5%. Two interim analyses were performed at a cumulative power of 48% (12 February 2024) and 77% (29 July 2024), but the study had remained blinded. The study significance boundaries were based on an O’Brien−Fleming alpha spending function.

A key secondary endpoint of overall survival was to have been tested hierarchically by stratified log-rank test if the primary endpoint was met. At the time of final RFS analysis, there were 148 deaths, representing 48% of the information fraction.

RFS was compared using a two-sided log-rank test on all randomized patients; hazard ratio and confidence intervals were estimated using a stratified Cox proportional hazards model and survival curves using Kaplan–Meier methodology. Hazard ratio and confidence intervals were estimated using a non-stratified Cox proportional hazards model on the prespecified subgroup analyses of patients. DMFS was similarly compared on a subset of patients defined as those with resected stage III−IVA−IVB melanoma. For biomarker analyses, the Wilcoxon test was used to compare frequencies of T cells in tumor and blood samples and between patients with resected melanoma (RELATIVITY-098) and advanced melanoma (RELATIVITY-047).

Efficacy analysis populations were as stated above. Safety was assessed in all patients who received at least one dose of study drug. Biomarker analyses were performed on patients for whom samples and biomarker data were available. Data collection software used was Medidata Classic Rave (version 2025). All clinical analyses were performed using SAS software (SAS Institute) and R version 4.3.1. Differences in biomarker levels between baseline and recurrence were evaluated using the Wilcoxon signed-rank test. Baseline biomarker expression was also compared between patients with recurrence and those censored (no recurrence) using the Wilcoxon rank-sum test. All statistical tests were two-sided, and *P* < 0.05 was considered significant.

### Reporting summary

Further information on research design is available in the Nature Portfolio Reporting Summary linked to this article.

## Online content

Any methods, additional references, Nature Portfolio reporting summaries, source data, extended data, supplementary information, acknowledgements, peer review information; details of author contributions and competing interests; and statements of data and code availability are available at 10.1038/s41591-025-04032-8.

## Supplementary information

Supplementary Information Institutional review board table, Protocol and Statistical analysis plan.

Reporting Summary

## Figures and Tables

**Extended Data Table 1:**
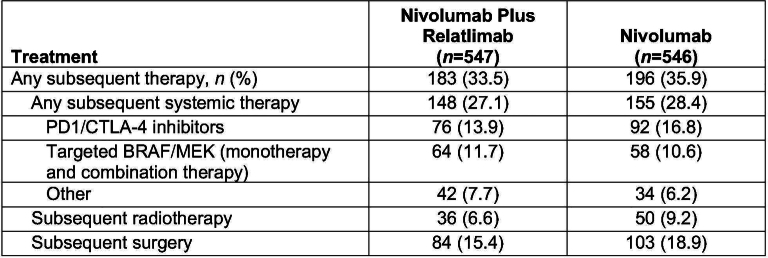
Subsequent treatment in all patients who underwent randomization^a^

^a^Patients may have received more than one type of post-protocol therapy, and more than one agent within each type. All percentages are based on the total number of patients in each arm. CTLA-4, cytotoxic T-lymphocyte antigen 4; PD-1, programmed death-1.

**Extended Data Table 2:**
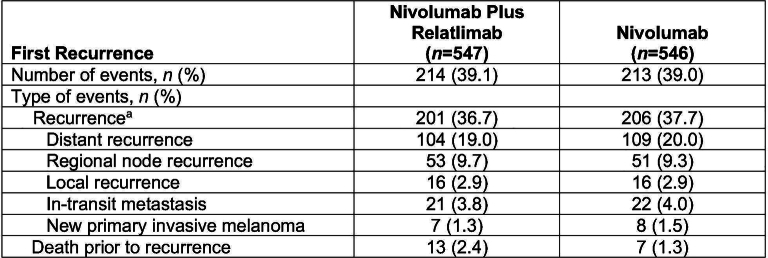
Pattern of recurrence

^a^For patients who had multiple recurrences identified on the same day, the most serious type is tabulated according to the displayed prespecified hierarchy.

**Extended Data Table 3:**
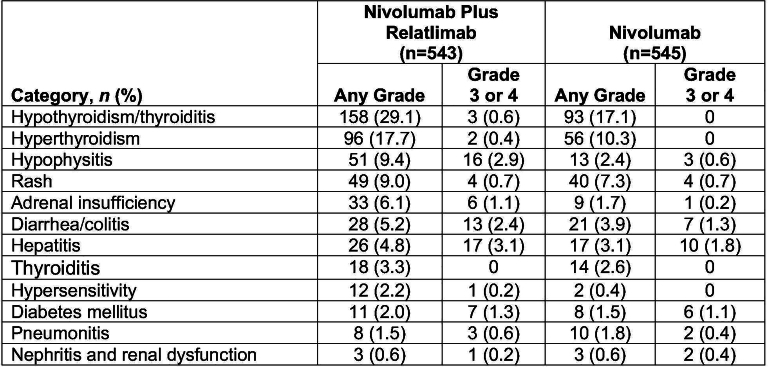
Immune-mediated adverse events by category^a^

^a^The safety population included all patients who had received at least one dose of a trial drug. Listed are events that were reported between the first dose and 135 days after the last dose. Includes patients with immune-mediated adverse events where immune modulating medication was initiated by category. The severity of adverse events was graded according to the National Cancer Institute Common Terminology Criteria for Adverse Events version 5.0.

**Extended Data Table 4:**
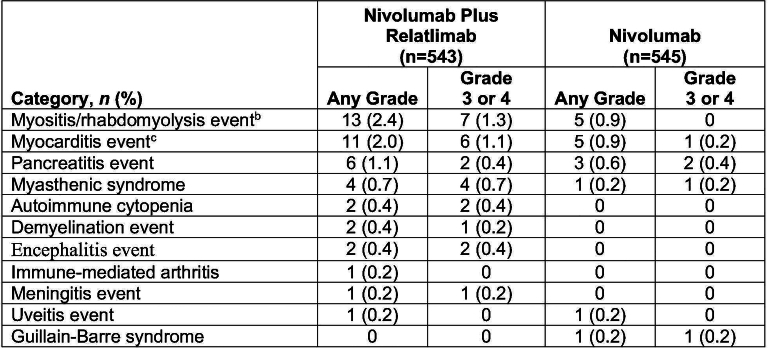
Other adverse events of special interest by category^a^

^a^The safety population included all patients who had received at least one dose of a trial drug. Listed are events that were reported between the first dose and 135 days after the last dose. The table includes patients with immune-mediated adverse events in which immune modulating medication was initiated by category. The severity of adverse events was graded according to the National Cancer Institute Common Terminology Criteria for Adverse Events version 5.0. ^b^There was 1 grade 5 event in the NIVO arm. This category includes the preferred terms of myositis, rhabdomyolysis, autoimmune myositis, and immune-mediated myositis. ^c^There was 1 grade 5 event for each arm.

## Data Availability

Bristol Myers Squibb will honor legitimate requests for our clinical trial data from qualified researchers with a clearly defined scientific objective. We share data from phase 2−4 interventional clinical trials completed on or after 1 January 2008 and evaluate medicines and indications approved in the United States, the European Union and other designated markets. Data shared may include non-identifiable patient-level and study-level clinical trial data, full clinical study reports and protocols. Sharing is subject to protection of patient privacy and respect for patient informed consent and publication of the primary results in peer-reviewed journals. Bristol Myers Squibb reserves the right to update and change criteria at any time. Other criteria may apply. For details, please visit Bristol Myers Squibb at https://www.bms.com/researchers-and-partners/independent-research/data-sharing-request-process.html. The option to submit data requests as well as review criteria for data requests are available at https://vivli.org/ourmember/bristol-myers-squibb/. The study protocol of RELATIVITY-098 is provided in the Supplementary Information.
