# Supplementary material for: Adjuvant nivolumab and relatlimab in stage III/IV melanoma: the randomized phase 3 RELATIVITY-098 trial
Source: Nat Med. 2025 Oct 18;31(12):4301–9. doi: 10.1038/s41591-025-04032-8 (PMC12705465; doi:10.1038/s41591-025-04032-8)
Supplement: Supplementary file 1 — Institutional review board table, Protocol and Statistical analysis plan. [file 41591_2025_4032_MOESM1_ESM.pdf]

nature medicine

Supplementary information https://doi.org/10.1038/s41591-025-04032-8

Adjuvant nivolumab and relatlimab in stage III/IV melanoma: the randomized phase 3 RELATIVITY-098 trial

In the format provided by the authors and unedited

Supplementary appendix

Adjuvant nivolumab and relatlimab in stage III/IV melanoma: the randomized phase 3 RELATIVITY-098 trial

G. V. Long, C. Garnett-Benson, S. Dolfi, P. A. Ascierto, J. Guo, A. A. Tarhini, S. Chandra, E. Muñoz-Couselo, M. Del Vecchio, A. C. de Melo, M. Callahan, H. Gogas, R. Dummer, D. Schadendorf, P. Köelblinger, G. Quereux, I. Thomas, J. X. Yu, A. Fisher, B. Wang, P. Djidel, A. Chouzy, M. Semaan, B. Chen, A. M.Y. Cheong, and H. A. Tawbi

Supplementary Table 1. Independent ethics committees and institutional review boards	Pages 2-39
Clinical trial protocol	Pages 40-234
Statistical analysis plan	Pages 235-327

Supplementary Table 1. Independent ethics committees and institutional review boards

Site No.	Principal Name	Name and Address of IRB/IEC and IRB/IEC chairpersons or FWA
CA224-098-0001[a]	Perfetti, Aldo Agustin, MD	Comite Independiente de Etica para<br>Ensayos en Farmacologia Clinica<br>774 Presidente Jose Evaristo Uriburu<br>1 er piso, Comuna 3<br>AAP, Distrito Federal, C1027<br>Argentina<br>Chairperson:<br>Luis, Zieher, MD
CA224-098-0002[a]	Cinat, Gabriela, MD	Comite de Etica en farmacologia Clinica<br>de la Funcdacion CIDEA<br>Paraguay 2035, 9 H<br>Buenos Aires, C1121ABE<br>Argentina<br><br>Chairpersons:<br>Modernell, Bargas, MD<br>Fabian, Claudio, MD
CA224-098-0003[a]	Fuentes, Christian Sebastian, MD	Comite Independiente de Etica para<br>Ensayos en Farmacologia Clinica del<br>Centro Medico Dra<br>De Salvo, 1548 Avenida Cabildo, 3 A<br>Colegiales, Comuna 13<br>Caba, Distrito Federal, 1426ABP<br>Argentina<br><br>Chairperson:<br>Jorge, Giannattasio, MD
CA224-098-0004[a]	Martin, Richardet, MD	Comite Institucional de Etica de<br>Investigacion en Salud<br>(CIEIS) del IONC<br>Chacabuco 716<br>Planta Alta<br>Barrio Nueva, Provincia de Corodoba<br>Argentina<br><br>Chairperson:

		Pacher, Maria E, MD
CA224-098-0005[a]	Pfluger, Yanina, MD	Comite de Etica en Investigacion Instituto Alexander Fleming<br>Cramer 1180, Ciudad Autonoma de<br>Buenos Aires, 1426<br>Argentina<br><br>Chairperson:<br>Jose, Mordoh, MD
CA224-098-0006[a]	Rojas, Carlos Ignacio, MD	Comite Etico Cientifico Bradford Hill<br>Las Hualtatas 9016<br>Santiago, Metropolitana<br>Chile<br><br>Chairperson:<br>Armando, Ortiz P, MD
CA224-098-0007[a]	Hernberg, Micaela, MD	*HUS* Tutkimuseettiset toimikunnat<br>Biomedicum<br>Helsinki 2 C, PL 705<br>Tukholmankatu 8 C<br>00029 HUS, Tukija<br>Ratapihantie 9, Helsinki, 00520<br>Finland<br><br>Chairperson:<br>Perola, Markus, MD
CA224-098-0009[a]	Hald, Sigurd, MD<br>Bjaanes, Gundersen, MH (Previous)<br>Henrik, Jespersen, MD (Previous)	REC Southeast<br>Gullhaugveien 1-3<br>Oslo, 0484<br>Norway<br>Chairperson:<br>Pal, Aukrust, MD
CA224-098-0010[a]	Ny, Lars, MD	Etikprovningsmyndigheten<br>Drottninggatan 4, Box 2110<br>Uppsala, 750 02<br>Sweden<br><br>Chairperson:<br>Peter, Stromberg, MD
CA224-098-0011[a]	Carneiro, Ana, MD	Etikprovningsmyndigheten<br>Box 2110<br>Uppsala, 750 02<br>Sweden<br><br>Chairperson:<br>Peter, Stromberg, MD
CA224-098-0012[a]	Helgadottir, Hildur, MD	Etikprovningsmyndigheten<br>Drottninggatan 4, Box 2110

		Uppsala, 750 02<br>Sweden<br><br>Chairperson:<br>Peter, Stromberg, MD
CA224-098-0013[a]	Vasquez, Rodrigo, MD<br>Salman, Pamela, MD<br>(Previous)	Comite Etico Cientifico<br>Bradford Hill<br>Las Hualtatas 9016, Vitacura<br>Santiago, Metropolitana<br>Chile<br>Chairperson:<br>Armando, Ortiz P, MD
CA224-098-0014[a]	Matamala, Luis A, MD	Comite Etico Cientifico FALP<br>Manuel Montt 427 piso 4<br>Providencia, RM, 7500000<br>Chile<br><br>Chairperson:<br>Veronica, Mackay A, MD
CA224-098-0015[a]	Sivonen, Veera, MD<br>Mattila, Kalle E, MD<br>(Previous)	Joint Authority Administration<br>Biomedicum Helsinki 2C, PL<br>705<br>Helsinki, Uusimaa, 00029<br>Finland<br><br>*HUS* Tutkimuseettiset<br>toimikunnat<br>Biomedicum<br>Helsinki 2 C, PL 705<br>Tukholmankatu 8 C<br>00029 HUS, Tukija<br>Ratapihantie 9, Helsinki,<br>00520<br>Finland<br><br>Chairperson:<br>Perola, Markus, MD
CA224-098-0017[a]	Winge-Main, Anna MD	REK South-east A<br>Gullhaugvein 1-3<br>Oslo, 0484<br>Norway<br>Chairperson:<br>Pal, Aukrust, MD
CA224-098-0018[a]	Svane, Inge Marie, MD	De Videnskabsetiske<br>Komitter for Region<br>Syddanmark<br>Damhaven 12<br>Regionshuset, Vejle, 7100<br>Denmark<br><br>Chairperson:<br>Ohm Kyvik, Kristen, MD

CA224-098-0019[a]	Skytta, Tanja, MD	*HUS* Tutkimuseettiset toimikunnat<br>Biomedicum<br>Helsinki 2 C, PL 705<br>Tukholmankatu 8 C<br>00029 HUS, Tukija<br>Ratapihantie 9, Helsinki, 00520<br>Finland<br><br>Chairperson:<br>Perola, Markus, MD
CA224-098-0020[a]	Bastholt, Lars, MD	De Videnskabsetiske Komitter for Region Syddanmark<br>Damhaven 12<br>Regionshuset, Vejle, 7100<br>Denmark<br><br>Chairperson:<br>Ohm Kyvik, Kristen, MD
CA224-098-0021[a]	Murillo Mayra, Graciela, MD	Comite de Etica en Investigacion de Investigacion biomedica para el<br>Desarrollo de Farmacos<br>SA, 3352 Calle Volcan Popocatepetl<br>Colli Urbano<br>Zapopan, Jalisco, 45070<br>Mexico<br>Comite de Investigacion de Investigacion<br>biomedica para el Desarrollo de Farmacos<br>SA de CV, 3352 Calle Volcan Popocatepetl<br>Colli Urbano<br>Zapopan, Jalisco, 45070<br>Mexico<br><br>Chairperson:<br>Mendez del Villar, Miriam, MD
CA224-098-0022[a]	Haslund, Charlotte, MD	De Videnskabsetiske Komitter for Region Syddanmark<br>Heden 16 St<br>Odense, Syddanmark, 5000<br>Denmark<br><br>Chairperson:<br>Ohm Kyvik, Kristen, MD

CA224-098-0023[a]	Hussain, Israr, MD	REK South-East A<br>Gullhaugvein 1-3<br>Oslo, 0484<br>Norway<br>Chairperson:<br>Pal, Aukrust, MD
CA224-098-0026[a]	Vantuchova, Yvetta, MD	Eticka Komise Fakultni<br>Nemocnice<br>Ostrava<br>17 Listopadu 1790/5<br>Ostrava, 70852<br>Czech Republic<br><br>Chairpersons:<br>Kacirkova, Ivana<br>Zelenik, Karol (Previous)
CA224-098-0027[a]	Hill, Andrew, MD	Bellberry Human Research<br>Ethics<br>Committee<br>123 Glen Osmond Road<br>Eastwood, South Australia,<br>5063<br>Australia<br><br>Chairperson:<br>Stoffell, Brian, MD
CA224-098-0028[a]	Atkinson, Victoria G,<br>MBBS FRACP	St Vincent's Hospital Human<br>Research<br>Ethics Committee<br>390 Victoria Street, de Lacey<br>Building,<br>Level 6<br>Darlinghurst, New South<br>Wales, 2010<br>Australia<br><br>Chairpersons:<br>Carland, Jane, MD<br>Brien, Jo-Anne, MD<br>(Previous)
CA224-098-0029[a]	Meniawy, Tarek, MD	Sir Charles Cairdner Hospital<br>Research<br>Governance Office<br>Hospital Avenue, Level 2, A<br>Block<br>Nedlands, Western Australia,<br>6009<br>Australia<br><br>St Vincent's Hospital Human<br>Research<br>Ethics Committee<br>97-105 Boundary Street<br>Darlinghurst, New South

		Wales, 2010<br>Australia<br><br>Chairperson:<br>Brien, Jo-Anne, MD
CA224-098-0030[a]	Van der Westhuizen, Andre, MBChB	St Vincent's Hospital Human Research<br>Ethics Committee<br>390 Victoria Street<br>de Lacey Building, Level 6<br>Darlinghurst, New South Wales, 2010<br>Australia<br><br>Chairperson:<br>Brien, Jo-Anne, MD
CA224-098-0031[a]	Sandhu, Shahneen, MBBS FRACP	St Vincent's Hospital Human Research<br>Ethics Committee<br>97-105 Boundary Street<br>Darlinghurst, New South Wales, 2010<br>Australia<br><br>Chairperson:<br>Brien, Jo-Anne, MD
CA224-098-0032[a]	Long, Georgina, MD	St Vincent's Hospital Human Research<br>Ethics Committee<br>Translational Research Centre<br>97-105 Boundary Street<br>Darlinghurst, New South Wales, 2010<br>Australia<br><br>Melanoma Institute of Australia<br>Governance<br>The Poche Centre, 40 Rocklands Road<br>North Sydney, New South Wales, 2060<br>Australia<br><br>Riverina Cancer Care Centre Research<br>Governance<br>31 Meurant Avenue<br>Wagga Wagga, New South Wales 2650<br>Australia<br><br>Chairperson:

		Carland, Jane, MD
CA224-098-0035[a]	Prithviraj, Prashanth, MD[b]	St Vincent's Hospital Human Research<br>Ethics Committee<br>97-105 Boundary Street<br>Darlinghurst, New South Wales, 2010<br>Australia<br><br>Chairperson:<br>Brien, Jo-Anne, MD
CA224-098-0039[a]	Quereux, Gaelle, MD	CPP Sud-Est III<br>59 Boulevard Pinel, Groupement<br>Hospitalier Est, Batiment Pinel<br>Bron, 69500<br>France<br><br>Chairperson:<br>Chapuis, Francois, MD
CA224-098-0040[a]	Robert, Caroline, MD	CPP Sud-Est III<br>59 Boulevard Pinel, Groupement<br>Hospitalier Est, GHE/Bat. Pinel<br>Bron, 69500<br>France<br><br>Chairperson:<br>Chapuis, Francois, MD
CA224-098-0041[a]	Dalle, Stephane, MD	CPP Sud-Est III<br>59 Boulevard Pinel, Groupement<br>Hospitalier Est, Batiment Pinel<br>Bron, 69500<br>France<br><br>Chairperson:<br>Chapuis, Francois, MD
CA224-098-0042[a]	Lebbe, Celeste, MD	CPP Sud-Est III<br>59 Boulevard Pinel, Groupement<br>Hospitalier Est, Batiment Pinel<br>Bron, 69500<br>France<br><br>Chairperson:<br>Chapuis, Francois, MD
CA224-098-0043[a]	Jeudy, Geraldine, MD<br>Dalac, Sophie, MD (Previous)	CPP Sud-Est III<br>59 Boulevard Pinel, Groupement

		Hospitalier Est, GHE/Bat. Pinel<br>Bron, 69500<br>France<br><br>Chairperson:<br>Chapuis, Francois, MD
CA224-098-0044[a]	Gaudy, Caroline, MD<br>Grob, Jean-Jacques, MD (Previous)	CPP Sud-Est III<br>59 Boulevard Pinel, Groupement<br>Hospitalier Est, Batiment Pinel<br>Bron, 69500<br>France<br><br>Chairperson:<br>Chapuis, Francois, MD
CA224-098-0046[a]	Pages Laurent, Cecile, MD<br>Meyer, Nicolas, MD (Previous)	CPP Sud-Est III<br>59 Boulevard Pinel, Groupement<br>Hospitalier Est, Batiment Pinel<br>Bron, 69500<br>France<br><br>Chairperson:<br>Chapuis, Francois, MD
CA224-098-0047[a]	Wainstein, Alberto Julius Alves, MD	Faculdade de Ciencias Medicas de Minas Gerias (FC-MG)<br>Alameda Ezequiel Dias N 275<br>Santa Efigenia<br>3 Andar Belo Horizonte<br>Minas Gerais, 30130-110<br>Brazil<br><br>Conep-Comissao Nacional De Etica E Pesquisa<br>SRTV 701, Via W 5 Norte, lote D,<br>Edificio PO 700, 3 andar, Asa Norte<br>Brasilia, Distrito Federal, 70719040<br>Brazil<br><br>Comite de Etica em Pesquisa do Hospital Lifecenter<br>Av do Contorno<br>4747 5 andar, Serra, Belo Horizonte

		Minas Gerais, 30110-921<br>Brazil (Previous)<br>Chairpersons:<br>Santos, Leila da Fatima, MD<br>Bonilha, Lais Alves de Souza, MD<br>Ferreira de Oliveira, Caroline, MD<br>(Previous)
CA224-098-0048[a]	Correa Tabajara Weiss, Bianca, MD	Comissao Nacional De Etica em Pesquisa<br>SRTV 701, Via W 5 Norte, lote D<br>Edificio PO 700, 3 andar, Asa Norte<br>Brasilia, Distrito Federal, 70719040<br>Brazil<br><br>Comite de Etica em Pesquisa Da Unisc<br>Av. Independencia 2293, Bloco 13 Sala<br>1306, 3717-7680<br>Santa Cruz do Sul, Rio Grande Do Sul, 96810110<br>Brazil<br><br>Chairperson:<br>Jorge, Venancio, MD<br>Renato, Nunes, MD
CA224-098-0049[a]	Menezes, Juliana de Janoski, MD	Comite de Etica Pesquisa Do Grupo<br>Hospitalar Conceicao<br>Av. Francisco Trein 596, Predio do<br>Ambulatorio, 3 andar<br>Porto Alegre, Rio Grande Do Sul, 91350-<br>200<br>Brazil<br><br>Conep - Comissao Nacional De Etica E<br>Pesquisa<br>SRTV 701, Via W 5 Norte, lote D,<br>Edificio PO 700, 3 andar, Asa Norte<br>Brasilia, Distrito Federal, 70719-040<br>Brazil

		Chairperson:<br>Bonilha, Lais, MD<br>Daniel, Faustino da S, MD
CA224-098-0056[a]	Franke, Fabio Andre, MD	Conep-Comissao Nacional De Etica E Pesquisa<br>SRTV 701, Via W 5 Norte, lote D<br>Edificio PO 700, 3 andar, Asa Norte<br>Brasilia, Distrito Federal, 70719-040<br>Brazil<br><br>Comite de Etica em Pesquisa da<br>Universidade Regional do Noroeste do<br>Estado do<br>3000 Bairro Universitario, Predio da<br>Biblioteca 2 Piso-Sala BIB 202-C<br>Ijui, Rio Grande do Sul, 98700-000<br>Brazil<br>Chairpersons:<br>Bonilha, Lais, MD<br>Aldemir, Berwig, MD<br>Jorge Alves de Almeida, Venancio, MD<br>(Previous)
CA224-098-0058[a]	Melo, Andreia Cristina de, MD	CEP-INCA<br>Rua do Resende, 128-Sala 203 Centro<br>Rio de Janeiro, 20231-092<br>Brazil<br>CONEP-Comissao Nacional de Etica em<br>Pesquisa<br>SEPN 510 NORTE, Bloco A, 3 andar,<br>Edificio Ex-INAN-Unidade II<br>Ministerio da Saude<br>Brasilia, Distrito Federal, 70750-521<br>Brazil<br>Chairperson:<br>Santa Rosa, Abilio Pereira A, MD

		Bonilha, Alves de Souza L, MD<br>Silva, Carlos Henrique, MD (Previous)
CA224-098-0062[a]	Krajsova, Ivana, MD	Eticka Komise Fakultni Nemocnice<br>Ostrava<br>17 Listopadu 1790/5<br>Ostrava, 70852<br>Czech Republic<br><br>Eticka komise Vseobecne fakultni<br>nemocnice v Praze<br>NA Bojisti 1<br>Praha 2, Prague, 12808<br>Czech Republic (Previous)<br>Chairpersons:<br>Kacirkova, Ivana<br>Zelenik, Karol, MD (Previous)<br>Zbynek, Sklenar, MD (Previous)
CA224-098-0064[a]	Ungureanu, Andrei, MD	Comisia Nationala de Bioetica a Medicamentului si a Dispozitivelor Medicale<br>Spital Colentina corp exterior C<br>Strada Dr. Grozovici nr 6, sector 2<br>Bucuresti, 20125<br>Romania<br>Comisia Nationala de Bioetica a Medicamentului<br>Sos. Stefan Cel Mare<br>Nr 19-21<br>Bucuresti, 20125<br>Romania<br><br>Chairpersons:<br>Draganescu, Doina F, MD<br>Dinu, Antonescu, MD
CA224-098-0065[a]	Ciuleanu, Tudor, MD[b]	Comisia Nationala de Bioetica a Medicamentului<br>Sos. Stefan Cel Mare<br>Nr 19-21<br>Bucuresti, 20125<br>Romania

		Chairperson:<br>Dinu, Antonescu, MD
CA224-098-0066[a]	Schenker, Michael, MD	Comisia Nationala de Bioetica a Medicamentului si a Dispozitivelor Medicale<br>Spitalul Colentina corp exterior C<br>Strada Dr. Grozovici Nr. 6, sector 2<br>Bucuresti, 020125<br>Romania<br>Comisia Nationala de Bioetica a Medicamentului si a Dispozitivelor Medicale<br>19-21 Soseaua Stefan cel Mare<br>Bucuresti, 020125<br>Romania (Previous)<br>Chairpersons:<br>Draganescu, Doina F, MD<br>Dinu, Antonescu, MD (Previous)
CA224-098-0068[a]	Vasconcellos, Vitor Fiorin de, MD	CIAS Unimed Vitoria<br>Rua Marins Alvarino 290-Itarare<br>Vitoria, Espirito Santo, 29047-660<br>Brazil<br><br>CONEP<br>SRTV 701, Via W 5 Norte Lote D<br>Edificio PO 700, 3 andar<br>Brasilia, Goias, 70719-04<br>Brazil<br>Chairpersons:<br>Bonilha, Alves de Souza L, MD<br>Largura Sessa, Sarti Karolyne, MD<br>Ribeiro, Zuqui Larissa, MD<br>Mainetti, Dayane Maciel B, MD<br>(Previous)

CA224-098-0070[a]	Hassan, Fernanda Menezes, MD	Comite de Etia em Pesquisa da Fundacao<br>Pio XII-Hospital de Cancer de barretos<br>Rua Antenor Duarte Vilella 1331<br>Barretos, Sao Paulo, 14784-400<br>Brazil<br>Conep - Comissao Nacional De Etica E<br>Pesquisa<br>SRTV 701, Via W 5 Norte<br>Lote D<br>Edificio PO 700, 3 andar Asa Norte<br>Brasilia, Distrito Federal, 70719-040<br>Brazil<br>Chairpersons:<br>Alves, Wilson Eduardo Fulan Matos, MD<br>Bonilha, Lais Alves de Souza, MD<br>Thiago, Silva, MD (Previous)
CA224-098-0071[a]	Lessa, Marco Antonio Oliveira, MD	Conep-Comissao Nacional De Etica E<br>Pesquisa<br>SRTV 701, Via W 5 Norte<br>Lote D<br>Edificio PO 700, 3 andar Asa Norte<br>Brasilia, Distrito Federal, 70719-040<br>Brazil<br><br>Research Ethics Committee of Hospital<br>Santo Antonio/Irma Dulce Social Works<br>Avenue Luiz Tarquinio S/N Gate 9<br>1st floor, room 1<br>Salvador, Bahia, 40414-120<br>Brazil<br><br>Chairpersons:<br>Bonilha, Lais, MD<br>Ignor de, Pinheiro M, MD
CA224-098-0072[a]	Santos, Fabio Nasser, MD	Conep- Comissao Nacional De Etica E

		Pesquisa<br>SRTV 701, Via W 5 Norte<br>Lote D<br>Edificio PO 700, 3 andar Asa Norte<br>Brasilia, Distrito Federal, 70719-040<br>Brazil<br>Hospital Sao Carlos CE<br>2571 Pontes Vieira Avenue, 5th floor<br>Fortaleza, Ceara, 60135237<br>Brazil<br>Chairpersons:<br>Lais Alves de Souza, Bonilha, MD<br>Rodrigues, Rogean N, MD
CA224-098-0073[a]	Mohr, Peter, MD	Ethik-Kommission der Medizinischen<br>Fakultat der Universitat Duisburg-Essen<br>Robert-Koch-Strasse 9-11<br>Essen, Nordrhein-Westfalen, 45147<br>Germany<br><br>Chairperson:<br>Schara-Schmidt, Ulrike, MD
CA224-098-0074[a]	Heppt, Markus, MD	Ethik-Kommission der Medizinischen<br>Fakultat der Universitat Duisburg-Essen<br>Robert-Koch-Strasse 9-11<br>Essen, Nordrhein-Westfalen, 45147<br>Germany<br><br>Chairperson:<br>Schara-Schmidt, Ulrike, MD
CA224-098-0075[a]	Hassel, Jessica Cecile, MD	Ethik-Kommission der Medizinischen<br>Fakultat der Universitat Duisburg-Essen<br>Robert-Koch-Strasse 9-11<br>Essen, Nordrhein-Westfalen, 45147<br>Germany<br><br>Chairperson:<br>Schara-Schmidt, Ulrike, MD
CA224-098-0076[a]	Heinzerling, Lucie, MD	Ethik-Kommission der Medizinischen

		Fakultat der Universitat<br>Duisburg-Essen<br>Robert-Koch-Strasse 9-11<br>Essen, Nordrhein-Westfalen, 45147<br>Germany<br><br>Chairperson:<br>Schara-Schmidt, Ulrike, MD
CA224-098-0077[a]	Meier, Friedegund, MD	Ethik-Kommission der Medizinischen<br>Fakultat der Universitat<br>Duisburg-Essen<br>Robert-Koch-Strasse 9-11<br>Essen, Nordrhein-Westfalen, 45147<br>Germany<br><br>Chairperson:<br>Schara-Schmidt, Ulrike, MD
CA224-098-0078[a]	Terheyden, Patrick, MD	Ethik-Kommission der Medizinischen<br>Fakultat der Universitat<br>Duisburg-Essen<br>Robert-Koch-Strasse 9-11<br>Essen, Nordrhein-Westfalen, 45147<br>Germany<br><br>Chairperson:<br>Schara-Schmidt, Ulrike, MD
CA224-098-0079[a]	Von Wasielewski, Imke, MD	Ethik-Kommission der Medizinischen<br>Fakultat der Universitat<br>Duisburg-Essen<br>Robert-Koch-Strasse 9-11<br>Essen, Nordrhein-Westfalen, 45147<br>Germany<br><br>Chairperson:<br>Schara-Schmidt, Ulrike, MD
CA224-098-0080[a]	Gesierich, Anja Heike, MD	Ethik-Kommission der Medizinischen<br>Fakultat der Universitat<br>Duisburg-Essen<br>Robert-Koch-Strasse 9-11<br>Essen, Nordrhein-Westfalen, 45147<br>German

		Chairperson:<br>Schara-Schmidt, Ulrike, MD
CA224-098-0081[a]	Sell, Sabine, MD	Ethik-Kommission der Medizinischen<br>Fakultat der Universitat Duisburg-Essen<br>Robert-Koch-Strasse 9-11<br>Essen, Nordrhein-Westfalen, 45147<br>Germany<br><br>Chairperson:<br>Schara-Schmidt, Ulrike, MD
CA224-098-0082[a]	Rohrer, Peter Michael, MD<br>Jost, Philipp, MD (Previous)<br><br>Pichler, Martin, MD (Previous)<br>Richtig, Erika, MD (Previous)	Ethikkommission der Medizinischen<br>Universitat Wien<br>Borschkegasse 8B/E06<br>Vienna, 1090<br>Austria<br><br>Chairperson:<br>Juergen, Zezula, MD
CA224-098-0083[a]	Gutzmer, Ralf, MD	Ethik-Kommission der Medizinischen<br>Fakultat der Universitat Duisburg-Essen<br>Robert-Koch-Strasse 9-11<br>Essen, Nordrhein-Westfalen, 45147<br>Germany<br><br>Chairperson:<br>Schara-Schmidt, Ulrike, MD
CA224-098-0084[a]	Hoeller, Christoph, MD	Ethikkommission der Medizinischen<br>Universitat Wien<br>Borschkegasse 8B/E06<br>Vienna, 1090<br>Austria<br><br>Chairperson:<br>Juergen, Zezula, MD
CA224-098-0085[a]	Laeubli, Heinz, MD	Kantonale Ethikkommission Zuerich<br>Stampfenbachstrasse 121<br>Zurich, 8090<br>Switzerland<br><br>Chairpersons:<br>Kleist, Peter, MD<br>Meier-Abt, Peter, MD (Previous)

CA224-098-0086[a]	Koelblinger, Peter, MD	Ethikkommission der Medizinischen Universitat Wien<br>Borschkegasse 8B/E06<br>Vienna, 1090<br>Austria<br><br>Chairperson:<br>Juergen, Zezula, MD
CA224-098-0087[a]	Von, Moss Roger, MD	Kantonale Ethikkommission Zurich<br>Stampfenbachstrasse 121<br>Zurich, 8090<br>Switzerland<br><br>Chairpersons:<br>Kleist, Peter, MD<br>Meier-Abt, Peter, MD (Previous)
CA224-098-0090[a]	Dummer, Reinhard, MD	Kantonale Ethikkommission Zuerich<br>Stampfenbachstrasse 121<br>Zurich, 8090<br>Switzerland<br><br>Chairpersons:<br>Kleist, Peter, MD<br>Meier-Abt, Peter, MD (Previous)
CA224-098-0091[a]	Kopecky, Jindrich, MD	Etica komise Fakultni nemocnice Ostrava<br>17 Listopadu 1790/5<br>Ostrava, 70852<br>Czech Republic<br><br>Etica komise FNHK<br>581 Sokolska Novy Hradec Kralove<br>Hradec Kralove, HK, 50005<br>Czech Republic (Previous)<br>Chairpersons:<br>Kacirkova, Ivana<br>Zelenik, Karol (Previous)<br>Vortel, Jiri, MD (Previous)
CA224-098-0092[a]	Leiter-Stoeppke, Ulrike, MD<br>Thomas, Ioannis, MD (Previous)	Ethik-Kommission der Medizinischen Fakultat der Universitat Duisburg-Essen<br>Robert-Koch-Strasse 9-11<br>Essen, Nordrhein-Westfalen, 45147<br>Germany

		Chairperson:<br>Schara-Schmidt, Ulrike, MD
CA224-098-0093[a]	Reinoso, Joaquin G, MD	Hospital La Mision<br>Av. del Hospital 112, 1 y 2 piso, Col.<br>Sertoma<br>Monterrey, Nuevo Leon, 64718<br>Mexico<br><br>Chairperson:<br>Islas Cisneros, Jose Francisco, MD
CA224-098-0098[a]	Bautista, Yolanda Lizbeth, MD	Investigacion Biomedica para el<br>Desarrollo de Farmacos<br>SA de CV, 5257 Avenida Sebastian Bach,<br>La Estancia<br>Zapopan, Jalisco, 45030<br>Mexico<br><br>Chairperson:<br>Mendez del Villar, Miriam, MD
CA224-098-0100[a]	Dip Abdel Karim, MD	Instituto Mexicano de Transplantes SC<br>580 Alta Tension, Cantarranas<br>Cuernavaca, Morelos, 62448<br>Mexico<br><br>Chairperson:<br>Sandra, Raya Santoyo, MD
CA224-098-0101[a]	Patel, Poulam, MRCP	East of England - Essex Research Ethics Committee<br>The Old Chapel, Royal Standard Place<br>Nottingham, NG1 6FS<br>United Kingdom<br><br>Chairperson:<br>Bannister, Niki, MD
CA224-098-0105[*]	Baurain, Jean-Francois, MD	Commissie Medische Ethiek UZ<br>Brussel/VUB<br>Laarbeeklaan 101

		Brussels, 1090<br>Belgium<br><br>Chairperson:<br>Michielson, Dirk, MD
CA224-098-0106[a]	Rutten, Annemie, MD	Commissie Medische Ethiek UZ<br>Brussel/VUB<br>Laarbeeklaan 101<br>Brussels, 1090<br>Belgium<br><br>Chairperson:<br>Michielson, Dirk, MD
CA224-098-0109[a]	Rorive, Andree, MD	Commissie Medische Ethiek UZ<br>Brussel/VUB<br>Laarbeeklaan 101<br>Brussels, 1090<br>Belgium<br><br>Chairperson:<br>Michielson, Dirk, MD
CA224-098-0110[a]	Neyns, Bart, MD	Commissie Medische Ethiek UZ<br>Brussel/VUB<br>Laarbeeklaan 101<br>Brussels, 1090<br>Belgium<br><br>Chairperson:<br>Michielson, Dirk, MD
CA224-098-0112[a]	Andreadis, Charalampos Ioannis, MD[A]	National Ethics Committee<br>284 Mesogeion Avenue<br>Cholargos, 15562<br>Greece<br>Chairperson:<br>Kolettis, Theofilos, MD
CA224-098-0113[a]	Bafaloukos, Dimitrios, MD	National Ethics Committee<br>284 Mesogeion Avenue<br>Cholargos, 15562<br>Greece<br><br>Chairperson:<br>Kolettis, Theofilos, MD
CA224-098-0114[a]	Gogas, Helen, MD	National Ethics Committee<br>284 Mesogeion Avenue<br>Cholargos, 15562<br>Greece<br><br>Chairperson:<br>Kolettis, Theofilos, MD

CA224-098-0115[a]	Del Vecchio, Michele, MD	Comitato Etico Territorale Lomabardia 4<br>Via G. Venezian 1<br>Milano, Lombardia, 20133<br>Italy<br><br>Comitato Etico INT Milano Istituto<br>Nazionale Dei Tumori, Via Venezian 1<br>Milano, 20133<br>Italy<br>Chairpersons:<br>Jankovic, Momcilo, MD<br>Apolene, Giovanni, MD (Previous)<br>La Bianca, Roberto, MD (Previous)
CA224-098-0116[a]	Bar-Sela, Gil, MD	Haemek EC<br>Yizhak rabin, building 8<br>Afula, 183400<br>Israel<br><br>Chairperson:<br>Goldstein, Lee Hilary, MD
CA224-098-0117[a]	Shapira Frommer, Ronnie, MD	IRB Sheba MC<br>derech sheba 2 Tel hashomer<br>Ramat Gan, 5265601<br>Israel<br><br>Chairperson:<br>Lidar, Merav, MD
CA224-098-0118[a]	Merims, Sharon, MD	Helsinki committee Hadassah M.C.<br>Kiryat Hadassah, Ein Karem<br>Jerusalem, 9112001<br>Israel<br><br>Chairperson:<br>Leitersdorf, Eran, MD
CA224-098-0119[a]	Ascierto, Paola A, MD	Comitato Etico Territorale Lomabardia 4<br>Via G. Venezian 1<br>Milano, Lombardia, 20133<br>Italy<br><br>Comitato etico<br>Via mariano semmola<br>Napoli, 80131<br>Italy<br>Chairpersons:

		Labianca, Roberto, MD<br>Daniele, Bruno, MD (Previous)<br>Franceso, Casavola, MD (Previous)
CA224-098-0120[a]	Pigozzo, Jacopo, MD<br>Chiarion-Sileni, Vanna, MD (Previous)	Comitato Etico Territorale Lomabardia 4<br>Via G. Venezian 1<br>Milano, Lombardia, 20133<br>Italy<br><br>Comitato etico Instituto Oncologico Veneto<br>Piazza Antenore 3, Palazzo Santo Stefano<br>Padova, Veneto, 35121<br>Italy<br><br>Chairpersons:<br>Labianco, Roberto, MD<br>Pegoraro, Renzo, MD (Previous)
CA224-098-0121[a]	Maio, Michele, MD	Comitato Etico Territorale Lomabardia 4<br>Via G. Venezian 1<br>Milano, Lombardia, 20133<br>Italy<br><br>Comitato Etico Regione Toscana Area Vasta Sud Est<br>16 Viale Mario Bracci<br>Siena, Provincia di Siena, 53100<br>Italy (Previous)<br><br>Chairpersons:<br>Jankovic, Momcilo, MD<br>Gonnelli, Stefano, MD (Previous)<br>Massimo, Alessandri, MD (Previous)
CA224-098-0122[a]	Mandala, Mario, MD	Comitato Etico Territorale Lomabardia 4<br>Via G. Venezian 1<br>Milano, Lombardia, 20133<br>Italy

		Comitato Etico Regionale dell Umbria<br>Ospedale S. Maria della Misericordia -<br>S. Andrea dellle Fratte<br>06156 Perugia, Presso CREO-Centro di<br>Ricerca Emato-Oncologico<br>Perugia, 06132<br>Italy (Previous)<br><br>Chairpersons:<br>Labianca, Roberto, MD<br>Maurizio, Caniglia, MD<br>(Previous)
CA224-098-0123[a]	De Marinis, Filippo, MD<br>Queirolo, Paola, MD	Comitato Etico Territorale Lomabardia<br>4<br>Via G. Venezian 1<br>Milano, Lombardia 20133<br>Italy<br><br>Comitato Etico degli IRCCS Instituto<br>Europeo di Oncologia e Centro<br>Cardiologica Monzino<br>Via Ripamonti 435<br>Milano, 20141<br>Italy<br>Chairpersons:<br>Labianca, Roberto, MD<br>Nonis, Atanasio, MD<br>(Previous)
CA224-098-0125	Kirkwood, John M, MD	WCG IRB<br>1019 39th Avenue Southeast, Suite 120<br>Puyallup, WA, 98374<br>USA<br><br>Chairperson:<br>Adekar, Sharad, MD
CA224-098-0127[a]	Wheater, Matthew, MD	East of England - Essex Research Ethics Committee<br>The Old Chapel, Royal Standard Place<br>Nottingham, NG1 6FS<br>United Kingdom<br><br>Chairperson:<br>Niki Bannister, MD

CA224-098-0130	Amin, Asim, MD	Advarra IRB<br>6100 Merriweather Drive, Suite 600<br>Columbia, MD, 21044<br>USA<br><br>Chairperson:<br>Westby, Christian, MD
CA224-098-0131[a]	Schadendorf, Dirk, MD	Ethik-Kommission der Medizinischen Fakultat der Universitat Duisburg-Essen<br>Robert-Koch-Strasse 9-11<br>Essen, Nordrhein-Westfalen, 45147<br>Germany<br><br>Chairperson:<br>Schara-Schmidt, Ulrike, MD
CA224-098-0133	Domingo-Musibay, Evidio, MD<br>Amatruda, Thomas, MD (Previous)	WCG IRB<br>1019 39th Avenue Southeast, Suite 120<br>Puyallup, WA, 98374<br>USA<br><br>Chairperson:<br>Adekar, Sharad, MD
CA224-098-0137	Jang, Sekwon, MD	WGC IRB<br>1019 39th Avenue Southeast Suite 120<br>Puyallup, WA, 98374<br>USA<br><br>Chairperson:<br>Adekar, Sharad, MD
CA224-098-0138	Medina, Theresa Michelle, MD	WCG IRB<br>212 Carnegie Center, Suite 301<br>Princeton, NJ 08540<br>USA<br><br>WCG IRB<br>1019 39th Avenue SE, Suite 120<br>Puyallup, WA, 98374<br>USA<br><br>Chairperson:<br>Adekar, Sharad, MD
CA224-098-0139	Pecora, Andrew, MD	WCG IRB<br>212 Carnegie Center, Suite 301

		Princeton, NJ 08540<br>USA<br>WCG IRB<br>1019 39th Ave S.E., Suite 120<br>Puyallup, WA, 98374<br>USA<br><br>Chairperson:<br>Adekar, Sharad, MD
CA224-098-0140	Mehnert, Janice, MD<br>Weber, Jeffrey S, MD (Previous)	NYU Grossman School of Medicine IRB<br>One Park Avenue, 6th Floor<br>New York, NY, 10016<br>USA<br><br>Chairperson:<br>Frederick, More, DDS
CA224-098-0141	Gastman, Brian, MD	Cleveland Clinic IRB<br>9500 Euclid Avenue, OS-I<br>Cleveland, OH, 44195<br>USA<br><br>Chairperson:<br>Lichtin, Alan, MD
CA224-098-0145	Salama, April K.S, MD	DUHS IRB<br>2424 Erwin Rd<br>4th Floor<br>Durham, NC, 27705<br>USA<br>FWA Number:<br>FWA 00009025
CA224-098-0146	Yorio, Jeffrey T, MD[b]	WCG IRB<br>1019 39th Ave. SE<br>Suite 120<br>Puyallup, WA, 98374<br>USA<br><br>Chairperson:<br>Adekar, Sharad MD
CA224-098-0147	Portnoy, David, MD	WCG IRB<br>1019 39th Avenue Southeast<br>Suite 120<br>Puyallup, WA, 98374<br>USA<br><br>Chairperson:<br>Adekar, Sharad MD
CA224-098-0148	Kounalakis, Nicole, MD	WCG IRB<br>1019 39th Avenue SE<br>Suite 120<br>Puyallup, WA, 98374-2115<br>USA

		Chairperson:<br>Adekar, Sharad MD
CA224-098-0150[a]	Rodas, Ivan Marquez, MD	CEim Hospital Gral. Univ, Gregorio<br>Maranon<br>Calle Maiquez 7, Madrid<br>Madrid, 28007<br>Spain<br><br>Chairperson:<br>Fernandez, Felipe Atienza, MD
CA224-098-0151[a]	Karmele, Mujika Eizmendi, MD	CEim Hospital Gral. Univ, Gregorio<br>Maranon<br>Calle Maiquez 7, Madrid<br>Madrid, 28007<br>Spain<br><br>Chairperson:<br>Felipe, Atienza Fernandez, MD<br>Ostrava, 70852<br>Czech Republic<br><br>Etica Komise MOU<br>Zluty kopec 7<br>Brno, 65653<br>Czech Republic (Previous)<br>Chairpersons:<br>Kacirkova, Ivana<br>Karol, Zelenik, MD (Previous)<br>Nemecek, Radim, MD (Previous)<br>Oldrich, Coufal, MD (Previous)
CA224-098-0158[a]	Miller, Wilson, MD	CIUSSS West-Central Montreal Research Ethics Board<br>3755, Chemin de la Cote-Ste-Catherine<br>Local A-925<br>Montreal, QC H3T 1E2<br>Canada<br><br>Chairpersons:<br>Margolese, Richard, MD<br>Bitzas, Vasiliki B, MD (Previous)

CA224-098-0159[a]	Jamal, Rahima, MD	CIUSSS West-Central Montreal Research Ethics Board<br>3755, Chemin de la Cote-Ste-Catherine<br>Local A-925<br>Montreal, QC, H3T 1E2<br>Canada<br><br>Chairperson:<br>Bitzas, Vasiliki B, MD
CA224-098-0160[a]	Claveau, Joel, MD	CIUSSS du Centre Ouest-de-l'Il-de<br>Montreal 3755, Chemin de la Cote-Ste-<br>Catherine<br>Montreal, QC, H3T 1E2<br>Canada<br><br>CIUSSS West - Central Montreal<br>Research Ethics Board<br>3755 Chemin de la Cote-Ste-Catherine<br>Local A-925<br>Montreal, QC, H3T 1E2<br>Canada (Previous)<br><br>Chairperson:<br>Margolese, Richard, MD<br>Bitzas, Vasiliki B, MD (Previous)
CA224-098-0162	Kim, Kevin, B, MD	WCG IRB<br>212 Carnegie Center, Suite 301<br>Princeton, New Jersey, 08540<br>USA<br><br>WCG IRB<br>1019 39th Avenue Southeast<br>Suite 120, Puyallup, WA, 98374<br>USA<br><br>Chairperson:<br>Adekar, Sharad MD
CA224-098-0163	Cowey, Charles L, MD	WCG IRB<br>1019 39th Ave. SE, Suite 120<br>Puyallup, WA, 98374<br>USA<br>Chairperson:<br>Adekar, Sharad MD

CA224-098-0164	Beck, Joseph Thaddeus, MD	WCG IRB<br>1019 39th Avenue SE, Suite 120<br>Puyallup, WA, 98374<br>USA<br><br>Chairperson:<br>Adekar, Sharad MD
CA224-098-0166	Gibney, Geoffrey T, MD	WCG IRB<br>1019 39th Avenue SE, Suite 120<br>Puyallup, WA, 98374<br>USA<br><br>Chairperson:<br>Adekar, Sharad MD
CA224-098-0170[a]	Sporis, Mariana, MD	Comisia Nationala de Bioetica a Medicamentului si a Dispozitivelor Medicale<br>19-21 Soseaua Stefan cel Mare<br>Bucuresti, 020125<br>Romania<br><br>Chairperson:<br>Dinu, Antonescu, MD
CA224-098-0171	Reuben, Daniel, MD[b]	WCG IRB<br>1019 39th Avenue SE, Suite 120<br>Puyallup, WA, 98374<br>USA<br><br>Chairperson:<br>Adekar, Sharad MD
CA224-098-0172	Tawbi, Hussein, MD	The University of Texas MD Anderson<br>Cancer Center Institutional Review Board<br>7007 Bertner Avenue, Unit #1637<br>Houston, Texas, 77030<br>USA<br>FWA Number:<br>FWA00000363
CA224-098-0175[a]	Weppler, Alison, MD	University of British Columbia<br>BC Cancer<br>Research Ethics Board<br>Fairmont Building<br>Suite 1315<br>750 West Broadway<br>Vancouver, BC, V5Z 1J3<br>Canada

		Chairperson:<br>Gelmon, Karen, MD
CA224-098-0176	Khalil, Maya, MD	WCG IRB<br>1019 39th Avenue SE, Suite 120<br>Puyallup, WA, 98374<br>USA<br><br>Chairperson:<br>Adekar, Sharad, MD
CA224-098-0177	Bajaj, Madhuri, MD	WCG IRB<br>1019 39th Avenue SE, Suite 120<br>Puyallup, WA, 98374<br>USA<br><br>Chairperson:<br>Adekar, Sharad, MD
CA224-098-0178	Doolittle, Gary, MD	WCG IRB<br>212 Carnegie Center, Suite 301<br>Princeton, NJ, 08540<br>USA<br><br>WCG IRB<br>1019 39th Avenue SE, Suite 120<br>Puyallup, WA, 98374<br>USA<br>Chairperson:<br>Adekar, Sharad
CA224-098-0179	Eldawy, Tarek, MD[b]	Ascension IRB<br>4600 Edmundson Rd<br>St. Louis, MO, 63134<br>USA<br><br>Sacred Heart Health Systems IRB<br>5151 North 9th Avenue, Northeast<br>Pensacola<br>Pensacola, FL, 32504<br>USA (Previous)<br>FWA Number:<br>FWA00015877
CA224-098-0183	Johnson, Douglas, MD[b]	Vanderbilt University Medical Center<br>3319 West End Avenue, Suite 600<br>Nashville, TN, 37203<br>USA<br><br>Chairperson:

		Peterson, Neeraja, MD
CA224-098-0185	Reddy, Sunil A, MD[b]	Administrative Panels on Human Subjects in Medical Research (Stanford IRBZ), Research Compliance Office, Stanford University 1705 El Camino Real, Suite 120 Palo Alto, CA, 94306 USA FWA Number: FWA00000935
CA224-098-0187[a]	Waterson, Ashita, MD<br>Tan, Yun Yi, MD (Previous)	East of England - Essex Research Ethics Committee The Old Chapel, Royal Standard Place Nottingham, NG1 6FS United Kingdom<br><br>Chairperson: Niki Bannister, MD
CA224-098-0188[a]	Gonzalez-Haba Martinez, Alba, MD	CEim Hospital Gral. Univ, Gregorio Maranon Calle Maiquez 7, Madrid Madrid, 28007 Spain<br><br>Chairperson: Fernandez, Felipe Atienza, MD
CA224-098-0191	Michael, Lowe, MD<br>Yushak, Melinda, MD (Previous)<br>Kudchadkar, Ragini, MD (Previous)	WCG Institutional Review Board 1019 39th Avenue Southeast, Suite 120 Puyallup, WA, 98374-2115 USA<br><br>Chairperson: Adekar, Sharad, MD
CA224-098-0197[a]	Macfarlane, Robyn, MD	Nova Scotia Health Research Ethics Board 5790 University Avenue Room 118 Halifax, NS, B3H 1V7 Canada<br><br>Chairperson: MacKnight Christopher, MD

CA224-098-0198[a]	Hanel, Robert, MD[b]	CIUSSS West-Central Montreal Research Ethics Board<br>3755, Chemin de la Cote-Ste-Catherine<br>Local A-925<br>Montreal, QC, H3T 1E2<br>Canada<br><br>Chairperson:<br>Bitzas, Vasiliki B, MD
CA224-098-0199[a]	Markel, Gal, MD	Helsinki Rabin<br>Zeev Jabotinsky Street<br>Nashim mertef C<br>Petah Tikva, 4941492<br>Israel<br><br>Chairperson:<br>Tur-Kaspa, Ran, MD
CA224-098-0200	Hamid, Omid, MD	WCG IRB<br>1019 39th Avenue SE, Suite 120<br>Puyallup, WA, 98374<br>USA<br><br>Chairperson:<br>Adekar, Sharad, MD
CA224-098-0201[a]	Munoz Couselo, Eva, MD	CEim Hospital Gral. Univ, Gregorio<br>Maranon<br>Calle Maiquez 7, Madrid<br>Madrid, 28007<br>Spain<br><br>Chairperson:<br>Fernandez, Felipe Atienza, MD
CA224-098-0202	Chandra, Sunandana, MD	Northwestern University Institutional Review Board<br>750 N Lake Shore Drive - 7th Floor<br>Chicago, IL, 60611<br>USA<br><br>Chairperson:<br>Gagnon, Christine, PhD
CA224-098-0206	Whitman, Eric, MD[A]	WCG IRB<br>1019 39th Avenue SE, Suite 120<br>Puyallup, WA, 98374<br>USA<br><br>Chairperson:<br>Adekar, Sharad, MD

CA224-098-0210	Smithy, James William, MD<br>Callahan, Margaret, MD<br>(Previous)	WCG IRB<br>212 Carnegie Center, Suite 301<br>Princeton, New Jersey, 08540<br>USA<br>WCG IRB<br>1019 39th Avenue SE, Suite 120<br>Puyallup, WA, 98374<br>USA<br>Chairperson:<br>Adekar, Sharad, MD
CA224-098-0214[a]	Guo, Jun, MD	Beijing Cancer Hospital Ethics Committee<br>52 Fucheng Road, Haidian District<br>Beijing, 100142<br>China<br>Chairperson:<br>Li, Jie, MD
CA224-098-0215[a]	Zou, Zhengyun, MD	Nanjing Drum Tower Hospital IEC/IRB<br>No.321 Zhongshan Road<br>First Floor of outpatient department<br>Nanjing, Jiangsu, 210008<br>China<br><br>Chairperson:<br>Yudong, Qiu, MD
CA224-098-0216[a]	Fang, Meiyu, MD[b]	Ethics Committee of Zhejiang Cancer<br>Hospital<br>No.1 East Banshan Road<br>Administrative Research Building<br>Hangzhou, Zhejiang, 310005<br>China<br>Chairperson:<br>Zhu, Liming, MD
CA224-098-0217[a]	Zhang, Xiaoshi, MD	Ethics committee of SUN YAT SEN<br>University Cancer Centre<br>Huatai Cuiyuan Building<br>Room 316<br>Guangzhou, Guangdong, 510060<br>China<br><br>Chairperson:

		Yuan, Zhongyu, MD<br>He, Yun, MD (Previous)
CA224-098-0219[a]	Wu, Di, MD	The First Hospital of Jilin University<br>Ethics Committee<br>No. 1, Xinmin Street<br>Changchun, Jilin, 130021<br>China<br>Chairperson:<br>Niu, Junqi, MD
CA224-098-0222[a]	Li, Ke, MD[b]	EC of Yunnan Cancer Hospital<br>No.519 Kunzhou Road<br>Jijiao Building<br>Floor 2<br>Kunming, Yunnan, 650106<br>China<br>Chairperson:<br>Liu, Zhimin, MD
CA224-098-0223	Babu, Sunil, MD[b]	WCG IRB<br>1019 39th Avenue SE, Suite 120<br>Puyallup, WA, 98374<br>USA<br>Chairperson:<br>Adekar, Sharad, MD
CA224-098-0224[a]	Liu, Hucheng, MD[b]	Ethics Committee of The First Affiliated<br>Hospital of Nanchang University<br>No.17, Yong Wai Zheng Street<br>The 9th Floor<br>General Building<br>Nanchang, Jiangxi, 330006<br>China<br><br>Chairperson:<br>Tan, Youwen, MD
CA224-098-0226[a]	Zhang, Weizhen, MD	Ethics Committee of Zhengzhou Third People's Hospital<br>No.136, Nanshuncheng Street<br>4F, 8th Building<br>Zhengzhou, Hunan, 450000<br>China<br>Chairperson:<br>Lee, Rui, MD
CA224-098-0228[a]	Jiang, Renbing, MD[b]	Affiliated Tumor Hospital of Xinjiang

		Medical University<br>No.789, Suzhou East Street<br>Xincheng District<br>3rd Floor of Complex Building<br>Urumqi, Xinjiang, 830000<br>China<br><br>Chairperson:<br>Zhang, Xiuhua, MD
CA224-098-0230[a]	Jiang, Yu, MD	Ethics Committee of West<br>China Hospital<br>Sichuan University<br>No.37 Guoxue Alley<br>Old Eight Teaching Building<br>Chengdu, Sichuan, 610041<br>China<br>Chairperson:<br>Yu, Yerong, MD
CA224-098-0232[a]	Payne, Mirinda, MD	East of England - Essex<br>Research Ethics<br>Committee<br>The Old Chapel, Royal<br>Standard Place<br>Nottingham, NG1 6FS<br>United Kingdom<br><br>Chairperson:<br>Niki Bannister, MD
CA224-098-0234[a]	Li, Xianan, MD	EC of Hunan Cancer Hospital<br>No.283 Tongzipo Road<br>Yuelu District<br>5F of Medical Technology<br>building<br>Changsha, Hunan, 410013<br>China<br>Chairperson:<br>Yu, Huayue, MD
CA224-098-0236	Chen, Yuanbin, MD	WCG IRB<br>1019 39th Avenue SE, Suite<br>120<br>Puyallup, WA, 98374<br>USA<br><br>Chairperson:<br>Adekar, Sharad, MD
CA224-098-0237[a]	Chen, Jing, MD	Union Hospital Tongji Medical<br>College<br>Huazhong University of<br>Science and<br>Technology

		No.1277, Jiefang Avenue<br>Room 402, No.7 Building<br>Wuhan, Hubei, 430022<br>China<br><br>Chairperson:<br>Hou, Xiaohua, MD
CA224-098-0239[a]	Zhang, Lihong, MD[b]	Ethics Committee of Drug Clinical Trials<br>of Huazhong University of Science and Technology<br>No.13, Hangkong Road, Hankou<br>Wuhan, Hubei, 430030<br>China<br>Chairperson:<br>Chen, Hui, MD
CA224-098-0241[a]	Wang, Enwen, MD	Medical Ethics Committee of Chongqing<br>Cancer Hospital<br>Ha Yu Lu<br>Chongqing, 400030<br>China<br>Chairperson:<br>Zhou, Hong, MD
CA224-098-0242[a]	Qu, Xiujuan, MD[b]	EC of the First Hospital of China Medical University<br>No.210 Baita 1st str<br>No.1, Building, 11th<br>Shenyang, Liaoning, 110001<br>China<br>Chairpersons:<br>Liu, Qunqing, MD<br>Qu, Shiwei, MD (Previous)
CA224-098-0243	Hernandez Aya, Leonel F, MD	University of Miami Human Subject<br>Research Office (HSRO) Institutional<br>Review Board<br>1320 South Dixie Highway, #650<br>Coral Gables, FL, 33146<br>USA<br><br>Chairperson:<br>Howard Landy, MD
CA224-098-0244[a]	Zhang, Junping, MD[b]	Medical Ethics Committee of Shanxi Bethune Hospital Drug Clinical Trial<br>No.99 Longcheng Dajie

		Taiyuan, Shanxi, 030032<br>China<br><br>Chairperson:<br>Liu, Xiansheng, MD
CA224-098-0245[a]	Chen, Yu, MD	Ethics Committee of Fujian Cancer<br>Hospital<br>No.420 Fuma Road<br>Jinan District<br>Fuzhou, Fujian, 350000<br>China<br><br>Chairperson:<br>Guo, Zhengqing, MD<br>He, Zhiyong, MD (Previous)
CA224-098-0250[a]	Roberts-Thomson, Rachel,<br>MD<br>Adelaide, South Australia, 5000<br>Australia<br><br>St Vincent's Hospital Human Research<br>Ethics Committee<br>390 Victoria Street, de Lacey Building,<br>Level 6<br>Darlinghurst, NSW, 2010<br>Australia<br><br>Chairpersons:<br>Carland Jane, MD<br>Brienne, Jo-Anne, MD<br>(Previous)	Local CALHN Research Services<br>136 North Terrace, Level 3, Roma<br>Mitchell Building
CA224-098-0251[a]	Warburton, Lydia, MD	SMHS RGO<br>14 Barry Marshall Parade<br>Murdoch, Western Australia, 6150<br>Australia<br><br>St Vincent's Hospital Human Research<br>Ethics Committee<br>97-105 Boundary Street<br>Darlinghurst, NSW, 2010<br>Australia<br><br>Chairperson:<br>Brien, Jo-Anne, MD
CA224-098-0252[a]	Carlino, Matteo, MD	Western Sydney Local Health District

		Human Research Ethics Committee<br>Corner Darcy Road & Hawkesbury Road,<br>REN Building Level 2<br>Westmead, NSW, 2145<br>Australia<br>St Vincent's Hospital Human Research<br>Ethics Committee<br>97-105 Boundary Street<br>Darlinghurst, NSW, 2010<br>Australia (Previous)<br><br>Chairperson:<br>Skapetis, Tony, MD<br>Brien, Jo-Anne, MD (Previous)
CA224-098-0253[a]	Kee, Damien, MD	Austin Health Human Research Ethics Committee<br>145 Studley Rd, Level 8, Harold Stokes Building<br>Heidelberg, Victoria, 3084<br>Australia<br><br>Austin Human Research Ethics Committee<br>145 Studley Road, Level 8, Harold Stokes Building<br>Heidelberg, Victoria, 3084<br>Australia<br><br>St Vincent's Hospital Human Research<br>Ethics Committee<br>97-105 Boundary Street<br>Darlinghurst, NSW, 2010<br>Australia<br><br>Chairperson:<br>Brien, Jo-Anne, MD
CA224-098-0257[a]	Pereira, Rodrigo, MD	Conep - Comissao Nacional De Etica E Pesquisa<br>SRTV 701<br>Via W 5 Norte, Lote D<br>Edificio PO 700, 3 andar<br>Brasillia, Distrito Federal,

		70719-040<br>Brazil<br><br>Comite de Etica em Pesquisa do Hospital<br>de Clinicas de Porto Alegre<br>2350 Rua Ramiro Barcelos, 2nd Floor,<br>Santa Cecilia<br>Porto Alegre, Rio Grande do Sul, 90035-<br>903<br>Brazil<br><br>Chairpersons:<br>Bonilha, Lais, MD<br>Felix, Temis Maria, MD<br>(Previous)
CA224-098-0258	Fescher, Leslie, MD	WCG IRB<br>1019 39th Avenue SE, Suite 120<br>Puyallup, WA, 98374<br>USA<br><br>Chairperson:<br>Adekar, Sharad, MD
CA224-098-0259[a]	Li, Hang, MD[b]	Biomedical Research Ethics Committee of<br>the First Hospital of Peking University<br>No.8 Xishiku Street<br>Xicheng District<br>Beijing, 100034<br>China<br><br>Chairperson:<br>Han, Xiaobin, MD
CA224-098-0261[a]	Niu, Xiaohui, MD	Beijing Jishuitan Hospital Ethics<br>Committee<br>No.31 Xinjiekou East Road<br>Xicheng District<br>4F, North Building<br>Beijing, 100035<br>China<br>Chairperson:<br>Li, Yumei, MD
CA224-098-0267	Nair, Suresh G, MD	Lehigh Valley Health Network<br>Institutional Review Board<br>1255 South Cedar Crest Boulevard<br>Suite 3200<br>Allentown, PA, 18103<br>USA

		Chairperson:<br>Leroy Kromis, MD
CA224-098-0268[a]	Chiaravalli, Stefano, MD	Comitato Etico della Fondazione<br>IRCCS Instituto Nazionale dei Tumori<br>Miloano<br>Via Giacomo Venezian<br>Milano, 20133<br>Italy<br><br>Chairperson:<br>Apolone, Giovanni, MD

[a]Non-IND sites. [b]Indicates sites with zero enrolment but drug shipped. FWA, federal-wide assurance; HREC, human research ethics committee; IEC, independent ethics committee; IND, investigational new drug; IRB, institutional review board.

CLINICAL PROTOCOL CA224098

A Phase 3, Randomized, Double-blind Study of Adjuvant Immunotherapy with Nivolumab + Relatlimab Fixed-dose Combination versus Nivolumab Monotherapy after Complete Resection of Stage III-IV Melanoma

Brief Title:

Adjuvant Immunotherapy with Nivolumab + Relatlimab Fixed-dose Combination versus Nivolumab Monotherapy after Complete Resection of Stage III-IV Melanoma (**RELA**TIVITY-098)

Protocol Amendment Number: 03
Incorporates Country-Specific Requirements for Countries in the European Union (EU)

[redacted]

24-hr Emergency Telephone Number
USA: 1-866-470-2267
International: +1-248-844-7390

Bristol-Myers Squibb Company
Route 206 & Province Line Road
Lawrenceville, NJ 08543

Avenue de Finlande 4
B-1420 Braine-l'Alleud, Belgium

REGULATORY AGENCY IDENTIFIER NUMBER(S)

IND: 136382

EUDRACT Number: 2021-001641-13

This document is the confidential and proprietary information of Bristol-Myers Squibb Company and its global affiliates (BMS). By reviewing this document, you agree to keep it confidential and to use and disclose it solely for the purpose of assessing whether your organization will participate in and/or the performance of the proposed BMS sponsored study. Any permitted disclosures will be made only on a confidential "need to know" basis within your organization or to your independent ethics committee(s). Any other use, copying, disclosure or dissemination of this information is strictly prohibited unless expressly authorized in writing by BMS. Any supplemental information (eg, amendments) that may be added to this document is also confidential and proprietary to BMS and must be kept in confidence in the same manner as the contents of this document. Any person who receives this document without due authorization from BMS is requested to return it to BMS or promptly destroy it. References to BMS in this protocol may apply to partners to which BMS has transferred obligations (eg, a Contract Research Organization [CRO]).

© 2023 Bristol-Myers Squibb Company

Approved v4.0 930169531 4.0

DOCUMENT HISTORY

Document	Date of Issue	Summary of Change
Protocol Amendment 03	27-Jul-2023	Main changes include:<br>• Updated and provided a more detailed endpoint description for the EQ-5D-5L index and visual analog scale assessment.<br>• Clarified the definition of the estimands and censoring rules for the supplemental estimand.<br>• Clarified timeframes for analysis for primary and secondary efficacy endpoints.<br>• Corrected typographical errors and included minor editorial updates in the Schedule of Activities, Biomarker Sampling Schedule, and Statistical Considerations sections.
Protocol Amendment 02	22-Feb-2023	Main changes include:<br>• Changed the trigger for interim RFS from time based to event based.<br>• Part of the secondary objective regarding outcomes on next line of therapy was moved to an exploratory objective. Duration on next line of therapy was moved to a new exploratory objective, and time to next line of therapy was added.<br>• Population for the distant metastasis-free survival (DMFS) secondary objective was limited to randomized participants with Stage III/IVA/IVB no evidence of disease (NED) melanoma.<br>• An additional clarification for neck magnetic resonance imaging (MRI) was provided for head and neck mucosal melanomas.<br>• To consolidate previously published/implemented country-specific amendments with the global amendment in preparation for the European Union Clinical Trials Regulation (EU CTR) transition, certain country-specific paragraphs were delineated while others were consolidated for easier reading without changing context.

Approved v4.0 930169531 4.0

Document	Date of Issue	Summary of Change
Protocol Amendment 01	18-May-2022	Major changes include:<br>• Addition of adolescent participants (≥ 12 years of age through < 18 years of age).<br>• An additional interim analysis for efficacy was added for the primary endpoint of recurrence-free survival (RFS). The analysis is planned 8 months after last participant is randomized (approximately 35 months after the first participant is randomized in the study).<br>• Distant metastasis-free survival was moved from key secondary endpoint to other secondary endpoint and consequently removed from the statistical testing hierarchy.<br>• Participants with a history of myocarditis, regardless of etiology, are excluded from participation in this study to prevent any potential added risk with study drug exposure; a requirement for baseline troponin testing was also added.<br>• Ultrasonography was updated as a study requirement for surveillance of participants who have a sentinel lymph node biopsy but do not undergo complete lymph node dissection (CLND) and left as optional for those with CLND.
Administrative Letter 04	24-Dec-2021	The "optional" label for the collection of pharmacokinetic samples during a drug-related Grade 3 or higher adverse event was inadvertently omitted from the protocol; this administrative letter corrects the omission.
Administrative Letter 02	12-Aug-2021	The Clinical Trial Physician of the protocol was changed.
Administrative Letter 01	28-May-2021	During finalization of the original protocol for CA224098, the IND and EUDRACT numbers were inadvertently left off both the cover page and the protocol signature page.<br>Additionally, it was noted that the acknowledgement page had a typo in the protocol number, which was incorrectly noted as CA2224098 with a superfluous "2."<br>This administrative letter corrects the IND/EUDRACT omission and the signature page typo.
Original Protocol	25-May-2021	Not applicable

Approved v4.0 930169531 4.0

OVERALL RATIONALE FOR PROTOCOL AMENDMENT 03

The primary reason for this protocol amendment is to clarify and ensure consistencies in the objectives and endpoints and statistical considerations sections. Additional minor changes include corrections in the schedule of activities, biomarker sampling schedule, and formatting updates.

SUMMARY OF KEY CHANGES FOR PROTOCOL AMENDMENT 03		
**Section Number & Title**	**Description of Change**	**Brief Rationale**
Protocol Summary	Text has been updated to align with changes made throughout the protocol.	Alignment between Protocol Summary and body of the protocol.
Table 2-2: On Study Treatment Procedural Outline (CA224098)<br>Table 2-3: Follow-up Assessments (CA224098)<br>Table 9.8-1: Biomarker Sampling Schedule: All Arms	• Corrected optional Biomarker Sample Collection to apply upon occurrence of ≥ Grade 3 drug-related AE.<br>• Corrected notes to state that biomarker sample collection is required at Follow-Up Visit 2 and then every 26 weeks (ie, at every alternating Survival Visit ± 90 days), but not to exceed 272 days between collections, thereafter to end of study or first recurrence, whichever occurs first.	• To correct typographical errors.<br>• To clarify the timing of the biomarker sample collection in relation to Survival Follow-up visits.
Table 4-1: Objectives and Endpoints	• Rephrased "next-line therapies" as "next-line systemic therapies" in the secondary objective to evaluate investigator-assessed outcomes, and as "next systemic therapies" in the exploratory objective to evaluate additional investigator-assessed outcomes, and deleted "next line of therapy" in the endpoint description for time to next treatment.<br>• Updated and provided a more accurate endpoint for the evaluation of participant's overall health status using the EQ-5D-5L index and visual analog scale objective.	To clarify and align with the intent of analysis.
Section 10.2.1: Recurrence-free Survival	• Corrected the nominal significance level for the first interim recurrence-free survival (RFS) analysis from 0.024 to 0.020.<br>• Corrected the critical hazard ratio for the final RFS analysis from 0.82 to 0.815.	• To correct a typographical error of the nominal significance level.<br>• To ensure the same precision (number of digits) is presented for the critical hazard ratio of the final RFS analysis across the document.

Approved v4.0 930169531 4.0

SUMMARY OF KEY CHANGES FOR PROTOCOL AMENDMENT 03		
**Section Number & Title**	**Description of Change**	**Brief Rationale**
Section 10.3: Analysis Sets	Clarified that the intercurrent event strategy for the analysis sets for main estimand of recurrence-free survival (RFS), distant metastasis-free survival (DMFS), and overall survival (OS) will be provided in Table 10.4.1-2.	To streamline the definitions for the analysis data sets.
Section 10.4.1: General Considerations	Statement regarding melanoma in situ not being considered a new primary melanoma moved to a footnote in Table 10.4.1-1.	To be consistent with the definition of RFS in Section 4.
Table 10.4.1-2: Definition of Estimands for Primary and Secondary Endpoints	• Clarified estimand definitions.<br>• Clarified the intercurrent event strategy for melanoma in situ, second non-melanoma primary cancer, and non-invasive basal cell carcinoma/squamous cell carcinoma with strategy equal to treatment policy.<br>• Updated population-level summaries for RFS, OS, and DMFS.<br>• Updated Intercurrent Events for PFS2 (Strategy) to state that details are presented in the statistical analysis plan.<br>• Added or updated Intercurrent Events (Strategy) for RFS, OS, DMFS, and safety.<br>• Removed summary statistics bullet as this will be further detailed in the statistical analysis plan.	To clarify and align with the intent of analysis and clarify that details are presented in the statistical analysis plan.
Table 10.4.1-2: Definition of Estimands for Primary and Secondary Endpoints<br>Section 10.4.2: Primary Endpoint(s)	Updated the phrase "secondary non-melanoma primary malignancy" to "second non-melanoma primary cancer").	To ensure accuracy and consistency of terminology.
Section 10.4.2: Primary Endpoints(s)	Clarified censoring rules for the supplemental estimand.	To clarify and align with the intent of analysis.
Table 10.4.2-1: Primary Endpoints<br>Table 10.4.3-1: Secondary Efficacy Endpoints	Clarified timeframes for analysis for primary and secondary efficacy endpoints.	To clarify and align with the intent of analysis.
Throughout	Editorial updates.	Minor; therefore, have not been summarized.

Approved v4.0 930169531 4.0

TABLE OF CONTENTS

TITLE PAGE 1
DOCUMENT HISTORY 3
OVERALL RATIONALE FOR PROTOCOL AMENDMENT 03 5
SUMMARY OF KEY CHANGES FOR PROTOCOL AMENDMENT 03 5
TABLE OF CONTENTS 7
1 PROTOCOL SUMMARY 11
2 SCHEDULE OF ACTIVITIES 19
3 INTRODUCTION 33
3.1 Study Rationale 34
3.1.1 Research Hypothesis 34
3.2 Background 35
3.2.1 Nivolumab Combined with Relatlimab Preclinical Activity 36
3.2.2 Nivolumab Combined with Relatlimab Clinical Activity 37
3.2.3 Nivolumab Clinical Activity 38
3.2.4 Relatlimab Mechanism of Action 39
3.2.5 Nivolumab Mechanism of Action 39
3.2.6 Nivolumab Combined with Relatlimab Clinical Safety 39
3.3 Benefit/Risk Assessment 41
3.3.1 Risk Assessment 43
3.3.2 Benefit Assessment 44
3.3.3 Overall Benefit/Risk Conclusion 44
4 OBJECTIVES AND ENDPOINTS 45
5 STUDY DESIGN 47
5.1 Overall Design 47
5.1.1 Data Monitoring Committee and Other Committees 49
5.2 Number of Participants 49
5.3 End of Study Definition 49
5.4 Scientific Rationale for Study Design 50
5.4.1 Participant Input Into Study Design 50
5.4.2 Rationale for Use of Nivolumab + Relatlimab Fixed Dose Combination 50
5.4.3 Rationale for Choice of Comparator 51
5.4.4 Rationale for Choice of Primary Endpoint 51
5.4.4.1 Rationale for Choice of Stratification Factors 51
5.4.5 Rationale for Blinding 52
5.4.6 Rationale for Evaluation of Biomarkers 52
5.4.7 Rationale for Inclusion of Adolescent Participants 53
5.4.8 Rationale for Ultrasound in Disease Surveillance 53
5.4.9 Rationale for MRI Surveillance of Head and Neck Mucosal Melanomas 54
5.5 Justification for Dose 54
5.5.1 Justification for Fixed Dose Combination Dosing 54
5.5.2 Dose Rationale for Adolescent Participants 55
5.5.3 Justification for Nivolumab Monotherapy Dose 56

Approved v4.0 930169531 4.0

5.5.4 Rationale for Continued Adjuvant Therapy for Patients Who Are Diagnosed with Melanoma in Situ During Study Treatment 57
5.6 Clinical Pharmacology Summary 57
5.6.1 Nivolumab Clinical Pharmacology Summary 57
5.6.2 Relatlimab Clinical Pharmacology Summary 58
6 STUDY POPULATION 59
6.1 Inclusion Criteria 59
6.2 Exclusion Criteria 62
6.3 Lifestyle Restrictions 64
6.4 Screen Failures 64
6.4.1 Retesting During Screening or Lead-in Period 65
7 STUDY INTERVENTION(S) AND CONCOMITANT THERAPY 65
7.1 Study Interventions Administered 66
7.1.1 Study Treatment Details 67
7.2 Method of Study Intervention Assignment 67
7.3 Blinding 68
7.4 Dosage Modification 69
7.4.1 Dose Delay Criteria 69
7.4.2 Criteria to Resume Treatment 76
7.4.3 Management of Algorithms for Immuno-Oncology Agents 76
7.4.4 Treatment of Infusion-Related Reactions 76
7.5 Preparation/Handling/Storage/Accountability 78
7.5.1 Retained Samples for Bioavailability/Bioequivalence/Biocomparability 78
7.6 Treatment Compliance 78
7.7 Concomitant Therapy 79
7.7.1 Prohibited and/or Restricted Treatments 79
7.7.1.1 Prohibited Treatments 79
7.7.2 Other Restrictions and Precautions 79
7.7.2.1 SARS-CoV-2 Vaccination Guidelines 79
7.7.2.2 Imaging Restriction and Precautions 80
7.8 Continued Access to Study Intervention After the End of the Study 80
8 DISCONTINUATION CRITERIA 80
8.1 Discontinuation From Study Treatment 80
8.1.1 Dose Discontinuation 81
8.1.2 Post-study Intervention Study Follow-up 82
8.2 Discontinuation From the Study 82
8.2.1 Individual Discontinuation Criteria 82
8.3 Lost to Follow-up 83
9 STUDY ASSESSMENTS AND PROCEDURES 83
9.1 Efficacy Assessments 84
9.1.1 Efficacy Assessment for the Study 84
9.1.2 Imaging Assessment for the Study 85
9.1.3 Investigator Assessment of Baseline Disease Status 86
9.1.4 Investigator Assessment of Recurrence 86
9.1.4.1 Unequivocal Recurrence 87
9.1.4.2 Equivocal Recurrence/Suspect Lesions 87

Approved v4.0 930169531 4.0

9.1.4.3 Definition of Recurrence 89
9.1.4.4 Date of Recurrence 89
9.1.5 Patient-Reported Outcomes 90
9.1.5.1 EORTC QLQ-C30 90
9.1.5.2 FACIT-GP5 91
9.1.5.3 EQ-5D-5L 91
9.2 Adverse Events 91
9.2.1 Time Period and Frequency for Collecting AE and SAE Information 92
9.2.2 Method of Detecting AEs and SAEs 93
9.2.3 Follow-up of AEs and SAEs 93
9.2.4 Regulatory Reporting Requirements for SAEs 93
9.2.5 Pregnancy 94
9.2.6 Laboratory Test Result Abnormalities 94
9.2.7 Potential Drug-induced Liver Injury 94
9.2.8 Other Safety Considerations 95
9.3 Overdose 95
9.4 Safety 95
9.4.1 Physical Examinations 95
9.4.2 Vital signs 95
9.4.3 Electrocardiograms 95
9.4.4 Clinical Safety Laboratory Assessments 95
9.4.5 Cardiac Evaluation 96
9.4.6 Imaging/Other Safety Assessment 97
9.5 Pharmacokinetics 97
9.6 Immunogenicity Assessments 99
9.7 Genetics 100
9.8 Biomarkers 100
9.8.1 Peripheral Blood Markers 103
9.8.1.1 Serum Biomarkers 103
9.8.1.2 Immunophenotyping 103
9.8.1.3 Whole Blood DNA/RNA Analysis 103
9.8.1.4 Circulating Tumor DNA Analysis and Other Plasma Biomarkers . 104
9.8.2 Tumor Samples 104
9.8.2.1 Tumor Sample Collection 104
9.8.2.2 LAG-3 and PD-L1 Expression 105
9.8.2.3 Characterization of Tumor-infiltrating Lymphocytes and Immune Biomarkers 105
9.8.2.4 Tumor Genotyping, Mutational Analysis, and Gene Expression Profiling 105
9.8.2.5 Tumor Markers Associated with Adverse Events 105
9.9 Additional Research 105
9.10 Other Assessments 107
9.11 Health Economics OR Medical Resource Utilization and Health Economics 107
10 STATISTICAL CONSIDERATIONS 107
10.1 Statistical Hypotheses 107

Approved v 4.0 930169531 4.0

10.1.1 Multiplicity Adjustment 108
10.2 Sample Size Determination 108
10.2.1 Recurrence-free Survival 108
10.2.2 Overall Survival 109
10.3 Analysis Sets 111
10.4 Statistical Analyses 111
10.4.1 General Considerations 112
10.4.2 Primary Endpoint(s) 115
10.4.3 Secondary Endpoint(s) 116
10.4.3.1 Safety Analysis 117
10.4.4 Exploratory Endpoint(s) 117
10.4.5 Other Safety Analysis 117
10.4.6 Other Analyses 118
10.4.6.1 Pharmacokinetic Analyses 118
10.4.6.2 Immunogenicity Analyses 118
10.4.6.3 Patient-Reported Outcomes 118
10.5 Interim Analyses 118
10.5.1 Interim Analyses of RFS 118
10.5.2 Interim Analysis of OS 119
10.5.3 Interim Analyses of RFS and OS: Additional Details 119
11 REFERENCES 120
12 APPENDICES 124
APPENDIX 1 ABBREVIATIONS AND TRADEMARKS 125
APPENDIX 2 STUDY GOVERNANCE CONSIDERATIONS 133
APPENDIX 3 ADVERSE EVENTS AND SERIOUS ADVERSE EVENTS: DEFINITIONS AND PROCEDURES FOR RECORDING, EVALUATING, FOLLOW-UP, AND REPORTING 144
APPENDIX 4 WOMEN OF CHILDBEARING POTENTIAL DEFINITIONS AND METHODS OF CONTRACEPTION 148
APPENDIX 5 MANAGEMENT ALGORITHMS FOR STUDIES UNDER CTCAE VERSION 5.0 152
APPENDIX 6 ECOG AND LANSKY/KARNOFSKY PERFORMANCE STATUS SCALE 161
APPENDIX 7 AJCC MELANOMA STAGING (CANCER STAGING MANUAL 8TH EDITION) 162
APPENDIX 8 MUCOSAL MELANOMA STAGING 166
APPENDIX 9 COUNTRY-SPECIFIC REQUIREMENTS 167
APPENDIX 10 PROTOCOL AMENDMENT SUMMARY OF CHANGE HISTORY 173

Approved v4.0 930169531 4.0

1 PROTOCOL SUMMARY

Protocol Title: A Phase 3, Randomized, Double-blind Study of Adjuvant Immunotherapy with Nivolumab + Relatlimab Fixed-dose Combination versus Nivolumab Monotherapy after Complete Resection of Stage III-IV Melanoma

Brief Title:

Adjuvant Immunotherapy with Nivolumab + Relatlimab Fixed-dose Combination versus Nivolumab Monotherapy after Complete Resection of Stage III-IV Melanoma (**RELA**TIVITY-098)

Rationale:

Surgical resection is currently considered standard of care for Stage III-IV resectable melanoma, followed by one year of adjuvant therapy. However, there remains an unmet need to improve recurrence-free survival (RFS) while maintaining a favorable safety profile for these patients. Targeting lymphocyte activation gene 3 (LAG-3), which is involved in the immune checkpoint pathway, is a novel approach that may further overcome immune evasion mechanisms.

Study CA224098 is a Phase 3, randomized, double-blind study of nivolumab and relatlimab (nivo + rela) fixed-dose combination (FDC) compared with nivolumab monotherapy in completely resected Stage III-IV melanoma. This study will generate efficacy and safety data for the patient population with Stage IIIA (> 1 mm tumor in lymph node)/B/C/D or Stage IV (no evidence of disease [NED]) melanoma following complete resection of their lesion(s).

Objectives and Endpoints:

Objectives	Endpoints
**Primary**	
• To compare the efficacy, as measured by RFS, provided by nivo + rela FDC vs nivolumab monotherapy in participants with completely resected Stage III/IV NED melanoma.	• RFS time as assessed by the investigator. RFS is defined as the time between the date of randomization and the first date of documented recurrence (local, regional, distant, new primary melanoma), or death due to any cause, whichever occurs first.
**Key Secondary**	
• To compare the OS provided by nivo + rela FDC vs nivolumab monotherapy in participants with completely resected Stage III/IV NED melanoma.	• OS is defined as the time between the date of randomization and the date of death due to any cause.
**Other Secondary**	
• To assess the efficacy as measured by DMFS, provided by nivo + rela FDC vs nivolumab monotherapy in participants with completely resected Stage III/IVA/IVB NED melanoma.	• DMFS, by investigator, is defined as the time between the date of randomization and the date of first distant metastasis or date of death due to any cause, whichever occurs first.
• To assess safety and toxicity of nivo + rela FDC and nivolumab monotherapy in participants with completely resected Stage III/IV NED melanoma.	• Incidence and severity of AEs, SAEs, IMAEs, drug-related AE/SAE, other select AEs, AEs leading to discontinuation, deaths, laboratory abnormalities.

Approved v4.0 930169531 4.0

Objectives	Endpoints
• To evaluate investigator-assessed outcomes on next-line systemic therapies.	• PFS2 defined as time from randomization to second recurrence/objective disease progression on next-line systemic therapy per investigator, or death from any cause, whichever occurs first.

Abbreviations: AE, adverse event; DMFS, distant metastasis-free survival; FDC, fixed dose combination; IMAE, immune mediated adverse event; NED, no evidence of disease; nivo, nivolumab; PFS2, progression-free survival 2; rela, relatlimab; RFS, recurrence free survival; OS, overall survival; SAE, serious adverse event.

Overall Design:

This is a Phase 3, randomized, double-blind study of nivo + rela FDC compared with nivolumab monotherapy in participants (≥ 12 years of age) with completely resected Stage IIIA (> 1 mm tumor in lymph node)/B/C/D or Stage IV NED melanoma by the American Joint Committee on Cancer, version 8 (AJCC v8). Efficacy of nivo + rela FDC, as measured by RFS time (as assessed by the investigator), will be compared to nivolumab monotherapy.

Participants will be randomized 1:1 to receive treatment with one of the following:

- Nivo + rela FDC dosing:
  - ≥ 18 years of age **OR** ≥ 12 years to < 18 years of age and ≥ 40 kg: nivolumab 480 mg and relatlimab 160 mg IV every 4 weeks (Q4W)
  - ≥ 12 years to < 18 years of age and < 40 kg: nivolumab 6 mg/kg and relatlimab 2 mg/kg IV Q4W
- Nivolumab dosing:
  - ≥ 18 years of age **OR** ≥ 12 years to < 18 years of age and ≥ 40 kg: nivolumab 480 mg IV Q4W
  - ≥ 12 years to < 18 years of age and < 40 kg: nivolumab 6 mg/kg IV Q4W

Participants will be stratified by AJCC v8 stage (Stage IIIA/IIIB vs IIIC vs IIID/IV [including all participants with mucosal melanoma, Stage III, Stage IVA, Stage IVB, and Stage IVC]), and geographic region (USA/Canada/Australia vs Europe vs rest of the world [ROW]). All participants will be treated until: recurrence of disease (excluding melanoma in situ), unacceptable toxicity, participant withdrawal of consent, or a maximum of 1 year of treatment from first dose (maximum of 13 doses), whichever occurs first.

This study will consist of 3 phases: screening, treatment, and follow-up.

The study design schematic is presented in Figure 1-1.

Approved v4.0 930169531 4.0

Figure 1-1: Study Design Schema

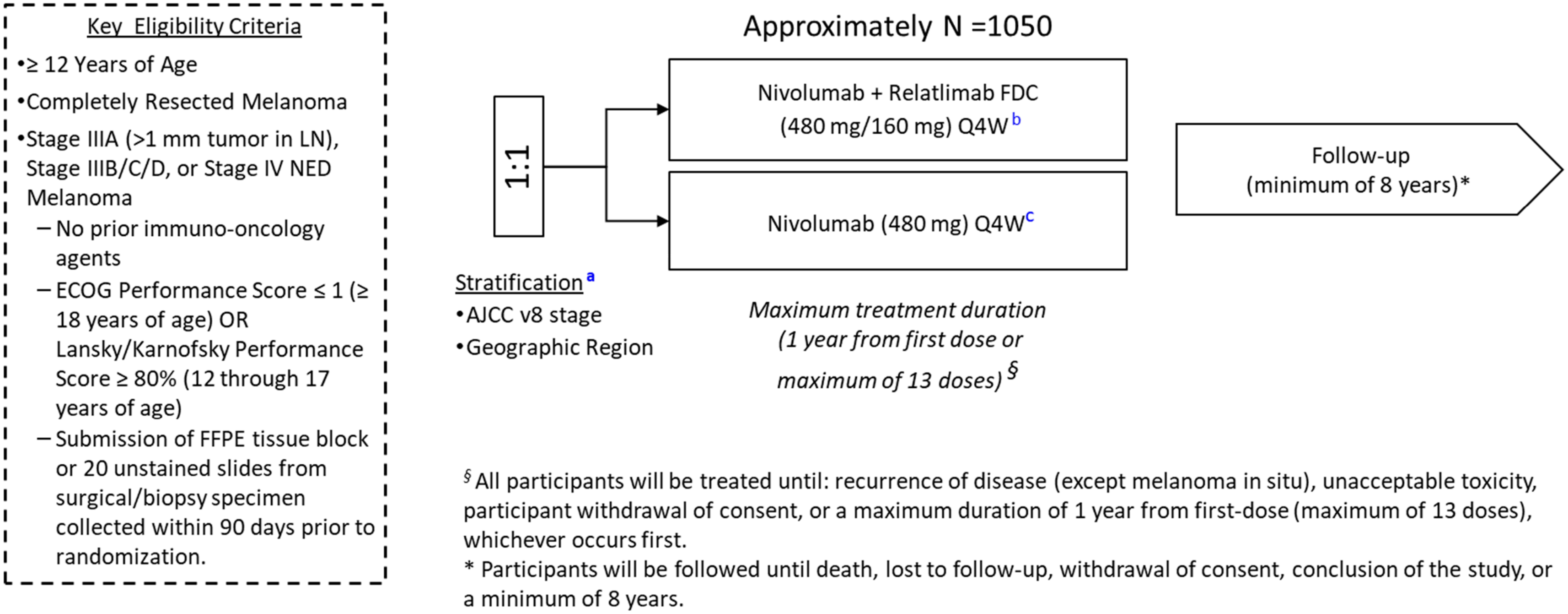

Abbreviations: AJCC v8, American Joint Committee on Cancer, version 8; ECOG, Eastern Cooperative Oncology Group; FDC, fixed dose combination; FFPE, formalin-fixed paraffin-embedded; IV, intravenous; LN, lymph node; NED, no evidence of disease; ROW, rest of the world; Q4W, every 4 weeks.

[a] Stratification:

- AJCC v8: Stages IIIA/IIIB, Stage IIIC, Stages IIID/IV (including all participants with mucosal melanoma, Stage III, Stage IVA, Stage IVB, and Stage IVC)
- Geographic Regions: USA/Canada/Australia, Europe, ROW

[b] Nivo + rela FDC dosing:

- ≥ 18 years of age **OR** ≥ 12 years to < 18 years of age and ≥ 40 kg: nivolumab 480 mg and relatlimab 160 mg IV Q4W
- ≥ 12 years to < 18 years of age and < 40 kg: nivolumab 6 mg/kg and relatlimab 2 mg/kg IV Q4W

[c] Nivolumab dosing:

- ≥ 18 years of age **OR** ≥ 12 years to < 18 years of age and ≥ 40 kg: nivolumab 480 mg IV Q4W
- ≥ 12 years to < 18 years of age and < 40 kg: nivolumab 6 mg/kg IV Q4W

Number of Participants:

Approximately 1,050 participants are expected to be randomized (1:1) to the following treatment arms:

- Nivo + rela FDC (n = 525)
- Nivolumab monotherapy (n = 525)

The randomization will be stratified by the following stratification factors:

- Geographic region (USA/Canada/Australia vs Europe vs ROW)
- AJCC v8 Stage IIIA/IIIB vs Stage IIIC vs Stage IIID/IV (including all participants with mucosal melanoma, Stage III, Stage IVA, Stage IVB, and Stage IVC)

Study Population:

Male and female participants ≥ 12 years of age with completely resected Stage IIIA (> 1 mm tumor in lymph node)/B/C/D or Stage IV NED melanoma.

Key Inclusion Criteria:

- All participants must have been diagnosed with either Stage IIIA (> 1 mm tumor in lymph node)/B/C/D or Stage IV melanoma by AJCC v8 and have histologically confirmed melanoma that is completely surgically resected (free of disease) with negative margins in order to be eligible. All melanomas, except ocular melanoma, regardless of primary site of disease, will be allowed.

  Note: Conjunctival melanoma is not considered to be ocular melanoma and is to be classified as mucosal melanoma.
- Participants are eligible if central nervous system (CNS) metastases have been resected and participants are neurologically stable.
  - Prior resected CNS metastases must be without evidence of recurrence, as determined by magnetic resonance imaging (MRI) performed at least 4 weeks after resection is complete and within 35 days prior to randomization.
  - Participants must be off immunosuppressive doses of systemic steroids (>10 mg/day prednisone or equivalent) for at least 14 days prior to study drug administration and must have returned to neurologic baseline post-operatively.
  - For CNS lesion(s), pathology report indicating that there has been complete resection of CNS lesion(s) will suffice as confirmation of negative margins.
- Complete resection must be performed within 90 days prior to randomization. Management of residual lymph nodes after positive sentinel lymph node biopsy (SLNB) (ie, completion lymph node dissection) will be as per local standards and recommendations for the individual participant.
- All participants must have disease-free status documented by a complete physical examination within 14 days prior to randomization and imaging studies within 35 days prior to

Approved v4.0 930169531 4.0

randomization. Imaging studies must include computed tomography (CT) scan of the chest, abdomen, pelvis, and all known sites of resected disease, and brain MRI.

- A formalin-fixed paraffin embedded (FFPE) tissue block (strongly preferred) containing 20 mm^3 of tumor tissue or of 20 positively charged unstained slides (minimum of 15) of tumor tissue obtained from surgical specimen or biopsy during resection (core biopsy, punch biopsy, or excisional biopsy) collected within 90 days prior to randomization, with no intervening systemic anti-cancer treatment between time of acquisition and enrollment, with an associated pathology report, must be submitted to the central laboratory (preferably prior to randomization). Fine needle aspirates or other cytology samples are not acceptable. Biopsies of bone lesions that do not have a soft tissue component are not acceptable. If insufficient tumor tissue content is provided for analysis, acquisition of additional tumor tissue (block and/or slides) for the biomarker analysis will be requested.
- Participants ≥ 18 years of age must have an Eastern Cooperative Oncology Group (ECOG) performance status ≤ 1. Adolescent participants between 12 and < 18 years of age must have a Lansky/Karnofsky performance score ≥ 80%.

Key Exclusion Criteria:

- History of ocular melanoma

  Note: Conjunctival melanoma is not considered to be ocular melanoma and is to be classified as mucosal melanoma
- Untreated/unresected CNS metastases or leptomeningeal metastases
- Participants with an active, known, or suspected autoimmune disease. Participants with type I diabetes mellitus, hypothyroidism only requiring hormone replacement, skin disorders (such as vitiligo, psoriasis, or alopecia) not requiring systemic treatment, or conditions not expected to recur in the absence of an external trigger are permitted to enroll.
- Participants with serious or uncontrolled medical disorder
- Previous severe acute respiratory syndrome coronavirus 2 (SARS-CoV-2) infection either suspected or confirmed within 4 weeks prior to screening. Acute symptoms must have resolved and based on investigator assessment, there are no sequelae that would place the participant at a higher risk of receiving investigational treatment.
- Concurrent non-melanoma malignancy (present during screening) requiring treatment or history of prior malignancy active within 2 years prior to randomization (ie, participants with a history of prior malignancy are eligible if treatment was completed at least 2 years before randomization and the participant has no evidence of disease). Participants with history of prior early stage basal/squamous cell skin cancer or non-invasive or in situ cancers that have undergone definitive treatment at any time are also eligible.
- Participants with a condition requiring systemic treatment with either corticosteroids (> 10 mg daily prednisone equivalent) within 14 days or other immunosuppressive medications within 30 days of start of study treatment. Inhaled or topical steroids, and adrenal replacement steroid doses > 10 mg daily prednisone equivalent, are permitted in the absence of active autoimmune disease.
- Participants with a history of myocarditis, regardless of etiology.

Approved v4.0 930169531 4.0

- Prior immunotherapy treatment for any prior malignancy: No prior immunotherapies are permitted (such as, but not limited to, anti-programmed death-1 (anti-PD-1), anti-programmed death ligand-1 (anti-PD-L1), anti-programmed death ligand-2 (PD-L2), or any other antibody or drug specifically targeting T-cell costimulation or immune checkpoint pathways).
- Prior treatment with LAG-3 targeted agents.
- Prior treatment with B-RAF proto-oncogene (BRAF)/mitogen-activated protein kinase (MEK)-targeted agents.
- Participants treated with anti-cancer therapy directed against the resected melanoma (for example but not limited to systemic, local, radiation, radiopharmaceuticals,) except:
  - Surgery for the melanoma lesion(s)
  - Adjuvant radiation therapy after neurosurgical resection for CNS lesions
  - Prior adjuvant interferon completed ≥ 6 weeks prior to randomization
- Treatment with any live/attenuated vaccine within 30 days of first study treatment (inactivated vaccines are permitted).
- Pregnant or breastfeeding females.
- Prior radiation therapy within 2 weeks prior to first dose of study medication. Participants must have recovered (ie, Grade ≤ 1 or at baseline) from radiation-related toxicities prior to first study treatment.
- Troponin T (TnT) or I (TnI) > 2 × institutional ULN. Participants with TnT or TnI levels between > 1 to 2 × ULN will be permitted if repeat levels within 24 hours are ≤ 1 × ULN. If TnT or TnI levels are between > 1 to 2 × ULN within 24 hours, the participant must undergo a cardiology consultation and cardiac evaluation and be considered for treatment, based on a favorable benefit/risk assessment by the Investigator. When repeat levels within 24 hours are not available, a repeat test should be conducted as soon as possible. If TnT or TnI repeat levels beyond 24 hours are < 2 × ULN, the participant must undergo a cardiology consultation and cardiac evaluation and be considered for treatment, based on a favorable benefit-risk assessment by the Investigator.

Intervention Groups and Duration:

As described in overall design.

Approved v4.0 930169531 4.0

Study Intervention:

Study Drug for CA224098		
**Medication**	**Potency**	**Investigational Product (IP)/Non-IP**
Nivo + rela FDC<br>(Nivolumab 240 mg/ Relatlimab 80 mg)/vial	16 mg/mL	IP
Nivolumab 100 mg/vial	10 mg/mL	IP

Abbreviations: FDC, fixed dose combination; IP, investigational product; nivo, nivolumab; rela, relatlimab.

Statistical Methods

- This is a double-blind, parallel group study in which participants will be randomized 1:1 to either of the 2 treatment groups: nivo + rela FDC or nivolumab monotherapy. The randomization will be stratified by AJCC v8 stage at screening and geographic region.
- The primary objective of the study is to assess the superiority of nivo + rela FDC over nivolumab monotherapy in improving RFS in participants with completely resected Stage IIIA (> 1 mm tumor in lymph node), Stage IIIB/C/D, or Stage IV NED melanoma over a treatment duration of 1 year. RFS distributions will be compared between the 2 treatment groups (nivo + rela FDC vs nivolumab monotherapy) using a 2-sided log-rank test stratified by AJCC stage at screening and geographic region.
- Two interim analyses for RFS will be performed based on number of events. The first interim RFS analysis (RFS IA#1) will be performed when approximately 309 RFS events have occurred (~75% information fraction). At that time, a minimum follow-up of 14 months is expected for all randomized participants (ie, approximately 28 months from first participant randomized in the study). If RFS IA#1 is not statistically significant, the second interim RFS analysis (RFS IA#2) will be performed when approximately 361 RFS events have occurred (~88% information fraction). At that time, a minimum follow-up of 24 months is expected for all randomized participants (ie, approximately 38 months from first participant randomized in the study).These interim analyses for RFS will allow for early formal testing for superiority, and the boundaries for declaring superiority will be derived based on the actual number of events using Lan-DeMets spending function with O'Brien and Fleming type of boundary in East® statistical software, based on the information fraction observed (based on 410 final RFS events).

Data Monitoring Committee: Yes

A Data Monitoring Committee will be used in the study.

Approved v4.0 930169531 4.0

Other Committee: Yes

A Study Steering Committee will be used in the study.

Brief Summary:

The purpose of this study is to assess the efficacy, measured as RFS, of nivo + rela FDC compared with nivolumab monotherapy, in the setting of adjuvant treatment of Stage III-IV melanoma after complete resection. Study details are as follows.

Study drug will be given for a maximum of 1 year from first dose (maximum of 13 doses), and participants will be followed for a minimum of 8 years.

While study drug is being administered, study visits will be every 4 weeks (for a maximum of 1 year). Once study intervention is completed or discontinued early, the first follow-up visit will be 30 days from the date of the last dose. The second follow-up visit is 135 days following last dose of study intervention. Long-term follow up visits will be every 12 weeks subsequently (in clinic or by telephone) for a minimum of 8 years or until a participant meets end-of-study criteria. Additional follow-up may continue for up to 5 years from the time of the final OS analysis. The trial will end once this follow-up has concluded. In-person biomarker collections (ctDNA) are required every 26 weeks, but not to exceed 272 days between collections, during long-term follow up until end of study or first recurrence, whichever occurs first. Biomarker collections should be stopped for participants who start on subsequent systemic therapy.

Approved v4.0 930169531 4.0

2 SCHEDULE OF ACTIVITIES

Table 2-1: Screening Procedural Outline (CA224098)

Procedure	Screening	Notes: [a]<br>All windows are based on calendar days.
**Eligibility Assessments**		
Informed Consent	X	Must be obtained prior to performing any screening procedures. See Section 6.1.<br>For adolescent participants (≥ 12 years to < 18 years), please refer to Informed Consent Process in Appendix 2. Where local regulations do not allow for participants < 18 years of age (adolescent population) to participate, the eligible participant population is ≥ 18 years of age (refer to Appendix 9).<br>Study allows for re-enrollment of a participant that has discontinued the study as a pre-treatment failure. If re-enrolled, the participant must be re-consented and assigned a new participant number from IRT.
Enroll in IRT	X	Register in IRT system to obtain participant number.
Inclusion/Exclusion Criteria	X	Assessed at screening and must be confirmed prior to randomization.
Medical History	X	All medical history relevant to the disease under study.<br>Document all available BRAF, NRAS, and KIT results.
Tumor Sample Submission	X	A FFPE tissue block (strongly preferred) containing 20 mm^3 of tumor tissue or 20 positively charged unstained slides (minimum of 15) of tumor tissue obtained from surgical specimen or biopsy during resection (core biopsy, punch biopsy, or excisional biopsy), collected within 90 days prior to randomization, with no intervening systemic anti-cancer treatment between time of acquisition and enrollment, with an associated pathology report, must be submitted to the central laboratory (preferably prior to randomization). Fine needle aspirates or other cytology samples are not acceptable. Biopsies of bone lesions that do not have a soft tissue component are not acceptable.<br>If insufficient tumor tissue content is provided for analysis, additional tumor tissue (block and/or slides) for the biomarker analysis will be requested.<br>Please refer to Section 9.8, the Laboratory Manual, and Tissue Collection Instructions for complete details.

Table 2-1: Screening Procedural Outline (CA224098)

**Procedure**	**Screening**	**Notes:** [a] **All windows are based on calendar days.**
Review of Pathology Report	X	Diagnosis must be stated in a pathology report confirming negative margins that has been finalized prior to randomization. A deidentified copy of the report must be provided to the central lab.
Body Imaging	X	Contrast-enhanced CT of the chest, abdomen, pelvis, and all suspected sites of disease, within 35 days prior to randomization. See Section 9.1.2 for further details and exceptions. Please refer to Appendix 9 for Germany-specific imaging language.
Brain Imaging	X	MRI of the brain (with and without contrast) is required for ALL participants during screening to rule out brain metastases, within 35 days prior to randomization. CT of the brain (with and without contrast) can be performed if MRI is contraindicated. See Section 9.1.2 for further details.
Ultrasound of Region(s) relevant to the Participant's Tumor Resection	X	Ultrasound of region(s) relevant to the participant's tumor resection must be performed for surveillance of participants who have a positive SNLB who do not undergo immediate CLND. Ultrasound is optional for participants who have undergone CLND relevant to the tumor resection.<br>All suspicious lesion(s) should undergo proper evaluation. If disease is detected, all lesions must be removed and NED must be confirmed within 35 days prior to randomization.<br>Ultrasounds should not be submitted to the imaging vendor.
**Safety Assessments**		
Complete Physical Examination, Measurements, Vital signs	X	Complete physical exam including height, weight, blood pressure, heart rate, and temperature. Must be collected within 14 days prior to randomization.
Performance Status	X	Within 14 days prior to randomization. ECOG Performance Status (≥ 18 years old) OR Lansky/Karnofsky Performance Score for adolescents (12 to < 18 years old) (Appendix 6).
Assessment of Signs and Symptoms	X	Within 14 days prior to randomization.
Concomitant Medications	X	Within 14 days prior to randomization.<br>Confirm no live vaccine use within 30 days prior to first study treatment.

Table 2-1: Screening Procedural Outline (CA224098)

Procedure	Screening	Notes: [a] All windows are based on calendar days.
SAE Assessment	X	SAEs collected from time of consent.<br>All AEs (SAEs or non-serious AEs) associated with SARS-CoV-2 infection collected from time of consent.
12-Lead ECG	X	12-lead ECGs should be recorded after the participant has been supine for at least 5 minutes. Must be performed within 14 days prior to randomization. ECG abnormalities should be reviewed and addressed, if needed, prior to randomization.
**Laboratory Tests**		
Clinical Laboratory Assessments	X	Laboratory tests must be performed within 14 days prior to randomization.<br>Viral testing to be completed within 35 days prior to randomization. For HIV: testing at sites where locally mandated (see Appendix 9).<br>Refer to Section 9.4.4 for list of laboratory tests to conduct.
Pregnancy Test (WOCBP)	X	Serum or urine pregnancy test (minimum sensitivity equivalent units 25 IU/L or equivalent units of HCG) to be done within 35 days prior to randomization.
FSH	X	Females under the age of 55 years must have a serum FSH level > 40 mIU/mL to confirm menopause. If not confirmed, protocol pregnancy testing requirements must be followed.
Cardiac Troponin (T or I)	X	Within 14 days prior to randomization.<br>TnT or TnI between > 1 to 2× ULN will be permitted if a repeat assessment within 24 hours remains < 2× ULN and participant undergoes a cardiology consultation and cardiac evaluation as described in Section 9.4.5.
**GERMANY ONLY:**<br>CPK	See Notes	**GERMANY ONLY:**<br>Within 14 days prior to randomization.
**Biomarker Assessments**		
Biomarker Sample Collection	See Table 9.8-1 for further details.	

Abbreviations: AE, adverse event; BRAF, B-RAF proto-oncogene; CLND, complete lymph node dissection; CPK, creatine phosphokinase; eCRF, electronic case report form; CT, computed tomography; ECG(s), electrocardiogram(s); ECOG, Eastern Cooperative Oncology Group; FFPE, formalin-fixed paraffin-embedded; FSH, follicle-stimulating hormone; HCG, human chorionic gonadotropin; HIV, human immunodeficiency virus; IRT, interactive response technology; MRI,

Approved v4.0 930169531 4.0

magnetic resonance imaging; NED, no evidence of disease; NRAS, neuroblastoma ras viral oncogene homolog; SAE(s), serious adverse event(s); SARS-CoV-2, severe acute respiratory syndrome coronavirus 2; SNLB, sentinel lymph node biopsy; TnI, troponin I; TnT, troponin T; ULN, upper limit of normal; WOCBP, women of child-bearing potential.

[a] Some of the assessments referred to in this section may not be captured as data in the eCRF. They are intended to be used as safety monitoring by the treating physician. Additional testing or assessments may be performed as clinically necessary or where required by institutional or local regulations.

Approved v4.0 930169531 4.0

Table 2-2: On Study Treatment Procedural Outline (CA224098)

Procedure	C1D1[a] (Cycle Duration = 4 weeks)	C2D1 and D1 of Each Subsequent Cycle (± 3 days)	Notes:[b]
**Study Treatment**			
Randomize	X		
IRT Drug Assignment	X		Participant must receive the first dose of study medication within 3 days from randomization.
Dispense Study Treatment	X	X	First drug to be administered within 3 calendar days following randomization, subsequent doses can be administered within 3 days before or after scheduled date if necessary (every 28 days ± 3 days). Treatment will continue until recurrence, unacceptable toxicity, withdrawal of consent, or after a maximum duration of 1 year from first-dose (maximum of 13 doses), whichever occurs first.<br>See treatment details in Section 7.1.1.
**Safety Assessment**			
Targeted Physical Examination, Measurements, and Vital Signs[c]	X*	X	Weight, blood pressure, heart rate, and temperature within 3 days prior to dosing.<br>*Targeted physical exam not required on C1D1 if a full physical exam was completed within 3 days of first dose.
Performance Status[c]	X	X	ECOG Performance Status (≥ 18 years old) OR Lansky/Karnofsky Performance Score for adolescents (12 to < 18 years old). See Appendix 6.
AE and SAE Assessment	Continuously		Record at each visit. All AEs (SAEs or non-serious AEs), including those associated with SARS-CoV-2 infection, must be collected continuously during the treatment period.
Concomitant Medications	Continuously		Record at each visit.
**GERMANY ONLY:**<br>Office Visit/Clinical Observation	See Notes		**GERMANY ONLY:**<br>Study visits/evaluations will occur weekly (± 1 day) to assess safety for the first 12 weeks of treatment (up to and including C3D22), regardless of dose delays. Thereafter, visits will only be on Day 1 of each subsequent cycle. All participants should be clinically evaluated for any IMAEs. Participants with any clinical symptoms, in particular fatigue, should immediately be evaluated.

Table 2-2: On Study Treatment Procedural Outline (CA224098)

Procedure	C1D1[a] (Cycle Duration = 4 weeks)	C2D1 and D1 of Each Subsequent Cycle (± 3 days)	Notes:[b]
**Laboratory Tests**			
Pregnancy Test (WOCBP)	X*	X^	Serum or urine (minimum sensitivity equivalent units 25 IU/L or equivalent units of HCG); a negative pregnancy test should be documented within 1 day prior to administration of first dose of study treatment. For Norway, see Appendix 9 for specific requirements.<br>*Pregnancy test not required to be repeated on C1D1 if completed within 24 hours of first dose (during screening).<br>^For subsequent cycles, a negative pregnancy test should be documented within 3 days prior to dosing.
Clinical Laboratory Assessments[c]	X	X	Laboratory testing should be performed within 3 days prior to each dose.<br>For the first treatment visit, labs need not be repeated if they were performed within 3 days and the results are available and have been reviewed for eligibility.<br>Refer to Section 9.4.4 for the list of laboratory tests to be conducted.
**GERMANY ONLY:** Troponin and CPK	See Notes		**GERMANY ONLY:**<br>Troponin and CPK evaluated at predose on C1D1* with weekly evaluations (± 1 day) up to and including Week 12 (C3D22), regardless of dose delays.<br>*Values taken at screening (prerandomization) may be used and need not be repeated.<br>All results must be checked prior to the next infusion. Symptom-based troponin testing may be performed as clinically indicated, thereafter.<br>For elevated troponin, please refer to Section 9.4.5 and the IMAE management algorithms in Appendix 5.

Approved v4.0 930169531 4.0

Table 2-2: On Study Treatment Procedural Outline (CA224098)

Procedure	C1D1[a] (Cycle Duration = 4 weeks)	C2D1 and D1 of Each Subsequent Cycle (± 3 days)	Notes:[b]
**Efficacy Surveillance**			
Body Imaging	See Notes		Contrast-enhanced CT of the chest, abdomen, pelvis, and all other suspected sites of disease should occur every 12 weeks (± 7 days) from randomization for the first 2 years and every 26 weeks (± 14 days) beyond the Week 108 imaging time point thereafter until investigator assessed local, regional, or distant recurrence (whichever comes first) for Stage IV participants and until distant recurrence for Stage III participants. For mucosal melanoma participants, assessment should continue until investigator assessed local, regional, or distant recurrence for Stage M1, and until distant recurrence for M0 participants. See Section 9.1.2 for further details and exceptions.<br>In case of suspected lesions in the extremities, contrast-enhanced MRI can be substituted for contrast-enhanced CT.<br>For head and neck mucosal melanomas, contrast-enhanced MRI of the head and neck is required at every time point for nodal surveillance.<br>Please refer to Appendix 9 for Germany-specific imaging language.
Brain Imaging	See Notes		Participants with a history of brain metastasis or symptoms should have a surveillance MRI (without and with contrast) approximately every 12 weeks ± 7 days from randomization for the first 2 years and every 26 weeks (± 14 days) beyond the Week 108 imaging time point thereafter until investigator assessed local, regional, or distant recurrence (whichever comes first) for Stage IV participants or sooner if clinically indicated. Participants without history of brain metastases should have MRI if clinically indicated. See Section 9.1.2 for further details.

Approved v4.0 930169531 4.0

Table 2-2: On Study Treatment Procedural Outline (CA224098)

Procedure	C1D1[a] (Cycle Duration = 4 weeks)	C2D1 and D1 of Each Subsequent Cycle (± 3 days)	Notes:[b]
Ultrasound of Region(s) relevant to the Participant's Tumor Resection	See Notes		For participants without CLND, active surveillance using ultrasound of region(s) relevant to the participant's tumor resection must occur every 12 weeks (± 7 days) from randomization for the first 2 years and every 26 weeks (± 14 days) beyond the Week 108 imaging time point thereafter until disease recurrence.<br>Ultrasound is not required for head and neck mucosal participants.<br>Ultrasound is optional for participants who have undergone CLND relevant to the tumor resection.<br>Ultrasounds should not be submitted to the imaging vendor.
**Health Outcomes Assessments**			
EORTC QLQ-C30	X	X	To be administered using eCOA at the site. Each assessment must be completed prior to the start of dosing and preferably as the first procedure on the day of dosing.<br>Adolescent participants (≥ 12 and < 18 years of age) only complete the EQ-5D-5L. Adolescent participants will continue to complete only the EQ-5D-5L even if they become ≥ 18 years of age during treatment or follow-up.<br>If Health Outcomes Assessments are collected but the dose is subsequently delayed, a data change form should be submitted to move the original data entry to an unscheduled visit.
FACIT-GP5	X	X	
EQ-5D-5L	X	X	
Healthcare Resource Utilization	X	X	Health care resource utilization data will be collected at each visit by study site staff and reported on an eCRF collection page. See details of data to be collected in Section 9.11.
**Biomarker Assessments**			
SARS-CoV-2 Serology	X		
Tumor Tissue Sample Collection (If Medically Feasible)	At disease recurrence (Table 9.8-1)		If biopsy or surgical resection is performed at recurrence, a tumor sample (a FFPE block or 20 positively charged unstained slides) must be submitted to central laboratory within 30 days of collection.<br>Fine needle aspirates or other cytology samples are not acceptable. Biopsies of bone lesions that do not have a soft tissue component are not acceptable.

Table 2-2: On Study Treatment Procedural Outline (CA224098)

Procedure	C1D1[a] (Cycle Duration = 4 weeks)	C2D1 and D1 of Each Subsequent Cycle (± 3 days)	Notes:[b]
Biomarker Sample Collection	See Table 9.8-1 for further details.		Upon first recurrence (excluding MMIS), sample is to be collected before subsequent systemic therapy and/or before subsequent radiation therapy. Can be collected up to 90 days after recurrence as long as subsequent therapy has not be started as noted above.<br>Upon occurrence of ≥ Grade 3 drug-related AE (optional). Samples should be collected before dosing is resumed.
**Pharmacokinetic Samples and Immunogenicity Assessments**			
Collect Blood Samples for PK/IMG	See Table 9.5-1 for further details.		PK/IMG collections should be stopped for participants who start on subsequent systemic therapy.

Abbreviations: AE, adverse event; C, cycle; CLND, complete lymph node dissection; CPK, creatine phosphokinase; eCRF, electronic case report form; CT, computed tomography; D, day; eCOA, electronic clinical outcome assessments; ECOG, Eastern Cooperative Oncology Group; EORTC-QLQ-C30, European Organization for the Research and Treatment of Cancer Quality of Life Questionnaire; FACIT-GP5, Functional Assessment of Chronic Illness Therapy-Item GP5; FFPE, formalin-fixed paraffin-embedded; HCG, human chorionic gonadotropin; IMAE, immune-mediated adverse event; IRT, interactive response technology; MMIS, malignant melanoma in situ; MRI, magnetic resonance imaging; PK/IMG, pharmacokinetic/immunogenicity; SAE, serious adverse event; SARS-CoV-2, severe acute respiratory syndrome coronavirus 2; WOCBP, women of child-bearing potential.

[a] If a dose is delayed, the procedures scheduled for that same time point should also be delayed to coincide with when that time point's dosing actually occurs, with the exception of tumor assessments which should occur as scheduled.

[b] Some of the assessments referred to in this section may not be captured as data in the eCRF. They are intended to be used as safety monitoring by the treating physician. Additional testing or assessments may be performed as clinically necessary or where required by institutional or local regulations.

[c] Screening assessments performed within 3 days of first dose are not required to be repeated at C1D1.

Approved v4.0 930169531 4.0

Table 2-3: Follow-up Assessments (CA224098)

Procedure	Follow-Up Visits 1 & 2[a]	Long-term Follow-up[b]	Notes:[c]
**Safety Assessment**			
Targeted Physical Examination, Vital Signs, and Performance Status	X		Include weight, blood pressure, heart rate, temperature, performance status.
AE and SAE Assessment	X* (see notes)	X* (see notes)	Record at each visit. All SAEs and non-serious AEs should be collected continuously during the treatment period and for a minimum of 135 days following discontinuation of study treatment.<br>*Beyond 135 days from the last dose of study therapy, participants will be followed for drug-related AEs/SAEs until resolution, return to baseline, or event(s) is deemed irreversible, or until the participant is lost to follow-up, or withdraws study consent.<br>For all confirmed or suspected SARS-CoV-2 infections, participants will be followed for all SAEs, non-serious AEs of special interest (as defined in Section 9.2.3), and all AEs (SAEs and non-serious AEs) until resolution, the condition stabilizes, the event is otherwise explained, the event is deemed irreversible, the participant is lost to follow-up (as defined in Section 8.3), or for suspected cases, until SARS-CoV-2 infection is ruled-out.<br>Please refer to Section 9.2, and Appendix 3 for further details regarding the collection and follow-up of AEs.
Concomitant Medications	X (see notes)	X (see notes)	All medications with a start date within 135 days of the last dose date of study drug are considered concomitant.
**Laboratory Tests**			
Pregnancy Test (WOCBP)	X		Serum or urine (minimum sensitivity equivalent units 25 IU/L or equivalent units of HCG). For Norway, see Appendix 9 for specific requirements.<br>Note: Pregnancy testing is only required at FU Visits 1 and 2 unless increased frequency and duration is required per local regulations.

Table 2-3: Follow-up Assessments (CA224098)

Procedure	Follow-Up Visits 1 & 2[a]	Long-term Follow-up[b]	Notes:[c]
Clinical Laboratory Assessments	X (see notes)		To be performed at Follow-up Visit 1, repeat at Follow-up Visit 2 if study treatment-related toxicity persists.<br>Refer to Section 9.4.4, Clinical Safety Laboratory Assessments for the list of laboratory tests.<br>Laboratory toxicities (eg, suspected drug induced liver enzyme elevations) will be monitored during the follow-up phase, based on results from on-site/local labs, until all study drug related toxicities resolve, return to baseline, or are deemed irreversible.
**Efficacy Surveillance**			
Survival Status[b]	X	X (See Notes)	During Long-term Follow-up, participant survival status is assessed every 12 weeks (± 14 days) by either a clinic visit or telephone contact, and must include documentation of subsequent therapy.<br>Participants will be followed until death, loss to follow-up, withdrawal of consent, conclusion of the study, or a minimum of 8 years. Additional follow-up may continue for up to 5 years from the time of the final OS analysis.
Body Imaging	See Notes		Contrast-enhanced CT of the chest, abdomen, pelvis, and suspected sites of disease should occur every 12 weeks (± 7 days) for the first 2 years from randomization and every 26 weeks (± 14 days) beyond the Week 108 imaging time point until investigator assessed local, regional, or distant recurrence (whichever comes first) for Stage IV participants and until distant recurrence for Stage III participants. For mucosal melanoma participants, assessment should continue until investigator assessed local, regional, or distant recurrence for Stage M1, and until distant recurrence for M0 participants. See Section 9.1.2 for further details and exceptions.<br>In cases of suspected lesions of the extremities, contrast-enhanced MRI may be substituted for contrast-enhanced CT.<br>For head and neck mucosal melanomas, contrast-enhanced MRI of head and neck is required at every time point for nodal surveillance.<br>Please refer to Appendix 9 for Germany-specific imaging language.

Approved v4.0 930169531 4.0

Table 2-3: Follow-up Assessments (CA224098)

Procedure	Follow-Up Visits 1 & 2[a]	Long-term Follow-up[b]	Notes:[c]
Brain Imaging	See Notes		Participants with a history of brain metastasis or symptoms should have surveillance MRIs (without and with contrast) per standard of care (approximately every 12 weeks ± 7 days from randomization for the first 2 years and every 26 weeks (± 14 days) beyond the Week 108 imaging time point thereafter until disease recurrence) for Stage IV participants or sooner if clinically indicated. See Section 9.1.2 for further details.
Ultrasound of Region(s) Relevant to the Participant's Tumor Resection	See Notes		For participants without CLND, active surveillance using ultrasound of region(s) relevant to the participant's tumor resection must occur every 12 weeks (± 7 days) from randomization for the first 2 years and every 26 weeks (± 14 days) beyond the Week 108 imaging time point thereafter until disease recurrence.<br>Ultrasound is not required for head and neck mucosal participants.<br>Ultrasound is optional for participants who have undergone CLND relevant to the tumor resection.<br>Ultrasounds should not be submitted to the imaging vendor.
Subsequent Systemic Therapy and Outcome/Response	X	X	Following first recurrence, participants will continue to be followed during the Long-term Follow-up visits. Timing of objectively documented progression after the next line of therapy per investigator assessment will be documented. The next-line therapy(ies) and outcome/response will also be collected.
**Health Outcomes Assessments**			
EORTC QLQ-C30	X		Follow-up Visit 1 and 2 assessments to be administered at the site using eCOA. Each assessment should be completed at the start of the clinic visit prior to other study assessments.

Approved v4.0 930169531 4.0

Table 2-3: Follow-up Assessments (CA224098)

Procedure	Follow-Up Visits 1 & 2[a]	Long-term Follow-up[b]	Notes:[c]
FACIT-GP5	X		Adolescent participants (≥ 12 and < 18 years of age) only complete the EQ-5D-5L. Adolescent participants will continue to complete only the EQ-5D-5L even if they become ≥ 18 years of age during treatment or follow-up.<br>Long-term Follow-up EQ-5D-5L assessments will occur every 12 weeks (± 14 days) (in-person visit or telephone contact) for up to 5 years after first dose of study treatment. BMS may request extension beyond 5 years.<br>If Health Outcomes Assessments are collected but the dose is subsequently delayed, a data change form should be submitted to move the original data entry to an unscheduled visit. See Section 9.1.5 for further details.
EQ-5D-5L	X	X	
Healthcare Resource Utilization	X		Healthcare resource utilization data will be collected at each visit by study site staff using the electronic case report form (eCRF). See details of data to be collected in Section 9.11.
**Biomarker Assessment**			
Biomarker Sample Collection	See Table 9.8-1	X	Biomarker sample (ctDNA) collection is required at Follow-Up Visit 2 and then every 26 weeks (ie, at every alternating Survival Visit ± 90 days), but not to exceed 272 days between collections, thereafter to end of study or first recurrence, whichever occurs first. Biomarker sample collection will require participant visit the investigator site for blood draw.<br>Note: Biomarker samples (in person visits) are required every 26 weeks (± 90 days) following Follow-up Visit 2 until end of study or first recurrence, whichever occurs first.<br>Biomarker collections should be stopped for participants who start on subsequent systemic therapy.
Tumor Tissue Sample Collection (if Medically Feasible)	See Table 9.8-1		If biopsy or surgical resection is performed at recurrence, a tumor sample (a FFPE block or 20 positively charged unstained slides) must be submitted to central laboratory within 30 days of collection.

Approved v4.0 930169531 4.0

Table 2-3: Follow-up Assessments (CA224098)

Procedure	Follow-Up Visits 1 & 2[a]	Long-term Follow-up[b]	Notes:[c]
			Fine needle aspirates or other cytology samples are not acceptable. Biopsies of bone lesions that do not have a soft tissue component are not acceptable.
**Pharmacokinetic Samples and Immunogenicity Assessments**			
Collect Blood Samples for PK/IMG	See Table 9.5-1		PK/IMG collections should be stopped for participants who start on subsequent systemic therapy.

Abbreviations: AE(s), adverse event(s); BMS, Bristol-Myers Squibb; CLND, complete lymph node dissection; CT, computed tomography; ctDNA, circulating tumor deoxyribose nucleic acid; eCRF, case report form; eCOA, electronic clinical outcome assessment; EORTC QLQ-C30, European Organization for the Research and Treatment of Cancer Quality of Life Questionnaire; FACIT-GP5, Functional Assessment of Chronic Illness Therapy-Item GP5; FFPE, formalin-fixed paraffin-embedded; FU, follow-up; HCG, human chorionic gonadotropin; MRI, magnetic resonance imaging; PK/IMG, pharmacokinetic/immunogenicity; SAE, serious adverse event; SARS-CoV-2, severe acute respiratory syndrome coronavirus 2; WOCBP, women of child-bearing potential.

[a] Participants must be followed for at least 135 days after last dose of study treatment. Follow-Up Visit 1 should occur 30 days from the last dose (± 7 days) or can be performed on the date of discontinuation if that date is greater than 30 days after last dose. Follow-up Visit 2 occurs approximately 135 days (± 7 days) from last dose of study medication if date of discontinuation is < 135 days after last dose. If date of discontinuation is ≥ 135 days after the last dose and the timing of Follow-up 2 overlaps with Follow-up 1, then only one follow-up visit (Follow-up 1) needs to be completed. Follow-up visits must be conducted in person.

[b] Long-Term Follow-up visits to occur every 12 weeks (± 14 days) from the last Follow-up visit completed. If Follow-up visit is not completed, then Survival visit should be based off the off-treatment date. Visit may be conducted in clinic or by telephone. BMS may request that survival data be collected on all treated participants outside of the 12-week specified window. At the time of this request, each participant will be contacted to determine their survival status unless the participant has withdrawn consent for all contact.

[c] Some of the assessments referred to in this section may not be captured as data in the eCRF. They are intended to be used as safety monitoring by the treating physician. Additional testing or assessments may be performed as clinically necessary or where required by institutional or local regulation.

3 INTRODUCTION

Study CA224098 is a Phase 3, randomized, double-blind study of nivolumab + relatlimab (nivo + rela) fixed dose combination (FDC) compared with nivolumab monotherapy in completely resected Stage III-IV melanoma. This study will generate efficacy and safety data for the patient population with Stage IIIA (> 1 mm tumor in lymph node)/B/C/D or Stage IV (no evidence of disease [NED]) melanoma following complete resection of their lesion(s). The study will allow for direct comparison of the clinical benefits, as measured by the primary endpoint of recurrence free survival (RFS), provided by nivo + rela FDC compared with nivolumab monotherapy administered over a treatment duration of 12 months.

Melanoma may modulate and evade the host immune response through a number of mechanisms, including down regulation of tumor-specific antigen expression and presentation, secretion of anti-inflammatory cytokines, and upregulation of inhibitory ligands. Immune checkpoint blockade has recently demonstrated clinical efficacy in several cancer types, including melanoma, hepatocellular carcinoma (HCC), gastric cancer, non-small cell lung cancer (NSCLC), and hematologic malignancies.[1,2,3]

Nivolumab is a fully human, immunoglobulin G4 (IgG4 [kappa]) isotype monoclonal antibody that binds the programmed death-ligand 1 (PD-1) receptor on activated immune cells and disrupts engagement of the receptor with its ligands PD-L1 (B7-H1/CD274) and programmed death-ligand 2 (PD-L2) (B7-DC/CD273), thereby abrogating inhibitory signals and augmenting the host anti-tumor response. In early clinical trials, nivolumab has demonstrated activity in several tumor types, including melanoma, renal cell cancer (RCC), and NSCLC.[4] Furthermore, adjuvant nivolumab after complete resection of Stage III-IV melanoma has become a standard of care.

Relatlimab is a fully human lymphocyte activation gene 3 (LAG-3) specific antibody that was isolated following immunization of transgenic mice expressing human immunoglobulin (Ig) genes. Relatlimab binds to LAG-3 receptors expressed on T-cells with high affinity and prevents binding of this receptor to cells bearing its ligands, such as major histocompatibility complex (MHC) Class II[5] which is the peptide antigen presentation molecule recognized by cluster of differentiation 4 (CD4)+ T cells and fibrinogen-like protein 1 (FGL-1)[6], which is upregulated in a variety of human solid tumors. Relatlimab binding inhibits the negative regulatory function of LAG-3 mediated through its interaction with ligands in vitro; and elevated plasma levels of FGL-1 are correlated with poor prognosis.[6] FGL-1 was shown to be capable of delivering an inhibitory signal into T cells upon binding to the LAG-3 receptor and has been implicated as having a suppressive effect on anti-tumor immunity that is dependent on LAG-3.[7] Preclinical evidence suggests that the FGL-1-LAG-3 pathway may be a previously unknown immune evasion mechanism limiting responses to current cancer immunotherapies, including PD-1 pathway blockade.[6] By blocking the inhibitory LAG-3 signaling pathway, relatlimab enhances the anti-tumor immune response and, thus, has the potential to inhibit the growth of multiple malignancies when administered in combination with other therapeutic immuno-oncology (IO) agents. Dual

Approved v4.0 930169531 4.0

checkpoint inhibition with nivolumab and relatlimab results in enhanced T-cell effector function that is greater than the effects of either antibody alone in murine syngeneic tumor models.[8]

3.1 Study Rationale

Surgical resection followed by 1 year of adjuvant therapy is currently considered standard of care for Stage III-IV resectable melanoma. However, there remains an unmet need to improve RFS while maintaining favorable safety profile for these patients. The clinical benefit observed with nivo + rela FDC in patients with untreated metastatic or unresectable melanoma in the CA224047 Phase 2/3 study supports the rationale of evaluating nivo + rela FDC in patients with resectable melanoma.

Targeting LAG-3, which is involved in the immune checkpoint pathway, is a novel approach that may further overcome immune evasion mechanisms. LAG-3 (CD223) is a checkpoint receptor and expressed on several immune cell types including activated CD4+ and CD8+ T cells, memory T cells, regulatory lymphocytes, and natural killer cells.[5] Activation of the LAG-3 pathway occurs when LAG-3 interacts with its ligands, such as MHC Class II or other emerging ligands (eg, FGL-1), which triggers inhibitory activity that reduces the function of effector T cells.[5,6]

LAG-3 is often expressed on chronically exhausted T-cells and is frequently co-expressed with PD-1 on tolerized tumor infiltrating lymphocytes (TILs) across many tumor types.[9,10,11] Increased expression of LAG-3 on TILs, especially in the context of PD-1 expression, further promotes T cell exhaustion, leading to an impaired ability to attack tumor cells and an increased potential for tumor growth.[5,12] Preclinical studies indicate that inhibition of the LAG-3 pathway may restore effector function of T cells, promoting proinflammatory cytokine signaling, and ultimately, an anti-tumor response. The combination of LAG-3 and PD-1 inhibition demonstrated enhanced anti-tumor activity by targeting independent pathways with distinct functions.[5]

Relatlimab is a blocking antibody specific to the LAG-3 receptor. Relatlimab is being investigated in different indications and lines of therapies in combination with nivolumab. In the CA224020 Phase 1/2a study, the combination of nivolumab and relatlimab demonstrated tolerability, and preliminary clinical activity in advanced melanoma participants that had been previously treated with anti-PD-1/PD-L1 therapy.[8,13,14] In the CA224047 Phase 2/3 study, nivo + rela FDC demonstrated clinical benefit measured by progression-free survival (PFS) in patients with previously untreated metastatic or unresectable melanoma. PFS at 24 months was 38.5% for the nivo + rela FDC group versus 29% for the nivolumab monotherapy group.[15]

3.1.1 Research Hypothesis

Treatment with systemically administered nivo + rela FDC when compared with nivolumab will result in improved RFS in participants with completely resected Stage III-IV melanoma.

Approved v4.0 930169531 4.0

3.2 Background

Melanoma accounts for less than 5% of all skin cancers; however, it causes the greatest number of skin cancer–related deaths worldwide.[16] In 2020, it is estimated that there will be 100,350 new cases of melanoma and 6,850 deaths due to melanoma.[17]

Early melanoma detection followed by surgical excision is usually curative. In contrast, advanced melanoma frequently metastasizes to the lung, brain, or liver, and usually has poor prognosis.

The 5-year melanoma-specific survival (MSS) rate according to Stage III subgroups ranges from 93% in patients with Stage IIIA disease (1-3 clinically occult, tumor-involved sentinel lymph nodes [N1a or N2a] and T1a, T1b, or T2a primaries) to 32% for those with Stage IIID disease (patients with a thick and ulcerated primary [T4b] and either ≥ 4 tumor-involved regional nodes [N3a or N3b] or ≥ 2 tumor-involved nodes and evidence of microsatellite, satellite, or in-transit metastases [N3c]). Stage IIIB and Stage IIIC had 5-year MSS rates of 83% and 69%, respectively. For patients with > 1 mm of tumor burden in the lymph node, the 5-year MSS is 72% as compared to those with tumor burden in the lymph node of ≤ 1 mm with 5-year MSS of 91%.[18,19]

Cancer immunotherapies and B-RAF proto-oncogene (BRAF)/mitogen-activated protein kinase (MEK) inhibitors have played a role in the Stage III/IV adjuvant setting; however opportunities exist to continue enhancing clinical outcomes.

A potential mechanism of resistance to anti-PD-1 therapies is the presence of alternative checkpoint receptors that may be upregulated and further impede the function of effector T cells within the tumor microenvironment. Thus, targeting such additional checkpoint pathways may provide a therapeutic means to delay recurrence in melanoma patients who may rely on such checkpoint pathways to suppress the anti-tumor immune response. LAG-3 is one such checkpoint and is a potential cancer immunotherapeutic target due to its negative regulatory role on T cells.

The disease course for melanoma has fundamentally changed with the introduction of immunotherapies targeting the cytotoxic T lymphocyte associated antigen 4 (CTLA-4) and PD-1 checkpoints as well as BRAF and MEK inhibitors. Such agents have dramatically improved outcome for patients with metastatic melanoma; however, their role in the adjuvant setting continues to be evaluated. Studies have demonstrated that these agents enhance survival in the adjuvant setting albeit with waning durability in the long term. Therefore, novel immunotherapy combinations could address this long-term unmet need.

RFS rates at 1 year for adjuvant checkpoint inhibitor use range from 60.8% with ipilimumab (IPI) to 70.5% with nivolumab and 75.4% with pembrolizumab, yet there is a decline to 45% to 58% at 3 years, irrespective of which checkpoint inhibitor was used.[20,21,22] Similarly, adjuvant therapy with BRAF and MEK inhibitor combinations yield 3- and 4-year RFS rates of 58% and 54%, respectively compared to RFS rates of 88% at 1 year and 67% at 2 years with combination Dabrafenib/Trametinib treatment.[23,24]

Additional data from key studies are provided in Table 3.2-1.

Approved v4.0 930169531 4.0

Table 3.2-1: Data Comparison of Adjuvant Trials

Patient Population	CM238 Completely resected Stage IIIB/C or IV melanoma[20]		COMBI-AD Completely resected, BRAF v600 E/K-positive Stage IIIA/B/C Melanoma[24]		KN054 Completely resected Stage IIIA/B/C Melanoma[22]		CM915 Completely resected Stage IIIB/C/D or IV Melanoma[25]	
Treatment	Nivo	Ipi	Dab/ Tram	Placebo	Pembro	Placebo	Nivo/Ipi	Nivo
N	453	453	438	432	514	505	920	924
RFS HR	0.65 (97.56% CI 0.51-0.83), P < 0.0001		0.47 (95% CI 0.39-0.58), P < 0.001		0.57 (98.4% CI 0.43-0.74), P < 0.001		0.92 (97.295% CI, 0.77–1.09); P = 0.269	
1-year RFS rate (%)	71	61	88	56	75	61	N/A	N/A
18-months RFS rate (%)	66	53	N/A	N/A	71.4	53.2	N/A	N/A
2-years RFS rate (%)	N/A	N/A	N/A	N/A	N/A	N/A	64.6%	63.2%
3-years OS rate (%)	N/A	N/A	86	77	N/A	N/A	N/A	N/A
Grade 3-5 TRAEs (%)	14	46	31	5	15	3	33	13
DC rate due to AEs (%)	10	43	26	3	14	2	34	12

Abbreviations: AE, adverse event; BRAF, B-RAF proto-oncogene; CI, confidence interval; Dab, dabrafenib; DC, discontinuation; HR, hazard ratio; Ipi, ipilimumab; IV, intravenous; N/A, not applicable; Nivo, nivolumab; OS, overall survival; Pembro, pembrolizumab; RFS, recurrence-free survival; TRAE, treatment-related adverse events; Tram, trametinib.

The safety profiles of therapies in the adjuvant setting remains an important consideration. For instance, in the COMBI-AD trial, 26% of patients had adverse events (AEs) leading to treatment discontinuation, 38% had AEs leading to a dose reduction, and 66% had AEs leading to a dose interruption.[24] It is important to closely monitor for Grade 3 and 4 toxicities, such as colitis, rash, pneumonitis, endocrinopathies, and hepatitis with immune checkpoint inhibitors. Additional safety information is provided in Table 3.2-1.

Targeting LAG-3 should present a valuable mechanism to improve RFS.

3.2.1 Nivolumab Combined with Relatlimab Preclinical Activity

LAG-3 has been shown to be expressed in TILs of several tumor types, including melanoma, HCC, breast, ovarian, and lung cancers, often in connection with increased PD-1+ T cells.[26] Preclinical data presented in recent years illustrate a clear synergy between the inhibitory receptors LAG-3 and PD-1 in controlling immune homeostasis, preventing autoimmunity, and enforcing tumor-induced tolerance.[7,8] Importantly, combined treatment of mice with blocking antibodies against both receptors resulted in more robust immune responses than either single-treated group

Approved v4.0 930169531 4.0

in these studies, and analyses of *Lag-3*$^{-/-}$*Pdcd1*$^{-/-}$ double knockout mice revealed a cooperative requirement for LAG-3 and PD-1 in maintaining immune homeostasis.

The single agent anti-tumor activity of anti-LAG-3 antibody (19C7) was evaluated in the very immunogenic Sa1N fibrosarcoma tumor model.[27] Compared with the isotype control group, all doses between 1 mg/kg and 30 mg/kg of anti-LAG-3 clone 19C7 demonstrated anti-tumor efficacy leading to between 30% and 60% of the mice being rendered tumor-free at the end of study.

Combined anti-PD1 and anti-LAG-3 activity was also assessed in the Sa1N model. In 2 different studies, anti-LAG-3 antibody, C9B7W, inhibited the growth of Sa1N tumors in mice when administered as monotherapy and when combined with anti-PD-1 antibody, 4H2.[28,29] The combination of these 2 antibodies resulted in 80% to 90% tumor-free mice and reductions in median tumor volumes superior to that of anti-PD-1 or anti-LAG-3 antibody monotherapy alone.[8]

Similarly, greater anti-tumor effect was also demonstrated for combined anti-PD1 and anti-LAG-3 in the MC38 colon adenocarcinoma models than with either agent alone.[8]

3.2.2 Nivolumab Combined with Relatlimab Clinical Activity

Clinical efficacy of relatlimab as monotherapy and in combination with nivolumab has been studied in CA224020 and CA224022 at different doses and schedules. As monotherapy, relatlimab demonstrated activity in hematologic malignancies in the Phase 1/2a study CA224022. Objective responses were observed in participants with relapsed or refractory marginal zone lymphoma, Hodgkin lymphoma, and mantle cell lymphoma.[8]

The Phase 1/2a study CA224020 is investigating the safety, tolerability, and effectiveness of relatlimab, with and without nivolumab, to treat various solid tumors. Initial results show encouraging clinical activity when nivolumab is combined with relatlimab in the treatment of participants with solid tumors.[8,13,14] The combination dose of nivolumab 240 mg Q2W + relatlimab 80 mg Q2W induced responses in previously heavily treated advanced solid tumors and effected responses in tumors that had demonstrated resistance to nivolumab therapy. As of the cutoff date of 25-Feb-2021 for Part C of CA224020, objective responses in the range of 5.3% to 47% were achieved with nivolumab plus relatlimab combination therapy in participants with IO-refractory and first-line (1L) advanced melanoma, IO-naive bladder cancer, IO-refractory/relapsed and 1L NSCLC, IO-naive RCC, IO-naive gastric cancer, IO-naive HCC, and IO-naive squamous cell carcinoma of the head and neck (SCCHN). Refer to the Relatlimab Investigator’s Brochure (IB) for further information.[8]

Additionally, in Parts D1, D2, and E, objective responses were achieved in participants with IO-refractory melanoma treated with nivolumab + relatlimab. Part D1 included heavily pretreated participants with advanced melanoma (with a high proportion of participants treated with 2 or more prior lines of therapy including anti-CTLA-4 and/or anti-PD-1) and with a number of additional poor prognostic factors such as primary resistance to prior anti-PD-1 Stage IV disease with liver metastasis and high lactate dehydrogenase who experienced disease progression. Part D1 provided supportive evidence of the anti-tumor activity of nivolumab + relatlimab at doses

Approved v4.0 930169531 4.0

equivalent to nivolumab 480 mg and relatlimab 160 mg every 4 weeks (Q4W), with long-term clinical benefit as demonstrated by blinded independent central review (BICR)-confirmed overall response rate (ORR) of 11.8% (22/186 response evaluable) and a meaningful number of complete responses (8) and durability of these responses. In advanced melanoma participants with progression on prior IO therapies in Part E treated with nivolumab 480 mg + relatlimab 480 mg coadministered Q4W, the ORR by BICR was 11.7%. With a minimum follow-up of 11.3 months, the median duration of response was not reached (95% confidence interval [CI]: 3.06, non estimable). Refer to the relatlimab IB[8] Section 5.4.1 and nivolumab + relatlimab IB[30] Section 5.4.1 for further information.

CA224047, a global, double-blind, randomized, Phase 2/3 study comparing a fixed-dose combination (FDC) of nivolumab 480 mg + relatlimab 160 mg Q4W to nivolumab 480 mg Q4W in participants with previously untreated advanced melanoma, demonstrated a statistically significant and clinically meaningful benefit by dual inhibition of the LAG-3 and PD-1 pathways. The study achieved its primary endpoint, demonstrating statistically significant improvement in PFS by BICR with nivo + rela FDC (3:1) compared to nivolumab monotherapy in all randomized subjects (N = 714) (PFS hazard ratio = 0.75 [95% CI: 0.62, 0.92], P-value = 0.0055). With a median follow-up of 13.2 months, the median PFS in the nivo + rela FDC group was 10.1 months (95% CI, 6.4–15.7) compared to 4.6 months (95% CI, 3.4–5.6) in the nivolumab monotherapy arm. PFS rates at 12 months were 47.7% (95% CI, 41.8–53.2) and 36.0% (95% CI, 30.5–41.6) for nivo + rela FDC (3:1) and nivo monotherapy, respectively. The PFS benefit of nivo + rela FDC (3:1) was consistent across key prespecified subgroups.[30,31] The secondary endpoint of overall survival (OS) showed clinically meaningful improvement with nivolumab + relatlimab FDC over nivolumab monotherapy (median OS non estimable (34.20, not reached [NR]) versus 34.1 months (25.23, NR); HR = 0.80 [0.64-1.01]); p = 0.0593) in all randomized participants but was not statistically significant.[30,31]

Due to the statistical non significance of the secondary endpoint of OS, ORR was not formally tested per the statistical testing hierarchy; however, clinically meaningful numerical differences were observed with the nivolumab + relatlimab FDC arm over nivolumab monotherapy (43.1% vs 32.6%).[32]

3.2.3 Nivolumab Clinical Activity

Nivolumab has demonstrated durable responses exceeding 6 months as monotherapy in several tumor types, including NSCLC, melanoma, RCC, classical Hodgkin's lymphoma (cHL), SCLC, gastric cancer, SCCHN, urothelial cancer, HCC, and colorectal cancer (CRC). In confirmatory trials, nivolumab as monotherapy demonstrated a statistically significant improvement in OS as compared with the current standard of care in patients with advanced or metastatic NSCLC, unresectable or metastatic melanoma, advanced RCC, or recurrent or metastatic SCCHN. Details of the clinical activity in these various malignancies are provided in the USPI and SmPC.

Approved v4.0 930169531 4.0

3.2.4 Relatlimab Mechanism of Action

Relatlimab is a fully human antibody specific for human LAG-3 that was isolated from immunized transgenic mice expressing human Ig genes.[8] It is expressed as an IgG4 isotype antibody that includes a stabilizing hinge mutation (S228P) for attenuated Fc receptor binding in order to reduce or eliminate the possibility of antibody- or complement-mediated target cell killing. Relatlimab binds to a defined epitope on LAG-3 with high affinity (dissociation constant [Kd], 0.25-0.5 nM) and specificity and potently blocks the interaction of LAG-3 with its known ligands, MHC Class II (half maximal inhibitory concentration [IC50], 0.7 nM) and FGL-1. The antibody exhibits potent in vitro functional activity in reversing LAG-3-mediated inhibition of an antigen-specific murine T cell hybridoma overexpressing human LAG-3 (IC50, 1 nM). In addition, relatlimab enhances activation of human T cells in superantigen stimulation assays when added alone or in combination with nivolumab.[8] Refer to Relatlimab IB Section 4.1 and Section 4.2.[8]

3.2.5 Nivolumab Mechanism of Action

Nivolumab (also referred to as BMS-936558, MDX1106, or ONO-4538) is a human monoclonal antibody (HuMAb; immunoglobulin G4 [IgG4]-S228P) that targets the PD-1 CD279 cell surface membrane receptor. PD-1 is a negative regulatory molecule expressed by activated T and B lymphocytes. Binding of PD-1 to its ligands, PD-L1 and PD-L2, results in the down-regulation of lymphocyte activation. Inhibition of the interaction between PD-1 and its ligands promotes immune responses and antigen-specific T-cell responses to both foreign antigens as well as self-antigens. Nivolumab is expressed in Chinese hamster ovary (CHO) cells and is produced using standard mammalian cell cultivation and chromatographic purification technologies. The clinical study product is a sterile solution for parenteral administration.

Nivolumab (OPDIVO™) is approved for the treatment of several types of cancer in multiple regions including the United States (US, Dec-2014), the European Union (EU, Jun-2015), and Japan (Jul-2014).

3.2.6 Nivolumab Combined with Relatlimab Clinical Safety

The Phase 1/2a study CA224020 enrolled the highest number of participants treated with the combination therapy of nivolumab + relatlimab in dose-escalation (Part B) and expansion cohorts in Parts C, D, and E tested in every 2 week (Q2W) and Q4W dosing intervals. Refer to IB Table 5.5.1-1 for details of the dose levels tested across a range of solid tumor cohorts in Part B and dose-limiting toxicities (DLTs) observed.[8] The safety profile was acceptable at all dose levels tested from nivolumab 480 mg in combination with relatlimab up to 1440 mg given Q4W, with the MTD not reached at this dose level. Part C included expansion cohorts with multiple tumor types and Parts D and E included advanced melanoma participants (1L and progressed on prior-PD-1 therapy). Refer to relatlimab IB Table 5.5.1-2 for the details of number of participants tested with the dose combinations.[8]

As of a database cutoff date of 18-Jun-2021 in study CA224020, 1415 participants have been treated with nivolumab + relatlimab across Parts B, C, D, and E demonstrating that the combination was well tolerated across multiple tumor types.[8] Based on an analysis of AEs in the

Approved v4.0 930169531 4.0

all-combination group, 1374 (97.1%) participants experienced at least 1 event and 1164 (82.3%) participants experienced at least 1 event occurring in ≥ 5% of participants (refer to relatlimab IB[8] Table 5.5.1.2-1 for details). Across Parts B, C, D, and E, drug-related AEs were reported in 960 (67.8%) participants, with the most commonly reported (≥ 5% of participants) being fatigue (16.7%), pruritus (12.2%), diarrhea (8.6%), rash (8.8%), hypothyroidism (7.9%), arthralgia (7.4%), asthenia (7.3%), increased lipase and nausea (6.9% each), and dry mouth (5.4%). Most drug-related AEs were Grades 1-2. Grade 3-4 drug-related AEs were reported in 230 (16.3%) participants and the most frequently reported included lipase increased (3.7%), and 2 (0.1%) participants reported Grade 5 events (dyspnea and pulmonary fibrosis). Refer to the IBs for relatlimab[8] and relatlimab + nivolumab FDC[30] (Section 5.5.1) for details of drug-related AEs analyzed separately for Part D1, which included 82 heavily pretreated participants with advanced melanoma, with poor prognostic factors treated with nivolumab 480 mg + relatlimab 160 mg FDC.

Drug-related SAEs were reported in 179 of the 1415 (12.7%) participants with Grade 3-4 drug-related SAEs occurring in 130 (9.2%) participants and Grade 5 events occurring in 2 (0.1%) participants (dyspnea and pulmonary fibrosis). At least 1 drug-related AE leading to discontinuation has been reported in 119 of 1415 (8.4%) participants. More information can be found in relatlimab IB Section 5.5.1.[8]

Troponin is a highly sensitive non-specific indicator of myocardial injury. Troponin is not typically monitored during treatment with immune checkpoint inhibitors due to uncertain value in preventing immune-related cardiac morbidity. In CA224020, myocarditis was uncommon despite the context of regular protocol-mandated troponin monitoring and a requirement for cardiac assessment in participants with raised troponin values. While myocarditis is a recognized risk with immune checkpoint inhibitors, routine troponin monitoring during treatment has not been recommended by clinical guidelines due to lack of demonstrated value in preventing morbidity or mortality. The protocol contains a myocarditis treatment algorithm for prompt diagnostic evaluation and treatment of symptoms potentially related to myocarditis and excludes participants with a prior history of myocarditis.[33]

The safety profile of the combination of nivolumab 480 mg + relatlimab 160 mg FDC Q4W was confirmed in the pivotal Phase 2/3 global, multicenter study CA224047 (n = 355), in previously untreated advanced melanoma participants (clinical data cutoff 09-Mar-2021). The study findings demonstrated a well-tolerated regimen with a manageable safety profile and without unexpected safety signals. The incidence of Grade 3-4 drug-related AEs was 18.9% in the nivolumab + relatlimab FDC group versus 9.7% in the nivolumab monotherapy group. There were 3 treatment-related deaths with nivolumab + relatlimab FDC and 2 with nivolumab monotherapy. Drug-related AEs (any grade) led to treatment discontinuation in 14.6% and 6.7% of patients in the nivolumab + relatlimab FDC and nivolumab monotherapy groups, respectively.[31] Refer to relatlimab + nivolumab FDC IB Section 5.5.2 for an updated safety analysis based on the clinical data cutoff date of 30-Jun-2021.[30]

Approved v4.0 930169531 4.0

3.3 Benefit/Risk Assessment

As described in Section 3.2.2, clinical activity was seen with the combination of nivolumab + relatlimab in multiple tumor types, including treatment naive and previously heavily treated tumors including previous anti-PD-1 therapy, in the CA224020 study.[8] The clinical efficacy of the combination was further confirmed in study CA224047, a global, double-blind Phase 2/3 randomized controlled trial that demonstrated a statistically significant and clinically meaningful PFS benefit of nivolumab + relatlimab FDC Q4W compared to nivolumab 480 mg Q4W,[31] as well as clinically meaningful improvement in OS and ORR, although the OS result was not statistically significant and ORR could not be formally tested.[32]

The combination of nivolumab and relatlimab has shown well-defined toxicity profiles based on a safety database comprised of participants treated with either relatlimab monotherapy or as a combination across multiple tumor types. Five ongoing studies have incorporated dosing of nivolumab + relatlimab sequentially or co-administered: CA224020, CA224022, CA224034, CA224087 (clinical data cutoff date of 18-Jun-2021), and CA224048 (clinical cutoff date 07-Jul-2021).[8] The safety profile of nivolumab combined with relatlimab is manageable with currently no MTD reached, as combination dose-escalation is ongoing.

Drug-related AEs were reported in 1225 of 1723 participants across studies CA224020, CA224022, CA224034, CA224048, and CA224087. The most frequent drug-related AEs included fatigue, decreased appetite, pruritus, diarrhea, rash, rash maculo-papular, anemia, arthralgia, increased lipase, nausea, dry mouth, asthenia, hypothyroidism, hyperthyroidism, cough, pyrexia, increased alanine aminotransferase, and increased aspartate aminotransferase.[8] Immune-mediated AEs (IMAEs) seen with nivolumab in combination with relatlimab appear consistent with those seen with other immunotherapy agents, and can potentially include pneumonitis, colitis, hepatitis, nephritis, endocrinopathy, neurologic, and skin AEs. In combination with nivolumab, the frequency, severity, and reversibility of IMAEs appear consistent with the mechanisms of action of either drug. The majority of all drug-related AEs and IMAEs were reversible or manageable by withholding study intervention administration, providing standard medical care, and/or following IMAE management algorithms (see Appendix 5). Most immune-mediated study intervention-related endocrinopathies will require life-long management by hormone replacement therapies. The overall safety profile of nivolumab + relatlimab across these studies remains unchanged and manageable with established management guidelines (including myocarditis cases). Refer to relatlimab IB Section 7.1 for further details.[8]

Furthermore, nivolumab 480 mg + relatlimab 160 mg FDC (3:1) administered Q4W was confirmed to be well tolerated with a manageable safety profile in 355 1L advanced melanoma participants treated in the CA224047 study. There were no unexpected safety signals from this combination.[31] Please refer to the nivolumab + relatlimab FDC IB Section 5.5.4[30] for details of other unexpected life-threatening and/or fatal serious adverse reactions.

Safety results to date indicate that sequential, co-administration, and the FDC of nivolumab + relatlimab administration have similar manageable safety profiles that are consistent with the

Approved v4.0 930169531 4.0

mechanisms of action of each agent, nivolumab and relatlimab. No new types of clinically important events have been identified.[8,30]

Given the safety profile of relatlimab as monotherapy and combination therapy observed to-date,[8] and the positive benefit-risk of the pivotal Phase 2/3 study of nivo + rela FDC (3:1) demonstrated in participants with previously untreated metastatic or unresectable advanced melanoma,[31] and the significant unmet medical need in adjuvant treatment for Stage III-IV resectable melanoma population, the continuing evaluation of nivo + rela FDC is justified for the patient population in this protocol.

Investigators in the CA224098 study were notified on 12-Sep-2022 regarding 6 reported cases of concurrent immune-related myositis, myasthenia gravis, or myocarditis. Five of the 6 identified participants (out of ~600 treated) presented with high-grade (Grade 3 or Grade 4) events. In 4 cases, the participants presented with features of marked muscle weakness (including respiratory weakness and ptosis/diplopia in 3 cases) associated with substantial elevations of creatinine phosphokinase, troponins, and transaminases. Two of these participants rapidly deteriorated despite immunosuppression and supportive management and died during hospital admission. All cases occurred within the first 2 cycles of the assigned treatment. BMS requested an ad hoc independent Data Monitoring Committee (DMC) meeting to permit a thorough review of all reported cases within the study. These events did not constitute an Urgent Safety Concern, and no change to the conduct of this study was recommended by the DMC. There is ongoing close monitoring of the AEs in the study.

Investigators and the Sponsor will utilize continuous safety assessments to determine whether additional safety measures or termination from the study is required at any time. In addition, AEs and SAEs will be reviewed on an ongoing basis by the Sponsor's Medical Monitor/designee and Worldwide Patient Safety (WS) representatives to monitor for any safety signals or trends. As relatlimab is an experimental agent, it is possible that unforeseen, unknown, or unanticipated reactions may occur; however, data shows that nivo + rela is safe and tolerable at 480 mg nivolumab + 160 mg relatlimab Q4W using FDC product in other studies.

Approved v4.0 930169531 4.0

3.3.1 Risk Assessment

Table 3.3.1-1: Risk Assessment

Potential Risk of Clinical Significance	Summary of Data/Rationale for Risk	Mitigation Strategy
**Study Interventions**		
Immune-mediated AEs (eg, colitis diarrhea/colitis, pneumonitis, hepatitis, nephritis, endocrinopathy, rash, neurologic, AEs)	FDC (BMS-986213) IB	Recommended IMAE management algorithms are included in Appendix 5 or as per institutional protocol/investigator discretion
Potential Infusion Related Reaction	FDC (BMS-986213) IB	Recommended management algorithms are included in Appendix 5 or as per institutional protocol/ investigator discretion
Dermatologic AEs	Nivolumab IB	Recommended management algorithms are included Appendix 5 or as per institutional protocol/ investigator discretion
Cardiovascular AEs (ie, myocarditis, troponin elevation)	FDC (BMS-986213) IB	Management of Myocarditis per AE Management Algorithm in Appendix 5 or as per institutional protocol/ investigator discretion.
Hemorrhage AE (eg, epistaxis, gingival bleeding, GI blood loss)	Relatlimab IB	Monitor clinical signs/symptoms/ CBC and coagulation parameters. Management as per institutional protocol/investigator
Opportunistic Infections due to Immunosuppression	FDC (BMS-986213) IB	Monitor signs/symptoms, may require additional laboratory tests. Management as per institutional protocol/investigator
Potential developmental toxicity	FDC (BMS-986213) IB	Exclusion criteria, pregnancy testing, contraception per protocol, as per Section 6.1, 4) and Appendix 4.
**Study Procedures**		
Tumor Biopsy (eg, pain, infection)	Not applicable	Per institutional protocol/ investigator discretion.
Phlebotomy (eg, pain, ecchymosis, bleeding, syncope)	Not applicable	Per institutional protocol/ investigator discretion.
MRI	Not applicable	Management per institutional protocol/investigator discretion.
CT	Not applicable	Management per institutional protocol/investigator discretion.
Ultrasound	Not applicable	Management per institutional protocol/investigator discretion.

Approved v4.0 930169531 4.0

Table 3.3.1-1: Risk Assessment

Potential Risk of Clinical Significance	Summary of Data/Rationale for Risk	Mitigation Strategy
**Other (if applicable)**		
Allergy to contrast agent (eg, reaction to anaphylaxis)	Not applicable	Prophylaxis and/or treatment per institutional protocol/ investigator discretion

Abbreviations: AE, adverse event; CBC, complete blood count; CT, computed tomography; FDC, fixed dose combination; GI, gastrointestinal; IB, investigator brochure; IMAE, immune-mediated adverse events; MRI, magnetic resonance imaging.

3.3.2 Benefit Assessment

This study investigates if the adjuvant use of systemic therapy of nivo + rela FDC will improve recurrence free survival (RFS) for Stage III-IV completely resected melanoma participants with no evidence of disease. Currently, nivolumab monotherapy is an approved treatment for adjuvant melanoma patients in many countries. While nivo + rela FDC provided PFS benefit for the treatment of untreated metastatic or unresectable melanoma, it is not yet approved for any indication.

Despite the availability of effective adjuvant therapies, there is an unmet need to provide safe and effective adjuvant systemic therapy that offers additional clinical benefit in RFS in this patient population. It is unknown if nivo + rela FDC will improve RFS compared to nivolumab monotherapy. Although benefits are hypothesized, actual clinical benefits to patients with Stage III or Stage IV resectable melanoma have not been established yet. Participation in this study may help offer new therapies for advanced resectable melanoma patients and establish the clinical benefits of the combination therapy.

Participants enrolled in this study will be randomized to either the nivo + rela FDC or nivolumab monotherapy. Participants will have an equal chance of being randomized to the nivo + rela FDC therapy or the standard of care nivolumab monotherapy.

3.3.3 Overall Benefit/Risk Conclusion

Taking into account the measures taken to minimize risk to participants in this study, the potential risks identified in association with nivo + rela FDC are justified by the anticipated benefits that may be afforded to participants with resectable melanoma.

The Sponsor will evaluate the risk/benefit profile of the study on an ongoing basis. This evaluation will be based on all available data – with particular attention to: (i) AEs or other safety trends in this or any other clinical study of nivo + rela FDC whose character, severity, and/or frequency suggest that participants would be exposed to an unreasonable and significant risk of illness or injury; (ii) new nonclinical data suggesting unreasonable and significant risk of illness or injury.

If such evaluation suggests that the risk/benefit profile of the study has become unfavorable to participants, the Sponsor will pause enrollment and/or treatment until further evaluation of data,

Approved v4.0 930169531 4.0

and interaction with the appropriate Health Authority(ies) can take place on potential actions. Such actions may include (but are not limited to) study continuation, substantial amendment, or termination of the study.

4 OBJECTIVES AND ENDPOINTS

Table 4-1: Objectives and Endpoints

Objectives	Endpoints
**Primary**	
• To compare the efficacy, as measured by RFS, provided by nivo + rela FDC vs nivolumab monotherapy in participants with completely resected Stage III/IV NED melanoma.	• RFS time as assessed by the investigator. RFS is defined as the time between the date of randomization and the first date of documented recurrence (local, regional, distant, new primary melanoma) or death due to any cause, whichever occurs first.
**Key Secondary**	
• To compare the OS provided by nivo + rela FDC vs nivolumab monotherapy in participants with completely resected Stage III/IV NED melanoma.	• OS is defined as the time between the date of randomization and the date of death due to any cause.
**Other Secondary**	
• To assess the efficacy, as measured by DMFS, provided by nivo + rela FDC vs nivolumab monotherapy in participants with completely resected Stage III/IVA/IVB NED melanoma.	• DMFS, by investigator, is defined as the time between the date of randomization and the date of first distant metastasis or date of death due to any cause, whichever occurs first.
• To assess safety and toxicity of nivo + rela FDC vs nivolumab monotherapy in participants with completely resected Stage III/IV NED melanoma.	• Incidence and severity of AE, SAEs, AEs leading to DC, IMAEs, drug-related AEs, deaths, laboratory abnormalities, and other select AEs.
• To evaluate investigator-assessed outcomes on next-line systemic therapies.	• PFS2 defined as time from randomization to second recurrence/objective disease progression on next-line systemic therapy per investigator, or death from any cause, whichever occurs first.
**Exploratory**	
• To characterize the pharmacokinetics of nivo + rela FDC and nivolumab monotherapy.	• End of infusion concentration and trough concentrations.
• To characterize the immunogenicity of nivo + rela FDC and nivolumab monotherapy.	• Immunogenicity assessed by anti-relatlimab and anti-nivolumab antibodies.
• To evaluate additional investigator-assessed outcomes on next systemic therapies.	• Duration of treatment on next-line therapies.<br>• Time to next treatment.
• To evaluate freedom from recurrence defined as the time from randomization to recurrence, with censoring of data for participants who had died from causes other than melanoma or treatment-related toxic effects.	• Freedom from recurrence.
• To evaluate treatment free interval defined as the time from last dose of study treatment to the start of subsequent systemic therapy or death.	• Treatment free interval.

Approved v4.0 930169531 4.0

Table 4-1: Objectives and Endpoints

Objectives	Endpoints
• To explore potential association of biomarkers (eg, LAG-3 expression, PD-L1 expression, BRAF mutation status, TMB, genomic signatures soluble biomarkers, etc.) with clinical efficacy (RFS, DMFS, and OS) and/or incidence of adverse events of nivolumab and/or relatlimab by analyzing biomarker measures within the tumor microenvironment and periphery (eg, blood, serum, plasma, tumor tissue and PBMCs) in comparison to clinical outcomes.[a]	• Association of biomarkers to RFS, DMFS, OS, incidence of AEs and LAG-3, PD-L1, BRAF mutation status, TMB, genomic signatures, soluble biomarkers, etc.[a]
• To explore the role of ctDNA to understand MRD and disease recurrence predictability.[a]	• Association of recurrence with ctDNA positivity and dynamic changes in levels.[a]
• To evaluate changes in the tumor microenvironment and peripheral indicative of immunomodulatory effect.	• Changes of PD-L1 and LAG-3, gene expression, and other biomarkers in the TME and peripheral.[a]
• To assess the impact of SARS-CoV-2 serologic status on participants receiving nivo + rela FDC and nivolumab monotherapy in melanoma participants and to support heath authority requests.	• Exploratory measurements of SARS-CoV-2 serology (anti-SARS-CoV-2 total or IgG), from serum samples collected at baseline and the potential association between these measurements and selected endpoints related to safety, efficacy, and/or biomarkers.
• To assess the participant's cancer-related QoL using the EORTC QLQ-C30.	• Mean changes from baseline in scores and proportion of participants achieving clinically meaningful changes in EORTC QLQ-C30 scores for functional scales, symptom scales, and global health status/QoL scale at all post-baseline assessments.
• To characterize participant perceptions of the bothersomeness of symptomatic AEs, based on FACIT-GP5 item.	• Summary changes and frequency of responses in FACIT-GP5 item measuring bother due to side effects of treatment.
• To evaluate the participant's overall health status using the EQ-5D-5L index and visual analog scale.	• Mean changes from baseline in VAS and utility index and proportion of participants achieving clinically meaningful changes in scores to all post-baseline assessments in EQ-5D-5L in both the visual analog scale and the utility index.
• To characterize health care resource utilization.	• Hospitalizations, non-protocol specified medical visits, diagnostics.

Abbreviations: AE(s), adverse event(s); BRAF, B-RAF proto-oncogene; ctDNA, circulating-tumor deoxyribose nucleic acid; DC, discontinuation; DMFS, distant metastasis-free survival; EORTC QLQ-C30, European Organization for the Research and Treatment of Cancer Quality of Life Questionnaire; FACIT-GP5, Functional Assessment of Chronic Illness Therapy-Item GP5; FDC, fixed dose combination; HRQoL, health-related quality of life; IgG, immunoglobulin G; LAG-3, lymphocyte activation gene 3; MRD, minimal residual disease; NED, no evidence of disease; nivo, nivolumab; PBMC, peripheral blood mononuclear cell; PD-L1, programmed death-ligand 1; PFS2, progression-free survival 2; QoL, quality of life; RFS, recurrence-free survival; OS, overall survival; rela, relatlimab; SAE, serious adverse event; SARS-CoV-2, severe acute respiratory syndrome coronavirus 2; TMB, tumor mutation burden; TME, tumor microenvironment; VAS, visual analog scale; vs, versus.

[a] Please refer to Appendix 9 for Denmark-specific differences.

Approved v4.0 930169531 4.0

5 STUDY DESIGN

5.1 Overall Design

This is a Phase 3, randomized, double-blind study of nivo + rela FDC compared with nivolumab monotherapy in participants (≥ 12 years of age) with completely resected Stage IIIA (> 1 mm tumor in lymph node)/B/C/D or Stage IV NED melanoma by American Joint Committee on Cancer version 8 (AJCC v8). Efficacy of nivo + rela FDC, as measured by RFS time (as assessed by the investigator), will be compared to nivolumab monotherapy.

Participants will be randomized 1:1 to receive treatment with one of the following:

- Nivo + rela FDC dosing:
  - ≥ 18 years of age **OR** ≥ 12 years to < 18 years of age and ≥ 40 kg: nivolumab 480 mg and relatlimab 160 mg IV every 4 weeks (Q4W)
  - ≥ 12 years to < 18 years of age and < 40 kg: nivolumab 6 mg/kg and relatlimab 2 mg/kg IV Q4W
- Nivolumab dosing:
  - ≥ 18 years of age **OR** ≥ 12 years to < 18 years of age and ≥ 40 kg: nivolumab 480 mg IV Q4W
  - ≥ 12 years to < 18 years of age and < 40 kg: nivolumab 6 mg/kg IV Q4W

Participants will be stratified by AJCC v8 stage (Appendix 7) and geographic region. All participants will be treated until: recurrence of disease (excluding melanoma in situ, refer to Section 5.5.4), unacceptable toxicity, participant withdrawal of consent, or a maximum of 1 year of treatment from first dose (maximum of 13 doses), whichever occurs first.

This study will consist of 3 phases: screening, treatment, and follow-up. For a complete list of study required procedures, please refer to Section 2.

The study design schematic is presented in Figure 5.1-1.

Approved v4.0 930169531 4.0

Figure 5.1-1: Study Design Schema

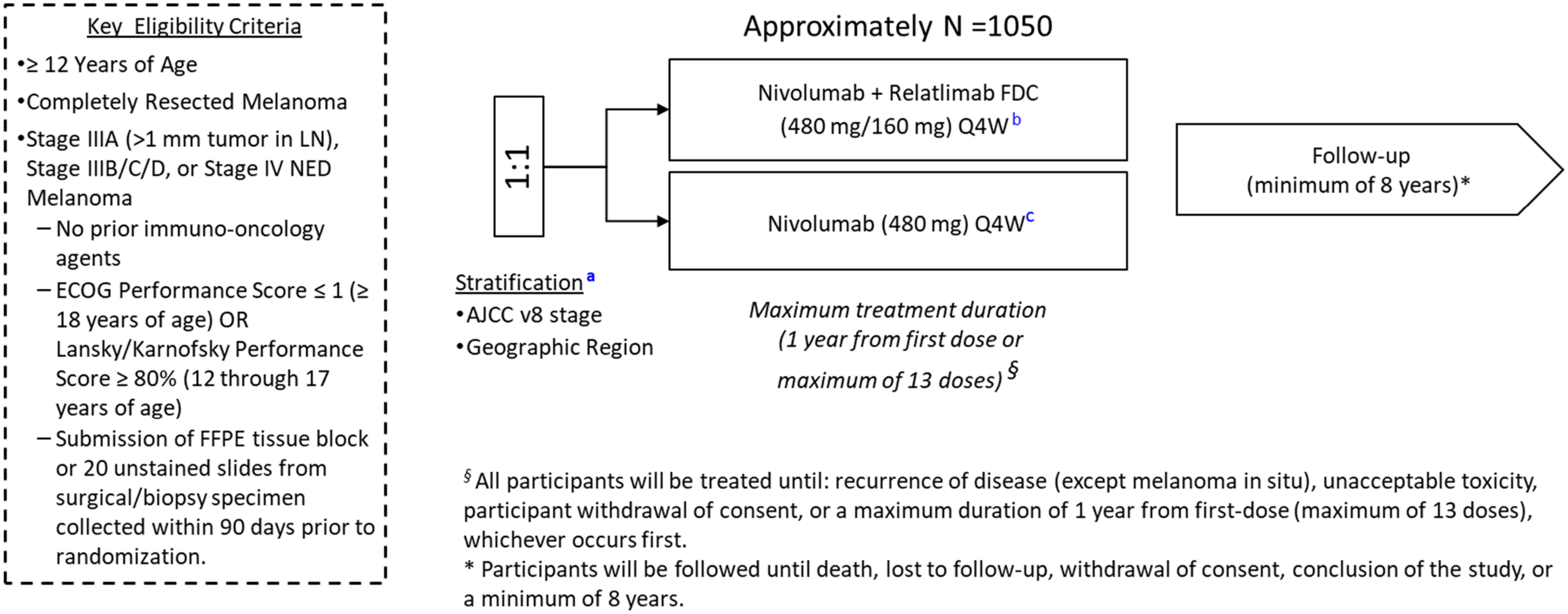

Abbreviations: AJCC v8, American Joint Committee on Cancer version 8; ECOG, Eastern Cooperative Oncology Group; FDC, fixed dose combination; FFPE, formalin-fixed paraffin-embedded; IV, intravenous; LN, lymph node; NED, no evidence of disease; ROW, rest of the world; Q4W, every 4 weeks

[a] Stratification:

- AJCC v8: Stages IIIA/IIIB, Stage IIIC, Stages IIID/IV (including all participants with mucosal melanoma, Stage III, Stage IVA, Stage IVB, and Stage IVC)
- Geographic Regions: USA/Canada/Australia, Europe, ROW

[b] Nivo + rela FDC dosing:

- ≥ 18 years of age **OR** ≥ 12 years to < 18 years of age and ≥ 40 kg: nivolumab 480 mg and relatlimab 160 mg IV Q4W.
- ≥ 12 years to < 18 years of age and < 40 kg: nivolumab 6 mg/kg and relatlimab 2 mg/kg IV Q4W.

[c] Nivolumab dosing:

- ≥ 18 years of age **OR** ≥ 12 years to < 18 years of age and ≥ 40 kg: nivolumab 480 mg IV Q4W.
- ≥ 12 years to < 18 years of age and < 40 kg: nivolumab 6 mg/kg IV Q4W.

Approved v4.0 930169531 4.0

5.1.1 Data Monitoring Committee and Other Committees

When required, adjudicated events will be submitted to the Data Monitoring Committee (DMC) and Health Authorities for review on a specified timeframe in accordance with the adjudication documentation.

A DMC will be established to provide oversight of safety and efficacy considerations and to provide advice to the Sponsor regarding actions the committee deems necessary for the continued protection of participants enrolled in the study. The DMC will be charged with assessing such actions in light of an acceptable benefit/risk profile for nivo + rela FDC. The DMC will act in an advisory capacity to BMS and will monitor participant safety and evaluate the available efficacy data for the study. The oncology therapeutic area of BMS has primary responsibility for design and conduct of the study.

The efficacy is not planned to be provided at regular DMC meetings. If the DMC requests efficacy summary data in order to perform benefit/risk assessment prior to the RFS IAs, then the efficacy will be available at the request of the DMC. The availability of efficacy will not be automatically carried over to the next DMC meeting unless the DMC requests efficacy again at the subsequent meeting. An administrative alpha penalty will occur and will be subtracted from the overall type I error of 0.05 for each additional unplanned look requested by the DMC.

Details of the DMC responsibilities and procedures will be outlined in the DMC charter.

A Study Steering Committee (SSC), consisting of Investigators and personnel members representing the Sponsor of the study, will be established to obtain scientific guidance and advice for the protocol and conduct of the study. Details of the SSC responsibilities and procedures will be specified in the SSC charter.

5.2 Number of Participants

Approximately 1,050 participants are expected to be randomized to the following treatment arms:

- Nivo + rela FDC (n = 525)
- Nivolumab monotherapy (n = 525)

See Section 10.2 for sample size determination.

The randomization will be stratified by the following stratification factors:

- Geographic region (USA/Canada/Australia vs Europe vs ROW)
- American Joint Committee on Cancer (AJCC v8) Stage IIIA/IIIB vs Stage IIIC vs Stage IIID/IV (including all participants with mucosal melanoma, Stage III, Stage IVA, Stage IVB, and Stage IVC - see Appendix 8)

5.3 End of Study Definition

The start of the trial is defined as the first participant screened. End of trial is defined as the last visit or scheduled procedure shown in the Schedule of Activities for the last participant.

Approved v4.0 930169531 4.0

Study completion is defined as the final date on which data for the primary endpoint was or is expected to be collected, if this is not the same.

A participant is considered to have completed the study if he/she has died, is lost to follow-up, has withdrawn consent, the study has concluded, the last visit completed, or has been followed for a minimum of 8 years. Additional follow-up may continue for up to 5 years from the time of the final OS analysis. The trial will end once this follow-up has concluded.

5.4 Scientific Rationale for Study Design

5.4.1 Participant Input Into Study Design

BMS may approach advocacy groups and other organizations to discuss key details of the study. Those suggestions may be implemented into the study design, at the determination of the sponsor.

5.4.2 Rationale for Use of Nivolumab + Relatlimab Fixed Dose Combination

The rationale for combining a LAG-3 inhibitor and an anti-PD-1/PD-L1 agent is based on the potential role of LAG-3 in T-cell exhaustion and anti-PD-1 resistance.[10] Relatlimab binds to LAG-3 receptors with high affinity and blocks its interaction with ligands such as major histocompatibility complex (MHC) Class II on antigen presenting cells. Nivolumab binds to PD-1 receptors with high affinity and blocks its interaction with its ligands, PD-L1 and PD-L2. LAG-3 and PD-1 are immune-checkpoint receptors that act synergistically on effector T cells, leading to the development of T-cell dysfunction and impaired cytotoxic function. Combined nivolumab and relatlimab mediated inhibition enables T-cell activation and restores effector function of T cells, that is greater than the effects of either antibody alone, leading to the initiation of an improved immune response and promoting tumor cell death.

The central hypothesis is that dual LAG-3 and PD-1 checkpoint blockade has the potential to offer superior efficacy compared with single agent anti-PD-1 therapy in the immunotherapy-naive treatment setting, as well as to provide meaningful efficacy to patients who experienced disease progression during anti-PD-1 therapy. The mechanism of action for both agents and preclinical data support the potential for producing clinical benefit. See Section 3.2 for more details on the mechanism of action and preclinical data of relatlimab and nivolumab. Preliminary data from a Phase 1/2a study of nivolumab (anti-PD-1) and relatlimab (anti-LAG-3) in combination showed promising clinical activity in participants with previously treated recurrent or metastatic melanoma, with a safety profile similar to nivolumab monotherapy. In the CA224047 Phase 2/3 study, nivo + rela FDC demonstrated clinical benefit measured by PFS in patients with previously untreated metastatic or unresectable melanoma. The FDC was well-tolerated and there were no new safety signals reported.

The FDC product contains nivolumab and relatlimab in protein-mass ratio 1:3 in a single vial. The FDC will be administered via intravenous (IV) infusion over approximately 30 minutes. Key advantages of a FDC are as follows:

- Patients benefit from reduced infusion time and less time in the clinic and/or doctor's office
- Increased ease of administration

Approved v4.0 930169531 4.0

- Pharmacists require less time in preparation of the intravenous solution to be administered to the patient
- Reduces potential error for medication preparation and administration

5.4.3 Rationale for Choice of Comparator

In the adjuvant Stage III/IV resected melanoma setting, nivolumab is an established standard of care that is recognized in guidelines globally with positive recommendation ratings. In CheckMate-238, nivolumab demonstrated clinical benefit with an acceptable safety profile in the patient populations (Stage IIIA with > 1 mm tumor in lymph node was not included) that are being evaluated in this study.

CheckMate-238 randomly assigned patients with resected Stage IIIB, IIIC, or IV disease to receive nivolumab vs ipilimumab. Patients' diseases were categorized by the AJCC v7, and all patients underwent resection and lymph node dissection. The 12-month RFS was 70.5% in the nivolumab group vs 60.8% in the ipilimumab group. At 24 months, RFS was 62.6% in the nivolumab group vs 50.2% in the ipilimumab arm. This benefit was found regardless of stage, PD-L1 status, or BRAF status. Treatment was better tolerated in the nivolumab group, with 14.4% of patients experiencing Grade 3 or 4 toxicity compared with 45.9% in the ipilimumab group. On the basis of the results of CheckMate 238, the use of ipilimumab is no longer recommended in the adjuvant setting given the superiority of nivolumab coupled with less toxicity.[20]

Based on the available data and given the fact that nivolumab is approved for adjuvant treatment of patients with melanoma with involvement of lymph nodes or metastatic disease who have undergone complete resection, nivolumab is deemed an appropriate clinical comparator.

5.4.4 Rationale for Choice of Primary Endpoint

Recurrence free survival (RFS) is a validated clinical endpoint and is defined as the time from randomization until first recurrence (loco-regional or distant metastasis) or death due to any cause, whichever was observed first. Treatment options that are clinically active are increasingly available to patients with resected Stage III/IV adjuvant melanoma. These options have demonstrated a RFS improvement and have subsequently been granted regulatory approvals.

Overall survival (OS) is often used as the primary endpoint in oncology clinical trials. However, OS, although clinically meaningful, has the disadvantages of requiring extended follow-up and of being confounded by subsequent lines of treatment. An endpoint that is reached more rapidly could potentially expedite decisions on efficacy.

Based on the available data, a RFS improvement compared with an established standard of care would demonstrate a potentially new combination in the resected Stage III/IV adjuvant melanoma setting.

5.4.4.1 Rationale for Choice of Stratification Factors

In order to minimize the potential for imbalances across treatment arms, there will be 2 stratification factors utilized in this trial: AJCC v8 - Melanoma Stage IIIA/IIIB vs Stage IIIC vs

Approved v4.0 930169531 4.0

Stage IIID/IV (including all participants with mucosal melanoma, Stage III, Stage IVA, Stage IVB, and Stage IVC) and geographic region (USA/Canada/Australia vs Europe vs ROW).

Prognostic implications of AJCC v8 staging are well established. There is a continued need to move beyond histopathologic and clinical criteria for risk stratification.

Geographic region (USA/Canada/Australia vs Europe vs ROW): Differences in patient populations and clinical practice patterns in the different regions are well known and can impact outcomes. Region has been included as a stratification factor to control for potential differences in patient populations and different approaches to clinical practice in the different regions. ROW will include Central America, Latin America, and China.

5.4.5 Rationale for Blinding

The study will be double-blinded in order to:

- Minimize bias including bias arising from differences in thresholds for classification of recurrence between the arms which could subsequently affect treatment duration between the arms and have an impact on the primary endpoint of RFS
- Curtail bias in reporting, classification, and management of adverse events

As participants who progress will not require knowledge of which treatment arm they were assigned to for selection of subsequent therapies, blinding will be maintained even after disease progression. The Sponsor's central protocol team (including but not limited to clinical, statistics, and data management) will remain blinded to treatment assignment throughout the duration of the study until the primary endpoint of RFS has been reached or the decision is made to stop the study.

5.4.6 Rationale for Evaluation of Biomarkers

Immune checkpoint signaling through immune inhibitory pathways such as the PD-1 axis to its ligand (PD-L1) and/or LAG-3 interaction with its ligands significantly dampens anti-tumor immune responses in different tumor types, including melanoma, NSCLC, and HCC. Unfortunately, not all patients respond to immune checkpoint blockade. Therefore, there is a continued medical need for a better understanding of factors (eg, biomarkers) that predict benefit and recurrence free survival (RFS).

Data from CA224020 demonstrated that participants with LAG-3 expression are more likely to respond to treatment with nivo + rela FDC in those with advanced melanoma with prior anti-PD-1/PD-L1 treatment.[13,14] In the CA224047 study of patients with previously untreated metastatic or unresectable melanoma, longer PFS was observed in participants with LAG-3 expression treated with either nivolumab monotherapy or nivo + rela FDC. In the current trial, biomarkers, such as LAG-3, PD-L1, MHC-II, FGL-1, and gene expression signatures will be evaluated for potential associations with clinical outcome. Serum, plasma, and peripheral blood immune cell analysis will be assessed in samples collected pretreatment and at time points during treatment. These analyses will allow for identification of potentially predictive biomarkers and pharmacodynamic changes in biomarkers after treatment. An emerging body of clinical evidence has shown that patients with detectable plasma ctDNA following complete resection of their tumors are at higher risk of disease

Approved v4.0 930169531 4.0

recurrence than those patients with undetectable ctDNA after resection. Pre-treatment and on-treatment ctDNA will be evaluated for potential associations with clinical outcome in this study. Additional analysis of tumor biopsies pre-treatment, on-treatment, and upon recurrence will be performed in order to identify additional pathways contributing to therapeutic benefit and/or resistance. See Section 9.8, Biomarkers, for additional details.

5.4.7 Rationale for Inclusion of Adolescent Participants

Advanced melanoma is generally regarded as a similar disease in adolescents and adults and is treated similarly. Metastatic melanoma does not commonly occur in the pediatric population from age 0 to less than 12 years.[34] The incidence reported in the US population for the age groups of 1 to 5 years, 5 to 9 years, and 10 to 14 years is 0.10, 0.16, and 0.37 per 100,000, respectively. The incidence in the US is higher in adolescents: 1.72/100,000 (15 to 19 years) (The SEER Carcinoma Statistics Review).[35] In the European Cooperative Study Group for Pediatric Rare Tumors (EXPeRT) study of melanoma incidence in Italy, Poland, Germany, and France, incidence of cutaneous melanoma was reported as 0.7 to 0.8 per million per year for ages 0 to 10 years and 10 per million per year for ages 15 to 19 years.[36] Overall, the pediatric incidence rates (5.4 per 1 million children and adolescents in the US)[37] are dramatically lower than the adult incidence and add to the difficulty of evaluating adolescent melanoma in clinical trials.

The key primary tumor characteristics, such as the site of the primary tumor, stage at diagnosis, tumor thickness, and level of invasion, are comparable between adolescent and adult melanoma patients. In an analysis of 1255 children (age younger than 20 years), the 10- to 19-year-old age group had similar baseline characteristics compared with the 20- to 24-year-old age group.[38] There are limited clinical studies evaluating treatment outcomes in pediatric and adolescent participants with melanoma. Despite the small number of participants, results of these studies showed that safety profiles and treatment effects (such as tumor shrinkage or pharmacodynamic effects of immunotherapy) in pediatric participants are comparable with adult participants.

Current treatment options for adolescent melanoma are the same as adults as specific guidelines for treatment do not exist. In short, biology is similar and treatment options are also similar as adults. The FDA approval in Mar-2022 of Opdualag™ for the treatment of adult and adolescent patients (12 years and older and weighing at least 40 kg) with unresectable or metastatic melanoma provides further justification for the inclusion of this population to cover an unmet need in future studies.

5.4.8 Rationale for Ultrasound in Disease Surveillance

Ultrasound is one of several imaging modalities (ie, CT scan, chest x-ray) and non-imaging modalities (ie, physical exam, blood tests) that have been applied in the follow-up of melanoma patients, all of which have had varying levels of effectiveness for detection of recurrence.[39] In a meta-analyses of 74 studies spanning 10,528 melanoma patients, ultrasonography was noted to have the greatest sensitivity, specificity, and diagnostic odds ratio for staging of regional lymph nodes as well as for surveillance of lymph node involvement as compared to CT, positron emission tomography (PET), and PET-CT.[40] National Comprehensive Cancer Network (NCCN) Guidelines

Approved v4.0 930169531 4.0

(V2.2021) include nodal basin ultrasound surveillance as preferred over complete lymph node dissection (CLND) in the management and primary treatment of Stage III melanoma patients.[39] In the Multicenter Selective Lymphadenectomy Trial (MSLT-II), it was determined that CLND did not lead to an increase in melanoma-specific survival in patients with sentinel lymph-node metastases compared to observation with ultrasonography.[41] In sum, the evidence and management guidelines to date suggest a role for ultrasonography in the follow-up melanoma patients and supports implementation of ultrasonography in this study for participants with SLND only.

5.4.9 Rationale for MRI Surveillance of Head and Neck Mucosal Melanomas

Though mucosal melanomas of the head and neck are rare tumors, they account for 55.4% of cases of mucosal melanomas. The majority of these arise in the nasal cavity followed by paranasal sinuses, oral cavity, and rarely the pharynx and larynx. The risk of lymph node involvement is significantly higher in oral mucosal melanomas, occurring in about 26% of all cases of oral mucosal melanomas, as compared to nasal mucosal melanomas. Given the likelihood of lymph node spread, metastatic regions of spread may include the brain, lungs, and liver. Imaging plays a vital role for the evaluation of these melanomas given the invasion into adjacent soft tissue and bone erosion. Enhanced imaging techniques such as MRI are particularly helpful in identifying sino-nasal melanomas as iso-attenuating or hyperattenuating mass-like lesions, especially in cases of bone involvement due to regional bone remodeling, erosive, or destructive changes.[42]

5.5 Justification for Dose

5.5.1 Justification for Fixed Dose Combination Dosing

The proposed dose of the FDC product is 480 mg nivolumab + 160 mg relatlimab Q4W. The dose and dosing regimen for this study was primarily based on the benefit/risk profile in metastatic melanoma participants from Study CA224047 and CA224020 pharmacokinetics (PK), pharmacodynamics, and extensive nivolumab monotherapy clinical experience.[8,13] The flat E-R relationship for clinical efficacy and safety including data from higher doses supported the proposed dose for the adjuvant treatment of melanoma.

While administration of FDC (3:1) using a 30-minute infusion time has not been evaluated in participants with cancer, no clinically meaningful differences in the frequency of hypersensitivity/infusion-related reactions are expected when FDC is administered over a 30-minute infusion compared with the 60-minute infusion.[8] Clinical studies of FDC for the treatment of cancer used a 60-minute infusion duration. Infusion reactions, including high-grade hypersensitivity reactions, were uncommon across the relatlimab clinical program. The length of infusion of 30- or 60-minutes is not expected to impact the PK profile of nivolumab and relatlimab. Overall, an infusion duration of 30 minutes for nivo + rela FDC is likewise not anticipated to present significant safety concerns (ie, the frequency of hypersensitivity/infusion-related reactions). The shorter infusion will provide patient convenience by reducing the overall infusion duration and chair time.

Approved v4.0 930169531 4.0

Study CA224020 provides a robust safety experience with nivolumab and relatlimab combination over the broad dose range of 480 mg and 160 mg to 480 mg and 1440 mg Q4W levels, respectively. Nivolumab and relatlimab was safely administered sequentially (nivolumab was given first, followed by relatlimab within 15 to 30 minutes of completing the infusion of nivolumab) up to a doses of 480 mg nivolumab + 1,440 mg relatlimab Q4W, which provides similar infusion rate of ~21 mg/min as the proposed infusion of nivo + rela FDC in this study.[8]

No apparent dose association was observed for infusion reactions and hypersensitivity reactions. (No cases were reported at nivolumab 480 mg + relatlimab 160 mg Q4W dose level [0/17 participants] and 1 event [1/15 participants] at nivolumab 480 mg + relatlimab 1440 mg Q4W dose level was Grade 1/2 and manageable.)

All infusion-related reactions reported with co-administration of nivolumab 480 mg + relatlimab 480 mg infused over 60 minutes were Grade 1-2 adverse events and were manageable.[8]

Based on PPK analysis, relatlimab baseline CL in subjects receiving relatlimab monotherapy and nivolumab + relatlimab SAV (sequential or co-administered) was similar to participants receiving nivo + rela FDC (≤ 5% and ≤ 18% difference, respectively). Similarly, nivolumab baseline CL in subjects receiving nivolumab monotherapy or nivolumab + relatlimab was similar (≤ 5% difference) to participants receiving nivo + rela FDC, thus indicating no clinically relevant effect of FDC formulation on relatlimab or nivolumab PK. Nivolumab and relatlimab both exhibit time-varying clearance (CL) associated with improving disease status over time in metastatic solid tumor patient population. However, in the adjuvant setting, participants are expected to have improved disease status at baseline and in turn clearance is expected to be time invariant and lower than baseline CL observed in metastatic solid tumors patient population. For example, nivolumab CL in adjuvant melanoma participants was 40% lower than baseline CL and approximately 20% lower than steady-state CL relative to participants with advanced melanoma.[43] These changes in CL and resulting increase in exposure is not expected to be clinically relevant given the flat E-R for safety in the metastatic setting.[8]

5.5.2 Dose Rationale for Adolescent Participants

The nivo + rela FDC dose in adolescent patients for this indication is supported by evidence from RELATIVITY 047, a global Phase 3, double-blinded, randomized controlled study in adults with unresectable or metastatic melanoma; additional data analyses that suggest that nivolumab and relatlimab exposures in pediatric patients 12 years of age or older are expected to result in similar safety and efficacy to that of adults. The pharmacokinetics of monoclonal antibodies and the course of unresectable or metastatic melanoma are sufficiently similar in adults and pediatric patients 12 years of age or older to allow extrapolation of data from adults to pediatric patients 12 years of age or older. In addition, overall exposure in adolescent participants in an adjuvant setting is expected to be well within established adult safe and tolerable exposure margin. Relatlimab flat doses of 20 to 800 mg Q2W as monotherapy and up to 1440 mg Q4W in combination with nivolumab 480 mg have been studied in adults.[44]

Approved v4.0 930169531 4.0

Nivo + rela FDC was administered as flat dosing in adults, therefore, a minimum body weight threshold in adolescents (≥ 40 kg) is defined to receive the same adult flat dose to prevent exceeding target adult exposures. Adolescents < 40 kg will be administered body weight adjusted nivo + rela FDC doses, up to the respective maximum adult flat-doses. Adolescent participants ≥ 12 years old who weigh ≥ 40 kg will be administered a flat-dose of nivo + rela FDC (nivolumab 480 mg and relatlimab 160 mg Q4W IV infusion [adult dosing]) or nivolumab monotherapy 480 mg Q4W IV infusion (adult dosing). Participants between ≥ 12 years and < 18 years of age who weigh < 40 kg will be administered body weight adjusted nivolumab 6 mg/kg and relatlimab dose 2 mg/kg Q4W of nivo + rela FDC or nivolumab monotherapy 6 mg/kg Q4W IV infusion over approximately 30 minutes.

5.5.3 Justification for Nivolumab Monotherapy Dose

The nivolumab dose of 480 mg Q4W was selected based on clinical data and modeling and simulation approaches using population PK (PPK) and exposure-response (E-R) analyses of data from studies in multiple tumor types (melanoma, NSCLC, and RCC) where body weight normalized dosing (mg/kg) was used.

Nivolumab PK has been extensively studied in multiple tumor types, including melanoma, NSCLC, RCC, cHL, SCCHN, CRC, and urothelial carcinoma and has been safely administered at doses up to 10 mg/kg Q2W. Nivolumab monotherapy was originally approved as a body-weight based dose of 3 mg/kg Q2W, and was updated to 240 mg Q2W or 480 mg Q4W in multiple indications.[6,8] Nivolumab 360 mg Q3W is also under evaluation in monotherapy and in combination therapy studies. Less frequent 360 mg Q3W and 480 mg Q4W dosing regimens can reduce the burden to patients of frequent, lengthy IV treatments and allow combination of nivolumab with other agents using alternative dosing regimens.

The benefit-risk profiles of nivolumab 240 mg Q2W, 360 mg Q3W and 480 mg Q4W are predicted to be comparable to 3 mg/kg Q2W. This assessment is based on a comprehensive characterization of nivolumab PK, safety, efficacy, and exposure-response relationships across indications. PPK analyses have shown that the PK of nivolumab is linear with proportional exposures over a dose range of 0.1 to 10 mg/kg; no clinically meaningful differences in PK across ethnicities and tumor types were observed. Using the PPK model, the exposures following administration of several dosing regimens of nivolumab administered as a flat dose were simulated, including 240 mg Q2W, 360 mg Q3W, and 480 mg Q4W. The simulated average serum concentration at steady state [Cavgss] following administration of nivolumab 360 mg Q3W and 480 mg Q4W are predicted to be similar to those following administration of nivolumab 240 mg Q2W and nivolumab 3 mg/kg Q2W administered to patients over a wide body weight range (34-180 kg) across tumor types.

Extensive E-R analyses of multiple PK measures (maximum serum concentration at Day 1[Cmax1], average serum concentration at Day 28 [Cavg28], and trough serum concentration at Day 28 [Cmin28]) and efficacy and safety endpoints indicated that the efficacy of the flat-dose 480 mg IV regimen are similar to that of 3 mg/kg Q2W IV regimen. In E-R efficacy analyses for OS and ORR conducted in melanoma, RCC, and NSCLC using Cavg28 as the exposure measure, probabilities of achieving a response and survival probabilities at 1 year and 2 years for IV

Approved v4.0 930169531 4.0

480 mg Q4W were similar to that of IV 3 mg/kg Q2W. In E-R safety analyses, it was demonstrated that the exposure margins for safety are maintained following nivolumab 480 mg Q4W, and the predicted risks of discontinuations due to AEs or death, AE Grade 3+, and immune-mediated AEs (IMAEs) Grade 2+ are similar following nivolumab 480 mg Q4W relative to nivolumab 3 mg/kg Q2W across tumor types. In addition, nivolumab exposures with 240 mg Q2W, 360 mg Q3W, and 480 mg Q4W flat-dose IV regimens across tumor types are maintained well below the corresponding exposures observed with the well-tolerated 10 mg/kg IV nivolumab Q2W dose regimen.

Additional details on nivolumab posologies and risk-benefit can be found in the nivolumab IB.

5.5.4 Rationale for Continued Adjuvant Therapy for Patients Who Are Diagnosed with Melanoma in Situ During Study Treatment

Malignant melanoma in situ (MMIS) Stage 0 includes Tis, N0, M0, demonstrates a radial growth phase in which the proliferation of malignant melanocytes is confined to the epidermis. Patients with a new Stage 0 melanoma are at minimal risk from that new tumor. The risk of death develops from advanced Stage IIIB/C/D or Stage IV melanoma for which they are receiving treatment during the clinical trial. Opdivo (nivolumab) is approved for the adjuvant treatment of adult patients with melanoma with involvement of lymph nodes or metastatic disease who have undergone complete resection. Consequently, in the absence of intolerable toxicity, patients with diagnosed MMIS should continue adjuvant therapy using the same dose and schedule for their original melanoma for a maximum of 1-year total duration starting from first dose (maximum of 13 doses). Melanoma in situ will not be considered a new primary malignancy but will be captured as a data point.

5.6 Clinical Pharmacology Summary

5.6.1 Nivolumab Clinical Pharmacology Summary

Nivolumab pharmacokinetics (PK) was assessed using a population PK approach for single agent nivolumab.

Nivolumab as a single agent: The PK of single-agent nivolumab was studied in patients over a dose range of 0.1 to 20 mg/kg administered as a single dose or as multiple doses of nivolumab as a 60-minute intravenous infusion every 2 or 3 weeks. Nivolumab CL decreases over time, with a mean maximal reduction (% coefficient of variation [CV%]) from baseline values of 24.5% (47.6%) resulting in a geometric mean steady-state clearance (CLss) (CV%) of 8.2 mL/h (53.9%) in patients with metastatic tumors; the decrease in CLss is not considered clinically relevant. Nivolumab clearance does not decrease over time in patients with completely resected melanoma, as the geometric mean population clearance is 24% lower in this patient population compared with patients with metastatic melanoma at steady state. The geometric mean volume of distribution at steady state (Vss) (CV%) is 6.8 L (27.3%), and geometric mean elimination half-life (t1/2) is 25 days (77.5%). Steady-state concentrations of nivolumab were reached by 12 weeks when administered at 3 mg/kg every 2 weeks, and systemic accumulation was 3.7-fold. The exposure to nivolumab increases dose proportionally over the dose range of 0.1 to 10 mg/kg administered every

Approved v4.0 930169531 4.0

2 weeks. The predicted exposure (Cavg and Cmax) of nivolumab after a 30-minute infusion is comparable to that observed with a 60-minute infusion.

Specific Populations: The population PK analysis suggested that the following factors had no clinically important effect on the clearance of nivolumab: age (29 to 87 years), weight (35 to 160 kg), gender, race, baseline lactate dehydrogenase (LDH), PD-L1 expression, solid tumor type, tumor size, renal impairment, and mild hepatic impairment.

Renal Impairment: The effect of renal impairment on the clearance of nivolumab was evaluated by a population PK analysis in patients with mild (estimated glomerular filtration rate [eGFR] 60 to 89 mL/min/1.73 m^2), moderate (eGFR 30 to 59 mL/min/1.73 m^2), or severe (eGFR 15 to 29 mL/min/1.73 m^2) renal impairment. No clinically important differences in the clearance of nivolumab were found between patients with renal impairment and patients with normal renal function.

Hepatic Impairment: The effect of hepatic impairment on the clearance of nivolumab was evaluated by population PK analyses in patients with HCC and in patients with other tumors with mild hepatic impairment (total bilirubin [TB] less than or equal to the upper limit of normal (ULN) and AST greater than ULN or TB greater than 1 to 1.5 times ULN and any AST) and in HCC patients with moderate hepatic impairment (TB greater than 1.5 to 3 times ULN and any AST). No clinically important differences in the clearance of nivolumab were found between patients with mild/moderate hepatic impairment.

In addition, nivolumab pharmacokinetics (PK) was characterized in adults and pediatric participants with solid tumors who received nivolumab alone and in combination with relatlimab. Nivolumab exhibits linear and time-varying PK. Nivolumab baseline clearance (CL) in participants receiving nivolumab monotherapy or nivolumab + relatlimab was similar (≤ 5% difference) to participants receiving nivolumab + relatlimab FDC. The magnitude of the reductions in nivolumab CL over time were also similar between relatlimab in combination with nivolumab and nivolumab monotherapy with a maximal reduction of 21.1% in adult 1L melanoma participants. Thus, no PK interaction between nivolumab and relatlimab was observed when these agents were given in combination.

Full details on the clinical pharmacology aspects of nivolumab can be found in the IB and product label.

5.6.2 Relatlimab Clinical Pharmacology Summary

The PK of relatlimab was characterized over a wide range of doses; 20 mg to 800 mg Q2W when administered as monotherapy or 20 mg to 240 mg Q2W and 160 mg to 1440 mg Q4W when administered in combination with nivolumab.[8]

The exposure to relatlimab, Cmax, and area under the concentration-time curve in 1 dosing interval (AUC[TAU]) increased approximately dose proportionally over a dose range of 20 mg to 800 mg Q2W or 160 mg to 1440 mg Q4W. The geometric mean accumulation index values based on AUC(TAU) were approximately 1.3- to 3-fold. The geometric mean of total clearance was 8.9 to 12.9 mL/h and the mean effective half-life ranged between ~12 to 28 days across dose groups.

Approved v4.0 930169531 4.0

Relatlimab exhibits non-linear and time-varying PK. Nonlinearity in relatlimab CL represents ~31% of total CL of relatlimab at the relatlimab 160-mg dose Q4W in combination with nivolumab. Relatlimab clearance coefficient of variation% (CV%) is 9.7% lower (geometric mean, 5.48 mL/h [41.3%]) at steady state than after the first dose (6.06 mL/h [38.9%]) and ~ 97% of the maximum concentration at steady state (Cmaxss) is predicted to be eliminated (approximating 5× half-lives) with a geometric mean (CV%) of 68.6 (35.7%) days. The geometric mean value (CV%) for relatlimab volume of distribution at steady state is 6.65 L (19.8%), consistent with the expected distribution of monoclonal antibodies (mAbs) limited to the vascular space. Relatlimab baseline CL in participants receiving relatlimab monotherapy and nivolumab + relatlimab single-agent vial (SAV) (sequential or coadministered) was similar to participants receiving nivolumab + relatlimab FDC (≤ 5% and ≤ 18% difference, respectively), indicating no clinically relevant effect of nivolumab on relatlimab PK.

The incidence of anti-relatlimab antibodies and anti-nivolumab antibodies was ~10 % and ~11%, respectively. The immunogenicity of relatlimab when given in combination with nivolumab has minimal clinical relevance as nivolumab and relatlimab antibodies (ADAs) have no effects on PK, safety, or efficacy profiles. Further details on the clinical pharmacology aspects of relatlimab can be found in the relatlimab IB.

6 STUDY POPULATION

Prospective approval of protocol deviations to recruitment and enrollment criteria, also known as protocol waivers or exemptions, is not permitted.

6.1 Inclusion Criteria

Participants are eligible to be included in the study only if all of the following criteria apply:

1) **Signed Written Informed Consent**
    a) Participants or their legally acceptable representative (LAR; see Appendix 2) must have signed and dated an Institutional Review Board (IRB)/Independent Ethics Committee (IEC)-approved written informed consent form (ICF) in accordance with regulatory, local, and institutional guidelines. This must be obtained before the performance of any protocol-related procedures that are not part of normal patient care.
    b) Participants must be willing and able to comply with scheduled visits, treatment schedule, laboratory tests, tumor biopsies, and other requirements of the study.
    c) Participant Re-enrollment: This study permits the re-enrollment of a participant that has discontinued the study as a pre-treatment failure (ie, participant has not been randomized/has not been treated). If re-enrolled, the participant must be re-consented.
2) **Type of Participant and Target Disease Characteristics**
    a) All participants must have been diagnosed with either Stage IIIA (> 1 mm tumor in lymph node)/B/C/D or Stage IV melanoma by AJCC v8 and have histologically confirmed melanoma that is completely surgically resected (free of disease) with negative margins in order to be eligible. All melanomas, except ocular melanoma, regardless of primary site of disease, will be allowed.

Approved v4.0 930169531 4.0

Note: Conjunctival melanoma is not considered to be ocular melanoma and is to be classified as mucosal melanoma.

b) Participants are eligible if central nervous system (CNS) metastases have been resected and participants are neurologically stable.
   i) Prior resected CNS metastases must be without evidence of recurrence, as determined by magnetic resonance imaging (MRI) performed at least 4 weeks after resection is complete and within 35 days prior to randomization.
   ii) Participants must be off immunosuppressive doses of systemic steroids (>10 mg/day prednisone or equivalent) for at least 14 days prior to study drug administration and must have returned to neurologic baseline post-operatively.
   iii) For CNS lesion(s), a pathology report indicating that there has been complete resection of CNS lesion(s) will suffice as confirmation of negative margins.

c) Complete resection must be performed within 90 days prior to randomization. Management of residual lymph nodes after positive sentinel lymph node biopsy (SLNB) (ie, completion lymph node dissection) will be as per local standards and recommendations for the individual participant.

d) All participants must have disease-free status documented by a complete physical examination within 14 days prior to randomization and imaging studies within 35 days prior to randomization. Imaging studies must include CT scan of the chest, abdomen, pelvis, and all known sites of resected disease, and brain MRI. See Section 9.1.2 for details and exceptions.

e) A FFPE tissue block (strongly preferred) containing 20 mm^3 of tumor tissue or 20 positively charged unstained slides (minimum of 15) of tumor tissue obtained from surgical specimen or biopsy during resection (core biopsy, punch biopsy, or excisional biopsy) collected within 90 days prior to randomization, with no intervening systemic anti-cancer treatment between time of acquisition and enrollment, with an associated pathology report, must be submitted to the central laboratory (preferably prior to randomization). Fine needle aspirates or other cytology samples are not acceptable. Biopsies of bone lesions that do not have a soft tissue component are not acceptable. If insufficient tumor tissue content is provided for analysis, acquisition of additional tumor tissue (block and/or slides) for the biomarker analysis will be requested.

   Please refer to Section 9.8 for additional details on tumor tissue requirements.

f) Participants ≥ 18 years of age must have an ECOG performance status of ≤ 1. Adolescent participants between 12 and < 18 years of age must have a Lansky/Karnofsky performance score ≥ 80% (Appendix 6).

g) Not applicable - China only.

3) Age of Participant

a) Not applicable per Protocol Amendment 01.

b) Participant must be ≥ 12 years of age or local age of majority inclusive, at the time of signing the informed consent.

   Except: Where local regulations do not allow for participants < 18 years of age (adolescent population) to participate. For those sites, the eligible participant population is ≥ 18 years of age. Refer to Appendix 9.

Approved v 4.0 930169531 4.0

4) **Reproductive Status**

Investigators shall counsel women of child bearing potential (WOCBP) on the importance of pregnancy prevention, the implications of an unexpected pregnancy, and the potential of fetal toxicity occurring due to transmission of study intervention to a developing fetus.

The investigator shall evaluate the effectiveness of the contraceptive method in relationship to the first dose of study intervention.

Local laws and regulations may require the use of alternative and/or additional contraception methods.

a) **Female Participants:**

i) Female participants must have documented proof that they are not of childbearing potential.

ii) Women who are not of childbearing potential are exempt from contraceptive requirements.

iii) WOCBP must have a negative highly sensitive negative serum or urine pregnancy test (minimum sensitivity 25 IU/L or equivalent units of HCG) within 24 hours prior to the start of study intervention. An extension up to 72 hours prior to the start of study treatment is permissible in situations where results cannot be obtained within the standard 24-hour window.

(1) If a urine test cannot be confirmed as negative (eg, an ambiguous result), a serum pregnancy test is required. In such cases, the participant must be excluded from participation if the serum pregnancy result is positive.

iv) Additional requirements for pregnancy testing during and after study intervention are located in Section 2, Schedule of Activities.

v) The investigator is responsible for review of medical history, menstrual history, and recent sexual activity to decrease the risk for inclusion of a woman with an early undetected pregnancy.

vi) WOCBP must agree to follow instructions for method(s) of contraception defined in Appendix 4 and as described below and included in the ICF.

vii) WOCBP are permitted to use hormonal contraception methods (as described in Appendix 4).

viii) A female participant is eligible to participate if she is not pregnant or breastfeeding, and at least 1 of the following conditions applies:

(1) Is not a WOCBP

OR

(2) Is a WOCBP and using a contraceptive method that is highly effective as described in Appendix 4 during screening and for the duration of treatment for a total of 5 months after the last dose and agrees not to donate eggs (ova, oocytes) for the purpose of reproduction for the same time period.

b) **Male Participants:**

i) No additional contraceptive measures are required to be used.

Approved v4.0 930169531 4.0

6.2 Exclusion Criteria

Participants are excluded from the study if any of the following criteria apply:

1) Medical Conditions

a) History of ocular melanoma.

Note: Conjunctival melanoma is not considered to be ocular melanoma and is to be classified as mucosal melanoma.

b) Untreated/unresected CNS metastases or leptomeningeal metastases.

c) Participants with an active, known or suspected autoimmune disease. Participants with type I diabetes mellitus, hypothyroidism only requiring hormone replacement, skin disorders (such as vitiligo, psoriasis, or alopecia) not requiring systemic treatment, or conditions not expected to recur in the absence of an external trigger are permitted to enroll.

d) Participants with serious or uncontrolled medical disorder.

e) Previous severe acute respiratory syndrome coronavirus 2 (SARS-CoV-2) infection either suspected or confirmed within 4 weeks prior to screening. Acute symptoms must have resolved and based on investigator assessment, there are no sequelae that would place the participant at a higher risk of receiving investigational treatment.

f) Concurrent non-melanoma malignancy (present during screening) requiring treatment or history of prior malignancy active within 2 years prior to randomization (i.e. participants with a history of prior malignancy are eligible if treatment was completed at least 2 years before randomization and the participant has no evidence of disease). Participants with history of prior early stage basal/squamous cell skin cancer or non-invasive or in situ cancers that have undergone definitive treatment at any time are also eligible.

g) Participants with a condition requiring systemic treatment with either corticosteroids (> 10 mg daily prednisone equivalent) within 14 days or other immunosuppressive medications within 30 days of start of study treatment. Inhaled or topical steroids, and adrenal replacement steroid doses > 10 mg daily prednisone equivalent, are permitted in the absence of active autoimmune disease.

h) Woman who are breastfeeding.

i) Participants with a history of myocarditis, regardless of etiology.

2) Prior/Concomitant Therapy

a) Prior immunotherapy treatment for any prior malignancy: No prior immunotherapies are permitted (such as, but not limited to anti-PD-1, anti-PD-L1, anti-PD-L2, or any other antibody or drug specifically targeting T-cell costimulation or immune checkpoint pathways).

b) Prior treatment with LAG-3 targeted agents.

c) Participants treated with anti-cancer therapy directed against the resected melanoma (for example, but not limited to, systemic, local, radiation, and radiopharmaceuticals) except:

i) Surgery for the melanoma lesion(s)

ii) Adjuvant radiation therapy after neurosurgical resection for CNS lesions

iii) Prior adjuvant interferon completed ≥ 6 weeks prior to randomization

Approved v4.0 930169531 4.0

d) Treatment with complementary medications (eg, herbal supplements or traditional Chinese medicines) to treat the disease under study within 2 weeks prior to randomization. Such medications are permitted if they are used as supportive care. Refer to Section 7.7.1 for prohibited therapies.

e) Treatment with any live/attenuated vaccine within 30 days of first study treatment (inactivated vaccines are permitted).

f) Participants currently in other interventional trials, including those for COVID-19, until the protocol specific washout period is achieved. If a study participant has received an investigational COVID-19 vaccine or other investigational product designed to treat or prevent COVID-19 prior to screening, enrollment must be delayed until the full dosing schedule of the vaccine has been completed and the biologic impact of the vaccine or investigational product is stabilized, unless the delay would compromise the participant's health or suitability for enrollment as determined by discussion between the investigator and the Medical Monitor/designee.

g) Prior treatment with BRAF/MEK targeted agents.

h) Prior radiation therapy within 2 weeks prior to first dose of study medication. Participants must have recovered (ie, Grade ≤ 1 or at baseline) from radiation-related toxicities prior to first study treatment.

3) **Physical and Laboratory Test Findings**

a) Positive pregnancy test at enrollment or prior to administration of study medication.

Note: May not transfuse, use growth factors, and/or coagulation factors within 14 days of randomization to meet criteria 3b to 3e.

b) White blood cell count < 2000/μL

c) Neutrophils < 1500/μL

d) Platelets < $100*10^3$/μL

e) Hemoglobin < 9.0 g/dL

f) Serum creatinine > 1.5 × upper limit of normal (ULN), unless calculated creatinine clearance (CrCl) ≥ 40 mL/min (using the Cockcroft-Gault formula)

g) Aspartate transaminase (AST) / alanine aminotransferase (ALT) > 3.0× ULN

h) Total bilirubin > 1.5 × ULN (except participants with Gilbert Syndrome who must have a total bilirubin level of < 3.0x ULN)

i) Any positive test result for hepatitis B virus (HBV) indicating presence of virus (eg, Hepatitis B surface antigen [HBsAg, Australia antigen]) positive.

j) Any positive test result for hepatitis C virus (HCV) indicating presence of active viral replication (detectable HCV-ribonucleic acid [RNA]). Note: Participants with positive HCV antibody and an undetectable HCV RNA are eligible to enroll.

k) Known human immunodeficiency virus (HIV) positive with an AIDS defining opportunistic infection within the last year, or a current CD4 count < 350 cells/uL. Participants with HIV are eligible if:

i) they have received antiretroviral therapy (ART dosing) for at least 4 weeks prior to randomizations as clinically indicated while enrolled on study

ii) they continue on ART as clinically indicated while enrolled on study

Approved v4.0 930169531 4.0

iii) CD4 counts and viral load are monitored per standard of care by a local health care provider.

NOTE: Testing for HIV must be performed at sites where mandated locally. HIV positive participants must be excluded where mandated locally (see Appendix 9).

l) Evidence of organ dysfunction or any clinically significant deviation from normal in physical examination, vital sign, electrocardiograms, or clinical laboratory determinations beyond what is consistent with the target population.

m) Troponin T (TnT) or I (TnI) > 2 × institutional ULN. Participants with TnT or TnI levels between > 1 to 2 × ULN will be permitted if repeat levels within 24 hours are ≤ 1 × ULN. If TnT or TnI levels are between >1 to 2 × ULN within 24 hours, the participant must undergo a cardiology consultation and cardiac evaluation and be considered for treatment, based on a favorable benefit/risk assessment by the Investigator. When repeat levels within 24 hours are not available, a repeat test should be conducted as soon as possible. If TnT or TnI repeat levels beyond 24 hours are < 2 × ULN, the participant must undergo a cardiology consultation and cardiac evaluation and be considered for treatment, based on a favorable benefit-risk assessment by the Investigator. Please refer to Section 9.4.5 for additional details.

4) Allergies and Adverse Drug Reaction

a) Participants with history of allergy or hypersensitivity to study treatment components.

b) History of life-threatening toxicity related to prior immune therapy (e.g. anti-CTLA4 or anti-PD-1/PD-L1 treatment or any other antibody or drug specifically targeting T-cell co-stimulation or immune checkpoint pathways) except those that are unlikely to re-occur with standard countermeasures (eg, hypothyroidism).

5) Other Exclusion Criteria

a) Prisoners or participants who are involuntarily incarcerated. (Note: Under certain specific circumstances and only in countries where local regulations permit, a person who has been imprisoned may be included or permitted to continue as a participant. Strict conditions apply, and BMS approval is required. [Please refer to Appendix 9 for Greece-specific differences.])

b) Participants who are compulsorily detained for treatment of either a psychiatric or physical (eg, infection illness).

Eligibility criteria for this study have been carefully considered to ensure the safety of the study participants and that the results of the study can be used. It is imperative that participants fully meet all eligibility criteria.

6.3 Lifestyle Restrictions

Not applicable. No restrictions are required.

6.4 Screen Failures

Screen failures are defined as participants who consent to participate in the clinical study but who are not subsequently randomized in the study/included in the analysis population. A minimal set of screen failure information is required to ensure transparent reporting of screen failure

Approved v4.0 930169531 4.0

participants, to meet the Consolidated Standards of Reporting Trials (CONSORT) publishing requirements, as applicable, and to respond to queries from regulatory authorities. Minimal information includes date of consent, demography, screen failure details, eligibility criteria, and any serious AEs.

6.4.1 Retesting During Screening or Lead-in Period

Participant Re-enrollment: This study permits the re-enrollment of a participant who has discontinued the study as a pretreatment failure (ie, participant has not been randomized/has not been treated). If re-enrolled, the participant must be re-consented.

Retesting of laboratory parameters and/or other assessments within any single Screening period will be permitted (in addition to any parameters that require a confirmatory value).

The most current result prior to randomization is the value by which study inclusion will be assessed, because it represents the participant's most current clinical state.

Laboratory parameters and/or assessments that are included in Screening Procedural Outline (see Table 2-1), may be repeated in an effort to find all possible well-qualified participants. Consultation with the Medical Monitor/designee may be needed to identify whether repeat testing of any particular parameter is clinically relevant.

Testing for asymptomatic SARS-CoV-2 infection, for example by RT-PCR or viral antigen is not required. However, some participants may develop suspected or confirmed symptomatic SARS-CoV-2 infection, or be discovered to have asymptomatic SARS-CoV-2 infection during the screening period. In such cases, participants may be considered eligible for the study after meeting all inclusion/exclusion criteria related to active infection, and after meeting the following criteria:

- At least 10 days (4 weeks for severe/critical illness) have passed since symptoms first appeared or positive RT-PCR or viral antigen test result.
- At least 24 hours have passed since last fever without the use of fever-reducing medications.
- Acute symptoms (eg, cough, shortness of breath) have resolved.
- In the opinion of the investigator, there are no COVID-19-related sequelae that may place the participant at a higher risk of receiving investigational treatment.
- Recommended negative follow-up SARS-CoV-2 RT-PCR or viral antigen test based on institutional, local, or regional guidelines.

7 STUDY INTERVENTION(S) AND CONCOMITANT THERAPY

Study intervention is defined as any investigational intervention(s), marketed product(s), placebo, procedure(s), or medical device intended to be administered to a study participant according to the study protocol.

Study intervention includes both Investigational [Medicinal] Product (IP/IMP) and Non-investigational/Auxiliary [Medicinal] Product (Non-IP/Non-IMP/AxMP) as indicated in Table 7.1-1.

Approved v4.0 930169531 4.0

An IP, also known as IMP in some regions, is defined a pharmaceutical form of an active substance or placebo being tested or used as a reference in a clinical study, including products already with a marketing authorization but used or assembled (formulated or packaged) differently from the authorized form, or used for an unauthorized indication, or when used to gain further information about the authorized form.

Other medications used as support or escape medication for preventative, diagnostic, or therapeutic reasons, as components of the standard of care for a given diagnosis, may be considered as IPs/AxMPs.

7.1 Study Interventions Administered

Table 7.1-1: Study Interventions

ARM Name	Nivo + rela FDC	Nivolumab
**Intervention Name**	BMS-986213 (Nivolumab 240 mg / Relatlimab 80 mg) vial	Nivolumab 100 mg/vial
**Type**	Drug	Drug
**Dose Formulation**	Solution for injection	Solution for injection
**Unit Dose Strength(s)**	16 mg/mL	10 mg/mL
**Dosage Level(s) for Adults (≥ 18 years of age) and Adolescents (≥ 12 years to < 18 years of age) ≥ 40 kg**	Nivolumab 480 mg<br>Relatlimab 160 mg<br>Total: 640 mg<br>Once every 4 weeks	Nivolumab 480 mg<br>Once every 4 weeks
**Dosage Level(s) for Adolescents (≥ 12 years to < 18 years of age) < 40 kg (weight-based)**	Nivolumab 6 mg/kg<br>Relatlimab 2 mg/kg<br>Total: 8 mg/kg<br>Once every 4 weeks	Nivolumab 6 mg/kg<br>Once every 4 weeks
**Route of Administration**	IV infusion	IV infusion
**Use**	Experimental	Active comparator
**IMP and Non-IMP/AxMP**	IMP	IMP
**Sourcing**	Provided centrally by the Sponsor	Provided centrally by the Sponsor
**Packaging and Labeling**	Study intervention will be provided in a kit comprised of 2 vials. Each vial and kit will be labeled as required per country requirement.	Study intervention will be provided in a vial. Each vial will be labeled as required per country requirement.
**Current/Former Name(s) or Alias(es)**	BMS-986213 (Relatlimab 80 mg/Nivolumab 240 mg/vial)[a]<br>Opdualag™	BMS-936558<br>Opdivo™

Abbreviations: AxMP, auxiliary medical product; FDC, fixed dose combination; IMP, investigational medicinal product; IP, investigational product; IV, intravenous; nivo, nivolumab; rela, relatlimab.

[a] Study Intervention for Nivo + Rela FDC Arm is currently BMS-986213 (Relatlimab 80 mg/Nivolumab 240 mg/vial) and will be updated to BMS-986213 (Nivolumab 240 mg/Relatlimab 80 mg/vial) approximately 1Q-2Q 2023.

Approved v4.0 930169531 4.0

For body weight dosing, the dosing calculations should be based on the body weight assessed at screening. Doses must be recalculated if there is a cumulative weight change of 10% or more (whether gained or lost) from screening weight. All doses should be rounded to the nearest milligram. For an adolescent who weighs < 40 kg reaching 18 years of age or an adolescent whose weight reaches ≥ 40 kg, the dose calculation should continue to be based on the body weight and not switched to flat dosing.

7.1.1 Study Treatment Details

Participants will receive nivo + rela FDC or nivolumab monotherapy on Day 1 of every 4-week cycle. Blinded study drug will be administered in a single bag IV over approximately 30 minutes. Participants should be carefully monitored for infusion reactions during IV administration. If an acute infusion reaction is noted, participant should be managed according to Section 7.4.4.

- There will be no dose escalations or reductions of immunotherapy allowed.
- Premedications are not recommended for the first dose of immunotherapy.
- Participants should be carefully monitored for infusion reactions during immunotherapy administration.
- If an acute infusion reaction is noted, participants should be managed according to Section 7.4.4.
- Participants should receive immunotherapy until recurrence (excluding melanoma in-situ, see Section 5.5.4), unacceptable toxicity, withdrawal of consent, a maximum treatment duration of 1 year from first dose (maximum of 13 doses), or the study ends, whichever occurs first.
- Doses of immunotherapy may be interrupted, delayed, or discontinued depending on how well the participant tolerates the treatment according to Section 7.4.1.
- Dosing visits must not be skipped, only delayed. See Section 7.4 and Section 8.1.

All infusions must be promptly followed by a diluent flush to clear the line of IP before starting infusion(s) of any additional treatment. Instruction for dilution and infusion of study drug injections will be provided in the Pharmacy Manual. Care must be taken to assure sterility of the prepared solution as the product does not contain any antimicrobial preservative or bacteriostatic agent. For details on prepared drug storage, preparation, and administration, please refer to the IBs and/or Pharmacy Manual. The selection and timing of dose for each participant is provided in Table 7.1-1.

Study treatment will be dispensed by interactive response technology (IRT) at the study visits as listed in Section 2 (Schedule of Activities). Further details regarding preparation and administration will be provided separately in site/pharmacy training materials.

7.2 Method of Study Intervention Assignment

All participants will be centrally randomized using Interactive Response Technology (IRT). Before the study is initiated, each user will receive log-in information and directions on how to access the IRT. After the participant's informed consent has been obtained and initial eligibility is established, the participant must be enrolled into the study by using IRT to obtain the participant

Approved v4.0 930169531 4.0

number. Every participant who signs the ICF must be assigned a participant number in IRT. The investigator or designee will register the participant for enrollment by following the enrollment procedures established by BMS. The following information is required for enrollment:

- Date that informed consent was obtained
- Year of birth
- Gender at birth

After enrollment in the IRT, participants who have met all eligibility criteria will be randomized through the IRT. The following information is required for participant randomization:

- Participant number
- Year of birth
- Melanoma type (cutaneous or mucosal)
- AJCC v8 stage at screening (cutaneous melanoma only)

Participants will be randomized in a 1:1 ratio (see Figure 5.1-1) and stratified by geographic region (USA/Canada/Australia vs Europe vs ROW) and AJCC v8 stage as described in Section 5.2.

The randomization procedures will be carried out via permuted blocks within each stratum, defined by combination of geographic region and AJCC v8 stage. The exact procedures for using the IRT will be detailed in the IRT manual.

7.3 Blinding

This is a double-blind study. Access to treatment codes will be restricted from all participants, and site and Sponsor personnel prior to primary database lock, with exceptions as specified below.

Blinding of study treatment assignment is critical to the integrity of this clinical study. However, in the event of a medical emergency or pregnancy in an individual participant in which knowledge of the IP is critical to the participant's management, the blind for that participant may be broken by the investigator. The participant's safety takes priority over any other considerations in determining if a treatment assignment should be unblinded.

Before breaking the blind of an individual participant's treatment, the investigator should determine that the unblinded information is necessary (ie, that it will alter the participant's immediate management). In many cases, particularly when the emergency is clearly not related to the IP, the problem may be properly managed by assuming that the participant is receiving the IP. It is highly desirable that the decision to unblind treatment assignment be discussed with the Medical Monitor/designee, but the investigator always has ultimate authority for the decision to unblind. The actual task of unblinding can be delegated by the investigator to a designee assigned the task on the Delegation of Authority. The Principal Investigator or appointed designee should only call in for emergency unblinding after the decision to unblind the participant has been documented.

For information on how to unblind in an emergency, consult the IRT manual.

Approved v4.0 930169531 4.0

In cases of accidental unblinding, contact the Medical Monitor/designee and ensure every attempt is made to minimize additional disclosure and the impact of unblinding.

Any request to unblind a participant for nonemergency purposes should be discussed with the Medical Monitor/designee. Discussions regarding unblinding with the Medical Monitor/designee must be documented.

In case of an emergency, the investigator(s) has unrestricted access to randomization information via the IRT and is capable of breaking the blind through the IRT system without prior approval from the Sponsor. Following the unblinding, the Investigator shall notify the Medical Monitor/designee that the unblinding took place.

A scientist in the NonClinical Disposition and Bioanalysis department of BMS (and/or a designee in the external bioanalytical laboratory) will be unblinded to the randomized treatment assignments in order to minimize unnecessary bioanalytical analysis of samples. Any results shared by the NonClinical Disposition and Bioanalysis group with the Sponsor's study team will be blinded to ensure integrity of the study.

Investigators will remain blinded to each participant's assigned study intervention throughout the course of the study. In order to maintain this blind, an otherwise uninvolved third party will be responsible for the reconstitution and dispensation of all study intervention and will endeavor to ensure that there are no differences in time taken to dispense following randomization.

In the event of a Quality Assurance audit, the auditor(s) will be allowed access to unblinded study intervention records at the site(s) to verify that randomization/dispensing has been done accurately.

The DMC will assess safety and risk-benefit on an ongoing basis, and will have access to unblinded treatment codes for individual subjects. An external analysis team (external to BMS), including a reporting statistician and programming support, who are not involved with the conduct of the study, will provide analyses to the DMC. The procedures to be respected and the reasons for unblinding of the DMC are discussed in the DMC charter. The DMC will perform all interim analyses of RFS and OS. The sponsor will be unblinded to individual treatment assignments at the time of any RFS analysis found to be significant or at the time of the final RFS, whichever comes first. Participants, investigator, and site staff will remain blinded to individual treatment assignments until OS result is statistically significant or until final analysis of the OS endpoint, whichever comes first. See Section 10.2.1 for timing of the RFS analyses and Section 10.2.2 for timing of OS analyses.

7.4 Dosage Modification

Dose reductions or dose escalations are not permitted. All dose modification rules apply to all arms, given the blinded nature of this study.

7.4.1 Dose Delay Criteria

Dose delay criteria apply for all drug-related AE. Delay administration of study treatment if any of the delay criteria in Table 7.4.1-1 are met. Delay dosing for any AE, laboratory abnormality, or intercurrent illness which, in the judgement of the investigator, warrants delaying the dose of study medication.

Approved v4.0 930169531 4.0

Dose must be delayed for SARS-CoV-2 infection either confirmed or suspected.

For participants who require delay of study treatment, re-evaluate weekly, or more frequently, if clinically indicated, and resume dosing when criteria to resume treatment are met (see Section 7.4.2). Continue tumor assessments per protocol even if dosing is delayed.

Approved v4.0 930169531 4.0

Table 7.4.1-1: Adverse Event Criteria for Delay, Resume, and Discontinue of Treatment

Drug-Related Adverse Event (AE) per CTCAE V5	Severity	Action Taken	Clarifications, Exceptions, and Resume Criteria
**Gastrointestinal**			
Colitis or Diarrhea	Grade 2	Delay dose	Dosing may resume when AE resolves to baseline
	Grade 3	Delay dose	Dosing may resume when AE resolves to baseline
	Grade 4	Permanently discontinue	
**Renal**			
Serum Creatinine Increased	Grade 2 or 3	Delay dose	Dosing may resume when AE resolves to Grade ≤ 1 or baseline value
	Grade 4	Permanently discontinue	
**Pulmonary**			
Pneumonitis	Grade 2	Delay dose	Dosing may resume after pneumonitis has resolved to ≤ Grade 1.
	Grade 3 or 4	Permanently discontinue	
**Hepatic**			
Aspartate aminotransferase (AST), alanine aminotransferase (ALT), or total bilirubin (T.bili) increased	AST or ALT > 3 × and ≤ 5 × upper limit of normal (ULN) or T.bili >1.5 × and ≤ 3 × ULN, regardless of baseline value	Delay dose	Dosing may resume when laboratory values return to baseline.
	AST or ALT > 5 × ULN or T.bili > 3 × ULN, regardless of baseline value	Permanently discontinue	
	Concurrent AST or ALT > 3 × ULN and T.bili > 2 × ULN, regardless of baseline value	Permanently discontinue	

Table 7.4.1-1: Adverse Event Criteria for Delay, Resume, and Discontinue of Treatment

Drug-Related Adverse Event (AE) per CTCAE V5	Severity	Action Taken	Clarifications, Exceptions, and Resume Criteria
**Endocrinopathy**			
Adrenal Insufficiency	Grade 2 adrenal insufficiency	Delay dose	Dosing may resume after adequately controlled with hormone replacement.
	Grade 3 or 4 adrenal insufficiency or adrenal crisis	Delay dose or permanently discontinue	Mandatory discussion with and documented approval from the Medical Monitor/designee needed prior to resuming therapy. If adrenal insufficiency resolves or is adequately controlled with physiologic hormone replacement, participant may not require discontinuation of study drug.
Hyperglycemia	Hyperglycemia requiring initiation or change in daily management (Grade 2 or 3)	Delay dose	Dosing may resume if hyperglycemia resolves to Grade ≤ 1 or baseline value, or is adequately controlled with glucose-controlling agents.
	Grade 4	Delay dose or permanently discontinue	Mandatory discussion with and documented approval from the Medical Monitor/designee needed prior to resuming therapy. If hyperglycemia resolves, or is adequately controlled with glucose-controlling agents, participant may not require discontinuation of study drug.
Hypophysitis/Hypopituitarism	Symptomatic Grade 1-3 that is also associated with corresponding abnormal lab and/or pituitary scan	Delay dose	Dosing may resume if endocrinopathy resolves to be asymptomatic, or is adequately controlled with only physiologic hormone replacement.
	Grade 4	Delay dose or permanently discontinue	Mandatory discussion with and documented approval from the Medical Monitor/designee needed prior to resuming therapy. If endocrinopathy resolves or is adequately controlled with physiologic hormone replacement, participant may not require discontinuation of study drug.
Hyperthyroidism or Hypothyroidism	Grade 2 or 3	Delay dose	Dosing may resume if endocrinopathy resolves to be asymptomatic, or is adequately controlled with only physiologic hormone replacement or other medical management.
	Grade 4	Delay dose or permanently discontinue	Mandatory discussion with and documented approval from the Medical Monitor/designee needed prior to resuming therapy. If endocrinopathy resolves or is adequately controlled with physiologic hormone replacement or other medical management, participant may not require discontinuation of study drug.

Approved v4.0 930169531 4.0

Table 7.4.1-1: Adverse Event Criteria for Delay, Resume, and Discontinue of Treatment

Drug-Related Adverse Event (AE) per CTCAE V5	Severity	Action Taken	Clarifications, Exceptions, and Resume Criteria
**Skin**			
Rash	Grade 2 rash covering > 30% body surface area or Grade 3 rash	Delay dose	Dosing may resume when rash reduces to ≤ 10% body surface area
	Suspected Stevens-Johnson syndrome (SJS), toxic epidermal necrolysis (TEN) or drug reaction with eosinophilia and systemic symptoms (DRESS)	Delay dose	Dosing may resume if SJS, TEN, or DRESS is ruled out and rash reduces to is ≤ 10% body surface area
	Grade 4 rash or confirmed SJS, TEN, or DRESS	Permanently discontinue	
**Neurological**			
Guillain-Barre Syndrome (GBS)	Any Grade	Permanently discontinue	
Myasthenia Gravis (MG)	Any Grade	Permanently discontinue	
Encephalitis	Any Grade encephalitis	Delay dose	After workup for differential diagnosis, (i.e. infection, tumor-related), if encephalitis is not drug related, then dosing may resume when AE resolves
	Any Grade drug-related encephalitis	Permanently discontinue	
Myelitis	Any Grade myelitis	Delay dose	After workup for differential diagnosis, (i.e. infection, tumor-related), if myelitis is not drug related, then dosing may resume when AE resolves
	Any Grade drug-related myelitis	Permanently discontinue	
Neurological (other than GBS, MG, encephalitis, or myelitis)	Grade 2	Delay dose	Dosing may resume when AE resolves to baseline
	Grade 3 or 4	Permanently discontinue	

Table 7.4.1-1: Adverse Event Criteria for Delay, Resume, and Discontinue of Treatment

Drug-Related Adverse Event (AE) per CTCAE V5	Severity	Action Taken	Clarifications, Exceptions, and Resume Criteria
**Cardiovascular**			
Cardiac troponin T or I increased	Asymptomatic	Delay dose	Troponin elevations (including asymptomatic elevations) will require a dose delay and a confirmatory repeat evaluation within 24 hours. Cardiology consultation and cardiac evaluation will be required during the first instance of troponin elevation while on study intervention. Cardiology consultation and cardiac evaluation for subsequent troponin elevations will be the purview of the investigator. If troponin elevation is not confirmed within 24 hours in an asymptomatic participant, the dose delay may not be required, provided that the cardiac evaluation is completed and based on investigator's judgment to proceed with treatment. Refer to Section 9.4.5 for details.<br>Otherwise, if troponin elevation is confirmed, dosing may only resume when AE resolves to baseline.
**Myocarditis**			
Myocarditis	Symptoms induced from mild to moderate activity or exertion	Delay dose	Dosing may resume after myocarditis has resolved
	Severe or life threatening, with symptoms at rest or with minimal activity or exertion, and/or where intervention indicated.	Permanently discontinue	
**Other Clinical AE**			
Pancreatitis:<br><br>Amylase or Lipase increased	Grade 3 with symptoms	Delay dose	Note: Grade 3 increased amylase or lipase without signs or symptoms of pancreatitis does not require dose delay.<br>Dosing may resume when patient becomes asymptomatic.
	Grade 4	Permanently discontinue	
Uveitis	Grade 2 uveitis	Delay dose	Dosing may resume if uveitis responds to topical therapy (eye drops) and after uveitis resolves to Grade ≤ 1 or baseline. If patient requires oral steroids for uveitis, then permanently discontinue study drug.
	Grade 3 or 4 uveitis	Permanently discontinue	

Approved v4.0 930169531 4.0

Table 7.4.1-1: Adverse Event Criteria for Delay, Resume, and Discontinue of Treatment

Drug-Related Adverse Event (AE) per CTCAE V5	Severity	Action Taken	Clarifications, Exceptions, and Resume Criteria
Other Drug-Related AE **(not listed above)**	Grade 2 non-skin AE, except fatigue	Delay dose	Dosing may resume when AE resolves to Grade ≤ 1 or baseline value.
	Grade 3 AE - First occurrence lasting ≤ 7 days	Delay dose	Dosing may resume when AE resolves to Grade ≤ 1 or baseline value.
	Grade 3 AE- First occurrence lasting > 7 days	Permanently discontinue	
	Recurrence of Grade 3 AE of any duration	Permanently discontinue	
	Grade 4 or Life-threatening adverse reaction	Permanently discontinue	
**Other Lab abnormalities**			
Other Drug-Related lab abnormality (not listed above)	Grade 3	Delay dose	Exceptions:<br>No delay required for: Grade 3 lymphopenia<br><br>Permanent Discontinuation for: Grade 3 thrombocytopenia > 7 days or associated with bleeding.
	Grade 4	Permanently discontinue	Exceptions: The following events do not require discontinuation of study drug:<br>• Grade 4 neutropenia ≤ 7 days<br>• Grade 4 lymphopenia or leukopenia<br>• Grade 4 isolated electrolyte imbalances/abnormalities that are not associated with clinical sequelae and are responding to supplementation/appropriate management within 72 hours of their onset
**Infusion Reactions (manifested by fever, chills, rigors, headache, rash, pruritus, arthralgia, hypotension, hypertension, bronchospasm, or other allergic-like reactions.)**			
Hypersensitivity reaction or infusion reaction	Grade 3 or 4	Permanently discontinue	Refer to Section 7.4.4 on Treatment of Infusion-Related Reactions

Abbreviations: AE, adverse event; ALT, alanine aminotransferase; AST, aspartate aminotransferase; BMS, Bristol Myers Squibb; CTCAE, Common Terminology Criteria for Adverse Events; DRESS, drug reaction with eosinophilia and systemic symptoms; GBS, Guillain Barre syndrome; MG, myasthenia gravis; SJS, Stevens-Johnson syndrome; T.bili, total bilirubin; TEN, toxic epidermal necrolysis; ULN, upper limit of normal.

Approved v4.0 930169531 4.0

7.4.2 Criteria to Resume Treatment

Participants may resume treatment with study intervention if they have completed AE management (ie, corticosteroid taper) or are on ≤ 10 mg prednisone or equivalent, and meet the requirements per Table 7.4.1-1.

Prior to re-initiating treatment in a participant with a dosing delay lasting > 10 weeks (excluding delays due to steroid taper to manage drug-related AEs), the Medical Monitor/designee must be consulted in a documented discussion. Continue efficacy assessments per protocol (see Section 2) even if dosing is delayed. Continue periodic study visits to assess safety and laboratory studies every 4 weeks or more frequently if clinically indicated during such dosing delays.

Participants with SARS-CoV-2 infection (either confirmed or suspected) may resume treatment after all of the following:

- At least 10 days (4 weeks for severe/critical illness) have passed since symptoms first appeared or positive test result (e.g. RT-PCR or viral antigen)
- Resolution of acute symptoms (including at least 24 hours has passed since last fever without fever reducing medications)
- Evaluation by the Investigator with confirmation that there are no sequelae that would place the participant at a higher risk of receiving investigational treatment

For suspected cases, treatment may also resume if SARS-CoV-2 infection is ruled-out and other criteria to resume treatment are met.

7.4.3 Management of Algorithms for Immuno-Oncology Agents

Immuno-oncology (IO) agents are associated with AEs that can differ in severity and duration from AEs caused by other therapeutic classes. Relatlimab and nivolumab are considered IO agents, and the management algorithms in Appendix 5 provide guidance on assessing and managing the following groups of AEs:

- Gastrointestinal
- Renal
- Pulmonary
- Hepatic
- Endocrinopathy
- Skin
- Neurological
- Myocarditis

7.4.4 Treatment of Infusion-Related Reactions

Since relatlimab and nivolumab contain only human immunoglobulin protein sequences, it is unlikely to be immunogenic and induce infusion or hypersensitivity reactions. However, if such a

Approved v4.0 930169531 4.0

reaction were to occur, it might manifest with fever, chills, rigors, headache, rash, pruritus, arthralgias, hypotension, hypertension, bronchospasm, or other allergic-like reactions. Report all Grade 3 or 4 infusion reactions within 24 hours as an SAE if it meets the criteria.

Treatment recommendations are provided below based on CTCAE v5 grading definitions and may be modified based on local treatment standards and guidelines, as appropriate:

For Grade 1 symptoms (mild reaction; infusion interruption not indicated; intervention not indicated):

- Remain at bedside and monitor participant until recovery from symptoms. The following prophylactic premedications are recommended for future infusions: diphenhydramine 50 mg (or equivalent) and/or acetaminophen/paracetamol 325 to 1000 mg at least 30 minutes before additional study treatment administrations.

For Grade 2 symptoms: (Therapy or infusion interruption indicated but responds promptly to symptomatic treatment [e.g., antihistamines, NSAIDS, narcotics, IV fluid]; prophylactic medications indicated for ≤ 24 hours):

- Stop the study drug infusion, begin an IV infusion of normal saline, and treat the participant with diphenhydramine 50 mg intravenous (or equivalent) and/or acetaminophen/paracetamol 325 to 1000 mg; remain at bedside and monitor participant until resolution of symptoms. Corticosteroid and/or bronchodilator therapy may also be administered as appropriate. If the infusion is interrupted, then restart the infusion at 50% of the original infusion rate when symptoms resolve; if no further complications ensue after 30 minutes, the rate may be increased to 100% of the original infusion rate. Monitor participant closely. If symptoms recur, then no further study medication will be administered at that visit.
- For future infusions, the following prophylactic premedications are recommended: diphenhydramine 50 mg (or equivalent) and/or acetaminophen/paracetamol 325 to 1000 mg should be administered at least 30 minutes before study treatment infusions. If necessary, corticosteroids (up to 25 mg of hydrocortisone or equivalent) may be used.

For Grade 3 or 4 symptoms: (severe reaction, Grade 3: prolonged [ie, not rapidly responsive to symptomatic medication and/or brief interruption of infusion]; recurrence of symptoms following initial improvement; hospitalization indicated for other clinical sequelae. Grade 4: Life threatening consequences; urgent intervention indicated):

- Immediately discontinue infusion of study drug. Begin an IV infusion of normal saline and treat the participant as follows: Recommend bronchodilators, epinephrine 0.2 to 1 mg of a 1:1000 solution for subcutaneous administration or 0.1 to 0.25 mg of a 1:10,000 solution injected slowly for intravenous administration, and/or diphenhydramine 50 mg intravenous with methylprednisolone 100 mg intravenous (or equivalent), as needed. Monitor participant until the Investigator judges that the symptoms will not recur. Study drug will be permanently

Approved v4.0 930169531 4.0

discontinued. Follow institutional guidelines for the treatment of anaphylaxis. Remain at bedside and monitor participant until recovery of the symptoms.

In case of late-occurring hypersensitivity symptoms (eg, appearance of a localized or generalized pruritus within 1 week after treatment), symptomatic treatment may be given (eg, oral antihistamine or corticosteroids).

7.5 Preparation/Handling/Storage/Accountability

The IP/AxMP must be stored in a secure area according to local regulations. It is the responsibility of the investigator, or designee where permitted, to ensure that IP/AxMP is only dispensed to study participants. The IP/AxMP must be dispensed only from official study sites by authorized personnel according to local regulations.

The product storage manager should ensure that the study intervention is stored in accordance with the environmental conditions (temperature, light, and humidity) as determined by BMS. If concerns regarding the quality or appearance of the study intervention arise, the study intervention should not be dispensed, and BMS should be contacted immediately.

Study intervention not supplied by BMS will be stored in accordance with the package insert.

IP/AxMP documentation (whether supplied by BMS or not) must be maintained that includes all processes required to ensure the drug is accurately administered. This includes documentation of drug storage, administration and, as applicable, storage temperatures, reconstitution, and use of required processes (eg, required diluents, administration sets).

- The investigator or designee must confirm appropriate temperature conditions have been maintained during transit for all study intervention received and any discrepancies are reported and resolved before use of the study intervention.
- The investigator, institution, or the head of the medical institution (where applicable) is responsible for study intervention accountability, reconciliation, and record maintenance (ie, receipt, reconciliation, and final disposition records).
- Further guidance and information for the final disposition of unused study interventions are provided in Appendix 2 and the Pharmacy Manual.

7.5.1 Retained Samples for Bioavailability/Bioequivalence/Biocomparability

Not Applicable.

7.6 Treatment Compliance

Treatment compliance will be monitored by drug accountability as well as the participant's medical record and electronic case report form (eCRF). This will be source data reviewed through regularly scheduled monitoring visits.

Approved v4.0 930169531 4.0

7.7 Concomitant Therapy

7.7.1 Prohibited and/or Restricted Treatments

7.7.1.1 Prohibited Treatments

The following medications and treatments are prohibited during the study (unless utilized to treat a drug-related AE):

- Any complementary medications (eg, herbal supplements or traditional Chinese medicines) intended to treat the disease under study. Such medications are permitted if they are used as supportive care.
- Immunosuppressive agents.
- Immunosuppressive doses of systemic corticosteroids. Participants are permitted the use of topical, ocular, intra-articular, intranasal, and inhalational corticosteroids (with minimal systemic absorption). A brief (less than 3 weeks) course of corticosteroids for prophylaxis (eg, contrast dye allergy) or for treatment of non-autoimmune conditions (eg, delayed-type hypersensitivity reaction caused by a contact allergen) is permitted. Adrenal replacement steroid doses > 10 mg daily prednisone are permitted.
- Any concurrent systemic anti-neoplastic therapy (ie, chemotherapy, hormonal therapy, immunotherapy, extensive, or standard or investigational agents for treatment of malignancy).
- Any radiation therapy.
- Any melanoma-directed surgery.
- Any live / attenuated vaccine (eg, varicella, zoster, yellow fever, rotavirus, oral polio and measles, mumps, rubella [MMR]) during treatment and until 135 days post last dose.

7.7.2 Other Restrictions and Precautions

Participants with a condition requiring long term systemic treatment with either corticosteroids (> 10 mg daily prednisone equivalent) or other immunosuppressive medications. Inhaled or topical steroids, and adrenal replacement steroid doses > 10 mg daily prednisone equivalent, are permitted in the absence of active autoimmune disease.

Participants are prohibited from joining another interventional clinical trial if they are still on study treatment. Participation in observation only studies is allowed at any time.

7.7.2.1 SARS-CoV-2 Vaccination Guidelines

Non-live COVID-19 vaccination is considered a simple concomitant medication within the study. However, the efficacy and safety of non-live vaccines (including non-live COVID-19 vaccines) in participants receiving study drug is unknown.

- For COVID-19 vaccines requiring more than 1 dose, the full series (e.g., both doses of a 2-dose series) should be completed prior to enrollment when feasible, and when a delay in enrollment would not put the study participant at risk.
- Vaccines that are capable of transmitting infectious SARS-CoV-2 or other viruses, referred to as “live vaccines” in the COVID-19 Testing, Treatment, and Prevention Guidance for

Approved v4.0 930169531 4.0

Protocols, should generally not be administered to a study participant during the study, including the treatment and safety follow-up period.

- COVID-19 vaccines that are NOT live are acceptable and should be handled in the same manner as other vaccines. The vaccine may be administered during the study, including during IP treatment, and after the last administration of IP if recommended by the treating physician/investigator.

7.7.2.2 Imaging Restriction and Precautions

It is the local imaging facility's responsibility to determine, based on participant attributes (eg, allergy history, diabetic history, and renal status), the appropriate imaging modality and contrast regimen per imaging study. Imaging contraindications and contrast risks are to be considered in this assessment. Participants with renal insufficiency are to be assessed as to whether or not they should receive contrast and if so, which contrast agent and dose is appropriate based on local standard of care.

Specific to MRI, participants with severe renal insufficiency (ie, estimated glomerular filtration rate (eGFR) < 30 mL/min/1.73 m^2) are at increased risk of nephrogenic systemic fibrosis, therefore MRI contrast is contraindicated. Participants may be excluded from MRI if they have tattoos, metallic implants, pacemakers, etc., following local standard of care.

Gentle hydration before and after IV contrast should follow local standard of care. The ultimate decision to perform MRI in an individual participant in this study rests with the site radiologist, the Investigator, and standards set by the local Ethics Committee.

7.8 Continued Access to Study Intervention After the End of the Study

At the end of the study, BMS will not continue to provide BMS-supplied study intervention to participants/investigators unless BMS chooses to extend the study. The investigator should ensure that the participant receives appropriate standard of care to treat the condition under study.

BMS reserves the right to terminate access to BMS-supplied study intervention if any of the following occur: a) the study is terminated due to safety concerns; b) the development of nivo + rela FDC is terminated for other reasons, including, but not limited to, lack of efficacy and/or not meeting the study objectives; c) the participant can obtain medication from a government-sponsored or other health program. In all cases, BMS will follow local regulations.

8 DISCONTINUATION CRITERIA

8.1 Discontinuation From Study Treatment

Participants MUST discontinue IP (and Non-IP/AxMP at the discretion of the investigator) for any of the following reasons:

- Participant's request to stop study intervention. Participants who request to discontinue study intervention will remain in the study and must continue to be followed for protocol-specified follow-up procedures. The only exception to this is when a participant specifically withdraws consent for any further contact with him/her or persons previously authorized by the participant to provide this information

Approved v4.0 930169531 4.0

- Any clinical AE, laboratory abnormality, or intercurrent illness which, in the opinion of the investigator, indicates that continued participation in the study is not in the best interest of the participant
- Termination of the study by BMS
- Loss of ability to freely provide consent through imprisonment or involuntarily incarceration for treatment of either a psychiatric or physical (eg, infectious disease) illness. (Note: Under specific circumstances and only in countries where local regulations permit, a participant who has been imprisoned may be permitted to continue as a participant. Strict conditions apply, and BMS approval is required.)
- Disease recurrence (local, regional, or distant) or new primary melanoma but excluding malignant melanoma in situ.
- Additional protocol-specified reasons for discontinuation (Section 8.1.1)
- Pregnancy (refer to Section 9.2.5)
- Significant noncompliance with protocol (eg, procedures, assessments, medications, etc.). The investigator should discuss such issues with the Medical Monitor/designee and be documented.

Refer to the Schedule of Activities for data to be collected at the time of treatment discontinuation and follow-up and for any further evaluations that can be completed.

All participants who discontinue study intervention should comply with protocol-specified follow-up procedures as outlined in Section 2. The only exception to this requirement is when a participant withdraws consent for all study procedures, including post-treatment study follow-up, or loses the ability to consent freely (eg, is imprisoned or involuntarily incarcerated for the treatment of either a psychiatric or physical illness).

If study intervention is discontinued prior to the participant's completion of the study, the reason for the discontinuation must be documented in the participant's medical records per local regulatory requirements in each region/country and entered on the appropriate eCRF page.

8.1.1 Dose Discontinuation

Study treatment must be permanently discontinued per criteria in Table 7.4.1-1 in Section 7.4.

Discontinue study treatment for any AE, laboratory abnormality, or intercurrent illness which in the judgment of the Investigator, presents a substantial clinical risk to the participant with continued dosing.

Any event that leads to delay in dosing lasting > 10 weeks from the previous dose requires discontinuation of study drug, with the following exceptions:

- Dosing delays to allow for prolonged steroid tapers to manage drug-related AE are allowed.
- Dosing delays lasting > 10 weeks from the previous dose that occur for non-drug-related reasons may be allowed if approved by the Medical Monitor/ designee.

Note: dosing delays that are approved by the Medical Monitor/designee must be documented.

Approved v4.0 930169531 4.0

8.1.2 Post-study Intervention Study Follow-up

In this study, RFS is a key endpoint of the study. Post-study follow-up is of critical importance and is essential to preserving participant safety and the integrity of the study. Participants who discontinue study intervention must continue to be followed (in this study or a rollover study) for collection of outcome and/or survival follow-up data as required and in line with Section 5.3 until death or the conclusion of the study. Stage III patients must continue to have imaging until distant recurrence. For mucosal melanoma participants, assessment should continue until investigator assessed local, regional, or distant recurrence for Stage M1, and until distant recurrence for M0 participants.

BMS may request that survival data be collected on all randomized participants outside of the protocol defined window as defined in Section 2. At the time of this request, each participant will be contacted to determine their survival status unless the participant has withdrawn consent for all contacts or is lost to follow-up.

8.2 Discontinuation From the Study

Participants who request to discontinue study intervention will remain in the study and must continue to be followed for protocol-specified follow-up procedures. The only exception to this is when a participant specifically withdraws consent for any further contact with him/her or persons previously authorized by participant to provide this information.

- Participants should notify the investigator of the decision to withdraw consent from future follow-up.
- The withdrawal of consent should be explained in detail in the medical records by the investigator, as to whether the withdrawal is from further treatment with study intervention only or also from study procedures and/or post-treatment study follow-up, and entered on the appropriate eCRF page.
- In the event that vital status (whether the participant is alive or dead) is being measured, publicly available information should be used to determine vital status only as appropriately directed in accordance with local law.
- If the participant withdraws consent for disclosure of future information, the Sponsor may retain and continue to use any data collected before such a withdrawal of consent.

8.2.1 Individual Discontinuation Criteria

- A participant may withdraw completely from the study at any time at his/her own request, or may be withdrawn at any time at the discretion of the investigator for safety, behavioral, compliance, or administrative reasons. This is expected to be uncommon. Stopping study intervention is not considered withdrawal from the study.
- Site should document if subject is unwilling to attend future visits, accept phone calls, allow contact with their personal physician, or allow review of medical records for health status information.

Approved v4.0 930169531 4.0

- At the time of discontinuing from the study, an immediate follow-up visit may be needed. See the Schedule of Activities (Section 2) for data to be collected at the time of study discontinuation and follow-up and for any further evaluations that need to be completed.
- The participant will be permanently discontinued both from the study intervention and from the study at that time.
- If the participant withdraws consent for disclosure of future information, the Sponsor may retain and continue to use any data collected before such a withdrawal of consent.

8.3 Lost to Follow-up

The following actions must be taken if a participant fails to return to the clinic for a required study visit:

- All reasonable efforts must be made to locate participants to determine and report their ongoing status. This includes follow-up with persons authorized by the participant.
- Lost to follow-up is defined by the inability to reach the participant after a minimum of **three (3)** documented phone calls, faxes, or emails, as well as lack of response by participant to one (1) registered mail letter. All attempts should be documented in the participant's medical records.
- If it is determined that the participant has died, the site will use permissible local methods to obtain date and cause of death.
- If the investigator's use of third-party representative to assist in the follow-up portion of the study has been included in the participant's informed consent, then the investigator may use a Sponsor-retained third-party representative to assist site staff with obtaining the participant's contact information or other public vital status data necessary to complete the follow-up portion of the study.
- The site staff and representative will consult publicly available sources, such as public health registries and databases, in order to obtain updated contact information.
- If, after all attempts, the participant remains lost to follow-up, then the last known alive date as determined by the investigator should be reported and documented in the participant's medical records.

9 STUDY ASSESSMENTS AND PROCEDURES

Study procedures and timing are summarized in the Schedule of Activities.

Protocol waivers or exemptions are not allowed.

All immediate safety concerns must be discussed with the Sponsor immediately upon occurrence or awareness to determine if the participant should continue or discontinue treatment.

Adherence to the study design requirements, including those specified in the Schedule of Activities, is essential and required for study conduct.

All screening evaluations must be completed and reviewed to confirm that potential participants meet all eligibility criteria before randomization. The investigator will maintain a screening log to

Approved v4.0 930169531 4.0

record details of all participants screened and to confirm eligibility or record reasons for screening failure, as applicable.

Procedures conducted as part of the participant's routine clinical management (e.g., blood count) and obtained before signing of informed consent may be utilized for screening or baseline purposes provided the procedure meets the protocol-defined criteria and has been performed within the timeframe defined in the Schedule of Activities.

Perform additional measures, including non-study required laboratory tests, should be performed as clinically indicated or to comply with local regulations. Laboratory toxicities (eg, suspected drug induced liver enzyme evaluations) will be monitored during the follow-up phase via on site/local labs until all study drug related toxicities resolve, return to baseline, or are deemed irreversible.

Evaluate participant immediately to rule out cardiac or pulmonary toxicity if participant shows cardiac or pulmonary-related signs (hypoxia, abnormal heart rate or changes from baseline) or symptoms (eg, dyspnea, cough, chest pain, fatigue, palpitations).

Some of the assessments referred to in this section may not be captured as data in the eCRF. They are intended to be used as safety monitoring by the treating physician. Additional testing or assessments may be performed as clinically necessary or where required by institutional or local regulations.

The maximum amount of blood collected from each participant over the duration of the study, including any extra assessments that may be required, will be at approximately 775 mL. However, the volume of blood drawn for local safety assessments, done before every dose, and in follow-up, will depend on local institutional practices and will vary from institution to institution.

9.1 Efficacy Assessments

9.1.1 Efficacy Assessment for the Study

Study evaluations will take place in accordance with Section 2, Schedule of Activities. Surveillance for recurrence will be performed until investigator assessed local, regional, or distant recurrence (whichever comes first) for Stage IV participants and until distant recurrence for Stage III participants. Follow-up scans should be discontinued only after distant recurrence for Stage III participants regardless of the start of subsequent systemic therapy. For Stage IV participants, follow-up scans should be discontinued after unequivocal melanoma recurrence.

Contrast-enhanced CT of the chest, abdomen, pelvis, and all suspected sites of disease must be performed every 12 weeks (± 7 days) from randomization for the first 2 years, and every 26 weeks (± 14 days) beyond the Week 108 imaging time point thereafter until investigator assessed local, regional, or distant recurrence (whichever comes first) for Stage IV participants and until distant recurrence for Stage III participants. For mucosal melanoma participants, assessment should continue until investigator assessed local, regional, or distant recurrence for Stage M1, and until distant recurrence for M0 participants.

Approved v4.0 930169531 4.0

Imaging should be obtained anytime there is suspected disease. In cases of suspected lesions of the extremities, contrast-enhanced MRI may be substituted for contrast-enhanced CT. Please refer to Appendix 9 for Germany-specific imaging language.

Participants with a history of brain metastasis or symptoms should have a surveillance MRI (without and with contrast) approximately every 12 weeks ± 7 days from randomization for the first 2 years and subsequently every 26 weeks (± 14 days) beyond the Week 108 imaging time point thereafter until investigator assessed local, regional, or distant recurrence (whichever comes first) for Stage IV participants or sooner if clinically indicated. Participants without history of brain metastases should have MRI if clinically indicated.

9.1.2 Imaging Assessment for the Study

Images will be submitted to a central imaging vendor and may undergo blinded independent central review (BICR) at any time during the study. Prior to scanning the first participant, sites should be qualified and understand the image acquisition guidelines and submission process as outlined in the Imaging Manual provided by the central imaging vendor.

Screening and on-study images should be acquired as outlined in Section 2, Schedule of Activities. Tumor assessments at other time points may be performed if clinically indicated and should be submitted to the central imaging vendor as soon as possible. All imaging that may demonstrate disease recurrence (including scans performed at unscheduled time points and/or at an outside institution) should be collected and submitted to the imaging vendor.

Assessments for recurrence should continue on the protocol defined imaging schedule regardless if dosing is delayed or discontinued. Assessment should continue until investigator assessed local, regional, or distant recurrence (whichever comes first) for Stage IV participants and until distant recurrence for Stage III participants. For mucosal melanoma participants, assessment should continue until investigator assessed local, regional, or distant recurrence for Stage M1, and until distant recurrence for M0 participants.

The same method of assessment used at Screening should be used for on-study time points. CT images should be acquired with slice thickness of 5 mm or less with no intervening gap (contiguous). Every attempt should be made to image each participant using an identical acquisition protocol on the same scanner for all imaging time points.

- If a participant has a contraindication for CT intravenous contrast, then a non-contrast CT of the chest and a contrast-enhanced MRI of the abdomen, pelvis, and suspected sites of disease should be obtained.
- If a participant has a contraindication for both MRI and CT intravenous contrasts, then a non-contrast CT of the chest and a non-contrast MRI of the abdomen, pelvis, and other suspected sites of disease should be obtained.
- If a participant has a contraindication for MRI (eg, incompatible pacemaker) in addition to contraindication to CT intravenous contrast, then a non-contrast CT of the chest, abdomen, pelvis, and suspected sites of disease is acceptable.

Approved v4.0 930169531 4.0

MRI of brain (without and with contrast) must be acquired as outlined in Section 2 (Schedule of Activities). CT of the brain (without and with contrast) can be performed if MRI is contraindicated.

Other imaging may be collected per local standards, as clinically indicated. See Section 7.7.2.2.

Use of CT component of a PET-CT scanner: Combined modality scanning such as with positron emission tomography-computed tomography (PET-CT) is increasingly used in clinical care, and is a modality/technology that is in rapid evolution; therefore, the recommendations outlined here may change rather quickly with time. At present, low dose or attenuation correction CT portions of a combined PET-CT are of limited use in anatomically-based efficacy assessments and it is therefore suggested that they should not be substituted for dedicated diagnostic contrast enhanced CT scans for anatomically-based measurements. However, if a site can document that the CT performed as part of a PET-CT is of identical diagnostic quality to a diagnostic CT (with intravenous and oral contrast), then the CT portion of the PET-CT can be used for measurements. Note, however, that the PET portion of the CT introduces additional data which may bias an investigator if it is not routinely or serially performed.

9.1.3 Investigator Assessment of Baseline Disease Status

Participant eligibility (disease-free status) must be confirmed by investigator prior to randomization. Baseline disease assessments should be performed within 35 days prior to randomization, including contrast-enhanced CT of the chest, abdomen, pelvis, and all suspected sites of disease. Baseline MRI of the brain (with and without contrast) is required for ALL participants during screening to rule out brain metastases, within 35 days prior to randomization. CT of the brain (without and with contrast) can be performed if MRI is contraindicated. Please refer to Appendix 9 for Germany-specific imaging language.

All participants must have disease-free status defined as no clinical or radiographic evidence of recurrence of disease documented by a complete physical examination within 14 days prior to randomization and imaging studies within 35 days prior to randomization. Participants with suspect lesions (such as equivocal lymph nodes ≥ 10 mm and < 15 mm in a short axis) may be eligible if suspicion of disease recurrence is ruled out by histology/cytology (all subjects must have disease-free status prior to randomization). If risk of biopsy is too high or biopsy is not feasible, 2 sequential CT or MRI scans (the second scan should occur at least 4 weeks after the initial scan) should be available showing no signs of progressive and measurable disease, or PET/CT demonstrating no fluorodeoxyglucose (FDG) uptake. Lymph nodes ≥ 15 mm in a short axis are defined as pathological.

9.1.4 Investigator Assessment of Recurrence

The same method of assessment used at Screening should be used for on-study time points. Post-baseline assessments will be performed at the time points described in Table 2-2 and Table 2-3 until local, regional, or distant recurrence (whichever comes first) for Stage IV participants and until distant recurrence for Stage III participants confirmed by investigator, death, or withdrawal from the study. For mucosal melanoma participants, assessment should continue until investigator assessed local, regional, or distant recurrence for Stage M1, and until distant recurrence for M0 participants.

Approved v4.0 930169531 4.0

Tumor assessments for ongoing study treatment decisions will be completed by the investigator. Additional imaging of potential disease sites should be performed whenever disease recurrence or occurrence of a secondary malignancy is suspected.

Cytology and/or histology are mandatory to confirm recurrence in solitary or in doubtful/suspect lesions, cutaneous, subcutaneous, or lymph node lesions. Histological or cytological evidence of recurrence should be attempted in all cases, except for brain metastases, when safe and clinically feasible. Tumor markers or auto-antibodies alone cannot be used to assess recurrence.

Although not considered recurrence, the incident of MMIS will be captured in the eCRF.

9.1.4.1 Unequivocal Recurrence

If recurrence is unequivocal (eg, multiple measurable lesions), confirmation with histology/cytology should be attempted if medically feasible. Appearance of multiple new lesions in the same time point generally constitutes unequivocal recurrence, even though they may be from different organs (eg, 1 liver lesion, 1 lung lesion, and 1 enlarged lymph node).

9.1.4.2 Equivocal Recurrence/Suspect Lesions

If recurrence is equivocal (presence of a suspect lesion such as lymph node only, solitary lesion), confirmation with histology/cytology must be attempted. If risk of biopsy is too high or biopsy not feasible, 2 sequential CT or MRI scans (the second scan should occur at least 4 weeks after the initial scan) should be available showing no signs of progressive and measurable disease, or PET/CT demonstrating no FDG uptake. The date of initial scan showing recurrence will count as recurrence date and not date of follow up exam.

Equivocal recurrence is upgraded to unequivocal recurrence (except in cases of CNS recurrence) by 1 or more of the following:

- A subsequent scan not earlier than 4 weeks, but no later than 12 weeks, from the time when recurrence was first suspected demonstrates that the lesion size is $\geq$ 5 mm over the size previously recorded, or the radiographic appearance of the lesion has become consistent with tumor recurrence. If this occurs, the date of recurrence will be the date when the lesion was first suspected.
- Positive histology/cytology

Approved v4.0 930169531 4.0

Table 9.1.4.2-1: Criteria for Diagnosing Recurrence

Anatomic Sites	Criteria
Non-Nodal Soft Tissue	Equivocal recurrence:<br>• Solitary lesion measuring ≤ 10 mm in LD or with radiographic (CT or MRI) appearance equivocal for tumor recurrence.<br>Unequivocal recurrence:<br>• One or more new lesions > 10 mm in LD with radiographic (CT or MRI) appearance consistent with tumor recurrence.<br>• Positive histology/cytology
Bone Lesions	Equivocal recurrence:<br>• Solitary lesion.<br>• Lesions identified on radionuclide bone scan. Findings on radionuclide bone scan must be confirmed by CT, MRI, or plain films in order to be upgraded to unequivocal.<br>Unequivocal recurrence:<br>• Two or more new lesions consistent with tumor recurrence.<br>• Positive histology/cytology
Lymph Nodes[a]	Equivocal recurrence:<br>• Lymph nodes measuring 10 – 14 mm short-axis diameter with radiographic (CT or MRI) appearance consistent with recurrence. (Normal lymph nodes are defined as < 10 mm in the short-axis diameter.)<br>• Lymph nodes ≥ 15 mm short-axis diameter without radiographic (CT or MRI) appearance consistent with recurrence.<br>Unequivocal recurrence:<br>• One or more previously normal or equivocal lymph nodes that enlarge to ≥ 15 mm short-axis diameter and with radiographic (CT or MRI) appearance consistent with recurrence.<br>• Positive histology/cytology
Fluid Collections<br>(eg, ascites, pleural/pericardial effusions)	Unequivocal recurrence:<br>• Positive pathology of malignant cells from fluid(s).<br>Presence of fluid alone, without pathological confirmation, does not constitute equivocal or unequivocal recurrence.
CNS	Unequivocal recurrence:<br>• Any new CNS lesion of any size on CT or MRI with a radiographic appearance consistent with tumor recurrence.

Abbreviations: CNS, central nervous system; CT, computed tomography; LD, longest diameter; MRI, magnetic resonance imaging.

[a] For lymph node with short axis 10-15 mm, consider biopsy when lymph node is progressively enlarged.

Clinically detected lesions

- The neoplastic nature of clinically detected lesions must be confirmed by cytology/histology.

Approved v4.0 930169531 4.0

- Deep subcutaneous lesions should be documented by ultrasound and histological/cytological evidence should be attempted. In absence of pathology report, lesion recurrence will be documented with a CT scan/MRI.
- Any recurrence identified by ultrasound must be recorded as a recurrence event even though ultrasound images are not submitted to the imaging vendor.

9.1.4.3 Definition of Recurrence

Recurrence is defined as the appearance of one or more new melanoma lesions (except MMIS), which can be local, regional, including in-transit and regional nodal recurrence, or distant in location from the primary resected site.

Local Recurrence:

Local recurrence is defined as tumor regrowth within 2 cm of the surgical incision following definitive excision of a primary melanoma with appropriate surgical margins. Lesions must be unequivocal as noted in Table 9.1.4.2-1.

In-Transit Metastases Recurrence:

Any skin or subcutaneous metastases more than 2 cm from the primary melanoma lesion, but not beyond the regional lymph node basin. Lesions must be unequivocal as noted in Table 9.1.4.2-1.

Regional Node Recurrence:

Regional node failure, usually at the periphery of the prior surgical procedure. Lesions must be unequivocal as noted in Table 9.1.4.2-1.

Distant Recurrence:

Any distant metastases with radiographic appearance consistent with tumor recurrence, or positive histology / cytology. Distant metastases include node relapses beyond the anatomical compartment of the primary melanoma basin, a nodal basin situated in a different anatomical compartment beyond the primary melanoma lesion, or in 2 nodal basins (even if contiguous; i.e. 2 pelvic nodal basins, 2 mediastinal nodal basins, etc.). Must be unequivocal as defined in Table 9.1.4.2-1.

New Primary Invasive Melanoma:

Melanoma determined by treating physician to be a new primary invasive melanoma.

9.1.4.4 Date of Recurrence

The first date when recurrence was observed is taken into account regardless of the method of assessment. Therefore recurrence will be declared for any lesion when:

- Only imaging was performed and recurrence confirmed
- Only pathology was done and malignancy confirmed (in solitary or in doubtful lesions, cutaneous, subcutaneous, or lymph node lesions).
- Both pathology (date of biopsy) and imaging were done and recurrence/malignancy confirmed. In this case, the date of whichever examination comes first is considered the date of recurrence.

Approved v4.0 930169531 4.0

- Pathology reports of biopsies confirming recurrence should be sent to the central laboratory vendor. The date of recurrence is the date of the biopsy which confirms the pathology and/or imaging confirms recurrence, not the date that the information was communicated to the participant.

9.1.5 Patient-Reported Outcomes

Health-related quality of life (HRQoL) will be assessed by European Organization for the Research and Treatment of Cancer Quality of Life Questionnaire (EORTC QLQ-C30), Functional Assessment of Chronic Illness Therapy-Item GP5 (FACIT-GP5), and EQ-5D-5L in participants who are ≥ 18 years of age at the time of informed consent as outlined in Section 2. Adolescent participants (≥ 12 and < 18 years of age) only complete the EQ-5D-5L. Adolescent participants will continue to complete only the EQ-5D-5L even if they become ≥ 18 years of age during treatment or follow-up.

If Health Outcomes Assessments are collected but the dose is subsequently delayed, a data change form should be submitted to move the original data entry to an unscheduled visit.

If the participant withdraws from the study prematurely, all attempts should be made to obtain EORTC QLQ-C30, FACIT-GP5, and EQ-5D-5L prior to withdrawal from the study in participants who are ≥ 18 years of age at the time of informed consent, or EQ-5D-5L for participants who are ≥ 12 and < 18 years of age at the time of informed consent. Reasons for missing patient-reported outcomes questionnaires should also be documented so that the appropriate imputation method can be employed to correct for missing data in the analysis.

The questionnaire will be completed by the participants before any clinical assessments are performed and treatments administered at any given visit. If participants refuse to complete all or any part of a questionnaire, this will be documented. Questionnaires should be completed in the language most familiar to each participant (if available), and participants should be given adequate time and space to complete the questionnaire. In order to preserve the integrity of trial data, no patient-reported data can be changed after the patient initially provides it.

EQ-5D-5L assessments in Long-term Follow Up (visits beyond Follow Up 2) may be completed by other modes of administration (eg, telephone) if deemed necessary by the study team. If exceptional circumstances preclude the continued administration of measures using planned modalities, then alternate administration methods may be required, after consultation with the Sponsor.

9.1.5.1 EORTC QLQ-C30

The EORTC QLQ-C30 will be used as a measure of HRQoL. The EORTC QLQ-C30 is composed of both multi-item scales and single item measures. These include 5 functional scales (physical, role, emotional, cognitive, and social), 3 symptom scales (fatigue, nausea/vomiting, and pain), a global health status/HRQoL scale, and 6 single items (dyspnea, insomnia, appetite loss, constipation, diarrhea, and financial difficulties). Each of the multi-item scales includes a different set of items; no item occurs in more than 1 scale.

Approved v4.0 930169531 4.0

The EORTC QLQ-C30 employs a week recall period for all items and a 4-point scale for the functional and symptom scales/items with response categories “Not at all”, “A little”, “Quite a bit” and “Very much”. The 2 items assessing global health status/HRQoL utilize a 7-point scale ranging from 1 (“Very Poor”) to 7 (“Excellent”).[45]

9.1.5.2 FACIT-GP5

Participants will be asked to complete a single item from the FACIT, Item GP5, that will be used to assess the extent of perceived bother due to symptomatic AEs. Evidence exists for the validity of this item and its usefulness as an overall summary measure of burden due to symptomatic treatment toxicities.[46]

9.1.5.3 EQ-5D-5L

The EQ-5D-5L is a standardized measure of health status developed by the EuroQol Group in order to provide a simple, generic measure of health for clinical and economic appraisal.[47,48] The EQ-5D-5L has 2 components: a descriptive system and a visual analogue scale (VAS). The EQ-5D-5L descriptive system comprises 5 dimensions: mobility, self-care, usual activities, pain/discomfort, and anxiety/depression. Each dimension has 5 levels including “no,” “slight,” “moderate,” “severe,” and “extreme” or “unable to.” A dimension for which there are no problems is said to be at level 1, while a dimension for which there are extreme problems is said to be at level 5. Accordingly, the vectors 11111 and 55555 represent the best health state and the worst health state, respectively, described by the EQ-5D-5L. Altogether, the instrument describes 5^5 = 3,125 health states. Empirically derived weights can be applied to an individual’s responses to the EQ-5D-5L descriptive system to generate a utility index measuring the value to society of his or her current health. In addition, the EQ-5D-5L VAS allows respondents to rate their own current health on a 101-point scale ranging from “best imaginable” to “worst imaginable” health. The EQ-5D-5L is available in more than 130 languages.

The instrument is scored as a single summary index using 1 of the available country-specific EQ-5D-5L value sets, or using an existing EQ-5D-3L value set to produce “crosswalk” values via a mapping algorithm.[49] The clinically meaningful thresholds for group-level and individual-level analyses will be pre-specified in the Statistical Analysis Plan based on the most recent recommendations published by that time.

A standardized script will be used to facilitate telephone administration of the EQ-5D-5L during Long-term Follow-up Visits.

9.2 Adverse Events

The definitions of an AE or SAE can be found in Appendix 3.

AEs will be reported by the participant (or, when appropriate, by a caregiver, a surrogate, or the participant’s LAR).

The investigator and any qualified designees are responsible for detecting, documenting, and reporting events that meet the definition of an AE or SAE and remain responsible for following up

Approved v4.0 930169531 4.0

on AEs that are serious, considered related to the study intervention or the study, or that caused the participant to discontinue before completing the study.

Use CTCAE v5 definitions and grading for safety reporting of all AE and SAEs on the case report form.

Immune-mediated adverse events are AEs consistent with an immune-mediated mechanism or immune-mediated component for which non-inflammatory etiologies (eg, infection or tumor progression) have been ruled out. IMAEs can include events with an alternate etiology which were exacerbated by the induction of autoimmunity. Information supporting the assessment will be collected on the participant's case report form.

Refer to Appendix 3 for SAE reporting.

9.2.1 Time Period and Frequency for Collecting AE and SAE Information

Collect all nonserious adverse events (not only those deemed to be treatment related) continuously during the treatment period and for a minimum of 135 days following discontinuation of study treatment. All AEs associated with SARS-CoV-2 infection must be collected from time of consent and during the treatment period. All SAEs must be collected from the time of signing the consent, including those thought to be associated with protocol-specified procedures, and within 135 days following discontinuation of dosing, except in cases where a study participant has started a new anti-neoplastic therapy. However, any SAE occurring after the start of a new treatment that is suspected to be related to study intervention by the investigator will be reported.

For participants randomized to treatment and never treated with study drug, collect SAEs for 30 days from the date of randomization.

All SAEs, and all AEs (SAEs and non-serious AEs) associated with confirmed or suspected SARS-CoV-2 infection must be collected from the date of the participant's written consent until 135 days following discontinuation of dosing. Collect all nonserious adverse events, not only those deemed to be treatment-related, (with the exception of non-serious AEs related to SARS-CoV-2 infection) continuously during the treatment period and for a minimum of 135 days following discontinuation of study treatment.

The investigator must report any SAE that occurs after these time periods and that is believed to be related to study intervention or protocol-specified procedure (eg, a follow-up skin biopsy).

- Medical occurrences that begin before the start of study intervention but after obtaining informed consent will be recorded on the appropriate section of the eCRF module.
- All SAEs will be recorded and reported to Sponsor or designee within 24 hours, as indicated in Appendix 3.
- The investigator will submit any updated SAE data to the sponsor or designee within 24 hours of updated information being available.

Investigators are not obligated to actively seek AEs or SAEs in former study participants. However, if the investigator learns of any SAE, including a death, at any time after a participant

Approved v4.0 930169531 4.0

has been discharged from the study, and he/she considers the event reasonably related to the study intervention or study participation, the investigator must promptly notify the Sponsor.

The method of evaluating and assessing causality of AEs and SAEs and the procedures for completing and reporting/transmitting SAE reports are provided in Appendix 3.

9.2.2 Method of Detecting AEs and SAEs

AEs can be spontaneously reported or elicited during open-ended questioning, examination, or evaluation of a participant. Care should be taken not to introduce bias when collecting AEs and/or SAEs. Inquiry about specific AEs should be guided by clinical judgement in the context of known AEs, when appropriate for the program or protocol.

All nonserious adverse events (not only those deemed to be treatment-related) should be collected continuously during the treatment period and for a minimum of 135 days following discontinuation of study treatment.

Every adverse event must be assessed by the investigator with regard to whether it is considered immune-mediated. For events which are potentially immune-mediated, additional information will be collected on the participant's case report form.

9.2.3 Follow-up of AEs and SAEs

- Nonserious AEs should be followed to resolution or stabilization, or reported as SAEs if they become serious (see Appendix 3).
- Follow-up is also required for nonserious AEs that cause interruption or discontinuation of study intervention and for those present at the end of study intervention as appropriate.
- All identified nonserious AEs must be recorded and described on the nonserious AE page of the CRF (paper or electronic). Completion of supplemental CRFs may be requested for AEs and/or laboratory test result abnormalities that are reported/identified during the study.

All SAEs will be followed until resolution, until the condition stabilizes, until the event is otherwise explained, or until the participant is lost to follow-up (as defined in Section 8.3).

Further information on follow-up procedures is given in Appendix 3.

9.2.4 Regulatory Reporting Requirements for SAEs

Prompt notification by the investigator to the Sponsor of SAEs is essential so that legal obligations and ethical responsibilities toward the safety of participants and the safety of a product under clinical investigation are met.

An investigator who receives an investigator safety report describing SAEs or other specific safety information (eg, summary or listing of SAEs) from the Sponsor will file it along with the Investigator's Brochure and will notify the IRB/IEC, if appropriate according to local requirements.

The Sponsor or designee must report AEs to regulatory authorities and ethics committees according to local applicable laws and regulations. A SUSAR (suspected, unexpected serious

Approved v4.0 930169531 4.0

adverse reaction) is a subset of SAEs and must be reported to the appropriate regulatory authorities and investigators following local and global guidelines and requirements.

9.2.5 Pregnancy

If, following initiation of the study intervention, it is subsequently discovered that a participant is pregnant or may have been pregnant at the time of study exposure, including during at least 5 months after study product administration, the investigator must immediately notify the BMS Medical Monitor/designee of this event and complete and forward a Pregnancy Surveillance Form to the BMS designee within 24 hours of awareness of the event and in accordance with SAE reporting procedures described in Appendix 3.

Follow-up information regarding the course of the pregnancy, including perinatal and neonatal outcome and, where applicable, offspring information, must be reported on the Pregnancy Surveillance Form. Protocol-required procedures for study discontinuation and follow-up must be performed on the participant.

For WOCBP who are partners of male participants in the study, pregnancy reporting is not required.

9.2.6 Laboratory Test Result Abnormalities

The following laboratory test result abnormalities should be captured on the nonserious AE CRF page or SAE eCRF, as appropriate. Paper forms are only intended as a back-up option when the electronic system is not functioning.

- Any laboratory test result that is clinically significant or meets the definition of an SAE
- Any laboratory test result abnormality that required the participant to have study intervention discontinued or interrupted
- Any laboratory test result abnormality that required the participant to receive specific corrective therapy

It is expected that, wherever possible, the clinical rather than laboratory term would be used by the reporting investigator (eg, anemia vs low hemoglobin value).

9.2.7 Potential Drug-induced Liver Injury

Wherever possible, timely confirmation of initial liver-related laboratory abnormalities should occur prior to the reporting of a potential drug-induced liver injury (DILI) event. All occurrences of potential DILIs meeting the defined criteria must be reported as SAEs (see Section 9.2 and Appendix 3 for reporting details).

Potential DILI is defined as:

- Aminotransferase (AT) ALT or AST elevation > 3 times upper limit of normal (ULN)
  AND
- Total bilirubin > 2 times ULN, without initial findings of cholestasis (elevated serum alkaline phosphatase)

Approved v4.0 930169531 4.0

AND

- No other immediately apparent possible causes of AT elevation and hyperbilirubinemia, including, but not limited to, viral hepatitis, pre-existing chronic or acute liver disease, or the administration of other drug(s) known to be hepatotoxic.

9.2.8 Other Safety Considerations

Any significant worsening of conditions noted during interim or final physical examinations, ECG, x-ray filming, or any other potential safety assessment required or not required by the protocol should also be recorded as a nonserious AE or SAE, as appropriate, and reported accordingly.

9.3 Overdose

An overdose is defined as the accidental or intentional administration of any dose of a product that is considered both excessive and medically important. Overdoses that meet the regulatory definition of SAE will be reported as an SAE (see Appendix 3).

In the event of an overdose, the investigator should:

- Contact the Medical Monitor/designee immediately
- Closely monitor the participant for AEs/SAEs and laboratory abnormalities
- Document the quantity of the excess dose as well as the duration of the overdosing in the CRF

Decisions regarding dose interruptions or modifications will be made by the investigator in consultation with the Medical Monitor/designee based on the clinical evaluation of the participant and documented.

9.4 Safety

Planned time points for all safety assessments are listed in the Schedule of Activities.

9.4.1 Physical Examinations

Refer to Schedule of Activities, Section 2.

9.4.2 Vital signs

Refer to Schedule of Activities, Section 2.

9.4.3 Electrocardiograms

Refer to Schedule of Activities, Section 2.

9.4.4 Clinical Safety Laboratory Assessments

Investigators must document their review of each laboratory safety report.

All clinical safety laboratory assessments will be performed locally per Section 2 (Schedule of Activities).

Approved v4.0 930169531 4.0

Table 9.4.4-1: Clinical Laboratory Assessments

**Hematology - CBC**	
Hemoglobin<br>Hematocrit<br>Total leukocyte count, including differential<br>Platelet count	
**Chemistry**	
Aspartate aminotransferase (AST)<br>Alanine aminotransferase (ALT)<br>Total bilirubin<br>Alkaline phosphatase (ALP)<br>Lactate dehydrogenase (LDH)<br>Creatinine<br>Blood urea nitrogen (BUN) or serum urea level<br>Glucose	Albumin - screening only<br>Sodium<br>Potassium<br>Chloride<br>Calcium<br>CPK (GERMANY ONLY)<br>Phosphorus<br>TSH, free (or total) T3 and free (or total) T4 - screening<br>TSH, with reflexive f(or t)T3 and f(or t)T4 if TSH is abnormal - on treatment
**Troponin** (local standard to be used/allowed) - screening only<br>Cardiac Troponin T (cTnt) or I (cTnI): Troponin elevations will require the participant to undergo a cardiac evaluation including cardiology consultation. Following this evaluation, determination of treatment will be based on the discretion of the Investigator. See Section 9.4.5 for details.<br>**GERMANY ONLY:**<br>**Troponin** - at screening and on treatment<br>Cardiology consultation and cardiac evaluation will be required during the first instance of troponin elevation while on study intervention. Cardiology consultation and cardiac evaluation for subsequent troponin elevations will be the purview of the investigator.	
**Serology**	
Hepatitis B/C (HBsAG, HCV antibody or HCV RNA), (screening only; testing for HIV must be performed at sites where mandated by local requirements [see Appendix 9]).	
**Other Analyses**	
Pregnancy test (WOCBP only; minimum sensitivity 25 IU/L or equivalent units of HCG; screening, predose, and, follow-up. During follow-up, pregnancy testing is only required at FU Visits 1 and 2 unless increased frequency and duration is required per local regulations.	
Follicle stimulating hormone (FSH) screening - only required to confirm menopause in women < age 55. If FSH does not confirm postmenopausal status, pregnancy testing is required as per SOA.	

Abbreviations: CBC, complete blood count; CPK, creatine phosphokinase; ft3, free T3; ft4, free T4; HBsAG, hepatitis B virus surface antigen; HCG, human chorionic gonadotropin; HCV, hepatitis C virus; HIV, human immunodeficiency virus; RNA, ribonucleic acid; SOA, Schedule of Activities; T3, triiodothyronine; T4, thyroxine; TSH, thyroid stimulating hormone; tT3, total T3; tT4, total T4; WOCBP, women of child bearing potential.

9.4.5 Cardiac Evaluation

During screening, participants with troponin T (TnT) or I (TnI) between > 1 to 2× ULN will be permitted if a repeat assessment within 24 hours remains < 2× ULN and participant undergoes a cardiac evaluation and consultation with consideration for treatment based on a favorable benefit-risk assessment by the Investigator.

Troponin elevations found during treatment will require a dose delay and require that the participant undergo a cardiac evaluation including cardiology consultation to rule out myocarditis. Following this evaluation, treatment can be resumed based on investigator's judgment to proceed with treatment, participant completion of AE management as needed (ie, corticosteroid taper or participant is on ≤ 10 mg prednisone or equivalent), and participant meets the requirements per Table 7.4.1-1. Refer to Table 7.4.1-1 for myocarditis-related dose delay criteria.

A cardiac evaluation may include but is not limited to the following:

- Laboratory testing: ECG, erythrocyte sedimentation rate, c-reactive protein, repeat serum troponin levels, metabolic panel, CPK
- Cardiac imaging: Chest radiograph, echocardiogram, cardiac MRI, coronary angiogram
- Cardiac biopsy (see Section 9.8.2.5 if biopsy was performed for management of Grade ≥ 3 drug-related AE)
- Cardiology consult

9.4.6 Imaging/Other Safety Assessment

Any incidental findings of potential clinical relevance that are not directly associated with the objectives of the protocol should be evaluated and handled by the study investigator as per standard medical/clinical judgment.

9.5 Pharmacokinetics

Collect samples for PK and immunogenicity assessment for participants at the time points indicated in Table 9.5-1.

All on-treatment PK time points are intended to align with days on which study treatment is administered. If it is known that a dose is going to be delayed, then collect the predose sample just prior to the delayed dose. However, if a predose sample is collected but the dose is subsequently delayed, do not collect an additional predose sample.

Draw blood samples from a site other than the infusion site (ie, contralateral arm) on days of infusion for all pre-dose and end of infusion-PK (EOI-PK) samples. Please ensure accurate documentation of the time and date of sample collection. Draw the EOI-PK when all the study drug has been infused. If the site infuses drug without a flush, then collect the EOI-PK sample within approximately 5 minutes after end of infusion. If a flush is administered to clear the IV lines of the drug and to ensure delivery of the entire drug dose, then draw the EOI-PK sample within approximately 5 minutes after end of the flush. Do not draw EOI samples from the same IV access that the drug was administered. If the infusion was interrupted, the interruption details will also be documented on the CRF.

Further details of sample collection, processing, and shipment will be provided in the laboratory manual. For participants < 26 kg or those who require blood draw volume modifications for other reasons, sample collection volumes will be appropriately reduced per guidelines outlined in the

Approved v 4.0 930169531 4.0

laboratory manual to meet pediatric institutional guidelines for maximum daily and monthly blood draw limits.

PK of relatlimab and nivolumab will be derived from serum concentration data. The PK parameters to be assessed, depending on the availability of data, include the following:

1) Ctrough: Trough observed serum concentration
2) Ceoinf: Observed concentration at the end of infusion

Table 9.5-1 lists the sampling schedule to be followed for the assessment of PK. Further details of blood collection and processing will be provided to the site in the procedure manual.

PK/IMG collections should be stopped for participants who start on subsequent systemic therapy.

Treatment assignments will be released to the bioanalytical laboratory in order to minimize unnecessary analysis and/or reanalysis of PK/anti-drug antibody (ADA) samples.

Concentration analyses for relatlimab and nivolumab will be performed by validated bioanalytical method(s).

Bioanalytical samples designated for assessments (eg, immunogenicity, PK, or biomarker) from the same collection time point may be used interchangeably for analyses, if required (including, but not limited to, insufficient volume for complement assessment, to follow-up on suspected immunogenicity related AE, etc).

Additionally, residual bioanalytical samples will be archived and may be used for potential exploratory bioanalysis (including, but not limited to, analysis of drug-ADA immune complexes, metabolite analyses, etc) and or for additional method purposes (including, but not limited to, cross-validation, ADA/PK selectivity, cutpoint, etc).

Potential results generated from any exploratory method are intended as informational for technology exploration purposes and will not be reported.

Approved v4.0 930169531 4.0

Table 9.5-1: Pharmacokinetic and Immunogenicity Sampling Schedule for All Arms (CA224098)

Study Day of Sample Collection (1 Cycle = 4 weeks)	Event	Time Relative to start of Dose Hour:Min	Relatlimab PK Serum Sample	Relatlimab IMG (ADA) Serum Sample	Nivolumab PK Serum Sample	Nivolumab IMG (ADA) Serum Sample
Cycle 1 Day 1	Predose[a]	0:00	X	X	X	X
	EOI[b]	0:30	X		X	
Cycle 2 Day 1	Predose[a]	0:00	X	X	X	X
Cycle 6 Day 1	Predose[a]	0:00	X	X	X	X
	EOI[b]	0:30	X		X	
Cycle 9 Day 1	Predose[a]	0:00	X	X	X	X
Cycle 12 Day 1	Predose[a]	0:00	X	X	X	X
Follow Up Visit 1			X	X	X	X
Follow Up Visit 2			X	X	X	X
**Upon Drug-related AE**						
Upon occurrence of Grade 3 and higher drug-related AE (optional)[c]			X	X	X	X

Abbreviations: ADA, anti-drug antibody; AE, adverse event; EOI, end of infusion; IMG, immunogenicity; Min, minute; PK, pharmacokinetics.

[a] All predose samples should be collected just before starting the infusion (preferably within 30 minutes). If it is known that a dose is going to be delayed, then the predose sample should be collected just prior to the delayed dose. However, if a predose sample is collected but the dose is subsequently delayed, an additional predose sample should not be collected.

[b] Since the end of infusion-PK (EOI-PK) sample is drawn with the intent of accurately estimating the maximum concentration (Cmax) of the drug, draw the EOI-PK when all the study drug has been infused. If the site infuses drug without a flush, then collect the EOI-PK sample within approximately 5 minutes after end of infusion. If a flush is administered to clear the IV lines of the drug and to ensure delivery of the entire drug dose, then draw the EOI-PK sample within approximately 5 minutes after end of the flush. Do not draw EOI samples from the same IV access that the drug was administered.

[c] Optional sample should be collected before dosing is resumed (± 7 days).

9.6 Immunogenicity Assessments

Serum samples for analysis of development of ADAs will be collected in conjunction with analysis of relatlimab and nivolumab serum concentrations and will be collected from all participants as indicated in Table 9.5-1. These serum samples will be analyzed for relatlimab and nivolumab

Approved v4.0 930169531 4.0

ADAs by validated immunoassay; samples may also be analyzed for neutralizing antibodies by validated methods. Samples collected from a participant in the nivolumab monotherapy group will only be analyzed for nivolumab ADAs. Selected serum samples may be analyzed by an exploratory orthogonal method that measures anti-relatlimab or anti-nivolumab antibodies. Potential results generated from any orthogonal method are intended as informational for technology exploration purposes and will not be reported. In addition, serum samples designated for PK or biomarker assessments may also be used for immunogenicity analysis if required (eg, insufficient volume for complete immunogenicity assessment or to follow up on suspected immunogenicity related AE). Further details of blood collection and processing will be provided to the site in the procedure manual.

9.7 Genetics

Pharmacogenomic assessments will be performed including but not limited to whole genome sequencing in whole blood, peripheral blood mononuclear cells, and/or tissue as described below in Section 9.8.

9.8 Biomarkers

A variety of factors that could potentially predict clinical response and incidence of AEs to nivo + rela FDC or nivolumab monotherapy will be investigated in peripheral blood and in tissue/tumor specimens taken from all participants prior to treatment and as outlined in Table 9.8-1. Data from these investigations will be evaluated for associations with efficacy and/or safety (ie, AE) data, and characterization of pharmacodynamic markers changing with treatment.

All samples collected may also be used for future exploratory analyses (unless restricted by local requirements and/or institutional policies) to assess biomarkers associated with melanoma or immunotherapy treatment. The biomarker-sampling schedule for this study is provided in Table 9.8-1. A separate procedure manual outlining details of biomarker sample collection, processing, handling, and shipment of all samples described herein will be provided to participating study sites.

Biomarker collections should be stopped for participants who start on subsequent systemic therapy.

For participants < 26 kg or those who require blood draw volume modifications for other reasons, sample collection volumes will be appropriately reduced per guidelines outlined in the laboratory manual to meet pediatric institutional guidelines for maximum daily and monthly blood draw limits.

Approved v4.0 930169531 4.0

Table 9.8-1: Biomarker Sampling Schedule: All Arms

Study Day of Sample Collection[a] (1 cycle = 4 weeks)	Serum Biomarkers	Whole Blood PBMC	Whole Blood RNA[b]	Whole Blood DNA[b,c]	Whole Blood Immuno-phenotyping	Plasma ctDNA[b]	Plasma Biomarkers	Tumor Biopsy	Organ Biopsy (optional)	SARS-CoV-2 Serology
Screening						X		X[d]		
Cycle 1 Day 1	X	X	X	X	X	X	X			X
Cycle 2 Day 1	X	X	X		X	X	X			
Cycle 3 Day 1	X					X	X			
Cycle 6 Day 1	X	X	X		X	X	X			
Cycle 9 Day 1	X					X	X			
Cycle 12 Day 1						X				
Follow Up Visit 2; then every 26 weeks (ie, at every alternating Survival Visit ± 90 days), but not to exceed 272 days between collections, thereafter until end of study or first recurrence, whichever comes first						X				
Upon First Recurrence - excluding MMIS[e]	X	X	X		X	X	X	X[f]		
Upon occurrence of ≥ Grade 3 drug-related AE (optional)[g]	X	X	X				X		X[h]	

Abbreviations: AE, adverse event; ctDNA, circulating tumor DNA; DNA, deoxyribonucleic acid; FFPE, formalin-fixed paraffin-embedded; LAG-3, lymphocyte activation gene 3; MMIS, malignant melanoma in situ; PBMC, peripheral blood mononuclear cell; PD-L1, programmed death ligand-1; RNA, ribonucleic acid; SAE, serious adverse event; SARS-CoV-2, severe acute respiratory syndrome coronavirus 2.

[a] Biomarker sampling usually occurs prior to dosing of study drug, however, it can occur ±3 days from the scheduled dose, with the exception of C1D1. Biomarker sampling on C1D1 must be taken prior to first dose.

[b] Denmark will not collect whole blood RNA, whole blood DNA, or plasma ctDNA.

[c] Whole blood DNA sample to be collected on C1D1. However, if C1D1 is missed, it can be collected at any other visit.

Approved v4.0 930169531 4.0

[d] Tumor tissue from surgery or presurgical biopsy within 90 days of randomization, with no intervening systemic anti-cancer therapy between time of acquisition and enrollment. Participant will have LAG-3 and PD-L1 expression tested. If insufficient tumor tissue content is provided for analysis, additional tumor tissue (block and/or slides) for the biomarker analysis will be requested.

[e] To be collected before subsequent systemic therapy and/or before subsequent radiation therapy. Can be collected up to 90 days after recurrence as long as subsequent therapy has not been started as noted above. Biomarker collections should be stopped for participants who start on subsequent systemic therapy.

[f] If recurrence biopsy or surgical resection is medically feasible, a tumor sample (FFPE block or 20 positively charged unstained slides) must be submitted to the central laboratory within 30 days of collection. Fine needle aspirates or other cytology samples are not acceptable. Biopsies of bone lesions that do not have a soft tissue component are not acceptable.

[g] Optional sample should be collected before dosing is resumed (± 7 days).

[h] Only if participant has consented to optional specimen submission: If biopsy is performed for management of ≥ Grade 3 drug-related AE, leftover tissue from effected organ biopsy and/or blood samples should be submitted to explore association with SAEs (see Section 9.8.2.5).

Approved v4.0 930169531 4.0

9.8.1 Peripheral Blood Markers

A variety of factors that may impact the immunomodulatory properties and efficacy of nivo + rela FDC may be investigated in peripheral blood specimens taken from all participants prior to or during treatment. Data from these investigations will be evaluated for associations with efficacy and/or safety (AE) data. Several analyses may be completed and are described briefly below.

9.8.1.1 Serum Biomarkers

Soluble factors, such as cytokines, chemokines, soluble receptors, and antibodies to tumor antigens may be characterized and quantified by immunoassays in serum. Analyses may include, but not limited to, soluble LAG-3, FGL-1, IFN-γ, chemokine (C-X-C motif) ligand 9 (CXCL-9), interleukin-2 receptor alpha (IL-2Rα), and extracellular matrix components. Collected serum samples may also be used for the assessment of tumor antigen-specific responses elicited following treatment with monotherapy and combination therapy to explore which anti-tumor antibodies are most associated with clinical response. Antibody levels to cancer test antigens may be assessed by multiplex assays and enzyme-linked immunosorbent assay (ELISA).

9.8.1.2 Immunophenotyping

Peripheral blood samples will be collected prior to initiation of study therapy and at designated time points on-treatment for immune cell subset analysis (Table 9.8-1). Blood samples may be used for immunophenotyping or characterization of the immune cell subsets in the circulation, including, but not limited to, T-cells, B-cells, natural killer (NK) cells, myeloid-derived suppressor cells (MDSC), or subpopulations of the aforementioned immune cell types. These samples may also be used to assess immune cell function or antigen specific T-cell proliferation or activation pending emerging information from other studies.

9.8.1.3 Whole Blood DNA/RNA Analysis

Whole blood will be collected from participants prior to treatment and on study to use as a substrate for various extractions to aid in our understanding of immunotherapy benefit, predictive and prognostic biomarkers, immunotherapy resistance, and AEs. Genomic expression analysis of RNA derived from whole blood collected pre-treatment and on-study may provide information on the broad effects of nivolumab combined with relatlimab and nivolumab monotherapy on gene expression that regulates immune modulation. Exploratory RNA expression analyses may include, but is not limited to, RNA-sequencing and microRNA analysis.

Genomic DNA will be extracted from whole blood samples for translational research activities including, but not limited to, the use of next generation sequencing (NGS) technologies to enable whole genome sequencing or whole exome sequencing (WES) along with assessing single nucleotide polymorphisms and the evaluation of other germline mutations/genetic variations that may be used to evaluate associations with immunotherapy benefit, recurrence, and AEs. Genomic DNA from blood samples may be used as a germline reference to determine somatic mutations within tumor samples.

In addition, RNA or DNA derived from peripheral blood may be assessed for somatic T-cell receptor rearrangements to provide information regarding the clonality of a T-cell repertoire,

Approved v4.0 930169531 4.0

which may change with immunotherapy treatment. In addition, baseline T-cell repertoire may be predictive of benefit of nivolumab combined with relatlimab and nivolumab monotherapy.

9.8.1.4 Circulating Tumor DNA Analysis and Other Plasma Biomarkers

The presence of cell-free DNA in circulating blood is a well-documented phenomenon. Fragments of DNA are shed into the blood stream from dividing cells during cell proliferation or cell death. In patients with cancer, a fraction of this DNA is tumor derived and is termed circulating tumor DNA (ctDNA). Albeit small, fragments of DNA average between 180 to 200 base pairs and specific genomic regions can be amplified with polymerase chain reaction. Moreover, several studies have detected mutations in ctDNA that exactly correspond to mutations from the parent tumor, using tissue and plasma from participants with known driver mutations in melanoma or head and neck cancer. Plasma samples may also be used for other biomarker investigation including but not limited to exosomal biomarkers.

9.8.2 Tumor Samples

Tumor biopsy specimens will be obtained from consenting participants prior to treatment to characterize immune cell populations, evaluate expression of selected tumor and immune markers, and assess gene expression and tumor genomics. Recent archived tumor sample less than 90 days old with no intervening systemic anticancer therapy, or tumor tissue acquired within 90 days of randomization as a fresh biopsy during screening (preferred) must be available for submission prior to randomization. Submission of on recurrence biopsy samples must be collected if medically feasible and submitted to the central laboratory within 30 days of collection.

9.8.2.1 Tumor Sample Collection

The Investigator, in consultation with the radiology staff, must determine the degree of risk associated with the procedure and find it acceptable. Biopsies may be done with local anesthesia or conscious sedation. Institutional guidelines for the safe performance of biopsies should be followed. Excisional biopsies may be performed to obtain tumor biopsy samples. Invasive procedures that require general anesthesia should not be performed to obtain a biopsy specimen; however, if a surgical procedure is performed for a clinical indication, excess tumor tissue may be used for research purposes with the consent of the participant. Detailed instructions of the obtaining, processing, labeling, handling, storing, and shipping of specimens will be provided in a separate Laboratory Manual.

A FFPE tissue block (strongly preferred) 20 mm^3 tumor tissue or 20 positively charged unstained slides (minimum of 15) of tumor tissue obtained from surgical specimen, core biopsy, punch biopsy, or excisional biopsy collected within 90 days prior to randomization, with no other intervening systemic anti-cancer treatment between time of acquisition and enrollment, with an associated pathology report, must be submitted to the central laboratory (preferably prior to randomization). Fine needle aspirations or other cytology samples are not acceptable. Biopsies of bone lesions that do not have a soft tissue component are not acceptable. Tumor samples submitted during screening must contain a minimum of 100 viable tumor cells for adequate biomarker testing. If insufficient tumor tissue content is provided for analysis, additional tumor tissue (block and/or slides) for the biomarker analysis will be requested.

Approved v4.0 930169531 4.0

9.8.2.2 LAG-3 and PD-L1 Expression

This study will be assessing for LAG-3 and PD-L1 expression in tumor specimens. LAG-3 expression on immune cells and PD-L1 expression on tumor cells will be measured using analytically validated immunohistochemical assays. Participant will have LAG-3 and PD-L1 expression tested.

9.8.2.3 Characterization of Tumor-infiltrating Lymphocytes and Immune Biomarkers

IHC or other technologies may be used to assess tumor markers and the number and composition of immune infiltrates in order to define the immune cell subsets present within FFPE tumor tissue before and after exposure to therapy. These IHC analyses may include, but not necessarily be limited to, the following markers: LAG-3, FGL-1, PD-L1, CD8, T cell immunoglobulin and mucin domain 3 (TIM-3), forkhead box P3 (FOXP3), CD56, CTLA-4, PD-1, MHC Class I, MHC Class II, components of antigen presentation machinery, and fibrotic markers.

9.8.2.4 Tumor Genotyping, Mutational Analysis, and Gene Expression Profiling

DNA and/or RNA isolated from the tumor tissue collected at baseline and on treatment may be analyzed using sequencing techniques including but not limited to whole exome and whole genome sequencing to assess tumor associated mutations and various gene expression signatures associated with inflammation (eg, IFNγ) and/or immune infiltration (eg, CD8, FoxP3), and their effect on response, survival or safety. DNA and/or RNA isolated from the tumor tissue may also be used to assess the composition of the T-cell receptor repertoire and its association with response to treatment.

9.8.2.5 Tumor Markers Associated with Adverse Events

Upon occurrence of ≥ Grade 3 drug-related AE, if a biopsy of the affected organ is performed, it is strongly recommended that a specimen for biomarker analysis be collected for potential predictive markers of toxicity. A deidentified copy of the pathology report should be provided to the central lab if tissue is submitted. Such analyses of these tissue specimens may include but are not limited to the assessment of cell markers via protein expression or molecular/genomic assessments (eg, DNA and RNA extraction followed by the appropriate biomarker assay and subsequent analysis). Examples include but are not limited to H&E staining, IHC assays, RNA-sequencing, and the use of NGS technologies to enable whole genome sequencing and WES. Blood samples may also be collected to explore biomarkers associated with SAE, see Table 9.8-1.

9.9 Additional Research

This protocol will include residual sample storage for additional research.

For All US Sites

Additional research is required for all study participants, except where prohibited by IRBs/ethics committees, prohibited by local laws or regulations, or academic/institutional requirements. Where one or more of these exceptions occurs, participation in the additional research should be encouraged but will not be a condition of overall study participation.

Approved v4.0 930169531 4.0

- If the IRB/ethics committees and site agree to the mandatory additional research retention and/or collection, then the study participant must agree to the mandatory additional research as a requirement for inclusion in the study.
- If optional participation is permitted and approved, then the study participants may opt out of the additional research retention and/or collection.

For non-US Sites

Additional research is optional for all study participants, except where retention and/or collection is prohibited by local laws or regulations, ethics committees, or institutional requirements.

This collection for additional research is intended to expand the translational R&D capability at BMS, and will support as yet undefined research aims that will advance our understanding of disease and options for treatment. It may also be used to support health authority requests for analysis, and advancement of pharmacodiagnostic development to better target drugs to the right patients. This may also include genetic/genomic exploration aimed at exploring disease pathways, progression and response to treatment etc.

Sample Collection and Storage

All requests for access to samples or data for additional research will be vetted through a diverse committee of the study Sponsor's senior leaders in Research and Development (or designee) to ensure the research supports appropriate and well-defined scientific research activities.

Residual PK samples, serum, plasma, whole blood, PBMC, and tissue/tumor biopsy from scheduled and unscheduled collections (see Table 9.9-1) will also be retained for additional research purposes. This includes all materials derived and extracted from these samples including but not limited to DNA, RNA, proteins, and other soluble factors.

Samples kept for future research will be stored at the BMS Biorepository in New Jersey, USA or an independent, BMS-approved storage vendor.

The manager of these samples will ensure they are properly used throughout their usable life and will destroy the samples at the end of the scheduled storage period, no longer than fifteen (15) years after the end of the study or the maximum allowed by applicable law.

Transfers of samples by research Sponsor to third parties will be subject to the recipient's agreement to establish similar storage procedures.

Samples will be stored in a coded fashion, and no researcher will have access to the key. The key is securely held by the Investigator at the clinical site, so there is no direct ability for a researcher to connect a sample to a specific individual.

Further details of sample collection and processing will be provided to the site in the procedure manual.

Approved v4.0 930169531 4.0

Table 9.9-1: Residual Sample Retention for Additional Research Schedule

Sample Type	Time points for which residual samples will be retained
PK/IMG	All
Tumor Biopsy	All
Surgical tissue	All
Serum	All
PBMC	All
Whole blood	All
Plasma	All
Isolated DNA/RNA	All
Cell pellets obtained from ctDNA sample buffy coats	All
Other derived/extracted materials from primary collection samples	All

Abbreviations: ctDNA, circulating tumor DNA; DNA, deoxyribonucleic acid; IMG, immunogenicity; PBMC, peripheral blood mononuclear cells; PK, pharmacokinetics; RNA, ribonucleic acid.

9.10 Other Assessments

Not applicable.

9.11 Health Economics OR Medical Resource Utilization and Health Economics

Medical resource utilization and health economics data associated with medical encounters will be collected in the eCRF by the investigator and study-site personnel for all participants throughout the study. Protocol-mandated procedures, tests, and encounters are excluded.

The data collected may be used to conduct exploratory economic analyses and will include:

- Number and duration of medical care encounters, including surgeries, and other selected procedures (inpatient and outpatient).
- Duration of hospitalization (total days length of stay, including duration by wards; eg, intensive care unit).
- Number and character of diagnostic and therapeutic tests and procedures.
- Outpatient medical encounters and treatments (including physician or emergency room visits, tests and procedures, and medications).

10 STATISTICAL CONSIDERATIONS

10.1 Statistical Hypotheses

The primary hypothesis for the study is that nivo + rela FDC is superior to nivolumab monotherapy in achieving RFS as assessed by the investigator.

Approved v4.0 930169531 4.0

The null hypothesis to be tested in relation to the primary endpoint is as follows:

1) Nivo + rela FDC is not different from nivolumab monotherapy with respect to RFS per investigator.

The null hypothesis corresponding to the key secondary endpoint is as follows:

1) Nivo + rela FDC is not different from nivolumab monotherapy with respect to overall survival.

If the primary endpoint is met, the key secondary endpoint (OS) will be tested.

10.1.1 Multiplicity Adjustment

The statistical comparisons for the primary efficacy endpoint (RFS) and the key secondary endpoint (OS) will be carried out in the hierarchical order as indicated in Section 10.1. This means that a statistically significant result for the primary comparison (RFS) is required to initiate the testing of the key secondary endpoint (OS). Since a step-down procedure is used, each comparison will be tested at a significance level of 0.05 (2-sided) and an overall two-sided alpha level of 0.05 will be preserved. If the DMC requests efficacy summary data in order to perform risk-benefit assessment prior to the RFS IAs, an administrative alpha penalty will occur and will be subtracted from the overall type I error of 0.05 for each additional unplanned look requested by the DMC per Section 5.1.1.

10.2 Sample Size Determination

10.2.1 Recurrence-free Survival

The primary objective of the study is to compare RFS of nivo + rela FDC to nivolumab monotherapy in participants with completely resected Stage IIIA (> 1 mm tumor in lymph node), Stage IIIB/C/D, or Stage IV NED melanoma. The number of events and power were calculated assuming a non-proportional hazards model with a 6-month delayed treatment effect, a cure rate in the 2 treatment arms and a plateauing of treatment effect from 7.5 years. The sample size calculations were simulated using East® and Statistical Analysis System (SAS®) software (SAS Institute, North Carolina, USA).

Approximately 410 RFS events would be required in the 2 respective treatment arms for a 2-sided alpha = 0.05 stratified log rank test to show a statistically significant difference in RFS between the treatment arms with approximately 90% overall power when the average hazard ratio (HR) of nivo + rela FDC arm to nivolumab monotherapy arm is 0.72, and a cure rate of 0.52 is assumed in the nivolumab monotherapy arm (0.63 cure rate in the nivo + rela FDC arm). This assumes that the RFS rate at 36 months is 58.6% and 68.5% in the nivolumab monotherapy and nivo + rela FDC arms, respectively. Initially, the calculations were based on the following projected accrual rates: 1-3 months: 12 participants/month, 4-5 months: 30 participants/month, ≥ 6 months: 44 participants/month; it is expected to take about 27 months to accrue the approximately 1,050 participants in the study. The accrual rate assumes a 5% overall drop out (lost to follow-up) rate

Approved v4.0 930169531 4.0

in each treatment arm by 52 months. However, the accrual has been completed in approximately 14 months which influences the timing of the expected events and analyses.

Under these assumptions of accrual, the observed RFS distribution of the nivolumab monotherapy arm in the CA209238 study, and the assumed HR stated above, it would take approximately 52 months from the randomization of the first participant to observe the required number of 410 RFS events. It is projected that an observed HR of 0.815 or less would result in a statistically significant improvement at the final analysis of RFS.

Two interim analyses for efficacy (RFS) will be performed based on number of events as detailed below.

The first interim analysis for RFS (RFS IA#1) will be performed when approximately 309 RFS events have occurred (~75% information fraction). At that time, a minimum follow-up of 14 months is expected for all randomized participants (ie, approximately 28 months from first participant randomized in the study). The significance boundaries at the interim analyses will be based on an O'Brien and Fleming alpha spending function (details in Section 10.5). With an interim RFS analysis at 309 RFS events, the nominal significance level would be 0.020 (2-sided) and an observed HR of 0.765 or less would result in a statistically significant improvement, with power of 47%.

If RFS IA#1 is not statistically significant, the second interim analysis for RFS (RFS IA#2) will be performed when approximately 361 RFS events have occurred (~88% information fraction). At that time, a minimum follow-up of 24 months is expected for all randomized participants (ie, approximately 38 months from first participant randomized in the study). With an interim RFS analysis at 361 RFS events, the nominal significance level would be 0.028 (2-sided) and an observed HR of 0.793 or less would result in a statistically significant improvement at the interim analysis with cumulative power of 75%. A decision may be taken not to perform RFS IA#2 if, at the time we reach the expected number of events for RFS IA#2, the predicted time frame to reach RFS FA number of events (410 events) is less than 6 months. Alpha spending will be adjusted appropriately to not include RFS IA#2 if this is not performed.

If the final RFS analysis occurs at 410 RFS events, the critical hazard ratio would be 0.815 (average HR of 0.72) and a nominal significance level of 0.04 (2-sided). The nominal significance level used for the analyses will be calculated based on the actual number of RFS events at interim and final analyses, respectively.

10.2.2 Overall Survival

The key secondary endpoint of OS will be tested if the primary objective of RFS is statistically significant.

For the comparison of OS between nivo + rela FDC and nivolumab monotherapy in all randomized participants, approximately 309 death events would be required in the 2 respective treatment arms for a 2-sided alpha = 0.05 stratified log-rank test to show a statistically significant difference in OS between the treatment arms with approximately 70% overall power. This assumes a target HR of nivo + rela FDC vs nivolumab monotherapy of 0.75 and the OS in the nivolumab monotherapy

Approved v4.0 930169531 4.0

arm is exponentially distributed with a survival rate at Year 4 of 78%. It is projected that an observed HR of 0.794 or less would result in a statistically significant improvement at the final analysis of OS. The nominal significance level used for the analyses will be calculated based on the actual number of OS events at interim and final analyses, using the O'Brien and Fleming alpha spending function.

Assuming the RFS result is significantly superior, there will be an interim analysis of OS performed by the DMC at the time of RFS significance (either at RFS IA#1, RFS IA#2, or RFS FA). In the event that RFS is statistically significant at one of the interims (RFS IA#1, RFS IA#2), but OS is not statistically significant, an additional interim OS analysis will be performed by the DMC at approximately 52 months and, if not statistically significant, then again at approximately 71 months. Final OS analysis will be performed when approximately 309 events have occurred or at 90 months, whichever comes first. Details for the OS interim analyses are provided in Table 10.2.2-1. The significance boundaries at the interim analysis will be based on the actual number of events and an O'Brien and Fleming alpha spending function.

Table 10.2.2-1: Formal Analysis of Overall Survival - Operating Characteristics at Each Time Point

Analysis	Criterion for Conducting Analysis	Estimated Time[a]	Estimated Number of OS Events	Information Fraction	Nominal Significance Level[b] 2-sided	Approx Cumulative Power[c]
OS IA#1[d]	At the time of RFS IA#1, if significant	28	87	28%	0.0004	0.3%
OS IA#1[d]	At the time of RFS IA#2, if significant	38	128	41%	0.001	5%
OS IA[d]	At the time of RFS FA or 52 months	52	183	59%	0.007	23%
OS IA[d]	71 months after first randomized	71	250	81%	0.023	51%
OS FA	90 months after first randomized	90	309	100%	0.042	70%

Abbreviations: Approx, approximate; DMC, Data Monitoring Committee; FA, final analysis; FPFV, first patient first visit; IA, interim analysis; OS, overall survival; RFS, recurrence-free survival.

[a] From first participant randomized.

[b] Assuming that the observed number of events is exactly as in the table. The actual significance boundaries at each analysis will be calculated based on the actual number of OS events, using an O'Brien and Fleming alpha spending function.

[c] The probability of crossing the boundary under the alternative hypothesis, cumulative across time points.

[d] In the event that RFS is statistically significant at one of the RFS interim analyses (but OS interim is not statistically significant), additional interim OS analyses will be performed by the DMC at approximately Years 4.3 and 6.

Approved v4.0 930169531 4.0

10.3 Analysis Sets

For the purposes of analysis, the following populations are defined:

Population	Description
Enrolled	All participants or their legally acceptable representative who signed an informed consent and were registered into IRT.
Randomized	All participants who were randomized to any treatment arm in the study.
Safety	All participants who take at least 1 dose of double-blind study treatment.<br>Data in this data set will be analyzed based on randomized treatment, except if a participant received the same incorrect treatment throughout the study, then the participant will be analyzed based on the treatment received.
PK	All randomized participants with available serum time concentration data.
Immunogenicity	All randomized participants with available ADA data.
Biomarker	All randomized participants with available biomarker data.

Abbreviations: ADA, anti-drug antibodies; IRT, interactive response technology; PK, pharmacokinetics.

Defined Analysis Data Sets	Description
Analysis set for main estimand of RFS	All randomized participants. Refer to Table 10.4.1-2 for details of intercurrent strategy.
Analysis set for main estimand of DMFS	All randomized Stage III/IVA/IVB participants. Refer to Table 10.4.1-2 for details of intercurrent strategy.
Analysis set for main estimand of OS	All randomized participants. For participants who discontinue or complete 1-year study intervention and continue to be followed for up to 7.5 years for survival, all on treatment and off-treatment deaths will be included. Refer to Table 10.4.1-2 for details of intercurrent strategy.
Analysis set for safety	All safety events reported for all randomized participants who are exposed to study drug. For participants who discontinue study intervention, all events post-discontinuation up to Day 135 post last dose of study intervention will be included in the safety summaries.

Abbreviations: DMFS, distant metastasis-free survival; OS, overall survival; RFS, recurrence-free survival.

10.4 Statistical Analyses

The statistical analysis plan (SAP) will be finalized prior to primary endpoint DBL and it will include a more technical and detailed description of the statistical analyses described in this section. This section is a summary of the planned statistical analyses of the most important endpoints, including primary and key secondary endpoint.

A description of the participant population will be included in a statistical output report, including subgroups of age, gender, race, and other study specific populations and demographic characteristics. A description of participant disposition will also be included in the clinical study report.

Approved v4.0 930169531 4.0

10.4.1 General Considerations

RFS will be programmatically determined based on the disease recurrence date provided by the investigator and is defined as the time between the date of randomization and the first date of documented recurrence (local, regional, distant, or new primary melanoma), or death (whatever the cause), whichever occurs first. Note: a participant who dies without reported recurrence will be considered to have recurred on the date of death. For participants who remain alive and whose disease has not recurred, RFS will be censored on the date of last evaluable disease assessment. For those participants who remained alive and had no recorded post-randomization tumor assessment, RFS will be censored on the day of randomization. Censoring rules for the primary endpoint of RFS are presented in Table 10.4.1-1.

Table 10.4.1-1: Censoring Scheme for Definition of Recurrence-free Survival

Situation	Date of Event or Censoring	Outcome
Recurrence (local, regional, distant, new primary melanoma[a])	Date of first recurrence	Event
Death without recurrence	Date of death	Event
Baseline disease	Date of randomization	Censored
No baseline disease assessment	Date of randomization	Censored
No on-study disease assessments and no death	Date of randomization	Censored
No recurrence and no death	Date of last evaluable disease assessment	Censored

[a] Melanoma in situ is not considered a new primary melanoma.

The stratification factors for analysis are:

- AJCC v8 stage: Stage IIIA/IIIB vs Stage IIIC vs Stage IIID/IV (including all participants with mucosal melanoma, Stage III, Stage IVA, Stage IVB, and Stage IVC)
- Geographic region (USA/Canada/Australia vs Europe vs ROW)

The overall alpha for this study is 0.05 (2-sided) which will be passed to the key secondary endpoint of OS if the primary endpoint of RFS is statistically significant.

DMFS, by investigator, is defined as the time between the date of randomization and the date of first distant metastasis or date of death (whatever the cause), whichever occurs first. For participants who remain alive and distant metastasis-free, DMFS will be censored on the date of last evaluable disease assessment.

OS is defined as the time between the date of randomization and the date of death. For participants without documentation of death, OS will be censored on the last date the participant was known to be alive. OS will be followed continuously while participants are on the study drug and every 12 weeks via in-person or phone contact after participants discontinue the study drug.

Approved v4.0 930169531 4.0

Table 10.4.1-2: Definition of Estimands for Primary and Secondary Endpoints

Objective	Endpoint	Estimand
**Primary**		
To compare the efficacy, as measured by RFS, provided by nivo + rela FDC vs nivolumab monotherapy in participants with completely resected Stage III/IV NED melanoma.	Recurrence or death per investigator	**Population:** All randomized participants with completely resected Stage III/IV NED melanoma.<br>**Population level summary:** Hazard ratio of the RFS with associated CI<br>**Treatment:** Nivo + rela FDC compared to nivolumab monotherapy<br>**Intercurrent Events (Strategy)**:<br>• Randomized but not treated (treatment policy)<br>• Discontinued treatment (treatment policy)<br>• Start of new systemic anticancer therapy, tumor-directed radiotherapy, or tumor-directed surgery (treatment policy)<br>• Melanoma in situ, second non-melanoma primary cancer, or non-invasive BCC/SCC (treatment policy)
**Key Secondary Efficacy**		
To compare the OS provided by nivo + rela FDC vs nivolumab monotherapy in participants with completely resected Stage III/IV NED melanoma.	OS	**Population:** All randomized participants with completely resected Stage III/IV NED melanoma.<br>**Population level summary**: Hazard ratio of the OS with associated CI<br>**Treatment:** Nivo + rela FDC compared to nivolumab monotherapy<br>**Intercurrent Events (Strategy):**<br>• Randomized but not treated (treatment policy)<br>• Discontinued treatment (treatment policy)<br>• Start of new systemic anticancer therapy, tumor-directed radiotherapy, or tumor-directed surgery (treatment policy)<br>• Melanoma recurrence, melanoma in situ, second non-melanoma primary cancer, or non-invasive BCC/SCC (treatment policy)
**Other Secondary Efficacy**		
To assess the efficacy as measured by distant metastasis-free survival (DMFS), provided by nivo + rela FDC vs nivolumab monotherapy in participants with completely resected Stage III/IVA/IVB NED melanoma.	Distant recurrence or death per investigator	**Population:** All randomized Stage III/IVA/IVB NED participants<br>**Population level summary:** Hazard ratio of the DMFS with associated CI<br>**Treatment:** Nivo + rela FDC compared to nivolumab monotherapy<br>**Intercurrent Events (Strategy)**:

Approved v4.0 930169531 4.0

Table 10.4.1-2: Definition of Estimands for Primary and Secondary Endpoints

Objective	Endpoint	Estimand
		• Randomized but not treated (treatment policy)<br>• Discontinued treatment (treatment policy)<br>• Local or regional recurrence (treatment policy)<br>• Start of new systemic anticancer therapy, tumor-directed radiotherapy or tumor-directed surgery (treatment policy)<br>• Melanoma in situ, second non-melanoma primary cancer, or non-invasive BCC/SCC (treatment policy)
To compare PFS2 between nivo + rela FDC vs nivolumab monotherapy in randomized participants.	PFS2	**Population:** All randomized participants<br>**Population level summary:** Hazard ratio of PFS2 with associated CI<br>**Treatment:** Nivo + rela FDC compared to nivolumab monotherapy<br>**Intercurrent Events for PFS2 (Strategy)**: Details are presented in the Statistical Analysis Plan
**Safety**		
To assess safety and toxicity of nivo + rela FDC vs nivolumab monotherapy in participants with completely resected Stage III/IV NED melanoma.	Incidence and severity of AE, SAEs, IMAEs, drug-related AE/SAE, AEs leading to discontinuation, deaths, other select AEs and laboratory abnormalities.	**Population:** Safety analysis population (defined in Section 10.3)<br>**Population level summary:** Percentage of participants reporting:<br>• AEs, SAEs, IMAES, drug-related AE/SAEs, other select AEs and laboratory abnormalities up to 135 days post last dose of study treatment.<br>• Deaths until study termination.<br>• AEs leading to discontinuation, until discontinuation of study treatment.<br>**Treatment:** Nivo + rela FDC compared to nivolumab monotherapy<br>**Intercurrent Events (Strategy)**: All ICEs (ie, start of subsequent anti-cancer therapy) will be handled with treatment policy (ie, include AEs after IEs if AEs are within 30 or 135 days after last dose of study medication).

Abbreviations: AE, adverse event; BCC, basal cell carcinoma; CI, confidence interval; DMFS, distant metastasis-free survival; FDC, fixed dose combination; ICEs, intercurrent events; NED, no evidence of disease; OS, overall survival; PFS2, progression-free survival 2; RFS, recurrence free survival; SAEs, serious adverse events; SCC, squamous cell carcinoma.

[a] Will only be provided if the endpoint is tested based on hierarchical strategy.

Approved v4.0 930169531 4.0

10.4.2 Primary Endpoint(s)

Table 10.4.2-1: Primary Endpoints

Primary Endpoint	Description	Timeframe
**Primary Efficacy**		
Recurrence Free Survival (RFS)	Time from randomization to the first documentation of recurrence (local, regional, distant, new primary melanoma), or death due to any cause, whichever occurs first.	Until recurrence event or death, whichever is earlier. Two interim analyses are planned, see Section 10.5 for details.

Table 10.4.2-2: Summary of Primary Endpoint Analysis

Endpoint	Statistical Analysis Methods
RFS as assessed by the investigator. RFS is defined as the time between the date of randomization and the first date of documented recurrence (local, regional, distant, new primary melanoma) or death due to any cause, whichever occurs first.	RFS distributions will be compared between treatment groups (nivo + rela FDC vs nivolumab monotherapy) using a 2-sided log-rank test stratified by AJCC stage at screening and geographic region in all randomized population. The hazard ratio and corresponding 100x (1-adjusted α)% CIs will be estimated using a Cox proportional hazards model, with treatment group as a single covariate, stratified by the above factors.<br>RFS curves will be estimated using K-M product-limit methodology. Median RFS with 2-sided 95% CIs using the log-log transformation will be computed. In addition, RFS rates at 6, 12, 18, 24, and 36 months (and yearly after depending on follow-up) with 2-sided 95% CIs using the log-log transformation will be computed.<br>The proportional hazards assumption will be assessed at the time of analysis and appropriate methods will be employed in case of non-proportional hazards, as sensitivity analysis for RFS endpoint.

Abbreviations: AJCC, American Joint Committee on Cancer; CI, confidence interval; FDC, fixed dose combination; K-M, Kaplan Meier; RFS, recurrence-free survival.

For the primary endpoint of RFS, a supplemental analysis will be performed using a hypothetical estimand strategy to handle the intercurrent events (ICEs) of start of new systemic anticancer therapy, tumor-directed radiotherapy, tumor-directed surgery or second non-melanoma primary cancer. Under this strategy, RFS will be censored on the date of last evaluable disease assessment prior to or on the same date of occurrence of the above ICEs (whichever occurs first). All other ICEs will be handled in the same manner as the main estimand (Table 10.4.1-2).

For the analysis the RFS curves will be estimated using Kaplan Meier (K-M) product-limit methodology. Median RFS with 2-sided 95% CIs using the log-log transformation will be computed.

Approved v4.0 930169531 4.0

10.4.3 Secondary Endpoint(s)

Table 10.4.3-1: Secondary Efficacy Endpoints

Secondary Endpoints	Description	Timeframe
**Key Secondary Efficacy**		
Overall Survival	Time from randomization date until death (whatever the cause)	Until death, or until OS final analysis provided in Table 10.2.2-1
**Other Secondary Efficacy**		
Distant metastasis-free survival	Time from date of randomization to the date of first distant metastasis or date of death (whatever the cause, whichever occurs first).	Until distant metastasis or death, or until OS final analysis provided in Table 10.2.2-1

Table 10.4.3-2: Summary of Secondary Endpoint Analysis

Secondary Endpoints	Statistical Analysis Methods
**Key Secondary**	
OS is defined as the time between the date of randomization and the date of death (whatever the cause).	If the RFS endpoint is statistically significant, then OS distributions will be compared between the 2 treatment arms (nivo + rela FDC vs nivolumab monotherapy) using a 2-sided log-rank test stratified by AJCC stage at screening and geographic region in all randomized participants.<br>The hazard ratio and corresponding 2-sided (100-adjusted α)% CIs will be estimated using a Cox proportional hazards model, with treatment group as a single covariate, stratified by the above factors. OS curves will be estimated using K-M product-limit methodology. Median OS with 2-sided 95% CIs using the log-log transformation will be computed. In addition, OS rates at 6, 12, 18, 24, and 36 months (and yearly after depending on follow-up) with 2-sided 95% CIs using the log-log transformation will be computed.
**Other Secondary**	
DMFS, by investigator, is defined as the time between the date of randomization and the date of first distant metastasis or date of death (whatever the cause), whichever occurs first.	The DMFS hazard ratio and corresponding 2-sided 95% CIs will be estimated using a Cox proportional hazards model, with treatment group as a single covariate, stratified by the above factors. DMFS curves will be estimated using K-M product-limit methodology. Median DMFS with 2-sided 95% CIs using the log-log transformation will be computed. In addition, DMFS rates at 6, 12, 18, 24, and 36 months (and yearly after depending on follow-up) with 2-sided 95% CIs using the log-log transformation will be computed.
PFS2 defined as time from randomization to second recurrence/objective disease progression on next-line systemic therapy per investigator, or death from any cause, whichever occurs first.	The hazard ratio of PFS2 and corresponding 95% CI will be estimated using a Cox proportional hazards model, with treatment group as a single covariate. No multiplicity adjustment will be applied.

Approved v4.0 930169531 4.0

Abbreviations: AJCC, American Joint Committee on Cancer; CI, confidence interval; DMFS, distant metastasis free survival; FDC, fixed dose combination; K-M, Kaplan Meier; OS, overall survival; PFS2, progression-free survival 2.

10.4.3.1 Safety Analysis

Table 10.4.3.1-1: Safety Analysis

Safety Endpoint	Description	Timeframe
Safety		
Incidence and severity of AEs, SAEs, IMAEs, other select AEs, laboratory abnormalities, drug related AEs/SAEs, death	Incidence and severity (i.e. CTC grade) of AEs, SAEs, IMAEs, other select AEs, laboratory abnormalities, drug related AEs/SAEs, death	Up to 135 days after the last dose of study treatment
Incidence of AEs leading to discontinuation	Incidence and severity of AEs leading to discontinuation of study treatment	Up to discontinuation of study treatment

Abbreviations: AE, adverse event; CTC, Common Terminology Criteria; IMAEs, immune mediated adverse events; SAE, serious adverse event.

10.4.4 Exploratory Endpoint(s)

Exploratory analyses will be described in the statistical analysis plan finalized before database lock. Details of the PK, pharmacodynamics, patient-reported outcomes, and biomarker exploratory analyses will be described in the statistical analysis plan finalized before database lock.

10.4.5 Other Safety Analysis

All safety analyses will be performed using the Safety analysis population.

Endpoint	Statistical Analysis Methods
The Safety and tolerability objective will be measured by the incidence and severity of adverse events (AEs), serious adverse events (SAEs), and adverse events leading to discontinuation study drug, select AEs, immune-mediated AEs (IMAEs), other events of special interest (OESIs), deaths, and laboratory abnormalities in each arm.	All safety analyses will be performed using the safety analysis population. The frequency of deaths, adverse events (AEs), serious adverse events (SAEs), AEs leading to discontinuation of study drug, select AEs, immune-mediated AEs (IMAEs), other events of special interest (OESIs), and abnormalities in specific clinical laboratory assessments will be presented. The safety summaries will be presented by severity were applicable. Analyses will be conducted using the 30-day and/or 135-day safety window from day of last dose received. AEs will be coded using the most recent Medical Dictionary for Regulatory Activities (MedDRA) version available during the conduct of the study. AEs and laboratory values will be graded for severity according to the National Cancer Institute (NCI) Common Terminology Criteria for Adverse Events (CTCAE) version 5.0.

Approved v4.0 930169531 4.0

10.4.6 Other Analyses

10.4.6.1 Pharmacokinetic Analyses

Trough concentrations of relatlimab will be plotted versus study day and cycle. Nivolumab end of infusion (Ceoi) and trough (Ctrough) concentrations will be tabulated using summary statistic, based on the PK analysis population.

The nivolumab and relatlimab concentrations data obtained in this study may be combined with data from other studies in the clinical development program to develop or refine a PPK model. This model may be used to evaluate the effects of intrinsic and extrinsic covariates on the PK and to determine measures of individual exposure (such as steady-state peak, trough, and time-averaged concentration). In addition, model-determined exposures may be used for exposure-response analyses. Results of population PK and exposure response-analyses will be reported separately.

10.4.6.2 Immunogenicity Analyses

All immunogenicity analyses will be performed using the Immunogenicity Evaluable Participants population.

A listing will be provided for all available immunogenicity data. Baseline ADA positive participant is defined as a participant with positive seroconversion detected in the last sample before initiation of treatment. ADA-positive participant is a participant with at least 1 ADA-positive sample relative to baseline after initiation of the treatment. For each drug, frequency distribution of baseline ADA positive participants and ADA positive participants after initiation of the treatment will be summarized. This analysis will be based on the immunogenicity analysis population. To examine the potential relationship between immunogenicity and safety, a table summarizing the frequency and type of AEs of special interest may be explored by immunogenicity status. In addition, potential relationships between immunogenicity and efficacy and/or PK may also be explored.

10.4.6.3 Patient-Reported Outcomes

Unless otherwise specified in the SAP, all exploratory PRO endpoints will be performed in the randomized population (defined in Section 10.3).

10.5 Interim Analyses

10.5.1 Interim Analyses of RFS

For the primary endpoint of RFS, up to 2 interim analyses for efficacy (RFS) will be performed by the DMC. The 2 interim analyses for efficacy (RFS) will be performed based on number of events. The first interim analysis for RFS (RFS IA#1) will be performed when approximately 309 RFS events have occurred (~75% information fraction). If RFS IA#1 is not statistically significant, the second interim analysis for RFS (RFS IA#2) will be performed when approximately 361 RFS events have occurred (~88% information fraction). Please see Section 10.2.1 for specific details.

Approved v4.0 930169531 4.0

10.5.2 Interim Analysis of OS

Assuming the RFS result is significantly superior, there will be an interim analysis of OS performed by the DMC at the time of RFS significance (either at RFS IA#1, RFS IA#2, or RFS FA). In the event that RFS is statistically significant at one of the interims (RFS IA#1, RFS IA#2), but OS is not statistically significant, an additional interim OS analysis will be performed by the DMC at approximately 52 months and, if not statistically significant, then again at approximately 71 months. Please see Section 10.2.2 for specific details.

10.5.3 Interim Analyses of RFS and OS: Additional Details

These interim analyses for RFS and OS will allow for early formal testing for superiority, and the boundaries for declaring superiority will be derived based on the actual number of events using Lan-DeMets spending function with O'Brien and Fleming type of boundary based on the information fraction observed.

An independent statistician external to BMS will perform interim analysis in conjunction with a review by the DMC. The DMC/Review Board charter will describe the procedures related to DMC operations in greater detail. The SAP will describe the planned interim analyses in greater detail.

Approved v4.0 930169531 4.0

11 REFERENCES

1 Mellman I, Coukos G, Dranoff G. Cancer immunotherapy comes of age. Nature. 2011;480:480-9.

2 Younes A, Santoro A, Zinzani PL, et al. Checkmate 205: Nivolumab (nivo) in classical Hodgkin lymphoma (cHL) after autologous stem cell transplant (ASCT) and brentuximab vedotin (BV)-A phase 2 study. J Clin Oncol. 34(Suppl 15):7535.

3 Moehler M, Shitara K, Garrido M, et al. Nivolumab (nivo) plus chemotherapy (chemo) versus chemo as first-line (1L) treatment for advanced gastric cancer/gastroesophageal junction cancer (GC/GEJC)/esophageal adenocarcinoma (EAC): First results of the CheckMate 649 study. Proceedings of the ESMO Virtual Congress; 2020 Sep 19-21 Abstract LBA6_PR.

4 Brahmer JR, Drake CG, Wollner I, et al. Phase I study of single-agent anti–programmed death-1 (MDX-1106) in refractory solid tumors: safety, clinical activity, pharmacodynamics, and immunologic correlates. J Clin Oncol. 2010; 28:3167-75.

5 Andrews LP, Marciscano AF, Drake CG et al. LAG3 (CD223) as a cancer immunotherapy target. Immunol Rev 2017; 276: 80–96.

6 Wang J, Sanmamed MF, Datar I, et al. Fibrinogen-like protein 1 is a major immune inhibitory ligand of LAG-3. Cell. 2019;176: 334-47.

7 Okazaki T, Okazaki IM, Wang J, et al. PD-1 and LAG-3 inhibitory coreceptors act synergistically to prevent autoimmunity in mice. J Exp Med 2011;208:395-407.

8 Investigator Brochure Relatlimab BMS-986016. Bristol Myers Squibb Company; 2022. Document Control No. 930071620.

9 Chauvin JM, Pagliano O, Fourcade J, et al. TIGIT and PD-1 impair tumor antigen-specific $CD8^+$ T cells in melanoma patients. J Clin Invest. 2015; 125:2046-58.

10 Speiser DE, Utzschneider DT, Oberle SG, et al. T cell differentiation in chronic infection and cancer: functional adaptation or exhaustion? Nat Rev Immunol. 2014;14:768-74.

11 Sharma P, Hu-Lieskovan S, Wargo JA, et al. Primary, adaptive, and acquired resistance to cancer immunotherapy. Cell. 2017;168:707-23.

12 Woo SR, Turnis ME, Goldberg MV, et al. Immune inhibitory molecules LAG-3 and PD-1 synergistically regulate T-cell function to promote tumoral immune escape. Cancer Res. 2012;72:917–27.

13 Ascierto PA, Melero I, Bhatia S, et al. Initial efficacy of anti-lymphocyte activation gene-3 (anti–LAG-3; BMS-986016) in combination with nivolumab (nivo) in pts with melanoma (MEL) previously treated with anti–PD-1/PD-L1 therapy. J Clin Oncol 2017; 35(Suppl 15):9520.

Approved v4.0 930169531 4.0

14 Ascierto PA, Bono P, Bhatia S, et al. Efficacy of BMS-986016, a monoclonal antibody that targets lymphocyte activation gene-3 (LAG-3), in combination with nivolumab in pts with melanoma who progressed during prior anti-PD-1/PD-L1 therapy (mel prior IO) in all-comer and biomarker enriched populations. Proceedings of the ESMO Congress; 2017 Sep 8-12; Madrid, Spain. Abstract 4998.

15 Long GV et al. Journal of Clinical Oncology 2022;40(36_supp): 360385-360385.

16 Ossio R, Roldan-Marin R, Martinez-Said H, et al. Melanoma: a global perspective. Nat Rev Cancer. 2017;17(7):393-39.

17 Cancer Statistics Center. 2020 Estimates. American Cancer Society. https://cancerstatisticscenter.cancer.org/?_ga=2.225532186.1475645657.1590009500-2006917398.1590009500#!/ Accessed 20 May 2020.

18 Gershenwald JE, Scolyer RA, Hess KR, et al. Melanoma staging: evidence-based changes in American Joint Committee on Cancer eight edition cancer staging manual. CA Cancer J Clin 2017; 67:472-92.

19 Madu MF, Franke V, van de Weil BA, et al. External validation of the AJCC 8th edition melanoma staging system: who needs the adjuvant treatment? Proceedings of the ASCO Annual Meeting; 2019 May 30 - June 3; Chicago, IL.

20 Weber J, Mandala M, Del Vecchio M, et al. Adjuvant nivolumab versus ipilimumab in resected Stage III or IV melanoma. N Engl J Med 2017; 377:1824–35.

21 Weber JS, Del Vecchio M, Mandala M, et al. Adjuvant nivolumab (NIVO) versus ipilimumab (IPI) in resected stage III/IV melanoma: 3-year efficacy and biomarker results from the phase III CheckMate 238 trial. Ann Oncol 2019;30(Suppl 5): 533-4.

22 Eggermont AMM, Blank CU, Mandala M, et al. Adjuvant pembrolizumab versus placebo in resected Stage III melanoma. N Engl J Med. 2018;378:1789–1801.

23 Hauschild A, Dummer R, Schadendorf D, et al. Longer follow-up confirms relapse-free survival benefit with adjuvant abrafenib plus trametinib in patients with resected BRAF V600-mutant Stage III melanoma. J Cin Onc. 2018;36:1-9.

24 Long GV, Hauschild A, Santinami M, et al. Adjuvant dabrafenib plus trametinib in Stage III BRAF-mutated melanoma. N Engl J Med. 2017;377(19):1813-23.

25 Long G. Adjuvant therapy with nivolumab combined with ipilimumab vs nivolumab alone in patients with resected stage IIIB–D/IV melanoma. Proceedings of the American Association for Cancer Research; 2021 Apr 10 - May 21.

26 He Y, Yu H, Rozeboom L, et al. LAG-3 protein expression in non-small cell lung cancer and its relationship with PD-1/PD-L1 and tumor-infiltrating lymphocytes. J Thorac Oncol 2017;12:814–23.

27 Efficacy of anti-LAG-3 antibody 19C7 in Sa1N tumor-bearing mice. (Study BDX-1408-251). Bristol Myers Squibb Company; 2013. Document Control No. 930071265.

Approved v4.0 930169531 4.0

28 Anti-tumor activity of anti-PD-1 and anti-LAG-3 antibodies alone and in combination in a SA1N fibrosarcoma tumor model (Study MDX-1106-059). Bristol Myers Squibb Company; 2013. Document Control No. 930054253.

29 Confirmation of antitumor activity of anti-LAG-3 antibody alone and in combination with anti-PD-1 antibody in a Sa1N fibrosarcoma tumor model (Study BDX-1408-224). Bristol Myers Squibb Company; 2013. Document Control No. 930071272.

30 BMS-986213: Relatlimab-nivolumab Investigator Brochure. Bristol-Myers Squibb Company; 2022. Document Control No. 930119921.

31 Lipson, EJ, Tawbi, HA, Schadendorf, D, et al. Relatlimab (RELA) plus nivolumab (NIVO) versus NIVO in first-line advanced melanoma: primary phase III results from RELATIVITY-047 (CA224-047). J Clin Oncol 2021;39(suppl; abstr9503).

32 Addendum 01 to the Primary Clinical Study Report for CA224047. A randomized, double-blind Phase 2/3 study of relatlimab combined with nivolumab versus nivolumab in participants with previously untreated metastatic or unresectable melanoma. Bristol-Myers Squibb Company; 2021. Document Control No. 930168350.

33 DOI: 10.1200/JCO.18.02464 Journal of Clinical Oncology 37, no. 25 (September 01, 2019) 2201-2205

34 Indini A, Brecht I, Del Vecchio M, et al. Cutaneous melanoma in adolescents and young adults. Pediatr Blood Cancer 2018;65:e27292.

35 The Surveillance, Epidemiology, and End Results (SEER) Carcinoma Statistics Review 2000-2005. Available from: https://seer.cancer.gov/explorer/application.html. Accessed 07-Mar-2008.

36 Brecht IB, De Paoli A, Bisogno G, et al. Pediatric patients with cutaneous melanoma: a European study. Pediatr Blood Cancer 2018;65:e26974.

37 Austin MT, Xing Y, Hayes-Jordan AA, et al. Melanoma incidence rises for children and adolescents: an epidemiologic review of pediatric melanoma in the United States. J Pediatr Surg 2013;48:2207-13.

38 Strouse JJ, Fears TR, Tucker MA, et al. Pediatric melanoma: risk factor and survival analysis of the surveillance, epidemiology and end results database. J Clin Oncol 2005;23:4735-41.

39 NCCN Guidelines Version 4.2020 Cutaneous Melanoma, NCCN.org (2020)

40 Xing Y, Bronstein Y, Ross M, et al. Contemporary diagnostic imaging modalities for the staging and surveillance of melanoma patients: a meta-analysis. Journal of the National Cancer Institute: 129-42.

41 Faries M, Thompson J, Cochran A, et al. Completion dissection or observation for sentinel-node metastasis in melanoma. New England Journal of Medicine 2017;376: 2211-22.

42 Saleh M, Javadi S, Elsherif S, et al. Multimodality imaging and genetics of primary mucosal melanomas and response to treatment [published correction appears in Radiographics 2022;42:E81]. Radiographics 2021;41:1954-72.

Approved v4.0 930169531 4.0

[43] Hamuro L, Statkevich P, Bello A, et al. Nivolumab clearance is stationary in patients with resected melanoma on adjuvant therapy: implications of disease status on time-varying clearance. Clin Pharmacol Ther 2019;106:1018-27.

[44] Zhao X, Shen J, Ivaturi V, et al. Model-based evaluation of the efficacy and safety of nivolumab once every 4 weeks across multiple tumor types. Ann Oncol 2020;31:302-9.

[45] Aaronson NK, Ahmedzai S, Bergman B, et al. The European Organization for Research and Treatment of Cancer QLQ-C30: a quality-of-life instrument for use in international clinical trials in oncology. J Natl Cancer Inst 1993;85:365-76.

[46] Pearman TP, Beaumont JL, Mroczek D, et al. Validity and usefulness of a single-item measure of patient-reported bother from side effects of cancer therapy. Cancer 2018;124:991-7.

[47] EuroQol Group. EuroQol--a new facility for the measurement of health-related quality of life. Health Policy. 1990;16:199-208.

[48] Herdman M, Gudex C, Lloyd A, et al. Development and preliminary testing of the new five-level version of EQ-5D (EQ-5D-5L). Qual Life Res 2011;20:1727-36.

[49] van Hout B, Janssen MF, Feng YS, et al. Interim scoring for the EQ-5D-5L: mapping the EQ-5D-5L to EQ-5D-3L value sets. Value Health. 2012;15:708-15.

Approved v4.0 930169531 4.0

12 APPENDICES

Approved v4.0 930169531 4.0

APPENDIX 1 ABBREVIATIONS AND TRADEMARKS

Term	Definition
ADA	anti-drug antibodies
AE(s)	adverse event(s)
AIDS	acquired immunodeficiency syndrome
AJCC	American Joint Committee on Cancer
ALP	alkaline phosphatase
ALT	alanine aminotransferase
ART	antiretroviral therapy
AST	aspartate aminotransferase
AUC(TAU)	area under the plasma concentration-time curve over dosing interval
AxMP	auxiliary medical product
BCC	basal cell carcinoma
BICR	blinded independent central review
BMS	Bristol Myers Squibb
BRAF	B-RAF proto-oncogene
BUN	blood urea nitrogen
Cavgss	steady state average concentration
CBC	complete blood count
C	cycle
CD	cluster of differentiation
CFR	Code of Federal Regulations
cHL	classical Hodgkin's lymphoma
CI	confidence interval
CIOMS	Council for International Organizations of Medical Sciences
CL	clearance
CrCl	creatinine clearance
CLNR	nonrenal clearance
CLND	complete lymph node dissection
CLss	steady-state clearance
Cmax	maximum observed concentration

Approved v4.0 930169531 4.0

Term	Definition
CNS	central nervous system
COVID-19	coronavirus disease 2019
CPK	creatine phosphokinase
CRC	colorectal cancer
CrCl	creatinine clearance
CRF	case Report Form, paper or electronic (eCRF)
CSR	clinical study report
CT	computed tomography
CTAg	clinical trial agreement
CTCAE	common terminology criteria for adverse events
ctDNA	circulating tumor deoxyribonucleic acid
CTLA	cytotoxic T-lymphocyte-associated protein 4
CV%	coefficient of variation %
D	day
DAB	dabrafenib
dL	deciliter
DILI	drug induced liver injury
DLT	dose limiting toxicity
DMC	data monitoring committee
DMFS	distant metastasis-free survival
DNA	deoxyribonucleic acid
DRESS	drug reaction with eosinophilia and systemic symptoms
eCOA	electronic clinical outcome assessments
ECG(s)	electrocardiogram(s)
ECOG	Eastern Cooperative Oncology Group
eCRF	electronic Case Report Form
EC50	half-maximal effective concentration
eg	exempli gratia (for example)
eGFR	estimated glomerular filtration rate
EOI	end of infusion

Approved v4.0 930169531 4.0

Term	Definition
EORTC QLQ-C30	European Organization for the Research and Treatment of Cancer Quality of Life Questionnaire
E-R	exposure-response
EUDAMED	European Databank on Medical Devices
FACIT-GP5	Functional Assessment of Chronic Illness Therapy-Item GP5
FDA	Food and Drug Administration
FDC	Fixed-dose combination
FDG	fluorodeoxyglucose
FFPE	formalin-fixed paraffin-embedded
FFR	Freedom From Relapse
FGL-1	fibrinogen-like protein 1
FSH	follicle stimulating hormone
fT3	free T3
fT4	free T4
FU	follow-up
GBS	Guillain-Barre Syndrome
GCP	Good Clinical Practice
GFR	glomerular filtration rate
GI	gastrointestinal
h	hour
HBsAg	hepatitis B virus surface antigen
HBV	hepatitis B virus
HCC	hepatocellular carcinoma
HCG	human chorionic gonadotropin
HCV	hepatitis C virus
H&E	hematoxylin & eosin
HIPAA	Health Insurance Portability and Accountability Act
HIV	Human Immunodeficiency Virus
HR	hazard ratio
HRQoL	health-related quality of life
HRT	hormone replacement therapy

Approved v4.0 930169531 4.0

Term	Definition
IA	interim analysis
IB	Investigator's Brochure
IC50	half-maximal inhibitory concentration
ICE	intercurrent event
ICF	informed consent form
ICH	International Conference on Harmonisation
ICMJE	International Committee of Medical Journal Editors
IDO	indoleamine 2,3-dioxygenase
ie	id est (that is)
IEC	Independent Ethics Committee
IFN-γ	Interferon-γ
IgG	immunoglobulin G
IgG4	immunoglobulin G4
IHC	immunohistochemistry
IMAE	immune-mediated adverse event
IMG	immunogenicity
IP/IMP	investigational [medicinal] products
IND	Investigational New Drug Exemption
IO	immuno-oncology
IPI	ipilimumab
IUS	intrauterine hormone-releasing system
IRB	Institutional Review Board
IRT	Interactive Response Technology
IU	International Unit
IV	intravenous
kg	kilogram
K-M	Kaplan Meier
LAG-3	lymphocyte activation gene-3
LAM	lactational amenorrhea method
LD	longest diameter

Approved v4.0 930169531 4.0

Term	Definition
LDH	lactate dehydrogenase
LN	lymph node
MedDRA	Medical Dictionary for Regulatory Activities
MEK	mitogen-activated protein kinase
mg	milligram
MG	Myasthenia Gravis
MHC	major histocompatibility complex
min	minute
mL	milliliter
mmHg	millimeters of mercury
MMIS	malignant melanoma in situ
MMR	measles, mumps, rubella
MRD	minimal residual disease
MRI	magnetic resonance imaging
MSLT-II	Multicenter Selective Lymphadenectomy Trial
MSS	melanoma specific survival rate
MTD	maximum tolerated dose
μg	microgram
N	number of subjects or observations
N/A	not applicable
NCCN	National Comprehensive Cancer Network
NCT	National clinical trial number
NED	no evidence of disease
ng	nanogram
NGS	next generation sequencing
NIMP	non-investigational medicinal products
Nivo	nivolumab
NK	natural killer
NRAS	neuroblastoma ras viral oncogene homolog
NSAID	nonsteroidal anti-inflammatory drug

Approved v4.0 930169531 4.0

Term	Definition
NSCLC	non-small cell lung cancer
OESI	other events of special interest
ORR	objective response rate
OS	overall survival
PBMC	peripheral blood mononuclear cells
PD-1	programmed death-1
PD-L1	programmed death ligand-1
PD-L2	programmed death ligand-2
PEMBRO	pembrolizumab
PET	positron emission tomography
PET-CT	positron emission tomography-computed tomography
PFS	progression-free survival
PFS2	progression-free survival 2
PK	pharmacokinetics
PPK	population pharmacokinetics
PRO	patient reported outcome
Q2W	every 2 weeks
Q3W	every 3 weeks
Q4W	every 4 weeks
QoL	quality of life
R&D	Research and Development
RCC	renal cell carcinoma
Rela	relatlimab
RFS	recurrence free survival
RNA	ribonucleic acid
RO	receptor occupancy
ROW	rest of the world
RT-PCR	reverse transcription polymerase chain reaction
SAE(s)	serious adverse event(s)
SARS-CoV-2	severe acute respiratory syndrome coronavirus 2

Approved v4.0 930169531 4.0

Term	Definition
SAP	statistical analysis plan
SAV	single agent vial
SCC	squamous cell carcinoma
SCCHN	squamous cell carcinoma of the head and neck
SJS	Stevens-Johnson syndrome
SLN	sentinel lymph nodes
SLNB	sentinel lymph node biopsy
SOA	Schedule of Activities
SPSD	Site Process and Source Documentation
SUSAR	suspected, unexpected serious adverse reaction
T3	triiodothyronine
T4	thyroxine
TB	total bilirubin
T.Bili	total bilirubin
TEN	toxic epidermal necrolysis
TMB	tumor mutational burden
TME	tumor microenvironment
TILs	tumor infiltrating lymphocytes
TnI	troponin I
TNM	tumor/node/metastasis
TnT	troponin T
TRAE	treatment-related adverse event
TRAM	trametinib
TSH	thyroid stimulating hormone
TSST	time to second subsequent therapy
tT3	total T3
tT4	total T4
ULN	upper limit of normal
USA	United States of America
USPI	United States Prescribing Information

Approved v4.0 930169531 4.0

Term	Definition
VAS	visual analog scale
Vss/F (or Vss)	apparent volume of distribution at steady state
Vz	Volume of distribution of terminal phase (if IV and if multi-exponential decline)
WES	whole exome sequencing
WOCBP	women of childbearing potential
WS	Worldwide patient safety

Approved v4.0 930169531 4.0

APPENDIX 2 STUDY GOVERNANCE CONSIDERATIONS

The terms “participant” and “subject” refer to a person who has consented to participate in the clinical research study. Typically, the term “participant” is used in the protocol and the term “subject” is used in the Case Report Form (CRF).

REGULATORY AND ETHICAL CONSIDERATIONS

This study will be conducted in accordance with:

- Consensus ethical principles derived from international guidelines, including the Declaration of Helsinki and Council for International Organizations of Medical Sciences (CIOMS) International Ethical Guidelines
- Applicable International Council for Harmonisation (ICH) Good Clinical Practice (GCP) Guidelines
- Applicable laws, regulations, and requirements

The study will be conducted in compliance with the protocol. The protocol, any revisions/amendments, and the participant informed consent form (ICF) will receive approval/favorable opinion by Institutional Review Board/Independent Ethics Committee (IRB/IEC), and regulatory authorities according to applicable regulations prior to initiation of the study.

All potential serious breaches must be reported to the Sponsor or designee immediately. A potential serious breach is defined as a Quality Issue (eg, protocol deviation) that is likely to affect, to a significant degree, one or more of the following: (1) the rights, physical safety or mental integrity of one or more participants; (2) the scientific value of the clinical trial (eg, reliability and robustness of generated data). Items (1) or (2) can be associated with either GCP regulation(s) or trial protocol(s).

Personnel involved in conducting this study will be qualified by education, training, and experience to perform their respective tasks.

This study will not use the services of study personnel where sanctions have been invoked or where there has been scientific misconduct or fraud (eg, loss of medical licensure, debarment).

INSTITUTIONAL REVIEW BOARD/INDEPENDENT ETHICS COMMITTEE

Before study initiation, the investigator must have written and dated approval/favorable opinion from the IRB/IEC for the protocol, Investigator’s Brochure, product labeling information, ICF, participant recruitment materials (eg, advertisements), and any other written information to be provided to participants.

The investigator, Sponsor, or designee should provide the IRB/IEC with reports, updates, and other information (eg, expedited safety reports, amendments, administrative letters) annually, or more frequently, in accordance with regulatory requirements or institution procedures.

The investigator is responsible for providing oversight of the conduct of the study at the site and adherence to requirements of the following where applicable:

Approved v4.0 930169531 4.0

- ICH guidelines,
- United States Code of Federal Regulations, Title 21, Part 50 (21CFR50)
- European Union Directive 2001/20/EC; or
- European Regulation 536/2014 for clinical studies (if applicable),
- European Medical Device Regulation 2017/745 for clinical device research (if applicable),
- the IRB/IEC
- and all other applicable local regulations.

COMPLIANCE WITH THE PROTOCOL AND PROTOCOL REVISIONS

The investigator should not implement any deviation or change to the protocol without prior review and documented approval/favorable opinion of an amendment from the IRB/IEC (and, if applicable, also by the local Health Authority), except where necessary to eliminate an immediate hazard(s) to study participants.

If a deviation or change to a protocol is implemented to eliminate an immediate hazard(s) prior to obtaining relevant approval/favorable opinion(s), the deviation or change will be submitted as soon as possible to:

- IRB/IEC
- Regulatory authority(ies), if applicable by local regulations (per national requirements)

Documentation of approval/favorable opinion signed by the chairperson or designee of the IRB(s)/IEC(s) and, if applicable, also by the local Health Authority, must be sent to Bristol-Myers Squibb (BMS).

If an amendment substantially alters the study design or increases the potential risk to the participant: (1) the ICF must be revised and submitted to the IRB(s)/IEC(s) for review and approval/favorable opinion; (2) the revised form must be used to obtain consent from participants currently enrolled in the study if they are affected by the amendment; and (3) the new form must be used to obtain consent from new participants prior to enrollment.

FINANCIAL DISCLOSURE

Investigators and sub-investigators will provide the Sponsor with sufficient, accurate financial information, in accordance with regulations, to allow the Sponsor to submit complete and accurate financial certification or disclosure statements to the appropriate Health Authorities. Investigators are responsible for providing information on financial interests during the course of the study and for 1 year after completion of the study.

INFORMED CONSENT PROCESS

Investigators must ensure that participants are clearly and fully informed about the purpose, potential risks, and other critical issues regarding clinical studies in which they volunteer to participate.

Approved v4.0 930169531 4.0

The Sponsor or designee will provide the investigator with an appropriate sample ICF, which will include all elements required by the ICH GCP, and applicable regulatory requirements. The sample ICF will adhere to the ethical principles that have their origin in the Declaration of Helsinki.

The investigator or his/her representative must:

- Obtain IRB/IEC written approval/favorable opinion of the written ICF and any other information to be provided to the participant prior to the beginning of the study and after any revisions are completed for new information.
- Provide a copy of the ICF and written information about the study in the language in which the participant is proficient prior to clinical study participation. The language must be nontechnical and easily understood.
- Explain the nature of the study to the participant or his/her legally acceptable representative and answer all questions regarding the study.
- Inform participant that his/her participation is voluntary. Participant or his/her legally acceptable representative will be required to sign a statement of informed consent that meets the requirements of 21 CFR 50, local regulations, ICH guidelines, Health Insurance Portability and Accountability Act (HIPAA) requirements, where applicable, and the IRB/IEC or study center.
- Allow time necessary for participant or his/her legally acceptable representative to inquire about the details of the study.

Obtain an ICF signed and personally dated by participant or his/her legally acceptable representative and by the person who conducted the informed consent discussion.

- Include a statement in participant's medical record that written informed consent was obtained before participant was enrolled in the study and the date the written consent was obtained. The authorized person obtaining the informed consent must also sign the ICF.
- Re-consent participant to the most current version of the ICF(s) during his/her participation in the study, as applicable.

Revise the ICF whenever important new information becomes available that is relevant to the participant's consent. The investigator, or a person designated by the investigator, should fully inform the participant or his/her legally acceptable representativeof all pertinent aspects of the study and of any new information relevant to the participant's willingness to continue participation in the study. This communication should be documented.

The confidentiality of records that could identify participants must be protected, respecting the privacy and confidentiality rules applicable to regulatory requirements, the participant's signed ICF, and, in the US, the participant's signed HIPAA Authorization.

The ICF must also include a statement that BMS and local and foreign regulatory authorities have direct access to participant records.

Approved v4.0 930169531 4.0

In situations where consent cannot be given by participants, their legally acceptable representatives (per country regulation) are clearly and fully informed about the purpose, potential risks, and other critical issues regarding clinical studies in which the participant volunteers to participate.

If informed consent is initially given by a participant's legally acceptable representative or legal guardian and the participant subsequently becomes capable of making and communicating his or her informed consent during the study, consent must additionally be obtained from the participant.

For minors, according to local legislation, one or both parents or a legally acceptable representative must be informed of the study procedures and must sign the ICF approved for the study prior to clinical study participation. The explicit wish of a minor, who is capable of forming an opinion and assessing this information to refuse participation in, or to be withdrawn from, the clinical study at any time, should be considered by the investigator.

Minors who are judged to be of an age of reason must also give their written consent. Minors who reach the age of majority (legal adulthood) during the clinical study must give their written consent.

The rights, safety, and well-being of the study participants are the most important considerations and should prevail over interests of science and society.

BMS COMMITMENT TO DIVERSITY IN CLINICAL TRIALS

The mission of BMS is to transform patients' lives through science by discovering, developing, and delivering innovative medicines that help them prevail over serious diseases.

BMS is committed to doing its part to ensure that patients have a fair and just opportunity to achieve optimal health outcomes.

BMS is working to improve the recruitment of a diverse participant population with the goal that the clinical trial becomes more reflective of the real-world population and the people impacted by the diseases studied.

DATA PROTECTION, DATA PRIVACY, AND DATA SECURITY

BMS collects and processes personal data of study participants, patients, health care providers, and researchers for biopharmaceutical research and development to advance innovative, high-quality medicines that address the medical needs of patients. BMS ensures the privacy, protection, and confidentiality of such personal data to comply with applicable laws. To achieve these goals, BMS has internal policies that indicate measures and controls for processing personal data. BMS adheres to these standards to ensure that collection and processing of personal data are limited and proportionate to the purpose for which BMS collects such personal data. This purpose is clearly and unambiguously notified to the individual at the time of collection of personal data. In the true spirit of science, BMS is dedicated to sharing clinical trial information and data with participants, medical/research communities, the media, policy makers, and the general public. This is done in a manner that safeguards participant privacy and informed consent while respecting the integrity of national regulatory systems. Clinical trial data, health-related research, and pharmacovigilance activities on key-coded health data transferred by BMS across national borders is done in compliance with the relevant data protection laws in the country and GCP requirements.

Approved v4.0 930169531 4.0

BMS protects Personal Information with adequate and appropriate security controls as indicated under the data protection laws. To align with the recommended security standards, BMS has adopted internal security standards and policies to protect personal data at every stage of its processing.

To supplement these standards, BMS enters into Clinical Trial Agreements (CTAgs) with confidentiality obligations to ensure proper handling and protection of personal data by third parties accessing and handling personal data.

BMS takes unauthorized access and disclosure of Personal Information very seriously. BMS has adopted the security standards that include National Institute of Standards and Technology Cybersecurity Framework for studies in the US. BMS aligns with these standards to continuously assess and improve its ability to protect, detect, and respond to cyber attacks and other unauthorized attempts to access personal data. These standards also aid in mitigating possible adverse effects. Furthermore, BMS Information Technology has defined 6 principles to protect our digital resources and information:

1) Responsibilities of IT Personnel
2) Securing the BMS Digital Infrastructure
3) Identity and Access Management
4) External Partner Connections
5) Cyber Threat Detection and Response
6) Internal Cyber Incident Investigation

SOURCE DOCUMENTS

Source documents provide evidence for the existence of the participant and substantiate the integrity of the data collected. Source documents are filed at the investigator's site.

Data reported on the CRF or entered in the electronic CRF (eCRF) that are transcribed from source documents must be consistent with the source documents or the discrepancies must be explained.

- The investigator may need to request previous medical records or transfer records, depending on the study. Also, current medical records must be available.
- Definitions of what constitutes source data can be found in the Site Process and Source Documentation (SPSD) form.

The investigator is responsible for ensuring that the source data are accurate, legible, contemporaneous, original, and attributable, whether the data are handwritten on paper or entered electronically. If source data are created (first entered), modified, maintained, archived, retrieved, or transmitted electronically via computerized systems (and/or any other kind of electronic devices) as part of regulated clinical trial activities, such systems must be compliant with all applicable laws and regulations governing use of electronic records and/or electronic signatures. Such systems may include, but are not limited to, electronic medical records/electronic health

Approved v4.0 930169531 4.0

records, adverse event (AE) tracking/reporting, protocol-required assessments, and/or drug accountability records.

When paper records from such systems are used in place of an electronic format to perform regulated activities, such paper records should be certified copies. A certified copy consists of a copy of original information that has been verified, as indicated by a dated signature, as an exact copy having all of the same attributes and information as the original.

STUDY INTERVENTION RECORDS

Records for study intervention (whether supplied by BMS, its vendors, or the site) must substantiate study intervention integrity and traceability from receipt, preparation, administration, and through destruction or return. Records must be made available for review at the request of BMS/designee or a Health Authority.

If	Then
Supplied by BMS (or its vendors):	Records or logs must comply with applicable regulations and guidelines and should include:<br>• amount received and placed in storage area<br>• amount currently in storage area<br>• label identification number or batch number<br>• amount dispensed to and returned by each participant, including unique participant identifiers<br>• amount transferred to another area/site for dispensing or storage<br>• nonstudy disposition (eg, lost, wasted)<br>• amount destroyed at study site, if applicable<br>• amount returned to BMS<br>• retain samples for bioavailability/bioequivalence/biocomparability, if applicable<br>• dates and initials of person responsible for Investigational Product dispensing / accountability, as per the Delegation of Authority Form
Sourced by site and not supplied by BMS or its vendors (examples include IP sourced from the sites stock or commercial supply or a specialty pharmacy)	The investigator or designee accepts responsibility for documenting traceability and study treatment integrity in accordance with requirements applicable under law and the standard operating procedures/standards of the sourcing pharmacy

BMS or its designee will provide forms to facilitate inventory control if the investigational site does not have an established system that meets these requirements.

Approved v4.0 930169531 4.0

CASE REPORT FORMS

An investigator is required to prepare and maintain adequate and accurate case histories designed to record all observations and other data pertinent to the investigation on each individual treated or entered as a control in the investigation. Data that are derived from source documents and reported on the CRF must be consistent with the source documents, or the discrepancies must be explained. Additional clinical information may be collected and analyzed in an effort to enhance understanding of product safety. CRFs may be requested for AEs and/or laboratory abnormalities that are reported or identified during the course of the study.

For sites using the Sponsor or designee electronic data capture (EDC) tool, eCRFs will be prepared for all data collection fields except for fields specific to SAEs and pregnancy, which will be reported on the electronic SAE form and Pregnancy Surveillance Form, respectively. If the electronic SAE form is not available, a paper SAE form can be used. Spaces may be left blank only in those circumstances permitted by study-specific CRF completion guidelines provided by the Sponsor or designee.

The confidentiality of records that could identify participants must be protected, respecting the privacy and confidentiality rules in accordance with the applicable regulatory requirement(s).

The investigator will maintain a signature sheet to document signatures and initials of all persons authorized to make entries and/or corrections on CRFs.

The completed CRF and SAE/pregnancy CRFs must be promptly reviewed, signed, and dated by the investigator or qualified physician who is a sub-investigator and who is delegated this task on the Delegation of Authority Form. Sub-investigators in Japan may not be delegated the CRF approval task. For eCRFs, review and approval/signature is completed electronically through the BMS EDC tool. The investigator must retain a copy of the CRFs, including records of the changes and corrections.

Each individual electronically signing eCRFs must meet Sponsor or designee training requirements and must only access the BMS EDC tool using the unique user account provided by the Sponsor or designee. User accounts are not to be shared or reassigned to other individuals.

MONITORING

Monitoring details describing strategy, including definition of study critical data items and processes (eg, risk-based initiatives in operations and quality such as risk management and mitigation strategies and analytical risk-based monitoring), methods, responsibilities, and requirements, including handling of noncompliance issues and monitoring techniques (central, remote, or on-site monitoring) are provided in the monitoring plan.

Representatives of BMS must be allowed to visit all study site locations periodically to assess the data quality and study integrity. On site, they will review study records and directly compare them with source documents, discuss the conduct of the study with the investigator, and verify that the facilities remain acceptable. Certain CRF pages and/or electronic files may serve as the source documents.

Approved v4.0 930169531 4.0

In addition, the study may be evaluated by the Sponsor or designee internal auditors and government inspectors who must be allowed access to CRFs, source documents, other study files, and study facilities. BMS audit reports will be kept confidential.

The investigator must notify BMS promptly of any inspections scheduled by regulatory authorities and promptly forward copies of inspection reports to the Sponsor or designee.

RECORDS RETENTION

The investigator (or head of the study site in Japan) must retain all study records and source documents for the maximum period required by applicable regulations and guidelines, or institution procedures, or for the period specified by BMS or its designee, whichever is longer. The investigator (or head of the study site in Japan) must contact BMS prior to destroying any records associated with the study.

BMS or its designee will notify the investigator (or head of the study site in Japan) when the study records are no longer needed.

If the investigator withdraws from the study (eg, relocation, retirement), the records shall be transferred to a mutually agreed-upon designee (eg, another investigator, study site, IRB). Notice of such transfer will be given in writing to BMS or its designee.

RETURN OF STUDY TREATMENT

For this study, study treatments (those supplied by BMS or a vendor or sourced by the investigator), such as partially used study treatment containers, vials, and syringes, may be destroyed on site.

If	Then
Study treatments supplied by BMS (including its vendors)	Any unused study interventions supplied by BMS can only be destroyed after being inspected and reconciled by the responsible Study Monitor, unless study treatments containers must be immediately destroyed as required for safety, or to meet local regulations (eg, cytotoxics or biologics).<br><br>Partially used study interventions and/or empty containers may be destroyed after proper reconciliation and documentation. But unused IMP must be reconciled by site monitor/Clinical Research Associate prior to destruction.<br><br>If study treatments will be returned, the return will be arranged by the responsible Study Monitor.

Approved v4.0 930169531 4.0

Study treatments sourced by site, not supplied by BMS (or its vendors; eg, study treatments sourced from the site's stock or commercial supply or a specialty pharmacy)	It is the investigator's or designee's responsibility to dispose of all containers according to the institutional guidelines and procedures.

It is the investigator's or designee's responsibility to arrange for disposal of study interventions, provided that procedures for proper disposal have been established according to applicable federal, state, local, and institutional guidelines and procedures, and provided that appropriate records of disposal are kept. The following minimal standards must be met:

- On-site disposal practices must not expose humans to risks from the drug.
- On-site disposal practices and procedures are in agreement with applicable laws and regulations, including any special requirements for controlled or hazardous substances.
- Written procedures for on-site disposal are available and followed. The procedures must be filed with the site's standard operating procedures and a copy provided to BMS upon request.
- Records are maintained that allow for traceability of each container, including the date disposed of, quantity disposed, and identification of the person disposing the containers. The method of disposal (eg, incinerator, licensed sanitary landfill, or licensed waste-disposal vendor) must be documented.
- Accountability and disposal records are complete, up-to-date, and available for the Study Monitor to review throughout the clinical trial period.

It is the investigator's or designee's responsibility to arrange for disposal of all empty containers.

If conditions for destruction cannot be met, the responsible Study Monitor will make arrangements for return of study treatments provided by BMS (or its vendors). Destruction of non-study treatments sourced by the site, not supplied by BMS, is solely the responsibility of the investigator or designee.

STUDY AND SITE START AND CLOSURE

The Sponsor/designee reserves the right to close the study site or to terminate the study at any time for any reason at the sole discretion of the Sponsor. Study sites will be closed upon study completion. A study site is considered closed when all required documents and study supplies have been collected and a study-site closure visit has been performed.

The investigator may initiate study-site closure at any time, provided there is reasonable cause and sufficient notice is given in advance of the intended termination.

Reasons for the early closure of a study site by the Sponsor or investigator may include, but are not limited to:

- Failure of the investigator to comply with the protocol, the requirements of the IRB/IEC or local Health Authorities, the Sponsor's procedures, or GCP guidelines
- Inadequate recruitment of participants by the investigator

Approved v4.0 930169531 4.0

- Discontinuation of further study intervention development

If the study is prematurely terminated or suspended, the Sponsor shall promptly inform the investigators, the IECs/IRBs, the regulatory authorities, and any contract research organization(s) used in the study of the reason for termination or suspension, as specified by the applicable regulatory requirements. The investigator shall promptly inform the participant and should assure appropriate participant therapy and/or follow-up.

DISSEMINATION OF CLINICAL STUDY DATA

In order to benefit potential study participants, patients, healthcare providers and researchers, and to help BMS honor its commitments to study participants, BMS will make information about clinical research studies and a summary of their results available to the public as per regulatory and BMS requirements. BMS will post study information on local, national or regional databases in compliance with national and international standards for disclosure. BMS may also voluntarily disclose information to applicable databases.

CLINICAL STUDY REPORT

A Signatory Investigator must be selected to sign the Clinical Study Report (CSR).

For each CSR related to this protocol, the following criteria will be used to select the Signatory Investigator:

- Participant recruitment (eg, among the top quartile of enrollers)
- External Principal Investigator designated at protocol development
- National Coordinating Investigator
- Study Steering Committee chair or their designee
- Involvement in trial design
- Regional representation (eg, among top quartile of enrollers from a specified region or country)
- Other criteria (as determined by the study team)

SCIENTIFIC PUBLICATIONS

The data collected during this study are confidential and proprietary to the Sponsor or designee. Any publications or abstracts arising from this study must adhere to the publication requirements set forth in the Clinical Trial Agreement (CTAg) governing [study site or investigator] participation in the study. These requirements include, but are not limited to, submitting proposed publications to the Sponsor or designee at the earliest practicable time prior to submission or presentation and otherwise within the time period set forth in the CTAg.

Scientific publications (such as abstracts, congress podium presentations and posters, and manuscripts) of the study results will be a collaborative effort between the study Sponsor and the external authors. No public presentation or publication of any interim results may be made by any Principal Investigator, sub-investigator, or any other member of the study staff without the prior written consent of the Sponsor.

Approved v4.0 930169531 4.0

Authorship of publications at BMS is aligned with the criteria of the International Committee of Medical Journal Editors (ICMJE, www.icmje.org). Authorship selection is based upon significant contributions to the study (ie, ICMJE criterion #1). Authors must meet all 4 ICMJE criteria for authorship:

1) Substantial intellectual contribution to the conception or design of the work; or the acquisition of data (ie, evaluable participants with quality data), analysis, or interpretation of data for the work (eg, problem solving, advice, evaluation, insights and conclusion); AND
2) Drafting the work or revising it critically for important intellectual content; AND
3) Final approval of the version to be published; AND
4) Agreement to be accountable for all aspects of the work in ensuring that questions related to the accuracy or integrity of any part of the work are appropriately investigated and resolved.

Those who make the most significant contributions, as defined above, will be considered by BMS for authorship of the primary publication. Sub-investigators will generally not be considered for authorship in the primary publication. Geographic representation will also be considered.

Authors will be listed by order of significant contributions (highest to lowest), with the exception of the last author. Authors in first and last position have provided the most significant contributions to the work.

For secondary analyses and related publications, author list and author order may vary from primary to reflect additional contributions.

Approved v4.0 930169531 4.0

APPENDIX 3 ADVERSE EVENTS AND SERIOUS ADVERSE EVENTS: DEFINITIONS AND PROCEDURES FOR RECORDING, EVALUATING, FOLLOW-UP, AND REPORTING

ADVERSE EVENTS

**Adverse Event Definition:**
An adverse event (AE) is defined as any new untoward medical occurrence or worsening of a pre-existing medical condition in a clinical investigation participant administered study treatment that does not necessarily have a causal relationship with this treatment.
An AE can therefore be any unfavorable and unintended sign (such as an abnormal laboratory finding), symptom, or disease temporally associated with the use of study treatment, whether or not considered related to the study treatment.
**Events <u>Meeting</u> the AE Definition**
• Any abnormal laboratory test results (hematology, clinical chemistry, or urinalysis) or results from other safety assessments (eg, electrocardiograms, radiological scans, vital signs measurements), including those that worsen from baseline, considered clinically significant in the medical and scientific judgment of the investigator. Note that abnormal lab tests or other safety assessments should only be reported as AEs if the final diagnosis is not available. Once the final diagnosis is known, the reported term should be updated to be the diagnosis.<br>• Exacerbation of a chronic or intermittent pre-existing condition, including either an increase in frequency and/or intensity of the condition.<br>• New conditions detected or diagnosed after study intervention administration, even though it may have been present before the start of the study.<br>• Signs, symptoms, or the clinical sequelae of a suspected drug-drug interaction.<br>• Signs, symptoms, or the clinical sequelae of a suspected overdose of either study intervention or a concomitant medication. Overdose, as a verbatim term (as reported by the investigator), should not be reported as an AE/serious adverse event (SAE) unless it is an intentional overdose taken with possible suicidal/self-harming intent. Such overdoses should be reported regardless of sequelae and should specify “intentional overdose” as the verbatim term.
**Events <u>NOT</u> Meeting the AE Definition**
• Medical or surgical procedure (eg, endoscopy, appendectomy); the condition that leads to the procedure is the AE.<br>• Situations in which an untoward medical occurrence did not occur (social and/or convenience admission to a hospital).

DEFINITION OF SAE

If an event is not an AE per definition above, then it cannot be an SAE, even if serious conditions are met.

Approved v4.0 930169531 4.0

SERIOUS ADVERSE EVENTS

**A serious adverse event (SAE) is defined as any untoward medical occurrence that, at any dose:**
Results in death.
Is life-threatening (defined as an event in which the participant was at risk of death at the time of the event; it does not refer to an event which hypothetically might have caused death if it were more severe).
Requires inpatient hospitalization or causes prolongation of existing hospitalization (see NOTE below).<br><br>NOTE:<br><br>The following hospitalizations are not considered SAEs in Bristol-Myers Squibb (BMS) clinical studies:<br><br>• A visit to the emergency room or other hospital department < 24 hours that does not result in admission (unless considered an important medical or life-threatening event).<br>• Elective surgery, planned prior to signing consent.<br>• Admissions as per protocol for a planned medical/surgical procedure.<br>• Routine health assessment requiring admission for baseline/trending of health status (eg, routine colonoscopy).<br>• Medical/surgical admission other than to remedy ill health and planned prior to entry into the study. Appropriate documentation is required in these cases.<br>• Admission encountered for another life circumstance that carries no bearing on health status and requires no medical/surgical intervention (eg, lack of housing, economic inadequacy, caregiver respite, family circumstances, administrative reason).<br>• Admission for administration of anticancer therapy in the absence of any other SAEs (applies to oncology protocols).
Results in persistent or significant disability/incapacity.
Is a congenital anomaly/birth defect.
Is an important medical event (defined as a medical event[s] that may not be immediately life-threatening or result in death or hospitalization but, based upon appropriate medical and scientific judgment, may jeopardize the participant or may require intervention [eg, medical, surgical] to prevent one of the other serious outcomes listed in the definition above). Examples of such events include, but are not limited to, intensive treatment in an emergency room or at home for allergic bronchospasm and blood dyscrasias or convulsions that do not result in hospitalization. Potential drug-induced liver injury (DILI) is also considered an important medical event. (See Section 9.2.7 for the definition of potential DILI.)

Pregnancy and DILI must follow the same transmission timing and processes to BMS as used for SAEs. (See Section 9.2.5 for reporting pregnancies.)

EVALUATING AES AND SAES

**Assessment of Causality**
• The investigator is obligated to assess the relationship between study intervention and each occurrence of each AE/SAE.<br>• A "reasonable possibility" of a relationship conveys that there are facts, evidence, and/or arguments to suggest a causal relationship, rather than a relationship cannot be ruled out.<br>• The investigator will use clinical judgment to determine the relationship.<br>• Alternative causes, such as underlying disease(s), concomitant therapy, and other risk factors, as well as the temporal relationship of the event to study intervention administration, will be considered and investigated.<br>• The investigator will also consult the Investigator's Brochure and/or product information for marketed products in his/her assessment.<br>• For each AE/SAE, the investigator must document in the medical notes that he/she has reviewed the AE/SAE and has provided an assessment of causality.<br>• There may be situations in which an SAE has occurred and the investigator has minimal information to include in the initial report to the Sponsor. However, it is very important that the investigator always make an assessment of causality for every event before the initial transmission of the SAE data to the Sponsor.<br>• The investigator may change his/her opinion of causality in light of follow-up information and send an SAE follow-up report with the updated causality assessment.<br>• The causality assessment is one of the criteria used when determining regulatory reporting requirements.

**Follow-up of AEs and SAEs**
If only limited information is initially available, follow-up reports are required. (Note: Follow-up SAE reports must include the same investigator term[s] initially reported.)<br><br>If an ongoing SAE changes in its intensity or relationship to study treatment or if new information becomes available, the SAE report must be updated and submitted within 24 hours to BMS (or designee) using the same procedure used for transmitting the initial SAE report.<br><br>All SAEs must be followed to resolution or stabilization.

Approved v4.0 930169531 4.0

REPORTING OF SAES TO SPONSOR OR DESIGNEE

- SAEs, whether related or not related to study treatment, and pregnancies must be reported to BMS (or designee) immediately within 24 hours of awareness of the event.
- SAEs must be recorded on the SAE Report Form.
  - The required method for SAE data reporting is through the electronic case report form (eCRF).
  - The paper SAE Report Form is intended only as a back-up option when the electronic data capture system is unavailable/not functioning for transmission of the eCRF to BMS (or designee).
    - ♦ In this case, the paper form is transmitted via email or confirmed facsimile transmission.
    - ♦ When paper forms are used, the original paper forms are to remain on site.
- Pregnancies must be recorded on paper Pregnancy Surveillance Forms and transmitted via email or confirmed facsimile transmission.

SAE Email Address: worldwide.safety@BMS.com

SAE Facsimile Number: *Will be provided by local site monitor.*

SAE Telephone Contact (required for SAE and pregnancy reporting): *Will be provided by local site monitor.*

Approved v4.0 930169531 4.0

APPENDIX 4 WOMEN OF CHILDBEARING POTENTIAL DEFINITIONS AND METHODS OF CONTRACEPTION

Appendix 4 provides general information and definitions related to Woman of Childbearing Potential and methods of contraception that can be applied to most clinical trials. For information specific to this study regarding acceptable contraception requirements for female and male participants, refer to Section 6.1 of the protocol. Only the contraception methods as described in Section 6.1 are acceptable for this study.

DEFINITIONS

Woman of Childbearing Potential (WOCBP)

A woman is considered fertile following menarche and until becoming postmenopausal unless permanently sterile. Permanent sterilization methods include hysterectomy, bilateral salpingectomy, and bilateral oophorectomy.

Women in the following categories are not considered WOCBP:

- Premenarchal
- Premenopausal female with 1 of the following:
  - Documented hysterectomy
  - Documented bilateral salpingectomy
  - Documented bilateral oophorectomy

Note: Documentation can come from the site personnel's review of the participant's medical records, medical examination, or medical history interview.

- Postmenopausal female
  - A postmenopausal state is defined as 12 months of amenorrhea in a woman over age 45 years in the absence of other biological or physiological causes. In addition, females under the age of 55 years must have a serum follicle-stimulating hormone (FSH) level > 40 mIU/mL to confirm menopause.

Note: Females treated with hormone replacement therapy (HRT) are likely to have artificially suppressed FSH levels and may require a washout period in order to obtain a physiologic FSH level. The duration of the washout period is a function of the type of HRT used. Suggested guidelines for the duration of the washout periods for HRT types are presented below. Investigators should use their judgement in checking serum FSH levels.

- 1-week minimum for vaginal hormonal products (rings, creams, gels)
- 4-week minimum for transdermal products
- 8-week minimum for oral products

Approved v4.0 930169531 4.0

Other parenteral products may require washout periods as long as 6 months. If the serum FSH level is > 40 mIU/mL at any time during the washout period, the woman can be considered postmenopausal.

End of Relevant Systemic Exposure

End of relevant systemic exposure is the timepoint where the Investigational Medicinal Product (IMP) or any active major metabolites have decreased to a concentration that is no longer considered to be relevant for human teratogenicity or fetotoxicity. This should be evaluated in context of safety margins from the no-observed-adverse-effect level or the time required for 5 half-lives of the IMP to pass.

METHODS OF CONTRACEPTION

Local laws and regulations may require use of alternative and/or additional contraception methods.

**Highly Effective Contraceptive Methods That Are User Dependent**<br>*Failure rate of < 1% per year when used consistently and correctly.*[a]
• Combined (estrogen- and progestogen-containing) hormonal contraception associated with inhibition of ovulation and/or implantation. (This method of contraception can only be used by WOCBP participants in studies where hormonal contraception is permitted by the study protocol.)[b]<br>– Oral (birth control pills)<br>– Intravaginal (rings)<br>– Transdermal<br>• Combined (estrogen-and progestogen-containing) hormonal contraception must begin at least 30 days prior to initiation of study therapy.
• Progestogen-only hormonal contraception associated with inhibition of ovulation. (This method of contraception can only be used by WOCBP participants in studies where hormonal contraception is permitted by the study protocol.)[b]<br>– Oral<br>– Injectable<br>• Progestogen-only hormonal contraception must begin at least 30 days prior to initiation of study therapy.
**Highly Effective Methods That Are User Independent**
• Implantable progestogen-only hormonal contraception associated with inhibition of ovulation and/or implantation. (This method of contraception can only be used by WOCBP participants in studies where hormonal contraception is permitted by the study protocol.)[b]<br>• Intrauterine device.

Approved v4.0 930169531 4.0

• Intrauterine hormone-releasing system (IUS). (This method of contraception can only be used by WOCBP participants in studies where hormonal contraception is permitted by the study protocol.)[b,c]<br>• Bilateral tubal occlusion.
• Vasectomized partner<br>Having a vasectomized partner is a highly effective contraception method provided that the partner is the sole male sexual partner of the WOCBP and the absence of sperm has been confirmed. If not, an additional highly effective method of contraception should be used.A vasectomy is a highly effective contraception method provided that the participant is the sole male sexual partner of the WOCBP and the absence of sperm has been confirmed. If not, an additional highly effective method of contraception should be used.
• Sexual abstinence<br>*Sexual abstinence is considered a highly effective method only if defined as refraining from heterosexual intercourse during the entire period of risk associated with the study treatment. The reliability of sexual abstinence needs to be evaluated in relation to the duration of the study and the preferred and usual lifestyle of the participant.*<br>• Continuous abstinence must begin at least 30 days prior to initiation of study therapy.<br>• It is not necessary to use any other method of contraception when complete abstinence is elected.<br>• WOCBP participants who choose complete abstinence must continue to have pregnancy tests, as specified in Section 2.<br>• Acceptable alternate methods of highly effective contraception must be discussed in the event that the WOCBP participant chooses to forego complete abstinence.<br>• Periodic abstinence (including, but not limited to, calendar, symptothermal, postovulation methods), withdrawal (coitus interruptus), spermicides only, and lactational amenorrhea method (LAM) are not acceptable methods of contraception for this study.
NOTES:<br>[a] Typical use failure rates may differ from failure rates when contraceptive methods are used consistently and correctly. Use should be consistent with local regulations regarding the use of contraceptive methods for participants in clinical studies.<br>[b] Hormonal contraception may be susceptible to interaction with the study treatment, which may reduce the efficacy of the contraceptive method. Hormonal contraception is permissible only when there is sufficient evidence that the IMP and other study medications will not alter hormonal exposures such that contraception would be ineffective or result in increased exposures that could be potentially hazardous. In this case, alternative methods of contraception should be utilized. For information specific to this study regarding permissibility of hormonal contraception, refer to Sections 6.1 INCLUSION CRITERIA and 7.7.1 PROHIBITED AND/OR RESTRICTED TREATMENTS of the protocol.<br>[c] IUSs are acceptable methods of contraception in the absence of definitive drug interaction studies when hormone exposures from intrauterine devices do not alter contraception effectiveness. For information specific to this study regarding permissibility of hormonal contraception, refer to Sections 6.1 INCLUSION CRITERIA and 7.7.1 PROHIBITED AND/OR RESTRICTED TREATMENTS of the protocol.

Approved v4.0 930169531 4.0

**Less Than Highly Effective Contraceptive Methods That Are User Dependent**<br>*Failure rate of > 1% per year when used consistently and correctly.*
• Male or female condom with or without spermicide. Male and female condoms cannot be used simultaneously.<br>• Diaphragm with spermicide.<br>• Cervical cap with spermicide.<br>• Vaginal sponge with spermicide.<br>• Progestogen-only oral hormonal contraception, where inhibition of ovulation is not the primary mechanism of action. (This method of contraception cannot be used by WOCBP participants in studies where hormonal contraception is prohibited.)
**Unacceptable Methods of Contraception**
• Periodic abstinence (calendar, symptothermal, postovulation methods).<br>• Withdrawal (coitus interruptus).<br>• Spermicide only.<br>• LAM.

COLLECTION OF PREGNANCY INFORMATION

Guidance for collection of pregnancy information and outcome of pregnancy on the Pregnancy Surveillance Form is provided in Section 9.2.5 and Appendix 3.

Approved v4.0 930169531 4.0

APPENDIX 5 MANAGEMENT ALGORITHMS FOR STUDIES UNDER CTCAE VERSION 5.0

These general guidelines constitute guidance to the Investigator and may be supplemented by discussions with the Medical Monitor representing the Sponsor. The guidance applies to all immuno-oncology agents and regimens.

A general principle is that differential diagnoses should be diligently evaluated according to standard medical practice. Non-inflammatory etiologies should be considered and appropriately treated.

Corticosteroids are a primary therapy for immuno-oncology drug-related adverse events. The oral equivalent of the recommended IV doses may be considered for ambulatory patients with low-grade toxicity. The lower bioavailability of oral corticosteroids should be taken into account when switching to the equivalent dose of oral corticosteroids.

Consultation with a medical or surgical specialist, especially prior to an invasive diagnostic or procedure, is recommended.

The frequency and severity of the related adverse events covered by these algorithms will depend on the immuno-oncology agent or regimen being used.

Approved v4.0 930169531 4.0

GI Adverse Event Management Algorithm

Rule out non-inflammatory causes. If non-inflammatory cause is identified, treat accordingly and continue I-O therapy.
Opiates/narcotics may mask symptoms of perforation. Infliximab should not be used in cases of perforation or sepsis.

Grade of Diarrhea/ Colitis (NCI CTCAE v5)	Management	Follow-up
**Grade 1**<br>Diarrhea: < 4 stools/day over baseline<br>Colitis: asymptomatic	• Continue I-O therapy per protocol<br>• Symptomatic treatment	• Close monitoring for worsening symptoms<br>• Educate patient to report worsening immediately<br>If worsens:<br>• Treat as Grade 2 or Grade 3-4
**Grade 2**<br>Diarrhea: 4-6 stools per day over baseline;<br>IV fluids indicated < 24 hrs;<br>Limiting instrumental ADL<br>Colitis: abdominal pain; blood in stool	• Delay I-O therapy per protocol<br>• Symptomatic treatment	If improves to Grade 1:<br>• Resume I-O therapy per protocol<br>If persists > 5-7 days or recurs:<br>• 0.5-1 mg/kg/day methylprednisolone or oral equivalent<br>• When symptoms improve to Grade 1, taper steroids over at least 1 month, consider prophylactic antibiotics for opportunistic infections, and resume I-O therapy per protocol.<br>If worsens or persists > 3-5 days with oral steroids:<br>• Treat as Grade 3-4
**Grade 3-4**<br>Diarrhea (G3): ≥ 7 stools per day over baseline;<br>IV fluids ≥24 hrs;<br>limiting self care ADL<br>Colitis (G3): severe abdominal pain, peritoneal signs<br>G4: life-threatening, perforation	• Discontinue or delay I-O therapy per protocol*<br>• 1 to 2 mg/kg/day methylprednisolone IV or IV equivalent<br>• Add prophylactic antibiotics for opportunistic infections<br>• Consider lower endoscopy	If improves:<br>• Continue steroids until grade 1, then taper over at least 1 month<br>If persists > 3-5 days, or recurs after improvement:<br>• Add infliximab 5 mg/kg (if no contraindication) or other immunosuppressants.<br>• Consider testing CMV for steroid refractory colitis<br>Note: Infliximab should not be used in cases of perforation or sepsis

Patients on IV steroids may be switched to an equivalent dose of oral corticosteroids (eg, prednisone) at start of tapering or earlier, after sustained clinical improvement is observed. Lower bioavailability of oral corticosteroids should be taken into account when switching to the equivalent dose of oral corticosteroids.

* Discontinue for Grade 4 diarrhea or colitis. For Grade 3 diarrhea or colitis, 1) Nivolumab monotherapy: Nivolumab can be delayed. 2) Nivolumab+ Ipilimumab combination: Ipilimumab should be discontinued while nivolumab can be delayed. Nivolumab monotherapy can be resumed when symptoms improve to Grade 1. Please refer to protocol for dose delay and discontinue criteria for other combinations.

28-Sep-2020

Renal Adverse Event Management Algorithm

Rule out non-inflammatory causes. If non-inflammatory cause, treat accordingly and continue I-O therapy.

Grade of Creatinine Elevation (NCI CTCAE v5)	Management	Follow-up
**Grade 1**<br>Creatinine > ULN to ≤ 1.5x ULN	• Continue I-O therapy per protocol<br>• Monitor creatinine weekly	<u>If returns to baseline</u>:<br>•Resume routine creatinine monitoring per protocol<br><u>If worsens:</u><br>• Treat as Grade 2 to Grade 4
**Grade 2-3**<br>Creatinine > 1.5x baseline to ≤ 6x ULN	• Delay I-O therapy per protocol<br>• Monitor creatinine every 2-3 days<br>• 0.5 to 1 mg/kg/day methylprednisolone IV or oral equivalent<br>• Consider renal biopsy with nephrology consult	<u>If returns to Grade 1:</u><br>•Taper steroids over at least 1 month, consider prophylactic antibiotics for opportunistic infections, and resume I-O therapy and routine creatinine monitoring per protocol<br><u>If elevations persist > 7 days or worsen:</u><br>•Treat as Grade 4
**Grade 4**<br>Creatinine > 6x ULN	• Discontinue I-O therapy per protocol<br>• Monitor creatinine daily<br>• 1-2 mg/kg/day methylprednisolone IV or IV equivalent<br>• Consult nephrologist<br>• Consider renal biopsy	<u>If returns to Grade 1</u>: Taper steroids over at least 1 month and add prophylactic antibiotics for opportunistic infections<br><br><u>If no improvement or worsening</u>, add additional immunosuppression.

Patients on IV steroids may be switched to an equivalent dose of oral corticosteroids (eg, prednisone) at start of tapering or earlier, after sustained clinical improvement is observed. Lower bioavailability of oral corticosteroids should be taken into account when switching to the equivalent dose of oral corticosteroids.

28-Sep-2020

Approved v4.0 930169531 4.0

Pulmonary Adverse Event Management Algorithm

Rule out non-inflammatory causes. If non-inflammatory cause, treat accordingly and continue I-O therapy.
Evaluate with imaging and pulmonary consultation.

Grade of Pneumonitis (NCI CTCAE v5)	Management	Follow-up
**Grade 1**<br>Radiographic changes only	• Consider delay of I-O therapy<br>• Monitor for symptoms every 2-3 days<br>• Consider Pulmonary and ID consults	• Re-image at least every 3 weeks<br><br>If worsens:<br>• Treat as Grade 2 or 3-4
**Grade 2**<br>Mild to moderate new symptoms	• Delay I-O therapy per protocol<br>• Pulmonary and ID consults<br>• Monitor symptoms daily, consider hospitalization<br>• 1 mg/kg/day methyl prednisolone IV or oral equivalent<br>• Consider bronchoscopy, lung biopsy	If improves to Grade 1:<br>• Resume I-O therapy per protocol<br>If persists > 5-7 days or recur:<br>• 0.5-1 mg/kg/day methylprednisolone or oral equivalent<br>• When symptoms improve to Grade 1, taper steroids over at least 1 month, consider prophylactic antibiotics for opportunistic infections, and resume I-O therapy per protocol.<br>If worsens or persists > 3-5 days with oral steroids:<br>• Treat as Grade 3-4
**Grade 3-4**<br>Severe new symptoms; New/worsening hypoxia; Life-threatening	• Discontinue I-O therapy per protocol<br>• Hospitalize<br>• Pulmonary and ID consults<br>• 2-4 mg/kg/day methyl prednisolone IV or IV equivalent<br>• Add prophylactic antibiotics for opportunistic infections<br>• Consider bronchoscopy, lung biopsy	If improves to baseline:<br>• Taper steroids over at least 6 weeks<br><br>If not improving after 48 hours or worsening:<br>• Add additional immunosuppression

Patients on IV steroids may be switched to an equivalent dose of oral corticosteroids (eg, prednisone) at start of tapering or earlier, after sustained clinical improvement is observed. Lower bioavailability of oral corticosteroids should be taken into account when switching to the equivalent dose of oral corticosteroids.

28-Sep-2020

Approved v4.0 930169531 4.0

Hepatic Adverse Event Management Algorithm

Rule out non-inflammatory causes. If non-inflammatory cause, treat accordingly and continue I-O therapy.
Consider imaging for obstruction.

Liver Function Test	Management	Follow-up
AST or ALT > ULN to 3.0 x ULN<br>and/or<br>T. bili > ULN to 1.5 x ULN	• Continue I-O therapy per protocol<br>• Consider increasing frequency of monitoring	• Continue LFT monitoring per protocol<br><br><u>If worsens</u>:<br>• Treat as below
AST or ALT > 3.0 to ≤ 5 x ULN<br>and/or<br>T. bili > 1.5 to ≤ 3 x ULN	• Delay I-O therapy per protocol<br>• Increase frequency of monitoring to every 3 days	<u>If returns to baseline</u>:<br>•Resume routine monitoring, resume I-O therapy per protocol<br><br><u>If elevations persist > 5-7 days or worsen:</u><br>• 0.5-1 mg/kg/day methylprednisolone or oral equivalent and when and AST ALT returns to <3X ULN and bili <1.5X ULN, taper steroids over at least 1 month, consider prophylactic antibiotics for opportunistic infections, and resume I-O therapy per protocol
AST or ALT > 5 x ULN<br>or<br>T.bili >3 x ULN	• Delay or Discontinue I-O therapy per protocol^<br>• Increase frequency of monitoring to every 1-2 days<br>• 1 to 2 mg/kg/day methylprednisolone IV or IV equivalent*<br>• Add prophylactic antibiotics for opportunistic infections<br>• Consult gastroenterologist or hepatologist	<u>If AST and ALT returns to <5X ULN and bili <3X ULN,</u><br>• Taper steroids over at least 1 month<br><br><u>If does not improve in >3-5 days, worsens, or rebounds:</u><br>• Add mycophenolate mofetil 1 g BID<br>• If no response within an additional 3-5 days, consider tacrolimus or other immunosuppressants per local guidelines<br>Note: Avoid infliximab due to potential risk of liver failure

Patients on IV steroids may be switched to an equivalent dose of oral corticosteroids (e.g. prednisone) at start of tapering or earlier, after sustained clinical improvement is observed. Lower bioavailability of oral corticosteroids should be taken into account when switching to the equivalent dose of oral corticosteroids.

^ Please refer to protocol dose delay and discontinue criteria for specific details.

*The recommended starting dose for AST or ALT > 20 x ULN or bilirubin >10 x ULN is 2 mg/kg/day methylprednisolone IV.

28-Sep-2020

Endocrinopathy Adverse Event Management Algorithm

Rule out non-inflammatory causes. If non-inflammatory cause, treat accordingly and continue I-O therapy.
Consider visual field testing, endocrinology consultation, and imaging.

Endocrinopathy	Management	Follow-up
Asymptomatic TSH abnormality<br>or<br>Hyperglycemia requiring no medical intervention	• Continue I-O therapy per protocol<br>• If TSH < 0.5 x LLN, or TSH > 2 x ULN, or consistently out of range in 2 subsequent measurements:<br>○ include fT4 at subsequent cycles as clinically indicated;<br>○ consider endocrinology consult	• Monitor TSH as per protocol<br>• Monitor blood sugar<br>• If symptomatic or blood sugar requiring initiation of treatment or change in management, manage as below
Symptomatic endocrinopathy<br>or<br>hyperglycemia requiring initiation of treatment or change in daily management or work up	• Evaluate endocrine function; consider endocrinology consult<br>• Consider pituitary scan<br><u>Symptomatic with abnormal lab/pituitary scan:</u><br>• Delay I-O therapy per protocol<br>• 1-2 mg/kg/day methylprednisolone IV or PO equivalent for hypophysitis<br>• Initiate appropriate hormone/medical therapy<br><u>No abnormal lab/pituitary MRI scan but symptoms persist:</u><br>• Continue IO therapy. Repeat labs in 1-3 weeks /MRI in 1 month<br><u>Hyperglycemia</u>:<br>Screen for Diabetic Ketoacidosis (DKA). If negative, continue IO and treat diabetes. If DKA hold IO, admit, manage DKA. Steroids not recommended for DKA/hyperglycemia	<u>If improves (with or without hormone replacement)</u>:<br>• Taper steroids over at least 1 month and consider prophylactic antibiotics for opportunistic infections<br>• Resume I-O therapy per protocol<br>• Patients with adrenal insufficiency may need to continue steroids with mineralocorticoid component
Suspicion of adrenal crisis (eg, severe dehydration, hypotension, shock out of proportion to current illness)	• Delay or discontinue I-O therapy per protocol<br>• Rule out sepsis<br>• Stress dose of IV steroids with mineralocorticoid activity<br>• IV fluids<br>• Consult endocrinologist	• Patients with adrenal insufficiency may need to continue steroids with mineralocorticoid component<br>• If adrenal crisis resolves and patient is adequately controlled with physiologic hormone replacement, patient can resume IO therapy per protocol.

Patients on IV steroids may be switched to an equivalent dose of oral corticosteroids (eg, prednisone) at start of tapering or earlier, after sustained clinical improvement is observed. Lower bioavailability of oral corticosteroids should be taken into account when switching to the equivalent dose of oral corticosteroids.

28-Sep-2020

Skin Adverse Event Management Algorithm

Rule out non-inflammatory causes. If non-inflammatory cause, treat accordingly and continue I-O therapy.

Rash	Management	Follow-up
Covering ≤ 30% BSA*	• Symptomatic therapy (eg, antihistamines, topical steroids)<br>• Continue I-O therapy per protocol	<u>If persists > 1-2 weeks or recurs</u>:<br>• Consider skin biopsy<br>• Delay I-O therapy per protocol<br>• Consider 0.5-1.0 mg/kg/day methylprednisolone IV or oral equivalent. Once improving , taper steroids over at least 1 month, consider prophylactic antibiotics for opportunistic infections, and resume I-O therapy per protocol<br><u>If worsens</u>:<br>• Treat as >30% BSA
Covering > 30% BSA (with or without symptoms)<br><br>Life threatening consequences*^	• Delay or discontinue I-O therapy per protocol<br>• Consider skin biopsy<br>• Dermatology consult<br>• 1.0 -2.0 mg/kg/day IV methylprednisolone IV or oral equivalent	<u>If improves to < 10% BSA:</u><br>• Taper steroids over at least 1 month and add prophylactic antibiotics for opportunistic infections<br>• Resume I-O therapy per protocol

Patients on IV steroids may be switched to an equivalent dose of oral corticosteroids (e.g. prednisone) at start of tapering or earlier, after sustained clinical improvement is observed. Lower bioavailability of oral corticosteroids should be taken into account when switching to the equivalent dose of oral corticosteroids.

*Refer to NCI CTCAE v5 for term-specific grading criteria.

^If Steven-Johnson Syndrome (SJS),toxic epidermal necrosis (TEN), Drug Reaction with Eosinophilia and Systemic Symptoms (DRESS) is suspected, withhold I-O therapy and refer patient for specialized care for assessment and treatment. If SJS, TEN, or DRESS is diagnosed, permanently discontinue I-O therapy.

28-Sep-2020

Approved v4.0 930169531 4.0

Neurological Adverse Event Management Algorithm

Rule out non-inflammatory causes. If non-inflammatory cause, treat accordingly and continue I-O therapy.

Grade of Neurological Toxicity (NCI CTCAE v5)	Management	Follow-up
**Grade 1**<br>Asymptomatic or mild symptoms;<br>Intervention not indicated	• Continue I-O therapy per protocol<br>• Discontinue I-O for select AEs^	• Continue to monitor per protocol.<br><u>If worsens:</u><br>• Treat as Grade 2 or Grade 3-4
**Grade 2**<br>Moderate symptoms;<br>Limiting instrumental ADL	• Delay I-O therapy per protocol<br>• Discontinue I-O for select AEs^<br>• Consider neurology consult<br>• Treat symptoms per local guidelines<br>• Consider 0.5 to 1 mg/kg/day methylprednisolone IV or PO equivalent	<u>If returns to baseline</u>:<br>• Resume I-O therapy per protocol when improved to baseline If worsens:<br>• Treat as Grade 3-4
**Grade 3-4**<br>Severe symptoms;<br>Limiting self-care ADL;<br>Life-threatening	• Discontinue I-O therapy per protocol<br>• Obtain neurology consult<br>• Treat symptoms per local guidelines<br>• 1-2 mg/kg/day IV methylprednisolone IV or IV equivalent<br>• Add prophylactic antibiotics for opportunistic infections	<u>If improves to Grade 2</u>:<br>• Taper steroids over at least 1 month<br><u>If worsens or atypical presentation</u>:<br>• Consider IVIG or other immunosuppressive therapies per local guidelines

Patients on IV steroids may be switched to an equivalent dose of oral corticosteroids (eg. prednisone) at start of tapering or earlier, after sustained clinical improvement is observed. Lower bioavailability of oral corticosteroids should be taken into account when switching to the equivalent dose of oral corticosteroids.

^Discontinue for any grade myasthenia gravis, Guillain-Barre syndrome, treatment-related myelitis, or encephalitis.

28-Sep-2020

MYOCARDITIS ADVERSE EVENT MANAGEMENT ALGORITHM

Myocarditis	Management	Follow-up
**Asymptomatic Troponin Elevation**	• Delay I-O therapy<br>• Repeat and monitor troponin<br>• Consider cardiology consultation with diagnostic workup; ECG, echocardiogram, cardiac MRI per recommendation of cardiologist<br>• Consider corticosteroids per treating physician	• Monitor troponin until recovery<br>• Resume I-O therapy upon recovery
Symptoms with mild to moderate activity or exertion	• Delay I-O therapy; hospitalization with cardiac monitoring<br>• Urgent cardiology consultation for evaluation and management<br>○ Troponin and BNP<br>○ ECG ± continuous cardiac monitoring<br>○ Echocardiogram<br>○ Cardiac MRI<br>• Prompt initiation of 2 mg/kg/day methylprednisolone IV or equivalent	• If worsens, intensify treatment according to grade<br>• Upon recovery, taper steroids over at least 1 month with close monitoring of troponin and BNP as well as for new symptoms<br>• Repeat cardiac MRI for post treatment assessment and cardiology follow-up<br>• Retreatment may be considered after recovery and completion of steroid taper
Severe with symptoms at rest or with minimal activity or exertion; intervention indicated<br><br>Life-threatening consequences; urgent intervention indicated (e.g., continuous IV therapy or mechanical hemodynamic support)	• Permanently discontinue I-O therapy<br>• Hospitalize to intensive cardiac monitoring<br>• Cardiac evaluation to include:<br>○ Troponin and BNP monitoring<br>○ ECG ± continuous cardiac monitoring<br>○ Echocardiogram<br>○ Cardiac MRI<br>○ Myocardial biopsy if feasible<br>• Immediate initiation of 2 mg/kg/day methylprednisolone IV or 1 g IV bolus<br>• Consider adding a second immunosuppressive agent<br>Additionally, for life threatening myocarditis:<br>• Hospitalize/transfer to institution with expertise in intensive cardiac monitoring<br>• Consider ATG as second agent given its immediate effect	• If no improvement, consider additional immunosuppression<br>• Upon recovery, taper steroids over at least 1 month with close monitoring of troponin and BNP as well as for new symptoms<br>• Repeat cardiac MRI for post treatment assessments and cardiology follow-up

For the protocols under CTCAE version 5.0. Patients on IV steroids may be switched to an equivalent dose of oral corticosteroids (eg, prednisone) at start of tapering or earlier, once sustained clinical improvement is observed. Lower bioavailability of oral corticosteroids should be taken into account when switching to the equivalent dose of oral corticosteroids. Prophylactic antibiotics should be considered in the setting of ongoing immunosuppression.
ATG = anti-thymocyte globulin; BNP = B-type natriuretic peptide; ECG = electrocardiogram; IV = intravenous; MRI = magnetic resonance imaging

10-Sep-2021

Approved v4.0 930169531 4.0

APPENDIX 6 ECOG AND LANSKY/KARNOFSKY PERFORMANCE STATUS SCALE

PERFORMANCE STATUS CRITERIA: ECOG Score	
**ECOG (Zubrod)**	
**Score**	**Description**
**0**	Fully active; able to carry on all pre-disease performance without restriction
**1**	Restricted in physically strenuous activity but ambulatory and able to carry out work of alight or sedentary nature, e.g., light house work, office work.
**2**	Ambulatory and capable of all self care but unable to carry out any work activities; up and about more than 50% of waking hours.
**3**	Capable of only limited self care; confined to bed or chair more than 50% of waking hours.
**4**	Completely disabled; cannot carry on any self care; totally confined to bed or chair.

PERFORMANCE STATUS CRITERIA: Karnofsky and Lansky		
**Score**	**Karnofsky Description (≥ 16 years of age)**	**Lansky Description (12 years to < 16 years age)**
**100**	Normal; no complaints; no evidence of disease.	Fully active, normal.
**90**	Able to carry on normal activity; minor signs or symptoms of disease.	Minor restrictions in physically strenuous activity.
**80**	Normal activity with effort; some signs or symptoms of disease.	Active, but tires more quickly.
**70**	Cares for self; unable to carry on normal activity or to do active work.	Substantial restriction of, and less time spent, in play activity.
**60**	Requires occasional assistance, but is able to care for most of their personal needs.	Out of bed, but minimal active play; keeps busy with quiet activities.
**50**	Requires considerable assistance and frequent medical care.	Gets dressed, but inactive much of day; no active play, able to participate in quiet play.
**40**	Disabled; requires special care and assistance.	Mostly in bed; participates in some quiet activities.
**30**	Severely disabled; hospital admission is indicated although death not imminent.	In bed; needs assistance even for quiet play.
**20**	Very sick; hospital admission necessary; active supportive treatment necessary.	Often sleeping; play limited to passive activities.
**10**	Moribund; fatal processes progressing rapidly.	No play; does not get out of bed.
**0**	Dead	Unresponsive

Approved v4.0 930169531 4.0

APPENDIX 7 AJCC MELANOMA STAGING (CANCER STAGING MANUAL 8TH EDITION)

Definition of Primary Tumor (T)

T Category	Thickness	Ulceration Status
**TX:** primary tumor thickness cannot be assessed (e.g., diagnosis by curettage)	Not applicable	Not applicable
**T0:** no evidence of primary tumor (e.g., unknown primary or completely regressed melanoma)	Not applicable	Not applicable
**Tis** (melanoma *in situ*)	Not applicable	Not applicable
**T1**	≤1.0 mm	Unknown or unspecified
T1a	<0.8 mm	Without ulceration
T1b	<0.8 mm<br>0.8-1.0 mm	With ulceration<br>With or without ulceration
**T2**	>1.0-2.0 mm	Unknown or unspecified
T2a	>1.0-2.0 mm	Without ulceration
T2b	>1.0-2.0 mm	With ulceration
**T3**	>2.0-4.0 mm	Unknown or unspecified
T3a	>2.0-4.0 mm	Without ulceration
T3b	>2.0-4.0 mm	With ulceration
**T4**	>4.0 mm	Unknown or unspecified
T4a	>4.0 mm	Without ulceration
T4b	>4.0 mm	With ulceration

Definition of Distant Metastasis (M)

M Category	Anatomic site	LDH level
**M0**	No evidence of distant metastasis	Not applicable
**M1**	Evidence of distant metastasis	See below
**M1a**	Distant metastasis to skin, soft tissue including muscle, and/or non-regional lymph node	Not recorded or unspecified
M1a(0)		Not elevated
M1a(1)		Elevated
**M1b**	Distant metastasis to lung with or without M1a sites of disease	Not recorded or unspecified
M1b(0)		Not elevated
M1b(1)		Elevated
**M1c**	Distant metastasis to non-CNS visceral sites with or without M1a or M1b sites of disease	Not recorded or unspecified
M1c(0)		Not elevated
M1c(1)		Elevated
**M1d**	Distant metastasis to CNS with or without M1a, M1b, or M1c sites of disease	Not recorded or unspecified
M1d(0)		Not elevated
M1d(1)		Elevated
Suffixes for M category: (0) LDH not elevated; (1) LDH elevated. No suffix is used if LDH is not recorded or is unspecified.		

Approved v4.0 930169531 4.0

Definition of Regional Lymph Node (N)

N Category	Number of tumor-involved regional lymph nodes	Presence of in-transit, satellite, or microsatellite metastases
**NX**	Regional nodes not assessed (e.g., SLN biopsy not performed, regional nodes previously removed for another reason)<br>**Exception**: pathological N category is not required for T1 melanomas, use cN	No
**N0**	No regional metastases detected	No
**N1**	One tumor-involved node or in-transit, satellite, and/or microsatellite metastases with no tumor-involved nodes	
N1a	One clinically occult (i.e., detected by SLN biopsy)	No
N1b	One clinically detected	No
N1c	No regional lymph node disease	Yes
**N2**	Two or three tumor-involved nodes or in-transit, satellite, and/or microsatellite metastases with one tumor-involved node	
N2a	Two or three clinically occult (i.e., detected by SLN biopsy)	No
N2b	Two or three, at least one of which was clinically detected	No
N2c	One clinically occult or clinically detected	Yes
**N3**	Four or more tumor-involved nodes or in-transit, satellite, or microsatellite metastases with two or more tumor-involved nodes, or any number of matted nodes with or without in-transit, satellite, and/or microsatellite metastases	
N3a	Four or more clinically occult (i.e., detected by SLN biopsy)	No
N3b	Four or more, at least one of which was clinically detected, or presence any number of matted nodes	No
N3c	Two or more clinically occult or clinically detected, and/or presence any number of matted nodes	Yes

Approved v4.0 930169531 4.0

AJCC Prognostic Stage Groups

Clinical (cTNM)

Clinical stage includes microstaging of the primary melanoma and clinical/radiologic/biopsy evaluation for metastases. By convention, clinical staging should be used after biopsy of the primary melanoma, with clinical assessment for regional and distant metastases. Note that pathological assessment of the primary melanoma is used for both clinical and pathological classification. Diagnostic biopsies to evaluate possible regional and/or distant metastasis also are included. Note there is only one stage group for clinical Stage III melanoma.

When T is....	And N is.....	And M is....	The clinical stage is...
Tis	N0	M0	0
T1a	N0	M0	IA
T1b	N0	M0	IB
T2a	N0	M0	IB
T2b	N0	M0	IIA
T3a	N0	M0	IIA
T3b	N0	M0	IIB
T4a	N0	M0	IIB
T4b	N0	M0	IIC
Any T, Tis	≥N1	M0	III
Any T	Any N	M1	IV

PATHOLOGICAL (pTNM)

Pathological staging includes microstaging of the primary melanoma, including any additional staging information from the wide excision (surgical) specimen that constitutes primary tumor surgical treatment and pathological information about the regional lymph nodes after SLN biopsy or therapeutic lymph node dissection for clinically evident regional lymph node disease.

When T is....	And N is.....	And M is....	The pathological stage is...
Tis	N0	M0	0
T1a	N0	M0	IA
T1b	N0	M0	IA
T2a	N0	M0	IB
T2b	N0	M0	IIA
T3a	N0	M0	IIA

Approved v4.0 930169531 4.0

When T is....	And N is.....	And M is....	The pathological stage is...
T3b	N0	M0	IIB
T4a	N0	M0	IIB
T4b	N0	M0	IIC
T0	N1b, N1c	M0	IIIB
T0	N2b, N2c, N3b or N3c	M0	IIIC
T1a/b-T2a	N1a or N2a	M0	IIIA
T1a/b-T2a	N1b/c or N2b	M0	IIIB
T2b/T3a	N1a-N2b	M0	IIIB
T1a-T3a	N2c or N3a/b/c	M0	IIIC
T3b/T4a	Any N ≥N1	M0	IIIC
T4b	N1a-N2c	M0	IIIC
T4b	N3a/b/c	M0	IIID
Ant T, Tis	Any N	M1	IV
Pathological Stage 0 (melanoma *in situ*) and T1 do not require pathological evaluation of lymph nodes to complete pathological staging; use cN information to assign their pathological stage.			

Adapted from: American Joint Committee on Cancer. Melanoma of the Skin. In: Amin MB, Edge SB, Greene FL, et al., editors. AJCC Cancer Staging Manual. 8th ed. Chicago: Springer International Publishing AG; 2018 pp 577-8.

Approved v4.0 930169531 4.0

APPENDIX 8 MUCOSAL MELANOMA STAGING

All mucosal melanoma, regardless of anatomical location, should be staged using the following classification.

The American Joint Committee on Cancer (AJCC) TNM Staging System for Mucosal Melanoma of the Head and Neck[a,b,c,d]

Primary Tumor (T)		Characteristics	
T3		Tumors limited to the mucosa and immediately underlying soft tissue, regardless of thickness or greatest dimension; for example, polypoid nasal disease, pigmented or nonpigmented lesions of the oral cavity, pharynx, or larynx	
T4		Moderately advanced or very advanced disease	
	T4a	Moderately advanced disease -<br>Tumor involving deep soft tissue, cartilage, bone, or overlying skin	
	T4b	Very advanced disease -<br>Tumor involving brain, dura, skull base, lower cranial nerves (IX, X, XI, XII), masticator space, carotid artery, prevertebral space, or mediastinal structures	
**Regional Lymph Nodes (N)**		**Characteristics**	
Nx		Regional lymph nodes cannot be assessed	
N0		No regional lymph node metastasis	
N1		Regional lymph node metastasis present	
**Distant Metastasis (M)**		**Characteristics**	
M0		No distant metastasis	
M1		Distant metastasis	

Staging group	Tumor	Node	Metastases
III	T3	N0	M0
IVA	T3-T4a	N1	M0
IVA	T4a	N0	M0
IVB	T4b	Any N	M0
IVC	Any T	Any N	M1

[a] NCCN Clinical Practice Guidelines in Oncology: Head and Neck Cancers. National Comprehensive Cancer Network. Version 1.2020 - February 12, 2020. Assessed March 22, 2020.

[b] Head and Neck. American Joint Committee on Cancer. AJCC Cancer Staging Manual. 8th edition. New York, NY: Springer, 2016.

[c] Histologic Grade (G): There is no recommended histologic grading system at this time.

[d] Prognostic Stage Groups: Currently, there is no clear ability to determine prognosis based on histologic differences.

Approved v4.0 930169531 4.0

APPENDIX 9 COUNTRY-SPECIFIC REQUIREMENTS

Country	Section Number and Title	Original Language	Country-specific Language or Differences
Argentina, Czech Republic, Germany, Romania, and Any Other Countries Where Exclusion of HIV Positive Participants is Locally Mandated	Table 2-1, Screening Procedural Outline - Clinical Laboratory Assessments		Add "HIV" to the list of laboratory tests
Argentina, Czech Republic, Germany, Romania, and Any Other Countries Where Exclusion of HIV Positive Participants is Locally Mandated	Section 6.2, Exclusion Criteria - 3k<br>Table 9.4.4-1, Clinical Laboratory Assessments	Known history of testing positive for human immunodeficiency virus (HIV) or known acquired immunodeficiency syndrome (AIDS)	Positive test for HIV.
Denmark, Germany, Greece, and Sweden	Table 2-1, Screening Procedural Outline - Informed Consent<br>Section 6.1, Inclusion Criteria - 3b		Adolescents can no longer be enrolled in these countries; participants must be ≥ 18 years of age.
**Denmark**			
Denmark	Table 4-1, Objectives and Endpoints - Exploratory	*Objective:*<br>• To explore potential association of biomarkers (eg, LAG-3 expression, PD-L1 expression, BRAF mutation status, TMB, genomic signatures soluble biomarkers, etc.) with clinical efficacy (RFS, DMFS, and OS) and/or incidence of adverse events of nivolumab and/or relatlimab by analyzing biomarker measures within the tumor microenvironment and periphery (eg, blood, serum, plasma, tumor tissue and PBMCs) in comparison to clinical outcomes.	*Objective:*<br>• To explore potential association of biomarkers (eg, LAG-3 expression, PD-L1 expression, soluble biomarkers, etc.) with clinical efficacy (RFS, DMFS, and OS) and/or incidence of adverse events of nivolumab and/or relatlimab by analyzing biomarker measures within the tumor microenvironment and periphery (eg, blood, serum, plasma, tumor tissue and PBMCs) in comparison to clinical outcomes.

Approved v4.0 930169531 4.0

Country	Section Number and Title	Original Language	Country-specific Language or Differences
		Endpoint:<br>• Association of biomarkers to RFS, DMFS, OS, incidence of AEs and LAG-3, PD-L1, BRAF mutation status, TMB, genomic signatures, soluble biomarkers, etc.	*Endpoint:*<br>• Association of biomarkers to RFS, DMFS, OS, incidence of AEs and LAG-3, PD-L1, soluble biomarkers, etc.
Denmark	Table 4-1, Objectives and Endpoints - Exploratory	*Objective:*<br>• To explore the role of ctDNA to understand MRD and disease recurrence predictability.<br>*Endpoint:*<br>• Association of recurrence with ctDNA positivity and dynamic changes in levels.	Not applicable to Denmark.
Denmark	Table 4-1, Objectives and Endpoints - Exploratory Endpoint	• Changes of PD-L1 and LAG-3, gene expression, and other biomarkers in the TME and peripheral.	• Changes of PD-L1 and LAG-3, and other biomarkers in the TME and peripheral.
Denmark	Section 5.4.6, Rationale for Evaluation of Biomarkers	... In the current trial, biomarkers, such as LAG 3, PD-L1, and MHC-II, and gene expression signatures will be evaluated for potential associations with clinical outcome. ... An emerging body of clinical evidence has shown that patients with detectable plasma ctDNA following complete resection of their tumors are at higher risk of disease recurrence than those patients with undetectable ctDNA after resection. Pre-treatment and on-treatment ctDNA will be evaluated for potential associations with clinical outcome in this study.	... In the current trial, biomarkers, such as LAG 3, PD-L1, and MHC-II, will be evaluated for potential associations with clinical outcome.
Denmark	Section 9.7, Genetics		This section is not applicable to Denmark.
Denmark	Section 9.8.1.3, Whole Blood DNA/RNA Analysis		This section is not applicable to Denmark.
Denmark	Section 9.8.1.4, Circulating Tumor DNA Analysis and		This section is not applicable to Denmark.

Country	Section Number and Title	Original Language	Country-specific Language or Differences
	Other Plasma Biomarkers		
Denmark	Section 9.8.2.4, Tumor Genotyping, Mutational Analysis, and Gene Expression Profiling		This section is not applicable to Denmark.
Denmark	Section 9.8.2.5, Tumor Markers Associated with Adverse Events	Upon occurrence of ≥ Grade 3 drug-related AE, if a biopsy of the affected organ is performed, it is strongly recommended that a specimen for biomarker analysis be collected for potential predictive markers of toxicity. A deidentified copy of the pathology report should be provided to the central lab if tissue is submitted. Such analyses of these tissue specimens may include but are not limited to the assessment of cell markers via protein expression or molecular/genomic assessments (eg, DNA and RNA extraction followed by the appropriate biomarker assay and subsequent analysis). Examples include but are not limited to H&E staining, IHC assays, RNA-sequencing, and the use of NGS technologies to enable whole genome sequencing and WES. Blood samples may also be collected to explore biomarkers associated with SAE, see Table 9.8-1.	Upon occurrence of ≥ Grade 3 drug-related AE, if a biopsy of the affected organ is performed, it is strongly recommended that a specimen for biomarker analysis be collected for potential predictive markers of toxicity. A deidentified copy of the pathology report should be provided to the central lab if tissue is submitted. Such analyses of these tissue specimens may include but are not limited to the assessment of cell markers via protein expression. Examples include but are not limited to H&E staining, IHC assays. Blood samples may also be collected to explore biomarkers associated with SAE, see Table 9.8-1.
**Germany**			
Germany	Table 2-1, Screening Procedural Outline - Body Imaging	Contrast-enhanced CT of the chest, abdomen, pelvis, and all suspected sites of disease, within 35 days prior to randomization. See Section 9.1.2 for further details and exceptions. For head and neck mucosal melanomas, contrast-enhanced CT of the neck also required at screening as reference for on-treatment nodal surveillance.	Contrast-enhanced CT of the chest, abdomen, pelvis, and all suspected sites of disease, within 35 days prior to randomization. See Section 9.1.2 for further details and exceptions. For head and neck mucosal melanomas, contrast-enhanced CT of the neck also required at screening as reference for on-treatment nodal surveillance. MRI may be used as an alternative imaging modality to CT at screening, during treatment, and in follow-up.

Approved v4.0 930169531 4.0

Country	Section Number and Title	Original Language	Country-specific Language or Differences
Germany	Table 2-2, On Study Treatment Procedural Outline - Body Imaging	In case of suspected lesions in the extremities, contrast-enhanced MRI can be substituted for contrast-enhanced CT.<br>For head and neck mucosal melanomas, contrast-enhanced MRI of the head and neck is required at every time point for nodal surveillance.	MRI may be used as an alternative imaging modality to CT at screening, during treatment, and in follow-up.
Germany	Table 2-3, Follow-up Assessments - Body Imaging	In cases of suspected lesions of the extremities, contrast-enhanced MRI may be substituted for contrast-enhanced CT.<br>For head and neck mucosal melanoma, contrast-enhanced MRI of the head and neck is required at every time point for nodal surveillance.	MRI may be used as an alternative imaging modality to CT at screening, during treatment, and in follow-up.
Germany	Section 3.2.6, Nivolumab Combined with Relatlimab Clinical Safety	Troponin is a highly sensitive non-specific indicator of myocardial injury. Troponin is not typically monitored during treatment with immune checkpoint inhibitors due to uncertain value in preventing immune-related cardiac morbidity. In CA224020, myocarditis was uncommon despite the context of regular protocol-mandated troponin monitoring and a requirement for cardiac assessment in participants with raised troponin values. While myocarditis is a recognized risk with immune checkpoint inhibitors, routine troponin monitoring during treatment has not been recommended by clinical guidelines due to lack of demonstrated value in preventing morbidity or mortality. The protocol contains a myocarditis treatment algorithm for prompt diagnostic evaluation and treatment of symptoms potentially related to myocarditis and excludes participants with a prior history of myocarditis.	The protocol contains a myocarditis treatment algorithm for prompt diagnostic evaluation and treatment of symptoms potentially related to myocarditis and excludes participants with a prior history of myocarditis.

Country	Section Number and Title	Original Language	Country-specific Language or Differences
Germany	Table 3.3.1-1, Risk Assessment	*Potential Risk of Clinical Significance:*<br>Cardiovascular AEs (ie, myocarditis, troponin elevation)<br>*Summary of Data:*<br>FDC (BMS-986213) IB<br>*Mitigation Strategy:*<br>Management of Myocarditis per AE Management Algorithm in Appendix 5 or as per institutional protocol/ investigator discretion.	*Potential Risk of Clinical Significance:*<br>Cardiovascular AEs (ie, myocarditis, troponin elevation)<br>*Summary of Data:*<br>FDC (BMS-986213) IB<br>SAV (BMS-986016) IB<br>*Mitigation Strategy:*<br>Management of Myocarditis per AE Management Algorithm in Appendix 5 or as per institutional protocol/ investigator discretion.<br>Management (including elevated asymptomatic troponin and NT-Pro-BNP), see Table 7.4.1-1, Section 9.4.5, and Appendix 5.
Germany	Section 9.1.1, Efficacy Assessment for the Study	In cases of suspected lesions of the extremities, MRI (with and without contrast) may be substituted for contrast-enhanced CT.	MRI may be used as an alternative imaging modality to CT at screening, during treatment and in follow-up.
Germany	Section 9.1.3, Investigator Assessment of Baseline Disease Status	Participant eligibility (disease-free status) must be confirmed by investigator prior to randomization. Baseline disease assessments should be performed within 35 days prior to randomization, including contrast-enhanced CT of the chest, abdomen, pelvis, and all suspected sites of disease. Baseline MRI of the brain (with and without contrast) is required for ALL participants during screening to rule out brain metastases, within 35 days prior to randomization. CT of the brain (without and with contrast) can be performed if MRI is contraindicated.	Participant eligibility (disease-free status) must be confirmed by investigator prior to randomization. Baseline disease assessments should be performed within 35 days prior to randomization, including contrast-enhanced CT of the chest, abdomen, pelvis, and all suspected sites of disease. MRI may be used as an alternative imaging modality to CT at screening, during treatment, and in follow-up.

Country	Section Number and Title	Original Language	Country-specific Language or Differences
Germany	Section 9.4.5, Cardiac Evaluation	Troponin elevations found during treatment will require a dose delay and require that the participant undergo a cardiac evaluation including cardiology consultation to rule out myocarditis. Following this evaluation, determination of treatment can be resumed based on Investigator's judgement to process with treatment, participant completion of AE management as needed (ie, corticosteroid taper or participant is on ≤ 10 mg prednisone or equivalent), and participant meeting the requirements per Table 7.4.1-1. Refer to Table 7.4.1-1 for myocarditis-related dose delay criteria.	All troponin elevations while on study will require a dose delay. Cardiology consultation and cardiac evaluation will be required during the first instance of troponin elevation while on study intervention. Cardiology consultation and cardiac evaluation for subsequent troponin elevations will be the purview of the investigator. Following a cardiology consultation and/or cardiac evaluation of on study troponin elevations, treatment can be resumed based on Investigator's judgement to proceed with treatment, participant completion of AE management as needed (ie, corticosteroid taper or participant is on ≤ 10 mg prednisone or equivalent), and participant meeting the requirements per Table 7.4.1-1.
**Greece**			
Greece	Section 6.2, Exclusion Criteria, 5a	Per Administrative Letter effective 13-Jul-2021: Greek sites should follow the stricter local regulations according to the Ministerial Decision G5a/59676/21-11-2016 (Government Gazette Issue No 4131/B/22-12-2016) - Article 11, and not allow participants in prison to participate in the trial.	
		Prisoners or participants who are involuntarily incarcerated. (Note: Under certain specific circumstances and only in countries where local regulations permit, a person who has been imprisoned may be included or permitted to continue as a participant. Strict conditions apply, and BMS approval is required.)	Prisoners or participants who are involuntarily incarcerated.
**Norway**			
Norway	Table 2-2, On Study Treatment Procedural Outline - Pregnancy Test (WOCBP)	For subsequent cycles, a negative pregnancy test should be documented within 3 days prior to dosing.	Pregnancy testing must be performed at monthly intervals during the same period as contraception is mandatory (until 5 months after end of study treatment) and during extended dose delays. Home pregnancy tests are acceptable, but results must be recorded.
	Table 2-3, Follow-up Assessments - Pregnancy Test (WOCBP)	Note: Pregnancy testing is only required at FU Visits 1 and 2 unless increased frequency and duration is required per local regulations.	

Approved v4.0 930169531 4.0

APPENDIX 10 PROTOCOL AMENDMENT SUMMARY OF CHANGE HISTORY

Overall Rationale for Protocol Amendment 02, 22-Feb-2023

The main reasons for this amendment are the following:

- The speedy randomization (expected 27 months but actual was 14 months) affects the estimated timing of the interim and final recurrence-free survival (RFS) analyses, holding all other statistical assumptions the same as the original calculations. This update in estimated timing of events clarified that number of RFS events should be the trigger for interim RFS analyses rather than time-based analysis trigger. Specifically, the first interim analysis of RFS (RFS IA#1) will be performed when approximately 309 RFS events have occurred (~75% information fraction). The second interim RFS analysis (RFS IA#2) will be performed when approximately 361 RFS events have occurred (~88% information fraction). In addition, 2 typos in the power for the interim analyses of RFS were corrected.
- A part of the secondary objective regarding outcomes on next line of therapy was moved to an exploratory objective. Progression-free survival 2 (PFS2) remains a secondary objective, but duration on next line of therapy was moved to a new exploratory objective. Added to this new exploratory objective was also time to next line of therapy.
- The population for the distant metastasis-free survival (DMFS) secondary objective was limited to randomized participants with Stage III/IVA/IVB no evidence of disease (NED) melanoma.
- An additional clarification for neck magnetic resonance imaging (MRI) was provided for head and neck mucosal melanomas, since imaging plays a vital role for the evaluation of these melanomas given the invasion into adjacent soft tissue.
- In order to consolidate the previously published and implemented country-specific amendments for Denmark, Germany, Greece, and Sweden with the global amendment in preparation for the European Union Clinical Trials Regulation (EU CTR) transition, certain country-specific paragraphs were delineated while others were consolidated for easier reading without changing context.

These revisions are specified below and have been incorporated into the Protocol Summary.

Revisions apply to all participants currently enrolled.

**Summary of Key Changes for Protocol Amendment 02**		
**Section Number & Title**	**Description of Change**	**Brief Rationale**
Table 2-1: Screening Procedural Outline	Added sentence about local regulations that do not allow adolescents (< 18 years) to participate in the study.<br>Added cardiology consultation to cardiac troponin (T or I).<br>Added creatine phosphokinase (CPK) screening row for Germany only.	Wording already present in country-specific amendments modified and/or added to consolidate with global amendment in preparation for EU CTR.

Approved v4.0 930169531 4.0

Summary of Key Changes for Protocol Amendment 02		
**Section Number & Title**	**Description of Change**	**Brief Rationale**
Table 2-2: On Study Treatment Procedural Outline	Added rows for "Office Visit/Clinical Observation" and "Troponin and CPK" evaluations for Germany only.	
Section 6.2: Exclusion Criteria	Added required cardiology consultation to cardiac troponin (T or I) to exclusion criterion 3)m).	
Table 7.4.1-1: AE Criteria for Delay, Resume, and Discontinue of Treatment	Added row for cardiac troponin T or I increased.	
Table 9.4.4-1: Clinical Laboratory Assessments	Added cardiology consultation to cardiac troponin (T or I).<br>Added CPK and on-treatment troponin evaluations for Germany only.	
Section 9.4.5: Cardiac Evaluation	Revised text regarding process following troponin elevations in participants during treatment.	
Table 9.8-1: Biomarker Sampling Schedule: All Arms	Added footnote that Denmark will not collect whole blood ribonucleic acid (RNA) or deoxyribose nucleic acid (DNA) or plasma circulating tumor DNA (ctDNA).	
Appendix 9: Country-Specific Requirements	Added and revised rows for Denmark-, Germany-, Greece-, and Sweden-specific requirements and differences.	
Table 2-1: Screening Procedural Outline<br>Table 2-2: On Study Treatment Procedural Outline<br>Table 2-3: Follow-up Assessments	Added that ultrasounds should not be submitted to imaging vendor.	To align with current BMS processes.
Table 2-2: On Study Treatment Procedural Outline<br>Table 2-3: Follow-up Assessments<br>Section 9.1.1: Efficacy Assessment for the Study	For body/brain imaging and ultrasounds, added that the assessments at every 26 weeks (± 14 days) should be "beyond the Week 108 imaging time point thereafter."	Clarification for the timing when sites should switch to imaging every 26 weeks.

Approved v4.0 930169531 4.0

Summary of Key Changes for Protocol Amendment 02		
**Section Number & Title**	**Description of Change**	**Brief Rationale**
Table 2-2: On Study Treatment Procedural Outline<br>Table 2-3: Follow-up Assessments<br>Section 8.1.2 Post-study Intervention Study Follow-up<br>Section 9.1.1: Efficacy Assessment for the Study<br>Section 9.1.2: Imaging Assessment for the Study<br>Section 9.1.4: Investigator Assessment of Recurrence	Clarified that imaging assessments for mucosal melanoma participants should continue based on M staging at study entry, ie, until distant recurrence for participants who are classified as Stage M0 and until regional recurrence for Stage M1 participants.	To align with the tumor/node/ metastasis (TNM) staging system for Mucosal Melanoma of the Head and Neck from the American Joint Committee on Cancer (AJCC v8).
Table 2-2: On Study Treatment Procedural Outline<br>Table 2-3: Follow-up Assessments	Added imaging requirements for mucosal melanoma of the head and neck to require MRI at every time point.	To ensure surveillance for mucosal head and neck participants is using the most clinically appropriate modality.
Table 2-2: On Study Treatment Procedural Outline<br>Table 2-3: Follow-up Assessments<br>Section 9.1.5: Patient-Reported Outcomes	Added that Health Outcomes assessments "must" be completed prior to dosing, and "preferably as the first procedure on day of dosing."<br>Added that adolescent participants will continue to complete only the EQ-5D-5L even if they become ≥ 18 years of age during treatment or follow-up.<br>Added that if dose is delayed after collection of Health Outcomes, submit data change form to move original data entry to unscheduled visit.	To align with current BMS processes and for clarity.
Table 2-3: Follow-up Assessments<br>Section 9.8: Biomarkers<br>Table 9.8-1: Biomarker Sampling Schedule: All Arms	Added that biomarker collections should be stopped for participants who start subsequent systemic therapy.	Collection of biomarkers after subsequent systemic therapy provides inconclusive findings of dynamic changes in disease biology and, hence, these collections are omitted.
Table 2-2: On Study Treatment Procedural Outline<br>Table 2-3: Follow-up Assessments<br>Section 9.5: Pharmacokinetics	Added that pharmacokinetics/ immunogenicity (PK/IMG) collections should be stopped for participants who start on subsequent systemic therapy.	Since PK collections are used for study intervention-intended estimations, collections are stopped once participant stops study intervention.

Approved v4.0 930169531 4.0

Summary of Key Changes for Protocol Amendment 02		
**Section Number & Title**	**Description of Change**	**Brief Rationale**
Table 2-3: Follow-up Assessments	Removed collection of corticosteroids and other immune-modulating medications beyond 135 days of last dose for AE/SAEs related to study drug.	Section 9.2.1 states that investigators are not obligated to actively seek AEs/SAEs in former study participants, but are required to continue to report drug-related AEs/SAEs and their management; therefore, this sentence was removed for consistency.
	Revised footnote that defines timing of Long-Term Follow-up and Survival visits.	In cases where a Follow-up visit is unable to be completed, timing of Survival Follow-up visit has been clarified.
Section 3.3: Benefit/Risk Assessment	Added paragraph with data from safety memo.	To ensure pertinent study information is included to support patient safety.
Table 4-1: Objectives and Endpoints<br>Section 10.3: Analysis Sets<br>Table 10.4.1-2: Definition of Estimands for Primary and Secondary Endpoints	In the DMFS secondary efficacy objective, changed “Stage III/IV” to “Stage III/IVA/IVB” in the description of participants with completely resected NED melanoma.	To align with prior adjuvant studies CM238 and CM915 as well as clinical relevance of occurrence of distant metastasis in Stage III disease rather than Stage IV.
Table 4-1: Objectives and Endpoints<br>Table 10.4.1-2: Definition of Estimands for Primary and Secondary Endpoints<br>Table 10.4.3-2: Summary of Secondary Endpoint Analysis	One component of the secondary objective regarding outcomes on next-line therapies (ie, duration of treatment on next-line therapies) was moved to a new exploratory objective. Also added to this new exploratory objective was time to next line of therapy. The other component of this secondary objective (ie, PFS2) remains a secondary objective.	Since duration of treatment on next line of therapies is valuable in the context of payor perspectives accounting for variation in clinical practice, this was moved to an exploratory endpoint.
	Removed exploratory objective and endpoint regarding time to confirmed deterioration in health-related quality of life (HRQoL).	To align with current research plan and reduce analytic burden.
Section 5.4.2: Rationale for Use of Nivolumab + Relatlimab Fixed Dose Combination	Removed text regarding characteristics of effector T cells.	To align with updated BMS standards for relatlimab protocols.
Section 5.4.9: Rationale for MRI Surveillance of Head and Neck Mucosal Melanomas	Added section.	To align with changes in protocol.
Section 7.3: Blinding	Removed paragraph regarding randomization schedules.	Included in error in previous amendment but not applicable to the study.

Approved v4.0 930169531 4.0

Summary of Key Changes for Protocol Amendment 02		
**Section Number & Title**	**Description of Change**	**Brief Rationale**
Section 8.1: Discontinuation From Study Treatment	Added "or new primary melanoma" to disease recurrence as reason for discontinuation.	To clarify existing discontinuation reason.
Section 9.1.1: Efficacy Assessment for the Study	Revised text regarding the discontinuation of follow-up scans for Stage III and IV participants.	To ensure comprehensive data collection for all Stage III and Stage IV participants regardless of initiation of subsequent systemic therapy or not.
Section 9.1.4.2: Equivocal Recurrence/Suspect Lesions	Added "but no later than 12 weeks" to timeframe of subsequent scan from the time when recurrence is suspected.	To define an expected timeframe for confirmation of recurrence.
	Added that even though ultrasounds are not to be submitted to imaging vendor, any recurrence identified by ultrasound must be recorded as recurrence event.	To clarify ultrasound-identified recurrence events.
Section 9.1.4.3: Definition of Recurrence<br>Table 9.8-1: Biomarker Sampling Schedule: All Arms	Excluded MMIS in defining recurrence as appearance of new melanoma lesions.	To clarify recurrence definition.
Section 9.1.5.3: EQ-5D-5L	Removal of reference to minimal important differences not existing.	New minimal important difference estimates were recently released.
Section 9.2.3: Follow-up of AEs and SAEs	Updated text to current practice.	To align with updated BMS processes.
Section 9.2.5: Pregnancy	Added that pregnancy reporting is not required for WOCBP partners of male participants.	For clarity.
Table 9.5-1: Pharmacokinetic and Immunogenicity Sampling Schedule for All Arms<br>Table 9.8-1: Biomarker Sampling Schedule: All Arms	Added footnote that upon drug-related adverse event (AE), optional sample should be collected before dosing is resumed.	For clarity.

Approved v4.0 930169531 4.0

Summary of Key Changes for Protocol Amendment 02		
**Section Number & Title**	**Description of Change**	**Brief Rationale**
Section 10.2.1: Recurrence-free Survival<br>Section 10.2.2: Overall Survival<br>Table 10.2.2-1: Formal Analysis of Overall Survival - Operating Characteristics at Each Time Point<br>Section 10.3: Analysis Sets<br>Table 10.4.2-1: Primary Endpoints<br>Section 10.5.1: Interim Analyses of RFS<br>Section 10.5.2: Interim Analysis of OS	Updated the RFS interim analyses to be event-based rather than time-based. Also revised text and tables regarding projected accrual rates, estimated number of months expected for follow-up at the time of the interim RFS analyses, and corrected cumulative power for both interim analyses.<br>Made adjustments to expected timing of OS events at the interim analyses based on the new projections for RFS interims and final analyses.	To adjust for shortened randomization timelines, and corrected a typo regarding the estimate of power at both RFS interim analyses.
	Clarified the condition under which RFS IA#2 may be skipped.	To ensure that no 2 RFS analyses are within 6 months of each other.
Section 10.3: Analysis Sets<br>Section 10.4.6.3: Patient-Reported Outcomes	Removed PRO analysis population.	To clarify population sets that are analyzed.
Table 10.4.1-1: Censoring Scheme for Definition of Recurrence-free Survival	Added row for baseline disease as censored beginning at date of randomization.<br>Removed secondary non-melanoma primary malignancy from the censoring algorithm.	To clarify censoring scheme.
Appendix 5: Management Algorithms for Studies Under CTCAE Version 5.0	Updated myocarditis management algorithm.	To provide guidance for asymptomatic troponin elevation management and follow-up.
Throughout	Updated Functional Assessment of Cancer Therapy (FACT-GP5) to Functional Assessment of Chronic Illness Therapy (FACIT-GP5).	The copyright holder has updated the preferred name.
	Added references to Appendix 9 where appropriate.	For clarity.
All	Minor formatting and typographical corrections.	Changes are minor and therefore have not been summarized.

Approved v4.0 930169531 4.0

Overall Rationale for Protocol Amendment 01, 18-May-2022

Despite limited clinical studies evaluating immuno-oncologic treatment outcomes in adolescent melanoma, with the small number of participants in these studies, results have shown that the safety profiles and treatment effects in pediatric participants are generally comparable to adult participants.[1,2,3,4,5] Surgical and medical management of adolescent melanoma continues to derive guidelines from adult melanoma treatment. Hence the addition of adolescent participants ≥ 12 years through < 18 years of age covers an unmet need within the melanoma population under study. The Food and Drug Administration (FDA) approval in Mar-2022 of Opdualag™ for the treatment of adult and adolescent patients (12 years and older and weighing at least 40 kg) with unresectable or metastatic melanoma provides further justification for the inclusion of this population in future studies.

To provide an earlier opportunity for evaluation of the treatment effect, an additional interim analysis for efficacy is being added for the primary endpoint of recurrence-free survival (RFS). The analysis is planned 8 months after the last patient is randomized. The second RFS interim analysis (IA) is as specified in the earlier version of the protocol.

Given the regulatory and clinical impact of overall survival (OS) as a positive meaningful study outcome, with the potential to test this at an earlier time point, distant metastasis-free survival (DMFS) was moved from key secondary endpoint to other secondary endpoint and consequently removed from the statistical testing hierarchy. Overall survival (OS) will remain as a key secondary endpoint. Overall survival assumptions were updated, based on updated data from CA209238. Interim analyses of OS were also specified to be performed by the Data Monitoring Committee (DMC).

Though novel anticancer therapies have revolutionized the field, the majority of these therapies including immune checkpoint inhibitors can cause a wide spectrum of cardiotoxic effects.[6] Both American Society of Clinical Oncology (ASCO)-2018 and European Society for Medical Oncology (ESMO)-2020 guidelines recommend performing a baseline cardiovascular risk assessment before anticancer treatment.[7,8] ASCO-2018 recommends electrocardiogram (ECG) and considering troponin, especially in patients treated with combination immune therapies.[8] Per these guidelines, participants with a history of myocarditis, regardless of etiology, were excluded from participation in this study to prevent any potential added risk with study drug exposure; a requirement for baseline troponin testing was also added.

Existing data and National Comprehensive Cancer Network guidelines support ultrasonography as a preferred modality compared to other imaging modalities in the management of Stage III melanoma patients based on sensitivity, specificity, and surveillance benefit.[9] Ultrasonography was updated as a study requirement for surveillance of participants who have a sentinel lymph node biopsy but do not undergo complete lymph node dissection (CLND) and left as optional for those with CLND.

Approved v4.0 930169531 4.0

The benefit/risk section of the protocol was updated to reflect updated data from the publication of the combination dosing study in first-line metastatic melanoma, which continues to suggest a favorable safety profile of the combination therapy.

Additional minor changes include protocol clarifications, harmonization of collection durations (days or weeks versus months), and formatting changes. Revisions to the protocol summary have been made to align with changes throughout the protocol. This amendment includes changes from approved Administrative Letters 01, 02 and 04 and those changes are not listed in the table below.

References

[1] Longo MI, Lázaro P, Bueno C, et al. Fluorodeoxyglucose-positron emission tomography imaging versus sentinel node biopsy in the primary staging of melanoma patients. Dermatol Surg 2003;29:245-8.

[2] Balch CM, Soong SJ, Smith T, et al. Long-term results of a prospective surgical trial comparing 2 cm vs. 4 cm excision margins for 740 patients with 1-4 mm melanomas. Ann Surg Oncol 2001;8:101-8.

[3] Harris MN, Shapiro RL, Roses DF. Malignant melanoma. Primary surgical management (excision and node dissection) based on pathology and staging. Cancer 1995;75(2 Suppl):715-25.

[4] Jen M, Murphy M, Grant-Kels JM. Childhood melanoma. Clin Dermatol 2009;27:529-36.

[5] Saiyed FK, Hamilton EC, Austin MT. Pediatric melanoma: incidence, treatment, and prognosis. Pediatric Health Med Ther 2017;8:39-45.

[6] Alexandre J, Cautela J, Ederhy S, et al. Cardiovascular toxicity related to cancer treatment: a pragmatic approach to the American and European cardio-oncology guidelines. J Am Heart Assoc 2020;9.

[7] Curigliano G, Lenihan D, Fradley M, et al. Management of cardiac disease in cancer patients throughout oncological treatment: ESMO consensus recommendations. Ann Oncol 2020;31:171-90.

[8] Brahmer JR, Lacchetti C, Schneider BJ, et al. Management of immune-related adverse events in patients treated with immune checkpoint inhibitor therapy: American Society of Clinical Oncology clinical practice guideline. J Clin Oncol 2018;36:1714-68.

[9] Xing Y, Bronstein Y, Ross MI, et al. Contemporary diagnostic imaging modalities for the staging and surveillance of melanoma patients: a meta-analysis. J Natl Cancer Inst 2011;103: 129-42.

Approved v4.0 930169531 4.0

SUMMARY OF KEY CHANGES FOR PROTOCOL AMENDMENT 01		
**Section Number & Title**	**Description of Change**	**Brief Rationale**
Throughout	To align with the naming convention in the United States prescribing information (USPI), nivolumab will precede relatlimab when in reference to any combination therapy, and relatlimab and nivolumab FDC has been renamed to nivolumab + relatlimab (nivo + rela) fixed-dose combination (FDC) throughout the protocol.	To align the naming convention with the USPI.
Protocol Summary	Text has been updated to align with changes made throughout the protocol.	Alignment between Protocol Summary and body of the protocol.
Table 2-1: Screening Procedural Outline (CA224098)	Specified complete physical examination at screening.	Clarification only.
Table 2-1: Screening Procedural Outline (CA224098)	In regard to the informed consent for adolescent participants, a note was added referencing Appendix 2 for details regarding minor assent.	Hyperlink added to reference to further details for minor assent.
Table 2-1: Screening Procedural Outline (CA224098)<br>Section 6.1: Inclusion Criteria<br>Table 9.8-1: Biomarker Sampling Schedule: All Arms<br>Section 9.8.2: Tumor Samples<br>Section 9.8.2.1: Tumor Sample Collection	For tumor sample submission procedures at baseline, the timeline for submission was updated from 3 months to 90 days for specificity.	Clarification on timing added for specificity.
Table 2-1: Screening Procedural Outline (CA224098)<br>Table 2-2: On Study Treatment Procedural Outline (CA224098)<br>Table 2-3: Follow-up Assessments (CA224098)<br>Section 9.1.1: Efficacy Assessment for the Study<br>Section 9.1.3: Investigator Assessment of Baseline Disease Status	Under the body imaging assessment, clarified that contrast-enhanced computed tomography (CT) scans for the listed body scans are required.	Clarified the requirement for contrast-enhanced CT imaging.

Approved v4.0 930169531 4.0

SUMMARY OF KEY CHANGES FOR PROTOCOL AMENDMENT 01		
**Section Number & Title**	**Description of Change**	**Brief Rationale**
Table 2-1: Screening Procedural Outline (CA224098)<br>Table 2-2: On Study Treatment Procedural Outline (CA224098)<br>Table 2-3: Follow-up Assessments (CA224098)<br>Section 5.4.8: Rationale for Ultrasound in Disease Surveillance	The following changes were made to the ultrasound assessments:<br>• Ultrasound will now be required of all participants with positive sentinel lymph node biopsy and who do not undergo immediate complete lymph node dissection (CLND). A note has also been added to indicate ultrasound is optional if the participant has undergone CLND.<br>• The ultrasound assessment interval on study and during follow-up was updated from 6 months to 26 weeks.<br>• A new section that provides the rationale for requiring ultrasound for surveillance has been added.	Updated to make ultrasound a study requirement for all sentinel lymph node dissection (SLND) participants (rather than optional) based on the sensitivity and specificity of ultrasound as a surveillance modality, and to harmonize this requirement across the global and country-specific protocols.<br><br>Clarified timing to align with language previously used in the protocol (weeks vs months) for imaging duration.
Table 2-1: Screening Procedural Outline (CA224098<br>Table 2-2: On Study Treatment Procedural Outline (CA224098)	Performance status assessment has been updated to include both Eastern Cooperative Oncology Group (ECOG) and Lansky/Karnofsky assessments depending on participant age.	Performance scale has been updated to be used depending on participant age (adults vs adolescent age group).
Table 2-1: Screening Procedural Outline (CA224098)<br>Table 9.4.4-1: Clinical Laboratory Assessments	Added troponin as an assessment at screening.	Included baseline troponin testing for identification of cardiac risk, including required cardiac evaluation in case of continued elevation on repeat of test during screening.
Table 2-2: On Study Treatment Procedural Outline (CA224098)	Participants must receive their first dose of study intervention within 3 days of randomization, changed from IRT drug assignment.	Consistency with other assessments that are based off randomization.
Table 2-2: On Study Treatment Procedural Outline (CA224098)<br>Table 2-3: Follow-up Assessments (CA224098)<br>Appendix 9: Country Specific Requirements	Norway will include monthly pregnancy testing during the same period as contraception is mandatory and during extended dose delays.	Added detailed information on Norway follow-up pregnancy testing as required per health authority response.

Approved v4.0 930169531 4.0

SUMMARY OF KEY CHANGES FOR PROTOCOL AMENDMENT 01		
**Section Number & Title**	**Description of Change**	**Brief Rationale**
Table 2-2: On Study Treatment Procedural Outline (CA224098)<br>Table 2-3: Follow-up Assessments (CA224098)<br>Section 9.1.1: Efficacy Assessment for the Study	The interval of timing between body imaging, brain imaging, and ultrasound has been updated from 6 months to 26 weeks, and 2 years has been clarified as 104 weeks.	Clarified timing to harmonize with language previously used in the protocol (weeks vs. months) for imaging duration.
Table 2-2: On Study Treatment Procedural Outline (CA224098)<br>Table 2-3: Follow-up Assessments (CA224098)<br>Section 9.1.1: Efficacy Assessment for the Study	Follow-up body and brain imaging scans has been discontinued during follow-up, once systemic therapy starts for a melanoma recurrence or for a new non-melanoma tumor after study drug discontinuation.<br>In Section 9.1.1, clarified when follow-up scans may be discontinued (ie, in cases when participant has started systemic therapy after unequivocal recurrence or for a new non-melanoma tumor after study drug discontinuation).	Clarification for when discontinuing imaging collections is allowed (after unequivocal recurrence or new non primary melanoma requiring systemic therapy).
Table 2-2: On Study Treatment Procedural Outline (CA224098)<br>Table 2-3: Follow-up Assessments (CA224098)<br>Section 9.1.5: Patient Reported Outcomes	Adolescent participants will only be required to complete the EQ-5D-5L assessment for the patient-reported outcomes.	Adolescent population is only required to complete EQ-5D-5L.
Table 2-2: On Study Treatment Procedural Outline (CA224098)<br>Table 2-3: Follow-up Assessments (CA224098)<br>Section 9.1.4.1: Unequivocal Recurrence<br>Table 9.8-1: Biomarker Sampling Schedule: All Arms<br>Section 9.8.2: Tumor Samples	Tumor tissue collection via biopsy or surgical resection at disease recurrence must be performed if medically feasible and must be submitted to the central laboratory within 30 days of collection.	Clarification of protocol requirement and incorporation of a timeframe for submission of tissue specimen to the central laboratory.

Approved v4.0 930169531 4.0

SUMMARY OF KEY CHANGES FOR PROTOCOL AMENDMENT 01		
**Section Number & Title**	**Description of Change**	**Brief Rationale**
Table 2-3: Follow-up Assessments (CA224098)	For concomitant medications, removed reference to subsequent therapy and specified collection of corticosteroid/immune-modulating medications only for AE/SAE considered study drug related.	Removal of subsequent therapy as it is addressed elsewhere and redundant and limiting the collection of corticosteroid/immune-modulating medications for safety.
Table 2-3: Follow-up Assessments (CA224098)<br>Figure 5.1-1: Study Design Schema<br>Section 5.3: End of Study Definition<br>Section 10.3: Analysis Sets<br>Section 10.4.1: General Considerations	For survival status in long-term follow-up, further specified timing of 12 weeks instead of 3 months and a window of 14 days was included for consistency with the footnote.<br>Minimum follow-up until end of study was changed from 6 to 8 years.<br>Additional follow-up may continue up to 5 years from the final overall survival (OS) analysis.	Harmonize timing of months to weeks and addition of window for scheduling flexibility.<br>Clarification provided on length of survival follow-up of 8 years minimum including up to 5 years after final OS analysis.
Table 2-3: Follow-up Assessments (CA224098)<br>Table 4.1: Objectives and Endpoints<br>Table 10.4.3-2: Summary of Secondary Endpoint Analysis	Clarified the wording and intent of collecting subsequent therapy and associated outcomes for participants who recur.	Clarification only.
Table 2-3: Follow-up Assessments (CA224098)<br>Table 9.8-1: Biomarker Sampling Schedule: All Arms	For biomarker circulating tumor DNA (ctDNA) collections, the 6-month collection timeframe was specified to 26 weeks. There is also a new collection window of 90 days but must not exceed 212 days between collections. In addition, a sentence from footnote b was moved to the notes section to emphasize the in-person visits of the biomarker collections in Long-term Follow-up.	Clarified timing to align with language used in the protocol (weeks vs months) with inclusion of window for flexibility. Defined timeframe between collections to allow collection to align with imaging timing.<br>Emphasized the in-person visits of the biomarker collections in Long-term Follow-up.
Section 3: Introduction	Updated the background language on FGL1-LAG3 pathway based on relatlimab interaction with FGL1 ligand.	Updated per nivolumab + relatlimab FDC IB.

Approved v4.0 930169531 4.0

SUMMARY OF KEY CHANGES FOR PROTOCOL AMENDMENT 01

Section Number & Title	Description of Change	Brief Rationale
Section 3.1: Study Rationale<br>Section 3.2.2: Nivolumab Combined with Relatlimab Clinical Activity<br>Section 3.2.6: Nivolumab Combined with Relatlimab Clinical Safety<br>Section 3.3: Benefit/Risk Assessment	Additional data from the CA224047 study has been provided to support the clinical efficacy and safety of nivo + rela FDC.	Data from publication of combination dosing study in first-line metastatic melanoma updated to support rationale for combined clinical efficacy and safety.
Section 3.2.2: Nivolumab Combined with Relatlimab Clinical Activity<br>Section 3.2.6: Nivolumab Combined with Relatlimab Clinical Safety	• Clinical efficacy and safety sections have been updated and streamlined.<br>• Efficacy numbers for Study CA224020 Parts D1 and E and CA224047 overall survival/overall response rate have been updated.<br>• Updated safety information added for study CA224020.	Updates per nivolumab + relatlimab FDC IB.
Section 3.2.6: Nivolumab Combined with Relatlimab Clinical Safety	Minor wording updates were made based on the addition of troponin during screening and exclusion of participants with history of myocarditis.	Safety data information updated and clarified for earlier identification of cardiac risk based on actual study information.
Section 3.3: Benefit/Risk Assessment	Updated benefit/risk assessment for nivolumab and relatlimab combination therapy.	Updates per nivolumab + relatlimab FDC IB.
Table 3.3.1-1: Risk Assessment	Ultrasound added as a study procedure risk for comprehensiveness.	For treatment of imaging risks in the study, ultrasound was added.

Approved v4.0 930169531 4.0

SUMMARY OF KEY CHANGES FOR PROTOCOL AMENDMENT 01		
**Section Number & Title**	**Description of Change**	**Brief Rationale**
Table 4-1: Objectives and Endpoints<br>Section 10.1: Statistical Hypotheses<br>Section 10.4.1: General Considerations<br>Table 10.4.1-2: Definition of Estimands for Primary and Secondary Endpoints<br>Table 10.4.3-1: Secondary Efficacy Endpoints<br>Table 10.4.3-2: Summary of Secondary Endpoint Analysis	Distant metastasis-free survival (DMFS) was moved to other secondary endpoints and is no longer a key secondary endpoint that will be tested formally.<br>OS will be tested on the statistical hierarchy and remains a key secondary endpoint.	To maximize the chance of testing for a potentially positive OS, which has more significant clinical and regulatory impact.
Table 4-1: Objectives and Endpoints<br>Section 5.5.4: Rationale for Continued Adjuvant Therapy for Patients Who Are Diagnosed with Melanoma in Situ During Study Treatment<br>Section 10.4.1: General Considerations<br>Table 10.4.1-1: Censoring Scheme for Primary Definition of Recurrence-free Survival<br>Table 10.4.2-1: Primary Endpoints<br>Table 10.4.2-2: Summary of Primary Endpoint Analysis.	Melanoma in situ will no longer be considered as a new primary malignancy and consequently will no longer be considered an event for the primary endpoint of RFS.	Recurrence rates for completely excised melanoma in situ are sufficiently low that patients are considered cured following excision, with the exception of certain subtypes that may recur locally (ie, lentigo maligna).
Table 4-1: Objectives and Endpoints	Objective response rates were removed as an exploratory biomarker endpoint.	Objective response rate is not applicable for a study with adjuvant treatment.
Table 4-1: Objectives and Endpoints	LAG-3 was included in an exploratory endpoint for an objective to evaluate changes in the tumor microenvironment and peripheral, indicative of immunomodulatory effect.	Elaboration of exploratory biomarkers in tumor microenvironment under study included.

Approved v4.0 930169531 4.0

SUMMARY OF KEY CHANGES FOR PROTOCOL AMENDMENT 01		
**Section Number & Title**	**Description of Change**	**Brief Rationale**
Section 5.1: Overall Design<br>Section 5.4.7: Rationale for Inclusion of Adolescent Participants<br>Section 5.5.2: Dose Rationale for Adolescent Participants<br>Table 7.1-1: Study Interventions	Adolescent participants ≥ 12 years are now eligible to participate in the study. The protocol has been updated throughout to include pediatric dosing information, which for a subset of pediatric participants will be weight based.<br>Rationale for the inclusion of adolescent participants and dosing rationale for this population have been added.	Addition of adolescent participants.<br>Addition of dosing for adolescent participants aged ≥ 12 years to 18 years, including weight-based differences for this group of participants.
Figure 5.1-1: Study Design Schema	The study design schema and associated footnotes were updated per the changes to Protocol Amendment 01.	Addition of dosing for pediatric participants for nivo + rela FDC and nivolumab only added to study schema including footnotes.
Section 5.1.1: Data Monitoring Committee and Other Committees<br>Section 10.1.1: Multiplicity Adjustment	Added a paragraph describing the administrative alpha penalty if the Data Monitoring Committee (DMC) requests unplanned look efficacy data prior to the RFS IA.	Clarification of alpha administrative penalty that will occur if DMC requests unplanned look at efficacy data prior to RFS IA.
Section 5.4.6: Rationale for Evaluation of Biomarkers	Updated to include more recent study results from CA224047 and add rationale for collection of ctDNA samples.	Updated data from publication of combination dosing study to support rationale for biomarker evaluation.
Section 5.5.1: Justification for Fixed Dose Combination Dosing	• Minor rewording throughout the section and updated infusion safety data have been provided.<br>• Also updated text in regard to population pharmacokinetic (PPK) analysis.<br>• Updated language for dose justification for 30-minute duration of infusion.	Clarification of protocol verbiage and updates per nivolumab + relatlimab FDC IB
Section 5.6.1: Nivolumab Clinical Pharmacology Summary<br>Section 5.6.2: Relatlimab Clinical Pharmacology Summary	Updated language for clinical pharmacology of nivolumab and relatlimab.	Updates per nivolumab + relatlimab FDC IB.

Approved v4.0 930169531 4.0

SUMMARY OF KEY CHANGES FOR PROTOCOL AMENDMENT 01		
**Section Number & Title**	**Description of Change**	**Brief Rationale**
Section 6.1: Inclusion Criteria<br>Section 9.2: Adverse Events<br>Section 10.3: Analysis Sets<br>Appendix 2: Study Governance Considerations	Multiple changes were made throughout the protocol to include legally acceptable representatives (LAR):<br>Inclusion criterion 1) a) was modified to permit legally acceptable representatives (LAR) with the inclusion of pediatric participants.<br>Added participant's LAR as an individual who may report AEs if applicable.<br>Redefined enrolled population as participant or their LAR who signed informed consent and were registered into Interactive Response Technology.<br>Inclusion of LAR in the informed consent process given inclusion of pediatric participants.	Updated the informed consent and AE reporting section for participants to conform with required regulatory and ethics guidelines for a legally acceptable representative.
Section 6.1: Inclusion Criteria<br>Section 6.2: Exclusion Criteria	Inclusion criterion 2) a) and exclusion criteria 1) a) were modified to exclude participants with ocular melanoma.	Terminology change from uveal to ocular.
Section 6.1: Inclusion Criteria	Inclusion criterion 2) c) was modified regarding timing of complete resection prior to randomization, from 12 weeks to 90 days.	Clarified timing to harmonize with language used in the protocol (days vs weeks).
Section 6.1: Inclusion Criteria	Inclusion criterion 2) d) was modified to require a brain magnetic resonance imaging (MRI) scan at screening.	Clarification on procedures required at study screening.
Section 6.1: Inclusion Criteria	Inclusion criterion 2) f) was modified to include both ECOG and Lansky/Karnofsky performance status scores, depending on participant age.	Clarification on performance scale for assessment of pediatric participants.

Approved v4.0 930169531 4.0

SUMMARY OF KEY CHANGES FOR PROTOCOL AMENDMENT 01		
**Section Number & Title**	**Description of Change**	**Brief Rationale**
Section 6.1: Inclusion Criteria	Inclusion criterion 2) g) was added as a placeholder to align formatting with country-specific protocol amendments.	Added for formatting alignment with country-specific amendments.
Section 6.1: Inclusion Criteria	Inclusion criterion 3)a) was updated to criterion 3)b) to permit pediatric participants ≥ 12 years of age to participate unless local regulations do not permit participants < 18 years of age.	Clarification provided for pediatric participant inclusion to conform with local regulatory and institutional policies.
Section 6.2: Exclusion Criteria	Exclusion criterion 1) f) was updated to specify concurrent non-melanoma malignancy.	Clarification that the criterion is referring to non-melanoma malignancy.
Section 6.2: Exclusion Criteria	Exclusion criterion 1) i) was added to exclude participants with a history of myocarditis, regardless of etiology.	Participants with history of myocarditis, regardless of etiology, will be excluded from participation in this study to prevent any potential added risk with study drug exposure.
Section 6.2: Exclusion Criteria	Exclusion criterion 2) g) was added to exclude participants who received prior treatment with BRAF/MEK targeted agents.	Included to clarify restrictions for prior treatment.
Section 6.2: Exclusion Criteria	Exclusion criterion 2) h) was added to exclude participants who have had prior radiation therapy within 2 weeks prior to first dose of study medication.	Clarification on washout duration for previous radiotherapy recipients.
Section 6.2: Exclusion Criteria	Exclusion criterion 3) m) introduces a troponin screening requirement.	Included baseline troponin testing for identification of cardiac risk, including required cardiac evaluation in case of continued elevation on repeat of test.
Table 7.1-1: Study Interventions	Added approved Opdualag™ to the current name of BMS-986213.	Naming update.

Approved v4.0 930169531 4.0

SUMMARY OF KEY CHANGES FOR PROTOCOL AMENDMENT 01		
**Section Number & Title**	**Description of Change**	**Brief Rationale**
Section 7.1: Study Interventions Administered	A note regarding the pediatric dosing calculations for body weight dosing was included.	Clarification on using cumulative weight change for dose adjustments for newly added pediatric population.
Section 7.1.1: Study Treatment Details	• The final bullet point regarding participant discontinuation was deleted as it is addressed in the discontinuation section of the protocol.<br>• The term study treatment has been replaced with immunotherapy.<br>• As all infusions are diluted, we have removed an inaccurate statement.	Minor rewording and corrections to the protocol as these items are addressed in different sections.
Section 7.3: Blinding	Text added to clarify that randomization schedules are provided directly to the individual dispensing blinded study intervention, but who are not involved in the study in any other aspect.	To indicate that the blinding is maintained throughout the study by differentiating blinded versus unblinded individuals.
Section 7.3: Blinding	Details regarding DMC access to unblinded treatment codes, and the timing of participant, investigator, site staff, and sponsor access to unblinded treatment assignments has been included.	Clarifying information regarding timing of unblinding for DMC, sponsor, participants, investigator, site staff, and sponsor access to unblinded treatment assignments is added.
Section 7.4.2: Criteria to Resume Treatment	For consistency with Section 8.1.1, an exception regarding delays due to steroid taper in management of drug related-adverse events (AEs) was included.	Clarification for consistency.
Section 7.6: Treatment Compliance	Clarified that source data will be reviewed at routine monitoring visits.	Reworded for accuracy.

Approved v4.0 930169531 4.0

SUMMARY OF KEY CHANGES FOR PROTOCOL AMENDMENT 01		
**Section Number & Title**	**Description of Change**	**Brief Rationale**
Section 7.7.1.1: Prohibited Treatments	Clarified that all radiation therapy, including palliative, is prohibited while on study.<br>Clarified any melanoma-directed surgery is prohibited while on study treatment.	Clarification surgery and radiation therapy prohibited treatments.
Section 7.7.2: Other Restrictions and Precautions	Revised wording of text detailing restrictions of participation in other interventional clinical trials.	Clarification on restrictions for participating in interventional trials vs observational studies.
Section 7.7.2.1: SARS-CoV-2 Vaccination Guidelines	Updated to state that the efficacy and safety of non-live vaccines (including non-live coronavirus disease 2019 [COVID-19] vaccines) in study participants is unknown.	Clarification of unknown COVID-19 vaccine effects from study drug.
Section 8.2.1: Individual Discontinuation Criteria	Clarified that at the time of discontinuation from the study, an immediate follow-up visit may be required.	Removed statement about an early termination visit as there is no such visit and clarified visit requirements at study drug discontinuation.
Section 9.1.2: Imaging Assessment for the Study	Removed a statement that assessments should be performed by the same investigator or delegate at all time points.	To reduce site burden of having the same investigator perform the assessment at all time points.
Section 9.1.3: Investigator Assessment of Baseline Disease Status<br>Section 9.1.4.2: Equivocal Recurrence/Suspect Lesions	Revised text to provide more detail on suspect lesions.	Clarified how to manage suspect lesions during screening.
Section 9.1.4: Investigator Assessment of Recurrence<br>Section 9.1.4.3: Definition of Recurrence	Removed malignant melanoma in situ (MMIS) from definition of recurrence as is not considered recurrence. Date of MMIS will be captured still to allow for future sensitivity analysis.	Clarification that MMIS is not considered recurrence.

Approved v4.0 930169531 4.0

SUMMARY OF KEY CHANGES FOR PROTOCOL AMENDMENT 01		
**Section Number & Title**	**Description of Change**	**Brief Rationale**
Section 9.1.5: Patient-Reported Outcomes	Minor clarification that questionnaires should be obtained prior to participant withdrawal from the study.	Clarification only.
Section 9.2.1: Time Period and Frequency for Collecting AE and SAE Information	For collection of nonserious adverse events, a timeframe of all study treatment and 135 days following discontinuation of study intervention was added, and added that adverse events associated with severe acute respiratory syndrome coronavirus-2 must be collected from time of consent and during treatment period.	The timeframe for the collection of non-serious AEs was added.
Table 9.4.4-1: Clinical Laboratory Assessments	Updated TSH assessment to add total T3 and total T4.	Updated to align with the local lab collection parameters.
Section 9.4.5: Cardiac Evaluation	New section defining a cardiac evaluation for participants who have elevated troponin at screening.	Provided details on “cardiac evaluation” requirements.
Section 9.5: Pharmacokinetics	Clarification to emphasize the EOI-PK sampling collection instructions.	Clarification on EOI sampling.
Section 9.5: Pharmacokinetics<br>Section 9.8:<br>Biomarkers	With inclusion of adolescent participants, added text for appropriate reduction of blood draw volume for participants < 26 kg or other reasons per institutional guidelines.	Necessary to permit appropriate reduction of blood draw volumes given adolescent population.
Table 9.8-1: Biomarker Sampling Schedule: All Arms	Footnote “e” was modified to clarify that submission of specimens, tissue, or blood, must be for participants who have consented to optional specimen submission.	Clarification that requires specimen submission only for participants who have consented to optional specimen submission.
Table 9.8-1: Biomarker Sampling Schedule: All Arms	A new column for plasma biomarkers has been added.	Separate plasma collection (K2EDTA tube) is needed for biomarkers that are measurable in plasma (ie, soluble FGL1).

Approved v4.0 930169531 4.0

SUMMARY OF KEY CHANGES FOR PROTOCOL AMENDMENT 01		
**Section Number & Title**	**Description of Change**	**Brief Rationale**
Section 9.8.1.2: Immunophenotyping	A paragraph regarding MDSC was removed.	The information was not applicable to the section.
Section 9.8.2.5: Tumor Markers Associated with Adverse Events<br>Appendix 9: Country Specific Requirements	Added a reminder that a deidentified copy of the pathology report should accompany tissue specimen submission for drug-related Grade 3 or higher AE.	Clarification only.
Section 10.1.1: Multiplicity Adjustment	Changed the statistical hierarchy to remove DMFS. The primary endpoint is RFS and the key secondary endpoint is OS.	To maximize the chance to test OS earlier for a more significant regulatory and clinical impact, DMFS was removed as a primary endpoint and moved to other secondary endpoint.
Section 10.2.1: Recurrence-Free Survival	The approximate time from first participant randomization to the required observed RFS events was modified from 56 to 59 months.	Change is the result of a different method of simulation after adding an additional interim analysis at 8 months minimum follow-up.
Section 10.2.1: Recurrence-Free Survival<br>Table 10.4.2-1: Primary Endpoints<br>Section 10.5.1:<br>Interim Analyses of RFS	Addition of a new time-based interim analysis (IA) of RFS, 8 months after the last patient is randomized.	An earlier interim analysis of RFS was added to provide an earlier opportunity for evaluation of treatment effect and is time based.
Section 10.2.2: Overall Survival<br>Section 10.5.2: Interim Analysis of OS<br>Section 10.5.3: Interim Analyses of RFS and OS: Additional Details	Multiple changes were made in these sections:<br>• Details regarding DMFS analyses were removed.<br>• Updated information was used from CA209238 5-Year survival for the calculations of OS sample size and power. Details added regarding addition of interim OS analyses were included.<br>• Final OS was changed to Year 8.	DMFS is no longer formally tested, and thus removed corresponding sections.<br>The target hazard ratio, expected number of deaths, and final timing of OS were all updated.<br>Interim OS analyses were added to provide an opportunity for evaluation of the treatment effect.

Approved v4.0 930169531 4.0

SUMMARY OF KEY CHANGES FOR PROTOCOL AMENDMENT 01		
**Section Number & Title**	**Description of Change**	**Brief Rationale**
Section 10.3: Analysis Sets<br>Table 10.4.1-1: Censoring Scheme for Primary Definition of Recurrence-free Survival<br>Table 10.4.1-2: Definition of Estimands for Primary and Secondary Endpoints	Second non-melanoma primary cancer has been modified to secondary non-melanoma primary malignancy.	Minor rewording for more accurate description.
Section 10.4.4: Exploratory Endpoints(s)<br>Section 10.4.6.1: Pharmacokinetic Analyses	Minor clarifying updates were made to the pharmacokinetic analyses.	Clarification of the pharmacokinetic analyses.
Section 10.4.6.3: Patient-Reported Outcomes	Minor edit to clarify that all exploratory patient reported outcome endpoints will be analyzed in the PRO analysis population	Clarification only.
Appendix 2: Study Governance Considerations	Added text for legally acceptable representatives in the informed consent process.	Added text based on inclusion of adolescent participants and participants who may not be able to provide consent.
Appendix 2: Study Governance Considerations	Added 2 new sections titled:<br>• BMS Commitment to Diversity in Clinical Trials<br>• Data Protection, Data Privacy, and Data Security	Added text for BMS' commitment to diversity in clinical trials, and to align BMS practice and comply with EU-CTR requirements.
Appendix 6: ECOG and Lansky/ Karnofsky Performance Status Scale	Lansky/Karnofsky performance status scale has been added.	Addition of performance assessment scale for pediatric participants.
Appendix 9: Country Specific Requirements	Germany's requirements have been removed from the global protocol and will be included in their country-specific protocol.	Removed Germany-specific requirements as Germany has a country-specific amendment.

Approved v4.0 930169531 4.0

SUMMARY OF KEY CHANGES FOR PROTOCOL AMENDMENT 01

Section Number & Title	Description of Change	Brief Rationale
Appendix 9: Country Specific Requirements	Revised such that whole blood DNA for Denmark will be collected, but extensive genomic mapping will not be performed.	Updated Denmark requirements to state extensive mapping will not be performed.
All	Minor formatting and typographical corrections.	Changes are minor and therefore have not been summarized.

Approved v4.0 930169531 4.0

STATISTICAL ANALYSIS PLAN
FOR CLINICAL STUDY REPORT

A PHASE 3, RANDOMIZED, DOUBLE-BLIND STUDY OF ADJUVANT IMMUNOTHERAPY WITH NIVOLUMAB + RELATLIMAB FIXED-DOSE COMBINATION VERSUS NIVOLUMAB MONOTHERAPY AFTER COMPLETE RESECTION OF STAGE III-IV MELANOMA

PROTOCOL CA224098

VERSION # 2.0

DATE: 31-Jul-2023

TABLE OF CONTENTS

STATISTICAL ANALYSIS PLAN FOR CLINICAL STUDY REPORT 1
TABLE OF CONTENTS 2
LIST OF TABLES 6
LIST OF FIGURES 7
1 BACKGROUND AND RATIONALE 8
2 STUDY DESCRIPTION 9
2.1 Study Design 9
2.2 Treatment Assignment 11
2.3 Blinding and Unblinding 12
2.4 Protocol Amendments 13
2.5 Data Monitoring Committee and Other Committees 20
3 OBJECTIVES 21
4 ENDPOINTS 23
4.1 Efficacy Endpoints 23
4.1.1 Recurrence-Free Survival (RFS) 23
4.1.1.1 Definition of Recurrence-Free Survival 23
4.1.2 Overall Survival 26
4.1.3 Distant Metastasis-Free Survival (DMFS) 26
4.1.4 Progression-Free Survival on Next-Line Therapy (PFS2) 26
4.1.5 Duration of Treatment on Next-Line Therapy 29
4.1.6 Time to Next Treatment 29
4.1.7 Treatment-Free Interval (TFI) 29
4.1.8 Freedom from Recurrence 29
4.1.9 Clinical Outcomes Assessments 29
4.1.9.1 EORTC QLQ-C30 30
4.1.9.2 EQ-5D-5L 31
4.1.9.3 FACIT-GP5 33
4.2 Safety Endpoints 33
4.3 Other Endpoints 34
4.3.1 Pharmacokinetics 34
4.3.2 Biomarkers 34
4.3.3 Immunogenicity 34
4.4 Estimands 35
4.4.1 Supplemental Estimand of Recurrence-Free Survival (Censoring for Subsequent Therapy) 40
5 SAMPLE SIZE AND POWER 43
5.1 Recurrence Free-Survival 43
5.2 Overall Survival 44
5.3 Testing Strategy 45
6 STUDY PERIODS, TREATMENT REGIMENS, AND POPULATIONS FOR ANALYSES 46
6.1 Study Periods 46
6.1.1 Baseline Period 46
6.1.2 Post Baseline Period 46
6.2 Treatment Regimens 47

6.3 Populations for Analyses ... 47
7 STATISTICAL ANALYSES ... 48
7.1 General Methods ... 48
7.2 Study Conduct ... 48
7.2.1 General Study Information ... 48
7.2.2 Accrual ... 48
7.2.3 Protocol Deviations ... 49
7.2.4 Relevant Protocol Deviations ... 49
7.3 Study Population ... 49
7.3.1 Participant Disposition ... 49
7.3.2 Demographics and Other Baseline Disease Characteristics ... 50
7.3.3 Medical History ... 51
7.3.4 Prior Melanoma Directed Therapy Agents ... 51
7.3.5 Physical Examinations ... 51
7.3.6 Discrepancies Between IRT and CRF Information ... 51
7.4 Extent of Exposure ... 52
7.4.1 Administration of Study Therapy ... 52
7.4.2 Modifications of Study Therapy ... 53
7.4.2.1 Dose Delays ... 53
7.4.2.2 Infusion Interruptions and Rate Changes ... 53
7.4.2.3 Dose Escalations ... 54
7.4.2.4 Dose Reductions ... 54
7.4.2.5 Dose Omissions ... 54
7.4.3 Concomitant Medications ... 54
7.4.3.1 Immune Modulating Medication ... 54
7.4.3.2 Subsequent Cancer Therapy ... 55
7.5 Efficacy ... 55
7.5.1 Analysis of Recurrence-Free Survival (RFS) ... 56
7.5.1.1 Supplemental Analysis of RFS ... 57
7.5.1.2 Sensitivity Analyses of RFS ... 58
7.5.1.3 Subset Analyses of RFS ... 58
7.5.1.4 Participant Follow-up for RFS ... 59
7.5.2 Analysis of Overall Survival ... 59
7.5.2.1 Supplemental Analyses of Overall Survival ... 60
7.5.2.2 Sensitivity Analyses of Overall Survival ... 60
7.5.2.3 Subset Analyses of Overall Survival ... 60
7.5.2.4 Current Status of OS Follow-up ... 61
7.5.3 Analysis of Distant Metastasis-Free Survival (DMFS) ... 61
7.5.4 Analysis of Progression-free Survival Through Next-line Therapy (PFS2) ... 62
7.5.5 Interim Analyses ... 63
7.5.5.1 Interim Analyses of RFS ... 63
7.5.5.2 Interim Analysis of OS ... 63
7.5.5.3 Interim Analyses of RFS and OS: Additional Details ... 64
7.6 Safety ... 64
7.6.1 General Methods for Adverse Events ... 64
7.6.2 Deaths ... 64

Approved v2.0 930199387 2.0

7.6.3 Serious Adverse Events 65
7.6.4 Adverse Events Leading to Discontinuation of Study Therapy 65
7.6.5 Adverse Events Leading to Dose Modification 65
7.6.6 Adverse Events 65
7.6.7 Select Adverse Events (EU/ROW Submissions) 66
7.6.7.1 General Methods for Select Adverse Events 66
7.6.7.2 Incidence of Select AE 66
7.6.7.3 Time-to Onset of Select AE 67
7.6.7.4 Time-to Resolution of Select AE 67
7.6.8 Immune-Mediated Adverse Events (US Submission) 68
7.6.8.1 General Methods for Immune-Mediated Adverse Events 68
7.6.9 Other Events of Special Interest 69
7.6.10 Multiple Events 70
7.6.11 Laboratory Parameters 70
7.6.11.1 General Methods for Laboratory Tests 70
7.6.11.2 Hematology 71
7.6.11.3 Serum Chemistry 71
7.6.11.4 Electrolytes 71
7.6.11.5 Additional Analyses 71
7.6.12 Vital Signs 72
7.6.13 Electrocardiogram 72
7.6.14 Physical Measurements 73
7.6.15 Non-Protocol Medical Procedures 73
7.6.16 Pregnancy 73
7.6.17 Adverse Events By Subgroup 73
7.6.18 Analysis of SARS-CoV-2 73
7.7 Pharmacokinetics 73
7.7.1 Pharmacokinetic Concentrations 73
7.7.2 Ctrough and Ceoi 74
7.8 Biomarkers 74
7.8.1 Analysis of PD-L1 Expression 74
7.8.2 Analysis of LAG-3 Expression 75
7.9 Immunogenicity Analysis 76
7.10 Clinical Outcomes Assessments 77
7.10.1 EORTC QLQ-C30 77
7.10.2 EuroQoL EQ-5D-5L 78
7.10.3 FACIT-GP5 79
7.11 COVID-19 Related Analyses 80
7.11.1 COVID-19 Related Disposition Events 80
7.11.2 COVID-19 Related Dose Modifications 80
7.11.3 COVID-19 Related Adverse Events 80
8 CONVENTIONS 80
8.1.1 Pharmacokinetic Summaries 81
9 CONTENT OF REPORTS 82
9.1 Within-Trial Analyses Performed To Date 82
10 DOCUMENT HISTORY 83

11 REFERENCES 85
APPENDIX 1 TIME-TO ONSET AND TIME-TO RESOLUTION DEFINITION AND CONVENTIONS FOR SELECT ADVERSE EVENTS, IMMUNE-MEDIATED ADVERSE EVENTS AND EVENTS OF SPECIAL INTEREST 88
APPENDIX 2 BIOMARKER ENDPOINTS 90
APPENDIX 3 IMMUNOGENICITY ANALYSIS: BACKGROUND AND RATIONALE 91

Approved v2.0 930199387 2.0

LIST OF TABLES

Table 2.4-1: Amendments to the protocol / primary and secondary endpoints.............13
Table 3-1: Study Objectives and Endpoints 21
Table 4.1.1.1-1: Censoring Scheme for Definition of Recurrence-Free Survival...............24
Table 4.1.9.1-1: Time Windows for EORTC-QLQ-C30 Assessments...............................31
Table 4.1.9.2-1: Time Windows for EQ-5D-5L Assessments 32
Table 4.4-1: List of Main Estimands...35
Table 4.4-2: List of Supplemental Estimands..39
Table 4.4-3: Censoring Scheme for Supplemental Estimand Analysis of Recurrence-Free Survival 40
Table 5.2-1: Formal Analysis of Overall Survival - Operating Characteristics at Each Time Point 45
Table 7.4.1-1: Administration of Study Therapy: Nivo + Rela FDC Arm 52
Table 7.4.1-2: Administration of Study Therapy: Nivolumab Arm 53
Table 10-1: Document History 83

LIST OF FIGURES

Figure 2.1-1: Study Design 10
Figure 4.1.1.1-1: RFS Definition - Main Estimand 25
Figure 4.1.4-1: PFS2 Definition 28
Figure 4.4-1: RFS Definition - Supplemental Estimand 42

Approved v2.0 930199387 2.0

1 BACKGROUND AND RATIONALE

Study CA224098 is a Phase 3, randomized, double-blind study of nivolumab + relatlimab (nivo + rela) fixed-dose combination (FDC) compared with nivolumab monotherapy in completely resected Stage III-IV melanoma.

The study will allow for direct comparison of the clinical benefits, as measured by the primary endpoint of recurrence free survival (RFS), provided by nivo + rela FDC compared with nivolumab monotherapy administered over a treatment duration of 12 months.

This statistical analysis plan (SAP) details analyses that are planned for inclusion in the Clinical Study Report (CSR) for CA224098 study.

Research Hypothesis:

Treatment with systemically administered nivo + rela FDC when compared with nivolumab will result in improved RFS in participants with completely resected Stage III-IV melanoma.

Schedule of Analyses:

The primary hypothesis for the study is that nivo + rela FDC is superior to nivolumab monotherapy in achieving RFS as assessed by the investigator.

Two interim analyses for efficacy (RFS) will be performed based on number of events. The first interim analysis for RFS (RFS IA#1) will be performed when approximately 309 RFS events have occurred (~75% information fraction). At that time, a minimum follow-up of 14 months is expected for all randomized participants (ie, approximately 28 months from first participant randomized in the study).

If RFS IA#1 is not statistically significant, the second interim analysis for RFS (RFS IA#2) will be performed when approximately 361 RFS events have occurred (~88% information fraction). At that time, a minimum follow-up of 24 months is expected for all randomized participants (ie, approximately 38 months from first participant randomized in the study).

These interim analyses for RFS will allow for early formal testing for superiority, and the boundaries for declaring superiority will be derived based on the actual number of events.

Final analysis will be performed when approximately 410 RFS events have occurred. This is projected to happen at approximately 52 months after the first participant is randomized.

Assuming the RFS result is significantly superior, there will be an interim analysis of OS performed by the DMC at the time of RFS significance (either at RFS IA#1, RFS IA#2, or RFS FA). If RFS is statistically significant at one of the interims (RFS IA#1, RFS IA#2), but OS is not statistically significant, an additional interim OS analysis will be performed by the DMC at approximately 52 months from the randomization of the first participant and, if not statistically significant, another OS IA will be performed at approximately 71 months from the randomization of the first participant. Final OS analysis will be performed when approximately 309 OS events have occurred or at 90 months from the randomization of the first participant, whichever comes first.

Approved v2.0 930199387 2.0

2 STUDY DESCRIPTION

2.1 Study Design

This is a Phase 3, randomized, double-blind study of nivo + rela FDC compared with nivolumab monotherapy in participants (≥ 12 years of age) with completely resected Stage IIIA (> 1 mm tumor in lymph node)/B/C/D or Stage IV NED melanoma by American Joint Committee on Cancer version 8 (AJCC v8). Efficacy of nivo + rela FDC, as measured by RFS time (as assessed by the investigator), will be compared to nivolumab monotherapy.

Participants will be randomized 1:1 to receive treatment with one of the following:

- Nivo + rela FDC dosing:
  - ≥ 18 years of age **OR** ≥ 12 years to < 18 years of age and ≥ 40 kg: nivolumab 480 mg and relatlimab 160 mg IV every 4 weeks (Q4W)
  - ≥ 12 years to < 18 years of age and < 40 kg: nivolumab 6 mg/kg and relatlimab 2 mg/kg IV Q4W
- Nivolumab dosing:
  - ≥ 18 years of age **OR** ≥ 12 years to < 18 years of age and ≥ 40 kg: nivolumab 480 mg IV Q4W
  - ≥ 12 years to < 18 years of age and < 40 kg: nivolumab 6 mg/kg IV Q4W

Randomization will be stratified by AJCC v8 stage and geographic region. All participants will be treated until recurrence of disease (excluding melanoma in situ), unacceptable toxicity, participant withdrawal of consent, or a maximum of 1 year of treatment from first dose (maximum of 13 doses), whichever occurs first.

This study will consist of 3 phases: screening, treatment, and follow-up. The study design schematic is presented in Figure 2.1-1.

Approved v2.0 930199387 2.0

Figure 2.1-1: Study Design

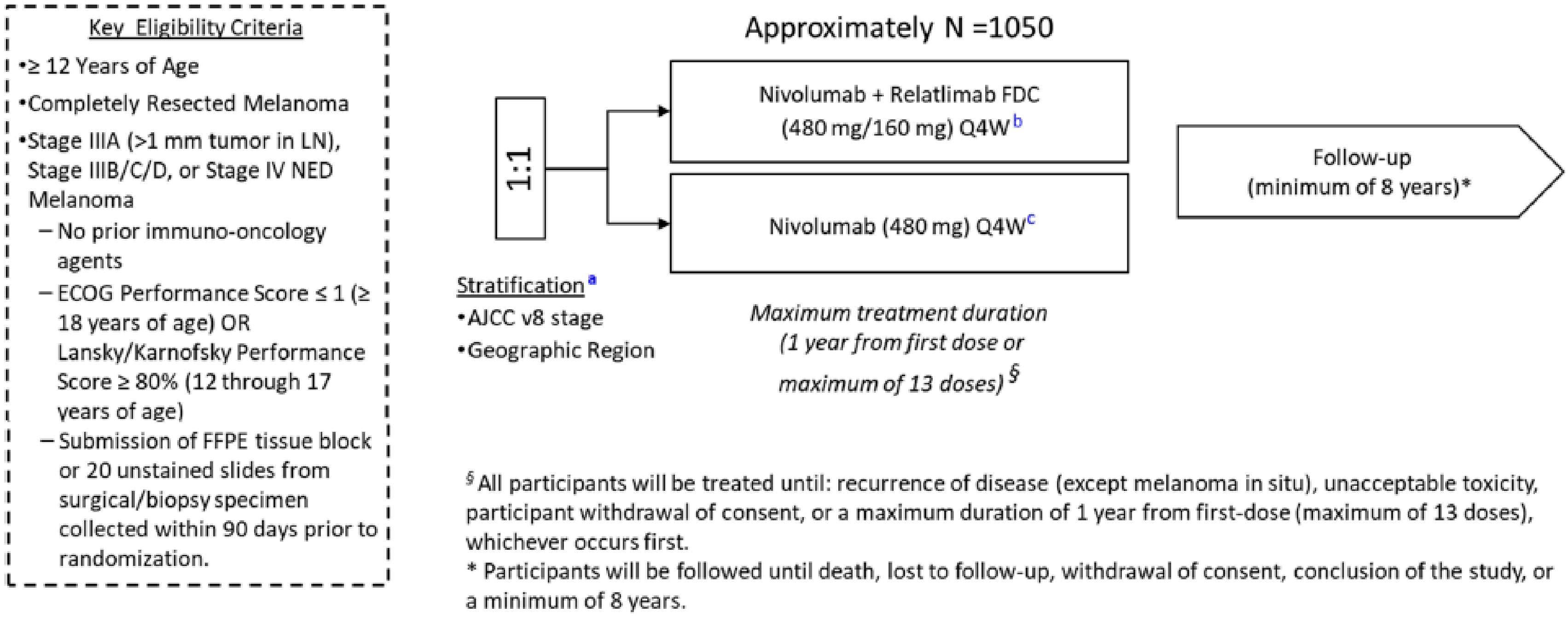

Abbreviations: AJCC v8, American Joint Committee on Cancer version 8; ECOG, Eastern Cooperative Oncology Group; FDC, fixed-dose combination; FFPE, formalin fixed paraffin embedded; LN, lymph node; NED, no evidence of disease; ROW, rest of the world; Q4W, every 4 weeks

[a] Stratification:

- AJCC v8: Stages IIIA/IIIB, Stage IIIC, Stages IIID/IV (including all participants with mucosal melanoma, Stage III, Stage IVA, Stage IVB, and Stage IVC)
- Geographic Regions: USA/Canada/Australia, Europe, ROW

[b] Nivo + rela FDC dosing:

- ≥ 18 years of age **OR** ≥ 12 years to < 18 years of age and ≥ 40 kg: nivolumab 480 mg and relatlimab 160 mg IV every 4 weeks (Q4W)
- ≥ 12 years to < 18 years of age and < 40 kg: nivolumab 6 mg/kg and relatlimab 2 mg/kg IV Q4W

[c] Nivolumab dosing:

- ≥ 18 years of age **OR** ≥ 12 years to < 18 years of age and ≥ 40 kg: nivolumab 480 mg IV Q4W
- ≥ 12 years to < 18 years of age and < 40 kg: nivolumab 6 mg/kg IV Q4W

2.2 Treatment Assignment

All participants will be centrally randomized using Interactive Response Technology (IRT). Before the study is initiated, each user will receive log-in information and directions on how to access the IRT. After the participant's informed consent has been obtained and initial eligibility is established, the participant must be enrolled into the study by using IRT to obtain the participant number. Every participant who signs the ICF must be assigned a participant number in IRT. The investigator or designee will register the participant for enrollment by following the enrollment procedures established by BMS. The following information is required for enrollment:

- Date that informed consent was obtained
- Year of birth
- Gender at birth

After enrollment in the IRT, participants who have met all eligibility criteria will be randomized through the IRT. The following information is required for participant randomization:

- Participant number
- Year of birth
- Melanoma type (cutaneous or mucosal)
- AJCC v8 stage at screening (cutaneous melanoma only)

Participants will be randomized in a 1:1 ratio (see Figure 2.1-1) and stratified by

- Geographic region (USA/Canada/Australia vs Europe vs ROW(Rest of the World))
- American Joint Committee on Cancer (AJCC v8) Stage IIIA/IIIB vs Stage IIIC vs Stage IIID/IV (including all participants with mucosal melanoma, Stage III, Stage IVA, Stage IVB, and Stage IVC – see protocol Appendix 8)

Stratification levels and Descriptions:

**Stratum Code**	**Stratum Description**
1	USA/Canada/Australia, Stage IIIA/IIIB
2	USA/Canada/Australia, Stage IIIC
3	USA/Canada/Australia, Stage IIID/IV
4	Europe, Stage IIIA/IIIB
5	Europe, Stage IIIC
6	Europe, Stage IIID/IV
7	ROW, Stage IIIA/IIIB
8	ROW, Stage IIIC
9	ROW, Stage IIID/IV

Approved v2.0 930199387 2.0

The randomization procedures will be carried out via permuted blocks within each stratum, defined by combination of geographic region and AJCC v8 stage. The exact procedures for using the IRT will be detailed in the IRT manual.

2.3 Blinding and Unblinding

This is a double-blind study. Access to treatment codes will be restricted from all participants, and site and Sponsor personnel prior to primary database lock, with exceptions as specified below.

Randomization schedules will be shipped directly to a pharmacist or other individual(s) who will be responsible for the dispensing of blinded study intervention. This (these) individual(s) will be unblinded to study intervention identification but will not be involved in any other aspect of study conduct. The randomization schedules will be maintained in a secure location with access limited to authorized personnel.

Blinding of study treatment assignment is critical to the integrity of this clinical study. However, in the event of a medical emergency or pregnancy in an individual participant in which knowledge of the IP is critical to the participant's management, the blind for that participant may be broken by the investigator. The participant's safety takes priority over any other considerations in determining if a treatment assignment should be unblinded.

In cases of accidental unblinding, contact the Medical Monitor/designee and ensure every attempt is made to minimize additional disclosure and the impact of unblinding. Any request to unblind a participant for nonemergency purposes should be discussed with the Medical Monitor/designee. Discussions regarding unblinding with the Medical Monitor/designee must be documented. In case of an emergency, the investigator(s) has unrestricted access to randomization information via the IRT and is capable of breaking the blind through the IRT system without prior approval from the Sponsor. Following the unblinding, the Investigator shall notify the Medical Monitor/designee that the unblinding took place.

A scientist in the NonClinical Disposition and Bioanalysis department of BMS (and/or a designee in the external bioanalytical laboratory) will be unblinded to the randomized treatment assignments in order to minimize unnecessary bioanalytical analysis of samples. Any results shared by the NonClinical Disposition and Bioanalysis group with the Sponsor's study team will be blinded to ensure integrity of the study.

Investigators will remain blinded to each participant's assigned study intervention throughout the course of the study. In order to maintain this blind, an otherwise uninvolved third party will be responsible for the reconstitution and dispensation of all study intervention and will endeavor to ensure that there are no differences in time taken to dispense following randomization.

In the event of a Quality Assurance audit, the auditor(s) will be allowed access to unblinded study intervention records at the site(s) to verify that randomization/dispensing has been done accurately.

The DMC will assess safety and risk-benefit on an ongoing basis and will have access to unblinded treatment codes for individual participants. An external analysis team (external to BMS), including a reporting statistician and programming support, who are not involved with the conduct of the study, will provide analyses to the DMC. The procedures to be respected and the reasons for

Approved v2.0 930199387 2.0

unblinding of the DMC are discussed in the DMC charter. The DMC will perform all interim analyses of RFS and OS prior to unblinding. The sponsor will be unblinded to individual treatment assignments at the time of any interim RFS analysis found to be statistically significant (performed by DMC) or at the time of the final RFS (performed by BMS), whichever comes first. Participants, investigator, and site staff will remain blinded to individual treatment assignments until OS result is statistically significant or until final analysis of the OS endpoint, whichever comes first. The sponsor will remain blinded to OS summary analysis by treatment arm until OS is significant or until the time of the final OS, whichever comes first.

See Section 5.1 for timing of the RFS analyses and Section 5.2 for timing of OS analyses.

2.4 Protocol Amendments

As described in protocol amendments 01 and 02, duration of treatment on next-line therapy has been downgraded to an exploratory endpoint. Table 2.4-1 summarizes these changes:

Table 2.4-1: Amendments to the protocol / primary and secondary endpoints

Amendment which removed/added endpoint	Rationale for addition, removal of endpoint / downgrading to exploratory endpoint	Planned analysis based on data collected
Amendment #02	One component of the secondary objective regarding outcomes on next-line therapies (ie, duration of treatment on next-line therapies) was moved to a new exploratory objective. Since duration of treatment on next line of therapies is valuable in the context of payor perspectives accounting for variation in clinical practice, this was moved to an exploratory endpoint.<br>Also added to this new exploratory objective was time to next line of therapy. The other component of this secondary objective (ie, PFS2) remains a secondary objective.	Duration of treatment on next-line therapy and time to next treatment will be described in greater details in an exploratory SAP. They will not be included in the CSR.<br>The estimate of the PFS2 hazard ratio by treatment group will be calculated using a stratified Cox proportional hazards model. The PFS2 distribution for each treatment group will be estimated using Kaplan-Meier techniques.
Amendment #01	Distant metastasis-free survival (DMFS) was moved to other secondary endpoints and is no longer a key secondary endpoint that will be tested formally. OS will be tested on the statistical hierarchy and remains a key secondary endpoint.	To maximize the chance of testing for a potentially positive OS, which has more significant clinical and regulatory impact.<br>The analysis for DMFS will be conducted using a stratified Cox proportional hazards model, with treatment as the single covariate. The DMFS distribution for each treatment group will be estimated using Kaplan-Meier techniques.

There have been 3 protocol amendments with the following changes that affect the analyses, as described below.

Approved v2.0 930199387 2.0

Summary of key changes for PROTOCOL AMENDMENT 03		
**Section Number & Title**	**Description of Change**	**Brief Rationale**
Protocol Summary	Text has been updated to align with changes made throughout the protocol.	Alignment between Protocol Summary and body of the protocol.
Table 2-2: On Study Treatment Procedural Outline (CA224098)<br>Table 2-3: Follow-up Assessments (CA224098)<br>Table 9.8-1: Biomarker Sampling Schedule: All Arms	• Corrected optional Biomarker Sample Collection to apply upon occurrence of ≥ Grade 3 drug-related AE.<br>• Corrected notes to state that biomarker sample collection is required at Follow-Up Visit 2 and then every 26 weeks (ie, at every alternating Survival Visit ± 90 days), but not to exceed 272 days between collections, thereafter to end of study or first recurrence, whichever occurs first.	• To correct typographical errors.<br>• To clarify the timing of the biomarker sample collection in relation to Survival Follow-up visits.
Table 4-1: Objectives and Endpoints	• Rephrased "next-line therapies" as "next systemic therapies" and deleted "next line of therapy" in the endpoint description for time to next treatment.<br>• Updated and provided a more accurate endpoint for the evaluation of participant's overall health status using the EQ-5D-5L index and visual analog scale objective.	To clarify and align with the intent of analysis.
Section 10.2.1: Recurrence-free Survival	• Corrected the nominal significance level for the first interim recurrence-free survival (RFS) analysis from 0.024 to 0.020.<br>• Corrected the critical hazard ratio for the final RFS analysis from 0.82 to 0.815.	• To correct a typographical error of the nominal significance level.<br>• To ensure the same precision (number of digits) is presented for the critical hazard ratio of the final RFS analysis across the document.
Section 10.3: Analysis Sets	Clarified that the intercurrent event strategy for the analysis sets for main estimand of recurrence-free survival (RFS), distant metastasis-free survival (DMFS), and overall survival (OS) will be provided in Table 10.4.1-2.	To streamline the definitions for the analysis data sets.
Section 10.4.1: General Considerations	Statement regarding melanoma in situ not being considered a new primary melanoma moved to a footnote in Table 10.4.1-1.	To be consistent with the definition of RFS in Section 4.

**Summary of key changes for PROTOCOL AMENDMENT 03**		
**Section Number & Title**	**Description of Change**	**Brief Rationale**
Table 10.4.1-2: Definition of Estimands for Primary and Secondary Endpoints	• Clarified estimand definitions.<br>• Clarified the intercurrent event strategy for melanoma in situ, second non-melanoma primary cancer, and non-invasive basal cell carcinoma/squamous cell carcinoma with strategy equal to treatment policy.<br>• Updated population level summaries for RFS, OS and DMFS<br>• Updated Intercurrent Events for PFS2 (Strategy) to state that details are presented in the statistical analysis plan.<br>• Added Intercurrent Events (Strategy) for RFS, OS, DMFS and safety.<br>• Removed summary statistics bullet as this will be further detailed in the statistical analysis plan.	To clarify and align with the intent of analysis and clarify that details are presented in the statistical analysis plan.
Table 10.4.1-2: Definition of Estimands for Primary and Secondary Endpoints<br>Section 10.4.2: Primary Endpoint(s)	Updated the phrase "secondary non-melanoma primary malignancy" to "second non-melanoma primary cancer").	To ensure accuracy and consistency of terminology.
Section 10.4.2: Primary Endpoints(s)	Clarified censoring rules for the supplemental estimand.	To clarify and align with the intent of analysis.
Table 10.4.2-1: Primary Endpoints<br>Table 10.4.3-1: Secondary Efficacy Endpoints	Clarified timeframes for analysis for primary and secondary efficacy endpoints.	To clarify and align with the intent of analysis.
Throughout	Editorial updates.	Minor; therefore, have not been summarized.

Approved v2.0 930199387 2.0

SUMMARY OF KEY CHANGES FOR PROTOCOL AMENDMENT 02

Section Number & Title	Description of Change	Brief Rationale
Section 10.2.1: Recurrence-free Survival<br>Section 10.2.2: Overall Survival<br>Table 10.2.2-1: Formal Analysis of Overall Survival - Operating Characteristics at Each Time Point<br>Section 10.3: Analysis Sets<br>Table 10.4.2-1: Primary Endpoints<br>Section 10.5.1: Interim Analyses of RFS<br>Section 10.5.2: Interim Analysis of OS	Updated the interim analyses to be event-based rather than time-based. Also revised text and tables regarding projected accrual rates, estimated number of months expected for follow-up at the time of the interim RFS analyses, and corrected cumulative power for both interim analyses.<br>Made adjustments to expected timing of OS events at the interim analyses based on the new projections for RFS interims and final analyses.	To adjust for shortened randomization timelines and corrected a typo regarding the estimate of power at both RFS interim analyses.
	Clarified the condition under which RFS IA#2 may be skipped.	To ensure that no 2 RFS analyses are within 6 months of each other.
Section 10.3: Analysis Sets<br>Section 10.4.6.3: Patient-Reported Outcomes	Removed PRO analysis population	To clarify population sets that are analyzed.
Section 9.1.4.3: Definition of Recurrence<br>Table 9.8-1: Biomarker Sampling Schedule: All Arms	Excluded MMIS in defining recurrence as appearance of new melanoma lesions.	To clarify recurrence definition.
Table 4-1: Objectives and Endpoints<br>Table 10.4.1-2: Definition of Estimands for Primary and Secondary Endpoints<br>Table 10.4.3-2: Summary of Secondary Endpoint Analysis	Part of the secondary objective regarding outcomes on next line of therapy was moved to an exploratory objective. Duration on next line of therapy was moved to a new exploratory objective, and time to next line of therapy was added. The other component of this secondary objective (ie, PFS2) remains a secondary objective.	Since duration of treatment on next line of therapies is valuable in the context of payor perspectives accounting for variation in clinical practice, this was moved to an exploratory endpoint and updated to clarify that it is next systemic therapy.
	Removed exploratory objective and endpoint regarding time to confirmed deterioration in health-related quality of life (HRQoL).	To align with current research plan and reduce analytic burden.

Approved v2.0 930199387 2.0

Table 4-1: Objectives and Endpoints<br>Section 10.3: Analysis Sets<br>Table 10.4.1-2: Definition of Estimands for Primary and Secondary Endpoints	Population for the distant metastasis-free survival (DMFS) secondary objective was limited to randomized participants with Stage III/IVA/IVB no evidence of disease (NED) melanoma.	To align with prior adjuvant studies CM238 and CM915 as well as clinical relevance of occurrence of distant metastasis in Stage III disease rather than Stage IV (non-mucosal).
Table 10.4.1-1: Censoring Scheme for Definition of Recurrence-free Survival	Added row for baseline disease as censored beginning at date of randomization. Removed second non-melanoma primary cancer from the censoring algorithm.	To clarify censoring scheme.

Approved v2.0 930199387 2.0

SUMMARY OF KEY CHANGES FOR PROTOCOL AMENDMENT 01		
**Section Number & Title**	**Description of Change**	**Brief Rationale**
Table 4-1: Objectives and Endpoints<br>Section 10.1: Statistical Hypotheses<br>Section 10.3: Analysis Sets<br>Section 10.4.1: General Considerations<br>Table 10.4.1-2: Definition of Estimands for Primary and Secondary Endpoints<br>Table 10.4.3-1: Secondary Efficacy Endpoints<br>Table 10.4.3-2: Summary of Secondary Endpoint Analysis	Distant metastasis-free survival (DMFS) was moved to other secondary endpoints and is no longer a key secondary endpoint that will be tested formally.<br>OS will be tested on the statistical hierarchy and remains a key secondary endpoint.	To maximize the chance of testing for a potentially positive OS, which has more significant clinical and regulatory impact.
Table 4-1: Objectives and Endpoints<br>Section 5.5.3: Rationale for Continued Adjuvant Therapy for Patients Who Are Diagnosed with Melanoma in Situ During Study Treatment<br>Section 10.4.1: General Considerations<br>Table 10.4.1-1: Censoring Scheme for Primary Definition of Recurrence-free Survival<br>Table 10.4.2.1-1: Primary Endpoints<br>Table 10.4.2.1-2: Summary of Primary Endpoint Analysis.	Melanoma in situ will no longer be considered as a new primary cancer and consequently will no longer be considered an event for the primary endpoint of RFS.	Recurrence rates for completely excised melanoma in situ are sufficiently low that patients are considered cured following excision, with the exception of certain subtypes may recur locally (i.e., lentigo maligna)
Table 4-1: Objectives and Endpoints	Objective Response Rates were removed as an exploratory biomarker endpoint.	Objective response rate is not applicable for a study with adjuvant treatment.
Table 4-1: Objectives and Endpoints	LAG-3 was included in an exploratory endpoint for an objective to evaluate changes in the tumor microenvironment and peripheral, indicative of immunomodulatory effect.	Elaboration of exploratory biomarkers in tumor microenvironment under study included.

Approved v2.0 930199387 2.0

SUMMARY OF KEY CHANGES FOR PROTOCOL AMENDMENT 01		
**Section Number & Title**	**Description of Change**	**Brief Rationale**
Section 5.1: Overall Design<br>Section 5.4.7: Rationale for Inclusion of Adolescent Participants<br>Section 5.5.4 : Dose Rationale of Adolescent Participants<br>Table 7.1-1: Study Interventions	Adolescent participants ≥ 12 years are now eligible to participate in the study. The protocol has been updated throughout to include pediatric dosing information, which for a subset of pediatric participants will be weight-based.<br>Rationale for the inclusion of adolescent participants and dosing rationale for this population have been added.	Addition of adolescent participants.<br><br>Addition of dosing for adolescent participants aged ≥ 12 years to 18 years, including weight-based differences for this group of participants.
Section 5.1.1: Data Monitoring Committee and Other Committees<br>Section 10.1.1: Multiplicity Adjustment	Added a paragraph describing the administrative alpha penalty if the Data Monitoring Committee (DMC) requests unplanned look efficacy data prior the RFS IA.	Clarification of alpha administrative penalty that will occur if DMC requests unplanned look at efficacy data prior to RFS IA.
Figure 5.1-1: Study Design Schema	The study design schema and associated footnotes were updated per the changes to Protocol Amendment 01.	Addition of dosing for pediatric participants for nivo + rela FDC and nivolumab only added to study schema including footnotes.
Section 7.3: Blinding	Details regarding DMC access to unblinded treatment codes, and the timing of participant, investigator, site staff, and sponsor access to unblinded treatment assignments has been included.	Clarifying information regarding timing of unblinding for DMC, sponsor, participants, investigator, site staff, and sponsor access to unblinded treatment assignments is added.
Section 9.1.2, Imaging Assessment for the Study	Removed a statement that assessments should be performed by the same investigator or delegate at all timepoints.	To reduce site burden of having the same investigator perform the assessment at all timepoints.
Section 9.1.4: Investigator Assessment of Recurrence<br>Section 9.1.4.3: Definition of Recurrence	Removed malignant melanoma in situ (MMIS) from definition of recurrence as is not considered recurrence. Date of MMIS will be captured still to allow for future sensitivity analysis.	Clarification that MMIS is not considered recurrence.

Approved v2.0 930199387 2.0

SUMMARY OF KEY CHANGES FOR PROTOCOL AMENDMENT 01		
**Section Number & Title**	**Description of Change**	**Brief Rationale**
Section 10.1.1 Multiplicity Adjustment	Changed the statistical hierarchy to remove DMFS. The primary end point is RFS and the secondary is OS.	To maximize the chance to test OS earlier for a more significant regulatory & clinical impact- DMFS was removed as a primary endpoint to other secondary endpoint.
Section 10.2.1: Recurrence-Free Survival	The approximate time from first participant randomization to the required observed RFS events was modified from 56 to 59 months.	Change is result of a different method of simulation after adding new interim analysis.
Section 10.2.1: Recurrence-Free Survival<br>Table 10.4.2-1: Primary Endpoints<br>Section 10.5.1:<br>Interim Analysis of RFS	Addition of a new interim analyses (IA) of time based RFS, 8 months after the last patient is randomized.	An earlier interim analysis of RFS was added to provide an earlier opportunity for evaluation of treatment effect and is time based.
Section 10.2.1: Recurrence-Free Survival<br>Section 10.5.3: Interim Analyses of RFS and OS: Additional Details	To avoid frequent renumbering, the version number for the statistical tool, EAST, was removed.	Version number was removed to allow for flexibility.
Section 10.2.2: Overall Survival<br>Section 10.5.2: Interim Analysis of OS<br>Section 10.5.3: Interim Analyses of RFS and OS: Additional Details	Multiple changes were made in these sections:<br>• Details regarding DMFS analyses were removed.<br>• Updated information was used from CA209238 5-Year survival for the calculations of OS sample size and power. Details added regarding addition of interim OS analyses were included.<br>Final OS was changed to Year 8.	DMFS is no longer formally tested, and thus removed corresponding sections.<br>The target hazard ratio, expected number of deaths, and final timing of OS were all updated.<br>Interim OS analyses were added to provide an opportunity for evaluation of the treatment effect.
Appendix 6: ECOG and Lansky/Karnofsky Performance Status Scale	Lansky/Karnofsky performance status scale has been added.	Addition of performance assessment scale for pediatric participants.

2.5 Data Monitoring Committee and Other Committees

When required, adjudicated events will be submitted to the Data Monitoring Committee (DMC) and Health Authorities for review on a specified timeframe in accordance with the adjudication documentation.

Approved v2.0 930199387 2.0

A DMC will be established to provide oversight of safety and efficacy considerations and to provide advice to the Sponsor regarding actions the committee deems necessary for the continued protection of participants enrolled in the study. The DMC will be charged with assessing such actions in light of an acceptable benefit/risk profile for nivo + rela FDC. The DMC will act in an advisory capacity to BMS and will monitor participant safety and evaluate the available efficacy data for the study. The oncology therapeutic area of BMS has primary responsibility for design and conduct of the study.

The efficacy is not planned to be provided at regular DMC meetings. If the DMC requests efficacy summary data in order to perform benefit/risk assessment prior to the RFS IAs, then the efficacy will be available at the request of the DMC. The availability of efficacy will not be automatically carried over to the next DMC meeting unless the DMC requests efficacy again at the subsequent meeting. An administrative alpha penalty will occur and will be subtracted from the overall type I error of 0.05 for each additional unplanned look requested by the DMC.

Details of the DMC responsibilities and procedures will be outlined in the DMC charter.

A Study Steering Committee (SSC), consisting of Investigators and personnel members representing the Sponsor of the study, will be established to obtain scientific guidance and advice for the protocol and conduct of the study. Details of the SSC responsibilities and procedures will be specified in the SSC charter.

3 OBJECTIVES

The study objectives are defined in the following sections. Consistent with the ICH E9 Addendum, the definition of the study estimands for the primary and key secondary objectives are described in Table 3-1.

Table 3-1: Study Objectives and Endpoints

Objective	Endpoints
**Primary**	
To compare the efficacy, as measured by RFS, provided by nivo + rela FDC vs nivolumab monotherapy in participants with completely resected Stage III/IV NED melanoma.	RFS time as assessed by the investigator. RFS is defined as the time between the date of randomization and the first date of documented recurrence (local, regional, distant, new primary melanoma) or death due to any cause, whichever occurs first.
**Key Secondary**	
To compare the OS provided by nivo + rela FDC vs nivolumab monotherapy in participants with completely resected Stage III/IV NED melanoma.	OS is defined as the time between the date of randomization and the date of death due to any cause.
**Other Secondary**	

Table 3-1: Study Objectives and Endpoints

Objective	Endpoints
To assess the efficacy, as measured by distant metastasis-free survival (DMFS), provided by nivo + rela FDC vs nivolumab monotherapy in participants with completely resected Stage III/ IVA/IVB NED melanoma.	DMFS, by investigator, is defined as the time between the date of randomization and the date of first distant metastasis or date of death due to any cause, whichever occurs first.
To assess safety and toxicity of nivo + rela FDC vs nivolumab monotherapy in participants with completely resected Stage III/IV NED melanoma.	Incidence and severity of AE, SAEs, AEs leading to DC, IMAEs, drug-related AEs, deaths, laboratory abnormalities, and other select AEs.
To evaluate investigator-assessed outcomes on next-line therapies.	PFS2 defined as time from randomization to second recurrence/objective disease progression on next-line systemic therapy per investigator, or death from any cause, whichever occurs first.
**Exploratory**	
To characterize the pharmacokinetics of nivo + rela FDC and nivolumab monotherapy.	End of infusion concentration and trough concentrations.
To characterize the immunogenicity of nivo + rela FDC and nivolumab monotherapy	Immunogenicity assessed by anti-relatlimab and anti-nivolumab antibodies.
To evaluate additional investigator-assessed outcomes on next systemic therapies.	Duration of treatment on next-line therapies.<br>Time to next treatment.
To evaluate freedom from recurrence defined as the time from randomization to recurrence or death, with censoring of data for participants who had died from causes other than melanoma or treatment-related toxic effects.	Freedom from recurrence.
To evaluate TFI defined as the time from last dose of study treatment to the start of subsequent systemic therapy or death.	Treatment free interval.
To explore potential association of biomarkers (e.g., LAG-3 expression, PD-L1 expression, BRAF mutation status, TMB, genomic signatures soluble biomarkers, etc.) with clinical efficacy (RFS, DMFS, and OS) and/or incidence of adverse events of nivolumab and/or relatlimab by analyzing biomarker measures within the tumor microenvironment and periphery (e.g., blood, serum, plasma, tumor tissue and PBMCs) in comparison to clinical outcomes.	Association of biomarkers to RFS, DMFS, OS, incidence of AEs and LAG-3, PD-L1, BRAF mutation status, TMB, genomic signatures, soluble biomarkers, etc.
To explore the role of ctDNA to understand MRD and disease recurrence predictability.	Association of recurrence with ctDNA positivity and dynamic changes in levels.
To evaluate changes in the tumor microenvironment and periphery indicative of immunomodulatory effect.	Changes of PD-L1 and LAG-3, gene expression, and other biomarkers in the TME and periphery.
To assess the impact of SARS-CoV-2 serologic status on participants receiving nivo + rela FDC and nivolumab monotherapy in melanoma participants and to support health authority requests.	Exploratory measurements of SARS-CoV-2 serology (anti-SARS-CoV-2 total or IgG), from serum samples collected at baseline and the potential association between these measurements and selected endpoints related to safety, efficacy, and/or biomarkers

Approved v2.0 930199387 2.0

Table 3-1: Study Objectives and Endpoints

Objective	Endpoints
To assess the participant's cancer-related QoL using the EORTC QLQ-C30.	Mean changes from baseline in scores and proportion of participants achieving clinically meaningful changes in EORTC QLQ-C30 scores for functional scales, symptom scales, and global health status/QoL scale at all post-baseline assessments..
To characterize participant perceptions of the bothersomeness of symptomatic AEs, based on FACIT-GP5 item.	Summary changes and frequency of responses in FACIT-GP5 item measuring bother due to side effects of treatment.
To evaluate the participant's overall health status using the EQ-5D-5L index and visual analog scale	Mean change from baseline in VAS and utility index and proportion of participants achieving clinically meaningful changes in scores to all post-baseline assessments in EQ-5D-5L in both the visual analog scale and the utility index.
To characterize health care resource utilization.	Hospitalizations, non-protocol specified medical visits, diagnostics.

Abbreviations: AE(s), adverse event(s); BRAF, B-RAF proto-oncogene; ctDNA, circulating-tumor deoxyribose nucleic acid; DC, discontinuation; DMFS, distant metastasis-free survival; EORTC QLQ-C30, European Organization for the Research and Treatment of Cancer Quality of Life Questionnaire; FACIT-GP5, Functional Assessment of Chronic Illness Therapy-Item GP5; FDC, fixed dose combination; HRQoL, health-related quality of life; IgG, immunoglobulin G; LAG-3, lymphocyte activation gene 3; MRD, minimal residual disease; NED, no evidence of disease; nivo, nivolumab; PBMC, peripheral blood mononuclear cell; PD-L1, programmed death-ligand 1; PFS2, progression-free survival 2; QoL, quality of life; RFS, recurrence-free survival; OS, overall survival; rela, relatlimab; SAE, serious adverse event; SARS-CoV-2, severe acute respiratory syndrome coronavirus 2; TMB, tumor mutation burden; TME, tumor microenvironment; vs, versus.

4 ENDPOINTS

4.1 Efficacy Endpoints

The primary and secondary endpoints are detailed below. Further information can be found in the estimand table in Section 4.4.

4.1.1 Recurrence-Free Survival (RFS)

4.1.1.1 Definition of Recurrence-Free Survival

RFS will be programmatically determined based on the disease recurrence date provided by the investigator and is defined as the time between the date of randomization and the date of first recurrence (local, regional [including in-transit or regional nodal recurrence], or distant metastasis), new primary invasive melanoma (melanoma in situ [MMIS] is not considered a new primary), or death (whatever the cause), whichever occurs first.

For participants who remain alive and whose disease has not recurred, RFS will be censored on the date of last evaluable disease assessment. For those participants who remained alive and had no recorded post-randomization tumor assessment, RFS will be censored on the day of randomization. Censoring rules for the endpoint of RFS are presented in Table 4.1.1.1-1 and Figure 4.1.1.1-1.

Approved v2.0 930199387 2.0

Table 4.1.1.1-1: Censoring Scheme for Definition of Recurrence-Free Survival

Situation	Date of Event or Censoring	Outcome
No baseline disease assessment	Date of randomization	Censored
No on-study disease assessments and no recurrence / death	Date of randomization	Censored
Disease at baseline	Date of randomization	Censored
No recurrence and no death	Date of last evaluable disease assessment	Censored
Recurrence (local, regional, distant, new primary invasive melanoma)*	Date of first recurrence	Event
Death without recurrence	Date of death	Event

* Regional recurrence includes in-transit or regional nodal recurrence. New primary invasive melanoma does not include in situ melanoma as an event.

Approved v2.0 930199387 2.0

Figure 4.1.1.1-1: RFS Definition - Main Estimand

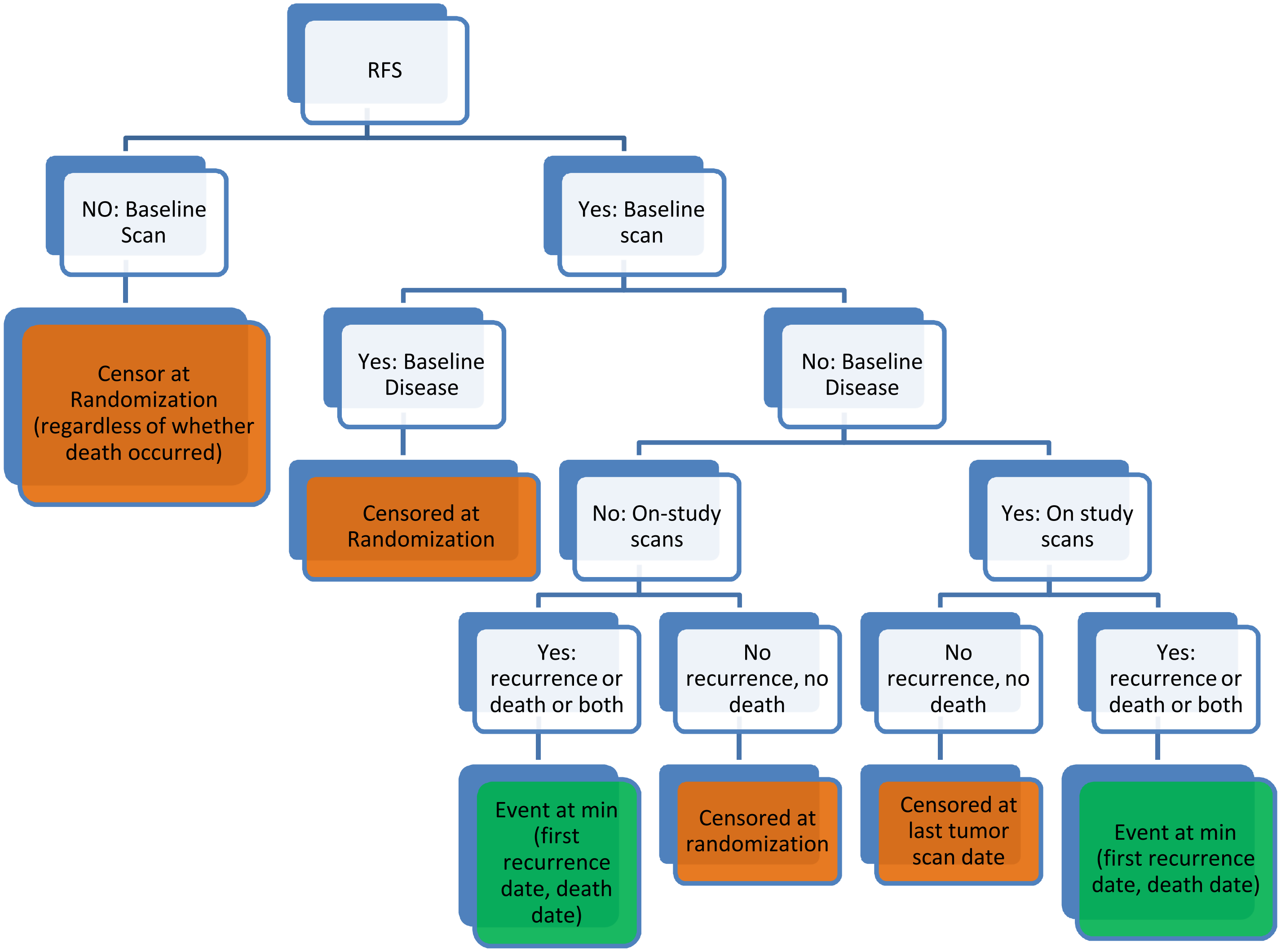

4.1.2 Overall Survival

Overall survival (OS) is defined as the time from randomization to the date of death from any cause. For participants that are alive, their survival time will be censored at the date of last contact (or "last known alive date"). Overall survival will be censored at the date of randomization for participants who were randomized but had no follow-up.

OS rate at time *T* (at fixed time points, e.g., at yearly interval, depending on the minimum follow-up) is defined as the probability that a participant is alive at time *T* following randomization.

Survival follow-up will be conducted every 12 weeks (± 14 days) during the survival follow-up period.

4.1.3 Distant Metastasis-Free Survival (DMFS)

The definition of Distant Metastasis-Free Survival (DMFS) is defined as the time between the date of randomization and the date of first recurrence of distant metastasis or the date of death due to any cause, whichever occurs first. For participants who remain alive and distant metastasis-free, DMFS will be censored on the date of last evaluable disease assessment. For those participants who remain alive and have no recorded post-randomization disease assessment, DMFS will be censored on the day of randomization.

DMS will be analyzed on randomized participants with either Stage III non-mucosal melanoma or with Stage III, Stage IVA, Stage IVB mucosal melanoma at study entry.

The censoring rules are similar to RFS definition censoring rules described in Section 4.1.1.1.

4.1.4 Progression-Free Survival on Next-Line Therapy (PFS2)

PFS on next-line therapy (PFS2) is defined as time from randomization to second recurrence/objective disease progression on next-line systemic therapy per investigator, or death from any cause, whichever occurs first. In other words, it is calculated as the time from randomization to documented recurrence (clinical/radiological) after the start of subsequent systemic anti-cancer therapy (SST) (following a 1st recurrence) or to death from any cause, whichever occurs first.

The following censoring rules will be applied for PFS2:

- Participants who do not have a documented disease-free status prior to randomization will be censored at randomization.
- For participants who did not receive SST (or SST only before first recurrence/disease progression):
  - Participants who developed a second non-melanoma primary cancer will be censored at the date of diagnosis of second non-melanoma primary cancer
  - Otherwise,
    - Participants who died will be considered as having the event on the date of death.
    - Participants who did not die will be censored at the last known alive date.
- For participants who received SST after first recurrence/disease progression*:

Approved v2.0 930199387 2.0

- Participants with a recurrence/disease progression after the start of subsequent systemic anti-cancer therapy will be:
    - ♦ considered as having the event on the date of recurrence/objective disease progression.
    - ♦ otherwise, censored at the date of diagnosis of second non-melanoma primary cancer if occurred before the recurrence/progression.
- Participants who did not have recurrence/disease progression after the start of subsequent systemic anti-cancer therapy will be:
    - ♦ censored at the date of diagnosis of second non-melanoma primary cancer if they developed a second non-melanoma primary cancer.
    - ♦ considered as having an event at the date of death if they died but did not develop a second non-melanoma primary cancer.
    - ♦ censored on the last known alive date otherwise.

<u>Notes:</u>

* if the date of recurrence/progression after SST missing in the eCRF:

- The end date of SST should be an event when reason for stopping the SST is marked as due to disease progression
- If end date of the discontinued SST is missing, the start date of the next SST (-1) will count as an event
- Any other reason for stopping SST should not be counted as an event (i.e., ignored), including missing reasons for stopping SST.

Second non-melanoma primary cancer is excluding non-invasive BCC/SCC.

Approved v2.0 930199387 2.0

Figure 4.1.4-1: PFS2 Definition

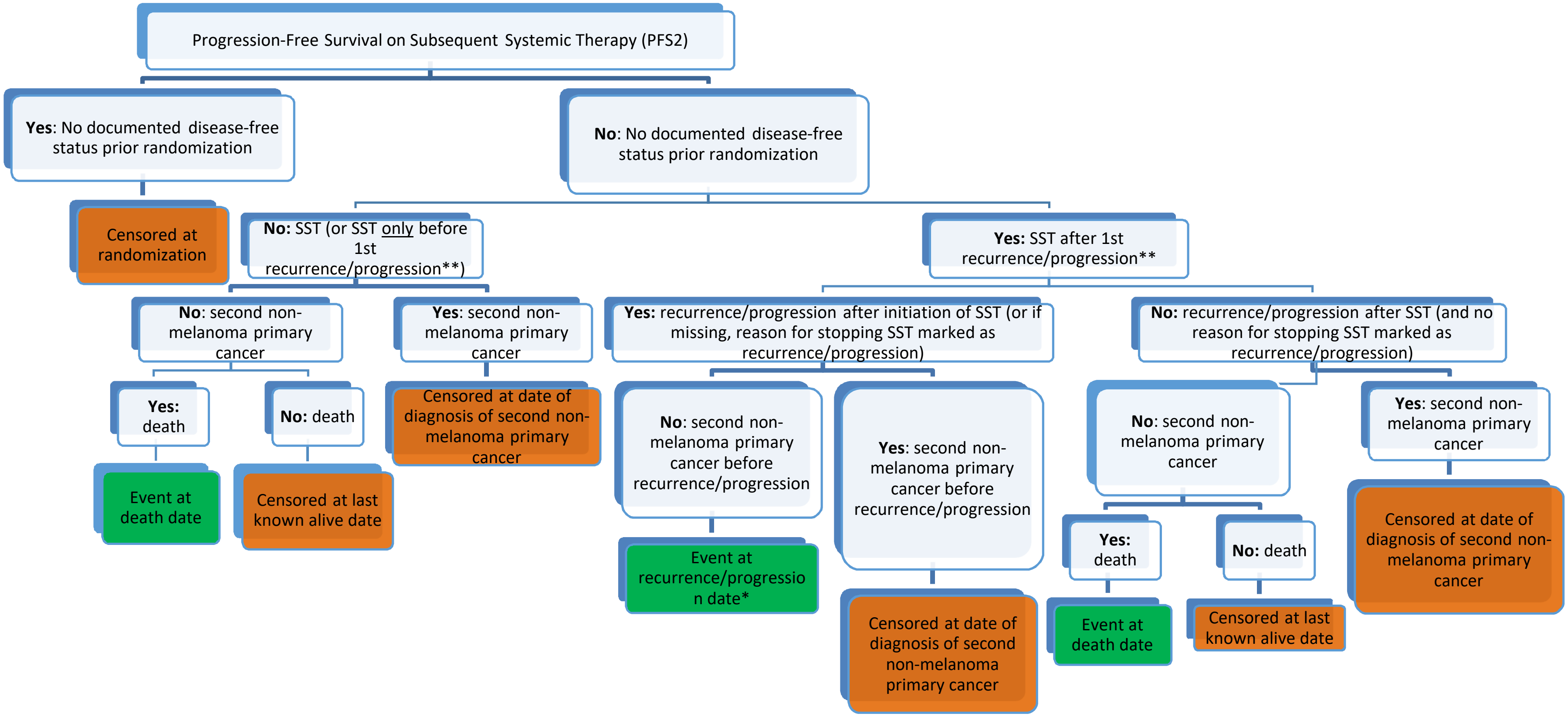

** The first ever recurrence/disease progression should be based on the data in the progression/recurrence eCRF page [and can be non-radiographic (e.g., clinical, or histopathologic) or radiographic].

SST in the figure pertains to subsequent systemic anti-cancer therapy. Second non-melanoma primary cancer is excluding non-invasive BCC/SCC.

4.1.5 Duration of Treatment on Next-Line Therapy

Duration of Treatment on Next-Line Therapy is an exploratory endpoint and will be detailed in an exploratory SAP. It will not be included in the CSR.

4.1.6 Time to Next Treatment

Time to Next Treatment (TTNT) is defined as the time from randomization to next systemic therapy or death, whichever comes first. The definition of next systemic therapy is any systemic anti-cancer therapy for the cancer under study with a start date on or after the date of discontinuation of study drug (randomization date if subject was never treated).

TTNT will be described in greater details in an exploratory SAP but will not be included in the CSR.

4.1.7 Treatment-Free Interval (TFI)

Treatment-Free Interval (TFI) is defined only in randomized participants who are off study treatment and continued to be followed in study prior to initiation of subsequent systemic therapy. TFI is defined as time from end of study therapy until last known alive date in those who never received subsequent systemic therapy and defined as time from end of study therapy until subsequent systemic therapy in those who received subsequent systemic therapy.

TFI will be described in greater details in an exploratory SAP but will not be included in the CSR.

4.1.8 Freedom from Recurrence

Freedom from recurrence (FFR) is defined as the time between the date of randomization and the date of first recurrence or the date of death due to melanoma or treatment-related toxic effects, whichever occurs first. Same rules for RFS as in the primary endpoint analysis will be used, except for a change in the criteria for when to include a death. Patients who die from causes other than melanoma or treatment-related toxic effects will be censored to death date. Otherwise, same censoring rules will be applied as the definition for RFS, see section 4.1.1.1.

FFR will be described in greater details in an exploratory SAP but will not be included in the CSR.

4.1.9 Clinical Outcomes Assessments

The COA analyses detailed within this SAP include the EQ-5D-5L to assess general health status and self-reported health preference; the European Organisation for Research and Treatment of Cancer Quality of Life Questionnaire (EORTC QLQ-C30) questionnaire to assess the effect of cancer on participants’ health-related quality of life (HRQoL), and the Functional Assessment of Cancer Therapy (FACIT-GP5) to assess the extent of perceived bother due to treatment-related effects.

Participants who are ≥ 18 years of age at the time of informed consent complete EQ-5D-5L, EORTC QLQ-C30 and FACIT-GP5 questionnaires. Adolescent participants (≥ 12 and < 18 years of age) only complete the EQ-5D-5L.

Approved v2.0 930199387 2.0

4.1.9.1 EORTC QLQ-C30

Health-related quality of life (HRQoL) will be assessed using the EORTC QLQ-C30 questionnaire Version 3. It is a 30-item instrument that has gained wide acceptance in oncology clinical studies. The EORTC QLQ-C30 is composed of multi-item and single scales. These include five functional scales (physical, role, emotional, social, and cognitive), three symptom scales (fatigue, nausea and vomiting and pain), a global health status/Quality of Life (GHS/QOL) scale, and six single items (dyspnea, insomnia, appetite loss, constipation, diarrhea, and financial difficulties). All scales and single items show good reliability. The reliability and validity of the questionnaire is highly consistent across different language-cultural groups[1]. Except for the overall health status and global QOL items, responses for all items are 4-point categorical scales ranging from 1 (Not at all) to 4 (Very much). The GHS/QOL responses are 7-point Likert scales.

Data will be scored according to the algorithm described in the EORTC QLQ-C30 scoring manual, as follows:

Functional scales:

- Physical functioning: (1 - ((Q1+Q2+Q3+Q4+Q5)/5-1)/3) * 100
- Role functioning: (1 - ((Q6+Q7)/2-1)/3) * 100
- Emotional functioning: (1 - ((Q21+Q22+Q23+Q24)/4-1)/3) * 100
- Cognitive functioning: (1 - ((Q20+Q25)/2-1)/3) * 100
- Social functioning: (1 - ((Q26+Q27)/2-1)/3) * 100

Global health status:

- GHS/QoL: ((Q29+Q30)/2-1)/6 * 100

Symptom scales/items:

- Fatigue: ((Q10+Q12+Q18)/3-1)/3 * 100
- Nausea and vomiting: ((Q14+Q15)/2-1)/3 * 100
- Pain: ((Q9+Q19)/2-1)/3 * 100
- Dyspnea: (Q8-1)/3 * 100
- Insomnia: (Q11-1)/3 * 100
- Appetite loss: (Q13-1)/3 * 100
- Constipation: (Q16-1)/3 * 100
- Diarrhea: (Q17-1)/3 * 100
- Financial difficulties: (Q28-1)/3 * 100

Missing values will be imputed for missing items by "assuming that the missing items have values equal to the average of those items which are present" for any scale in which at least half the items are completed. A scale in which less than half of the items are completed will be treated as missing. This is the method proposed in the scoring manual[2]. For missing responses on any single-item

measures, the score is set to missing. A questionnaire will be considered as valid if at least one of the 15 scales/items is non-missing (after imputation).

All questionnaires completed at baseline and on-study will be assigned to a time-point according to the windowing criteria and included in the analysis. In case a participant has more than one valid on-study assessments within the same window, the valid assessment closest to the time-point will be used. In the case of two valid assessments at a similar distance to the time-point, the latest one will be chosen. In the event where the participant has no valid assessment at all in a specific window, the observation will be treated as missing for that time-point. The selection should be done at the questionnaire level and not at the question or scale level within the questionnaire.

Table 4.1.9.1-1: Time Windows for EORTC-QLQ-C30 Assessments

Nominal Time-Point	Time Window
Baseline (C1D1)[a]	On or prior to first dose on Day 1
Week 5	Nominal Day 29 (Day 2 through Day 43, inclusive)
Week 9 and every 4 weeks	Nominal Day of previous visit + 28 (- 13 days, +14 days, inclusive)
Follow-Up 1[a,b]	Nominal Day 31 Post Last Dose Date (Day 2 Post Last Dose Date through Day 83 Post Last Dose Date, inclusive)[c]<br>*D83 is derived as:*<br>*Nominal Day 31 + [(D136-D31)/2] days = Nominal Day 31 + 52 days*<br>If date of discontinuation is after Nominal Day 31 Post Last Dose Date, then Nominal Day = Date of discontinuation (Date of discontinuation through Date of discontinuation + (Nominal Day 136 Post Last Dose Date- Date of discontinuation)/2, inclusive).
Follow-Up 2[a,b]	Nominal Day 136 Post Last Dose Date (Day 84 Post Last Dose Date through Day 178 Post Last Dose Date, inclusive)[c]<br>*Day 178 is derived as:*<br>*Nominal Day 136 + 42 days (half window's wide)*<br>If date of discontinuation is after Day 31, then Nominal Day = Nominal Day 136 Post Last Dose Date (Date of discontinuation + (Nominal Day 136 Post Last Dose Date- Date of discontinuation)/2 + 1 through Day 178 Post Last Dose Date, inclusive).

[a] For participants randomized and not treated: Baseline will be defined as "On or prior to randomization date". Any assessment after the randomization date will be considered as follow-up visit. "Last dose" date reference in Follow-Up 1 and Follow-Up 2 Time Window derivation will be replaced by randomization date.

[b] These Time Window definitions are based on the definition of Follow-up visits definition in the study protocol (See Section 2, footnote of Table 2-3).

[c] Last dose date considered as Day 1 of Follow-Up. So Nominal Day 31 corresponds to 30 days post last dose date, Nominal Day 136 corresponds to 135 days post last dose date.

4.1.9.2 EQ-5D-5L

Participants' reports of general health status will be assessed using the five-level EQ-5D (EQ-5D-5L). EQ-5D-5L has 2 components: the descriptive system and the visual analogue scale (VAS).

The instrument's descriptive system comprises 5 dimensions: mobility, self-care, usual activities, pain/discomfort, and anxiety/depression. Each dimension has 5 levels, reflecting "no", "slight", "moderate", "severe", and "extreme" or "unable to". A dimension for which there are no problems is said to be at level 1, while a dimension for which there are extreme problems is said to be at level 5. Thus, the vectors 11111 and 55555 represent the best health state and the worst health state, respectively, described by the EQ-5D-5L. Altogether, the instrument describes $5^5 = 3125$ health states. Empirically derived weights can be applied to an individual's responses to the EQ-5D-5L descriptive system to generate a utility index measuring the value to society of his or her current health. As the EQ-5D-5L English value set is not yet accepted by the National Institute for Health and Care Excellence (NICE), this study will use a crosswalk method to map to the EQ-5D-3L UK value set to derive health utility scores based on the mapping function developed by the Decision Support Unit (DSU) EEPRU model developed by Hernandez-Alava et. al.[3,4], and/or using the strategy recommended by NICE at the time of the analysis.

In addition, the EQ-5D-5L includes a VAS, which allows respondents to rate their own current health on a 101-point scale ranging from 0="worst imaginable" health to 100="best imaginable" health state[5].

A questionnaire will be considered as valid if all 5 questions of EQ-5D-5L descriptive system are completed or if the EQ-5D-5L VAS is answered.

All questionnaires completed at baseline and on-study will be assigned to a time-point according to the windowing criteria in Table 4.1.9.2-1 and included in the analysis. In case a participant has more than one valid on-study assessment within the same window, the valid assessment closest to the time-point will be used. In the case of two valid assessments at a similar distance to the time-point, the latest one will be chosen. In the event where the participant has no valid assessment at all in a specific window, the observation will be treated as missing for that time-point. The selection should be done at the questionnaire level and not at the question or component level within the questionnaire.

Table 4.1.9.2-1: Time Windows for EQ-5D-5L Assessments

Nominal Time-Point	Time Window
Baseline (C1D1)[a]	On or prior to first dose on Day 1
Week 5	Nominal Day 29 (Day 2 through Day 43, inclusive)
Week 9 and every 4 weeks	Nominal Day of previous visit + 28 (- 13 days, +14 days, inclusive)
Follow-Up 1[a,b]	Nominal Day 31 Post Last Dose Date (Day 2 Post Last Dose Date through Day 83 Post Last Dose Date, inclusive)[c].<br>*D83 is derived as:*<br>*Nominal Day 31 + [(D136-D31)/2] days = Nominal Day 31 + 52 days*<br>If date of discontinuation is after Nominal Day 31 Post Last Dose Date, then Nominal Day = Date of discontinuation (Date of discontinuation through Date of discontinuation + (Nominal Day 136 Post Last Dose Date- Date of discontinuation)/2, inclusive).

Approved v2.0 930199387 2.0

Table 4.1.9.2-1: Time Windows for EQ-5D-5L Assessments

Nominal Time-Point	Time Window
Follow-Up 2[a,b]	Nominal Day 136 Post Last Dose Date (Day 84 Post Last Dose Date through Day 178 Post Last Dose Date, inclusive)[c]<br>*Day 178 is derived as:*<br>*Nominal Day 136 + 42 days (half window's wide)*<br>If date of discontinuation is after Day 31, then Nominal Day = Nominal Day 136 Post Last Dose Date (Date of discontinuation + (Nominal Day 136 Post Last Dose Date- Date of discontinuation)/2 + 1 through Day 178 Post Last Dose Date, inclusive).
Long-term Follow-Up[b]	**Survival Follow-Up 1**<br>Nominal Day 85 Post Follow-up 2 Assessment = Nominal Day 220 Post Last Dose Date (Day 179 Post Last Dose Date through Day 262 Post Last Dose Date, inclusive)[a]<br>Subsequent Survival Follow-Up visits<br>Every 12 weeks, i.e.<br>Nominal Day of previous visit + 84 (+ 42 days/- 41 days, inclusive)

[a] For participants randomized and not treated: Baseline will be defined as "On or prior to randomization date". Any assessment after the randomization date will be considered as follow-up visit. "Last dose" date reference in Follow-Up 1 and Follow-Up 2 Time Window derivation will be replaced by randomization date.

[b] These Time Window definitions are based on the definition of Follow-up and long-term Follow-up visits definition in the study protocol (See Section 2, footnote of Table 2-3).

[c] Last dose date considered as Day 1 of Follow-Up. So Nominal Day 31 corresponds to 30 days post last dose date, Nominal Day 136 corresponds to 135 days post last dose date.

4.1.9.3 FACIT-GP5

Participants will be asked to complete a single item from the Functional Assessment of Cancer Therapy (FACIT-GP5), that will be used to assess the extent of perceived bother due to symptomatic AEs. Evidence exists for the validity of this item and its usefulness as an overall summary measure of burden due to symptomatic treatment toxicities. Time windows are the same as for EORTC-QLQ-C30 assessments.

4.2 Safety Endpoints

The assessment of safety will be based on the incidence of adverse events (AEs, including drug-related AEs), serious adverse events (SAEs), adverse events leading to discontinuation, adverse events leading to dose modification, AEs leading to death, select adverse events (select AEs) for EU/ROW Submissions, immune-mediated AEs (IMAEs) for US Submission, other events of special interest (OEOSI), and deaths. The use of immune modulating medication will be also summarized. In addition, clinical laboratory tests, and immunogenicity (i.e. development of anti-drug antibody) will be analyzed.

Approved v2.0 930199387 2.0

4.3 Other Endpoints

4.3.1 Pharmacokinetics

PK will be determined from serum nivolumab and relatlimab concentrations. Samples will be collected to characterize pharmacokinetics of nivolumab and relatlimab and to explore exposure-safety and exposure-efficacy relationships. Nivolumab and relatlimab concentration-time data at scheduled trough (Ctrough) and end-of-infusion timepoints will be evaluated.

4.3.2 Biomarkers

A variety of factors that could potentially be associated with clinical efficacy and safety will be investigated in peripheral blood and in tissue/tumor specimens taken from all participants prior to treatment and as outlined in the protocol. Data from these investigations will be evaluated for associations with efficacy and/or safety data. Biomarker endpoints include, but are not limited to, PD-L1 expression, LAG-3 expression, tumor mutational burden (TMB), BRAF mutation status, genomic signatures, soluble biomarkers and levels of circulating tumor DNA (ctDNA).

4.3.3 Immunogenicity

Serum samples for analysis of development of ADAs will be collected in conjunction with analysis of nivolumab and relatlimab serum concentrations and will be collected from all participants. These serum samples will be analyzed for nivolumab and relatlimab ADAs by validated immunoassay; samples may also be analyzed for neutralizing antibodies by validated methods. Samples collected from a participant in the nivolumab monotherapy group will only be analyzed for nivolumab ADAs. Neutralizing antibodies for nivolumab monotherapy group will be estimated too, if the sample is ADA positive.

4.4 Estimands

Main Estimands for primary and secondary endpoints are defined in Table 4.4-1:

Table 4.4-1: List of Main Estimands

Estimand Attribute	Definition		
**Endpoint**	Primary Endpoint: To compare the efficacy, as measured by RFS (defined as Recurrence (local, regional[a], distant), new primary invasive melanoma[a], or death whichever comes first), provided by nivo + rela FDC vs nivolumab monotherapy in all randomized participants.		
**Treatment**	Nivo + rela FDC compared to nivolumab monotherapy		
**Population**	All randomized participants		
**Variable**	RFS per investigator		
**Intercurrent Events (ICEs)**	**Event**	**Strategy**	**Description**
	Randomized by not treated	Treatment Policy	Recurrence or death that occurs after ICE will be counted as event.
	Discontinued treatment	Treatment Policy	Recurrence or death that occurs after ICE will be counted as event.
	Start of new systemic anticancer therapy, tumor-directed radiotherapy, or tumor-directed surgery	Treatment Policy	Recurrence or death that occurs after ICE will be counted as event.
	Melanoma in situ, second non-melanoma primary cancer, or non-invasive BCC/SCC[b]	Treatment Policy	Recurrence or death that occurs after ICE will be counted as event.
**Population-level Summary**	HR with confidence interval from stratified Cox proportional hazard model, KM estimates, stratified log-rank test for group comparison.		
**Endpoint**	Secondary endpoint in hierarchy: Overall Survival: To compare the efficacy, as measured by OS, provided by nivo + rela FDC vs nivolumab monotherapy in all randomized participants.		
**Treatment**	Nivo + rela FDC compared to nivolumab monotherapy		
**Population**	All randomized participants		
**Variable**	Overall survival		
**Intercurrent Events (ICEs)**	**Event**	**Strategy**	**Description**
	Randomized by not treated	Treatment Policy	Death after ICE will be counted as an event.

Approved v2.0 930199387 2.0

Table 4.4-1: List of Main Estimands

Estimand Attribute	Definition		
	Discontinued treatment	Treatment Policy	Death after ICE will be counted as an event.
	Start of new systemic anti-cancer therapy, tumor-directed radiotherapy, or tumor-directed surgery	Treatment policy	Death after ICE will be counted as an event.
	Melanoma in situ, second non-melanoma primary cancer, or non-invasive BCC/SCC[b]	Treatment Policy	Death after ICE will be counted as an event.
**Population-level Summary**	HR with confidence interval from stratified Cox proportional hazard model, KM estimates, stratified log-rank test for group comparison.		
**Endpoint**	Other Secondary Endpoint:<br>To assess the efficacy as measured by distant metastasis-free survival (DMFS), provided by nivo + rela FDC vs nivolumab monotherapy in all randomized Stage III for non-mucosal participants, Stage III/IVA/IVB for mucosal participants		
**Treatment**	Nivo + rela FDC compared to nivolumab monotherapy		
**Population**	All randomized stage III for non-mucosal participants, Stage III/IVA/IVB for mucosal participants		
**Variable**	DMFS per investigator		
**Intercurrent Events (ICEs)**	**Event**	**Strategy**	**Description**
	Randomized by not treated	Treatment Policy	Distant recurrence or death that occurs after ICE will be counted as event.
	Discontinued treatment	Treatment Policy	Distant recurrence or death that occurs after ICE will be counted as event.
	Local or regional recurrence	Treatment Policy	Distant recurrence or death after ICE will be counted as an event.
	Start of new systemic anticancer therapy, tumor-directed radiotherapy, or tumor-directed surgery	Treatment Policy	Distant recurrence or death after ICE will be counted as an event.
	Melanoma in situ, second non-melanoma primary cancer, or non-invasive BCC/SCC[b]	Treatment Policy	Distant recurrence or death after ICE will be counted as an event.
**Population-level Summary**	HR with confidence interval from stratified Cox proportional hazard model, KM estimates. No statistical testing.		

Approved v2.0 930199387 2.0

Table 4.4-1: List of Main Estimands

Estimand Attribute	Definition		
**Endpoint**	Other Secondary Endpoint:<br>To assess the efficacy as measured by PFS on next-line therapy (PFS2), provided by nivo + rela FDC vs nivolumab monotherapy in all randomized participants		
**Treatment**	Nivo + rela FDC compared to nivolumab monotherapy		
**Population**	All randomized participants		
**Variable**	PFS2 per investigator		
**Intercurrent Events (ICEs)**	**Event**	**Strategy**	**Description**
	Starting SST before 1st recurrence/progression	Treatment Policy	Recurrence or death after ICE will be counted as an event.
	Non-systemic therapy (e.g., surgery/radiotherapy)	Treatment Policy	Recurrence or death after ICE will be counted as an event.
	Any recurrence/progression after non-systemic therapy and before the 1st SST	Treatment Policy	Recurrence or death after ICE will be counted as an event. Recurrence or PD occurring in this scenario would likely be treated with local therapies only.
	Second non-melanoma primary cancer	Hypothetical	Recurrence or death that occurs after ICE will **not** be counted as event. Participant will be censored on the date of diagnosis of second non-melanoma cancer.
	Melanoma in situ, non-invasive BCC/SCC[b]	Treatment Policy	Recurrence or death after ICE will be counted as an event.
	Starting 2nd (or later) SST regimen where Previous SST was stopped due to PD (with missing PD date)	Composite	Recurrence or death that occurs after ICE will **not** be counted as event. Participant will be considered having an event at the end date of previous SST[c].
	Starting 2nd (or later) SST regimen where previous SST was stopped due to non-PD reason (including missing reason)	Treatment Policy	Recurrence or death after ICE will be counted as an event.
**Population-level Summary**	HR with confidence interval from stratified Cox proportional hazard model, KM estimates. No statistical testing.		

Approved v2.0 930199387 2.0

[a] Regional recurrence includes in-transit or regional nodal recurrence. New primary invasive melanoma does not include in situ melanoma as an event.

[b] Non-invasive BCC and SCC are not considered new second non-melanoma primary cancers. They are skin cancers with low chance of invasion and metastasizing and are generally curable.

[c] If the end date of previous SST is missing, then the participant will be considered having an event at the start date of the following regimen.

Approved v2.0 930199387 2.0

Supplemental Estimands for primary and secondary endpoints are defined in Table 4.4-2:

Table 4.4-2: List of Supplemental Estimands

Estimand Attribute	Definition		
**Endpoint**	Primary Endpoint: To compare the efficacy, as measured by RFS (defined as Recurrence (local, regional[a], distant), new primary invasive melanoma[a], or death whichever comes first), provided by nivo + rela FDC vs nivolumab monotherapy in all randomized participants.		
**Treatment**	Nivo + rela FDC compared to nivolumab monotherapy		
**Population**	All randomized participants		
**Variable**	RFS per investigator		
**Intercurrent Events (ICEs)**	**Event**	**Strategy**	**Description**
	Randomized by not treated	Treatment Policy	Recurrence or death that occurs after ICE will be counted as event.
	Discontinued treatment	Treatment Policy	Recurrence or death that occurs after ICE will be counted as event.
	Start of new systemic anticancer therapy, tumor-directed radiotherapy, or tumor-directed surgery	Hypothetical	Recurrence or death that occurs after ICE will **not** be counted as event. Participant will be censored on the date of last evaluable disease assessment prior to or on the same date of initiation of new systemic anticancer therapy, tumor-directed radiotherapy, or tumor-directed surgery
	Second non-melanoma primary cancer[b]	Hypothetical	Recurrence or death that occurs after ICE will **not** be counted as event. Participant will be censored on the date of last evaluable disease assessment prior to or on the same date of diagnosis of second non-melanoma cancer
	Melanoma in situ, non-invasive BCC/SCC[b]	Treatment Policy	Recurrence or death after ICE will be counted as an event.
**Population-level Summary**	HR with confidence interval from stratified Cox proportional hazard model, KM estimates, stratified log-rank test for group comparison.		

[a] Regional recurrence includes in-transit or regional nodal recurrence. New primary invasive melanoma does not include in situ melanoma as an event.

[b] Non-invasive BCC and SCC are not considered new second non-melanoma primary cancers. They are skin cancers with low chance of invasion and metastasizing and are generally curable.

4.4.1 Supplemental Estimand of Recurrence-Free Survival (Censoring for Subsequent Therapy)

For the primary endpoint of RFS, as described in Table 4.4-2, a supplemental analysis will be performed using a hypothetical estimand strategy to handle the intercurrent events of start of new anticancer systemic therapy, tumor-directed radiotherapy, tumor-directed surgery or second non-melanoma primary cancer. Under this strategy, RFS will be censored:

- on the date of last evaluable disease assessment prior to or on the same date of initiation of new systemic anticancer therapy, tumor-directed radiotherapy, or tumor-directed surgery
- on the date of last evaluable disease assessment prior to or on the same date of diagnosis of second non-melanoma cancer

whichever occurs first.

Censoring rules for supplemental estimand of RFS are presented in Table 4.4-3 and Figure 4.4-1.

Table 4.4-3: Censoring Scheme for Supplemental Estimand Analysis of Recurrence-Free Survival

Situation	Date of Event or Censoring	Outcome
No baseline disease assessment	Date of randomization	Censored
Disease at baseline	Date of randomization	Censored
No on-study disease assessments and no recurrence or death	Date of randomization	Censored
No recurrence and no death*, no new anti-cancer therapy started and no new primary non-melanoma skin cancer	Date of last evaluable disease assessment	Censored
New systemic anti-cancer therapy, tumor-directed radiotherapy, or tumor-directed surgery started without death or recurrence reported prior to disease assessment	Date of last disease assessment prior to or on the date of initiation of the subsequent anti-cancer therapy, tumor-directed radiotherapy, or tumor-directed surgery	Censored
Second non-melanoma primary cancer reported prior or on the same day of disease assessment	Date of last evaluable disease assessment prior to or on the same date of diagnosis of second non-melanoma primary cancer	Censored
Recurrence (local, regional, distant, new primary invasive melanoma)	Date of first recurrence	Event
Death without recurrence	Date of death	Event

Regional recurrence includes in-transit or regional nodal recurrence. New primary invasive melanoma does not include in situ melanoma as an event.

Approved v2.0 930199387 2.0

Non-invasive BCC and SCC are not considered new non-melanoma cancers. They are skin cancers with low chance of invasion and metastasizing and are generally curable.

* Disease assessments, recurrence, and death if any, occurring either after start of subsequent anti-cancer therapy or after diagnosis of second non-melanoma primary cancer are not considered.

Abbreviations: BCC, basal cell carcinoma; SCC, squamous cell carcinoma

Approved v2.0 930199387 2.0

Figure 4.4-1: RFS Definition - Supplemental Estimand

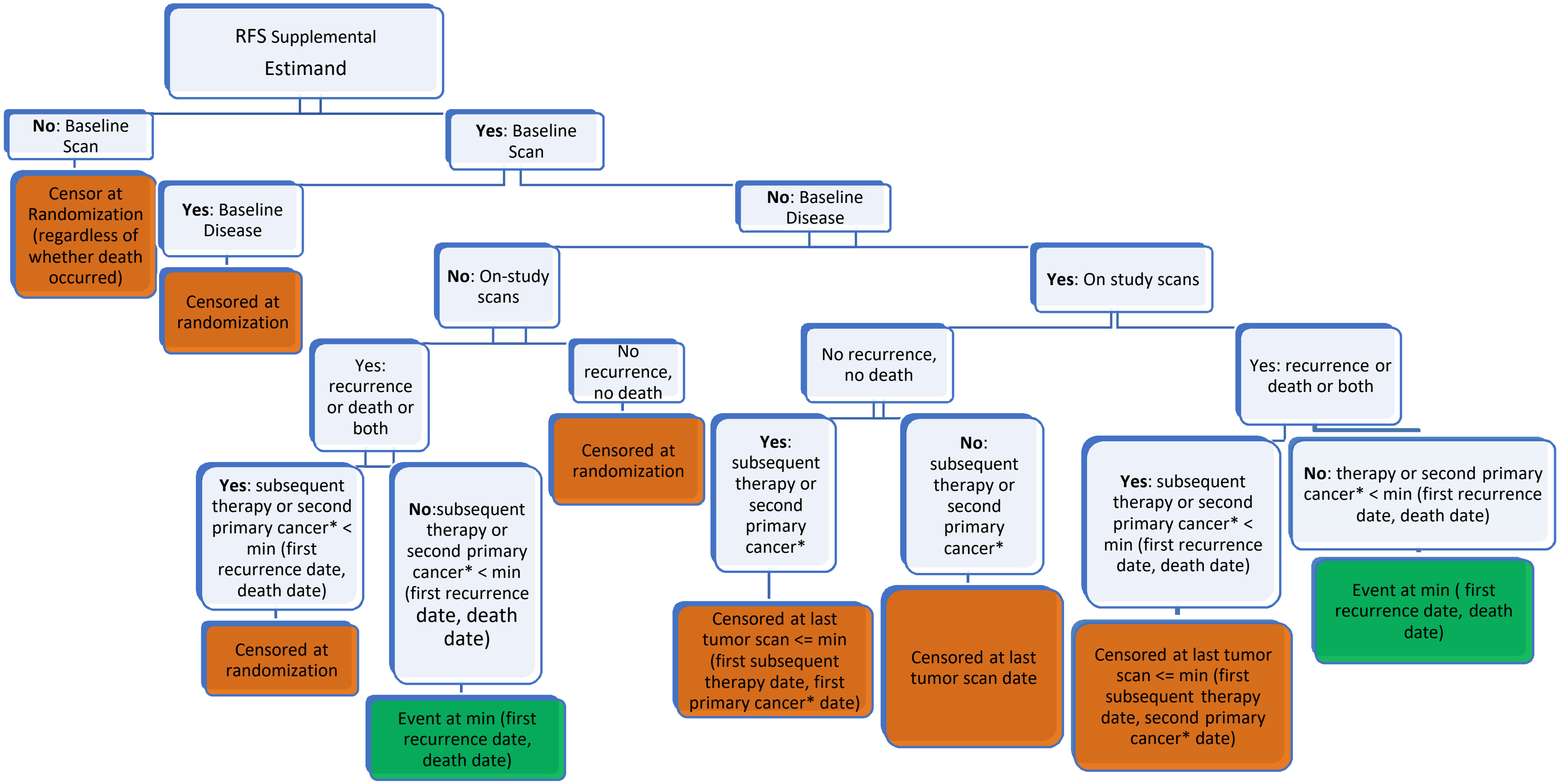

* non-melanoma primary cancer

Approved v2.0 930199387 2.0

5 SAMPLE SIZE AND POWER

5.1 Recurrence Free-Survival

The primary objective of the study is to compare RFS of nivo + rela FDC to nivolumab monotherapy in participants with completely resected Stage IIIA (> 1 mm tumor in lymph node), Stage IIIB/C/D, or Stage IV NED melanoma. The number of events and power were calculated assuming a non-proportional hazards model with a 6-month delayed treatment effect, a cure rate in the 2 treatment arms and a plateauing of treatment effect from 7.5 years. The sample size calculations were simulated using EAST and Statistical Analysis System (SAS®) software (SAS Institute, North Carolina, USA).

Approximately 410 RFS events would be required in the 2 respective treatment arms for a 2-sided alpha = 0.05 stratified log rank test to show a statistically significant difference in RFS between the treatment arms with approximately 90% overall power when the average hazard ratio (HR) of nivo + rela FDC arm to nivolumab monotherapy arm is 0.72, and a cure rate of 0.52 is assumed in the nivolumab monotherapy arm (0.63 cure rate in the nivo + rela FDC arm). This assumes that the RFS rate at 36 months is 58.6% and 68.5% in the nivolumab monotherapy and nivo + rela FDC arms, respectively. Initially, the calculations were based on the following projected accrual rates; 1-3 months: 12 participants/month, 4-5 months: 30 participants/month, ≥ 6 months: 44 participants/month; it was expected to take about 27 months to accrue the approximately 1,050 participants in the study. The accrual rate assumed a 5% overall drop out (lost to follow-up) rate in each treatment arm by 52 months. However, the accrual has been completed in approximately 14 months which influences the timing of the expected events and analyses.

Under these assumptions of accrual, the observed RFS distribution of the nivolumab monotherapy arm in the CA209238 study, and the assumed HR stated above, it would take approximately 52 months from the randomization of the first participant to observe the required number of 410 RFS events. It is projected that an observed HR of 0.815 or less would result in a statistically significant improvement at the final analysis of RFS.

Two interim analyses for efficacy (RFS) will be performed based on number of events detailed below.

The first interim analysis for RFS (RFS IA#1) will be performed when approximately 309 RFS events have occurred (~75% information fraction). At that time, a minimum follow-up of 14 months is expected for all randomized participants (i.e., approximately 28 months from first participant randomized in the study). The significance boundaries at the interim analyses will be based on an O'Brien and Fleming alpha spending function. With an interim RFS analysis at 309 RFS events, the nominal significance level would be 0.020 (2-sided) and an observed HR of 0.765 or less would result in a statistically significant improvement, with power of 47%.

If RFS IA#1 is not statistically significant, the second interim analysis for RFS (RFS IA#2) will be performed when approximately 361 RFS events have occurred (~88% information fraction). At that time, a minimum follow-up of 24 months is expected for all randomized participants (i.e., approximately 38 months from first participant randomized in the study). With an interim RFS

Approved v2.0 930199387 2.0

analysis at 361 RFS events, the nominal significance level would be 0.028 (2-sided) and an observed HR of 0.793 or less would result in a statistically significant improvement at the interim analysis with cumulative power of 75%. A decision may be taken by BMS not to perform RFS IA#2 if, at the time we reach the expected numbers of events for RFS IA#2, the predicted time frame to reach RFS FA number of events (410 events) is less than 6 months. Alpha spending will be adjusted appropriately to not include RFS IA#2 if this is not performed.

If the final RFS analysis occurs at 410 RFS events, the critical hazard ratio would be 0.815 (average HR of 0.72) and a nominal significance level of 0.04 (2-sided). The nominal significance level used for the analyses will be calculated based on the actual number of RFS events at interim and final analyses, respectively.

5.2 Overall Survival

The key secondary endpoint of OS will be tested if the primary objective of RFS is statistically significant.

For the comparison of OS between nivo + rela FDC and nivolumab monotherapy in all randomized participants, approximately 309 death events would be required in the 2 respective treatment arms for a 2-sided alpha = 0.05 stratified log-rank test to show a statistically significant difference in OS between the treatment arms with approximately 70% overall power. This assumes a target HR of nivo + rela FDC vs nivolumab monotherapy of 0.75 and the OS in the nivolumab monotherapy arm is exponentially distributed with a survival rate at Year 4 of 78%. It is projected that an observed HR of 0.794 or less would result in a statistically significant improvement at the final analysis of OS. The nominal significance level used for the analyses will be calculated based on the actual number of OS events at interim and final analyses, using the O’Brien and Fleming alpha spending function.

Assuming the RFS result is significantly superior, there will be an interim analysis of OS performed by the DMC at the time of RFS significance (at RFS IA#1, RFS IA#2, or RFS FA). If RFS is statistically significant at one of the interims (RFS IA#1, RFS IA#2), but OS is not statistically significant, an additional interim analysis of OS will be performed by the DMC at approximately 52 months from the randomization of the first participant (~59% information fraction, 183 OS events out of 309). If this OS IA is not statistically significant, another OS IA will be performed at approximately 71 months from the randomization of the first participant (~81% information fraction, 250 OS events out of 309). Final OS analysis will be performed when approximately 309 OS events have occurred or at 90 months from the randomization of the first participant, whichever comes first. Details for the OS interim analyses are provided in Table 5.2-1. The significance boundaries at the interim analysis will be based on the actual number of events and an O’Brien and Fleming alpha spending function.

Approved v2.0 930199387 2.0

Table 5.2-1: Formal Analysis of Overall Survival - Operating Characteristics at Each Time Point

Analysis	Criterion for Conducting Analysis	Estimated Time[a]	Estimated Number of OS Events	Information Fraction	Nominal Significance Level[b] 2-sided	Cumulative Power[c]
**OS IA#1[d]**	At the time of RFS IA#1, if significant	28	87	28%	0.0004	0.3%
**OS IA#1[d]**	At the time of RFS IA#2, if significant	38	128	41%	0.001	5%
**OS IA[d]**	At the time of RFS FA or 52 months	52	183	59%	0.007	23%
**OS IA[d]**	71 months after first randomized	71	250	81%	0.023	51%
**OS FA**	90 months after first randomized	90	309	100%	0.042	70%

Abbreviations: Approx, approximate; DMC, Data Monitoring Committee; FA, final analysis; FPFV, first patient first visit; IA, interim analysis; OS, overall survival; RFS, recurrence-free survival.

[a] From first participant randomized.

[b] Assuming that the observed number of events is exactly as in the table. The actual significance boundaries at each analysis will be calculated based on the actual number of OS events, using an O'Brien and Fleming alpha spending function.

[c] The probability of crossing the boundary under the alternative hypothesis, cumulative across time points.

[d] In the event that RFS is statistically significant at one of the RFS interims analyses (but OS interim is not statistically significant), additional interim OS analyses will be performed by the DMC at 52 months and 71 months.

5.3 Testing Strategy

The statistical comparisons for the primary efficacy endpoint (RFS) and the key secondary endpoint (OS) will be carried out in the hierarchical order: if the primary efficacy endpoint (RFS) is met, the key secondary endpoint (OS) will be tested.

This means that a statistically significant result for the primary comparison (RFS) is required to initiate the testing of the key secondary endpoint (OS). Since a step-down procedure is used, each comparison will be tested at a significance level of 0.05 (2-sided) and an overall two-sided alpha level of 0.05 will be preserved. If the DMC requests efficacy summary data in order to perform risk-benefit assessment prior to the RFS IAs or at any time outside pre-specified analysis, an administrative alpha penalty of 10^{-4} will occur and will be subtracted from the overall type I error of 0.05 for each additional unplanned look requested by the DMC.

At the interim analysis for RFS and OS, the boundaries for declaring superiority will be derived based on the actual number of events using Lan-DeMets spending function with O'Brien and Fleming type of boundary based on the corresponding information fraction observed. For both RFS and OS, the boundaries for concluding superiority at interim analysis will be provided to the DMC by the independent statistician. The RFS and OS distributions will be compared between

Approved v2.0 930199387 2.0

treatment groups using a two-sided stratified log-rank test in all randomized participants. Other endpoints than RFS and OS will not be tested formally.

6 STUDY PERIODS, TREATMENT REGIMENS, AND POPULATIONS FOR ANALYSES

6.1 Study Periods

6.1.1 Baseline Period

- Baseline evaluations or pre-treatment events will be defined as evaluations or events that occur before the date and time of the first dose of study treatment. Evaluations (laboratory tests, pulse oximetry, vital signs, biomarkers, and eCOAs) on the same date and time of the first dose of study treatment will be considered as baseline evaluations. Events (AEs) on the same date and time of the first dose of study treatment will not be considered as pre-treatment events. For participants who are randomized and not treated, baseline evaluation or events will be defined as those that occur before the date and time of randomization.

  For efficacy, the baseline disease assessment will be on or prior to the date of randomization.
- In cases where the time (onset time of event or evaluation time and dosing time) is missing or not collected, the following definitions will apply:
  - Pre-treatment AEs will be defined as AEs with an onset date prior to but not including the day of the first dose of study treatment.
  - Baseline evaluations (laboratory tests, pulse oximetry, vital signs, biomarkers, and eCOAs) will be defined as evaluations with a date on or prior to the date of first dose of study treatment.
  - For eCOAs, participants randomized and not treated will have baseline assessments defined as on or prior to the randomization date.
- If there are multiple valid observations in the baseline period, then the latest non missing observation will be used as the baseline in the analyses. If multiple observations exist on the latest collection date (and time if collected), the record with the latest data entry date and time will be used. If multiple observations exist on the latest collection date (and time if collected) and data entry date and time, then the first observation is used as baseline, unless otherwise specified.

6.1.2 Post Baseline Period

- Post-baseline efficacy assessments are defined as disease assessment after the date of randomization.
- On-treatment AEs will be defined as AEs with an onset date and time on or after the date and time of the first dose of study treatment (or with an onset date on or after the day of first dose of study treatment if time is not collected or is missing). For participants who are off study treatment, AEs will be included if event occurred within a safety window of 135 days after the last dose of study treatment.
- On-treatment evaluations (laboratory tests) will be defined as evaluations taken after the day (and time, if collected and not missing) of first dose of study treatment. For participants

who are off study treatment, evaluations should be within a safety window of 135 days after the last dose of study treatment.

– Post-baseline eCOA evaluations will be defined as evaluations with a date after the date of first dose of study treatment. For participants who were randomized and not treated, any assessment after the randomization date will be considered a follow-up visit.

6.2 Treatment Regimens

Treatment group "as randomized" corresponds to the treatment group assigned by the Interactive Response Technology (IRT) system.

- Arm A: Experimental arm: BMS-986213 (nivo + rela FDC)
- Arm B: Control arm: nivolumab monotherapy

The treatment group "as treated" corresponds to the treatment group the participant actually received and is generally expected to be the same as the treatment group "as randomized" by IRT.

Unless otherwise specified, the safety analysis will be based on the treatment group "as treated".

Unless otherwise specified, the efficacy analysis and COA analysis will be based on the treatment group "as randomized".

6.3 Populations for Analyses

- Global study population: all subjects enrolled during the global accrual window (from first patient first consent date to last patient outside of China's sub-study consent date [i.e., 07Nov2022]). Any patients from China enrolled during global accrual window will be included.
- Enrolled population: All participants from the global study population who signed the informed consent form and obtained a participant number from the IRT.
- Randomized population: All participants from the global study population who were randomized through the IRT. Analyses will use the treatment arm as randomized, unless otherwise specified.
- Treated population: All participants from the global study population who received at least one dose of any study medication. Analysis will use the treatment arm as treated, unless otherwise specified.
- PK population: All participants from the global study population who received at least one dose of any study medication and with at least one pre-dose or post-dose available serum concentration-time data.
- Immunogenicity population: All participants from the global study population with available ADA data.
- Biomarker population: All randomized participants with at least one evaluable pre-dose biomarker assessment (PD-L1 and LAG-3 will be analyzed separately).

Approved v2.0 930199387 2.0

7 STATISTICAL ANALYSES

7.1 General Methods

Unless otherwise noted, discrete variables will be tabulated by the frequency and proportion of participants falling into each category, grouped by treatment. Percentages given in these tables will be rounded to the first decimal and, therefore, may not always sum to 100%. Percentages less than 0.1 will be indicated as '< 0.1'. Continuous variables will be summarized by treatment group using the mean, standard deviation, median, minimum, and maximum values.

Time-to-event variables (e.g., time-to resolution) will be analyzed using the Kaplan-Meier technique. When specified, the median will be reported along with 95% CI using Brookmeyer and Crowley method[6] (using log-log transformation for constructing the confidence intervals[7]).

The conventions to be used for imputing missing and partial dates for analyses requiring dates are described in Section 7.11.

Laboratory results, adverse events, and other symptoms will be graded using the National Cancer Institute (NCI) Common Terminology Criteria for Adverse Events (CTCAE), Version 5.0, except where CTCAE grades are not available. Adverse events will be categorized using the most current version of Medical Dictionary for Regulatory Activities (MedDRA), by system organ class and preferred term. Prior therapies will be summarized using the most current version of the World Health Organization (WHO) drug dictionary.

In the open report, the summary tables and listings will be presented as pooled across the two treatment arms, without revealing the treatment identity. In the closed report, partially unblinded summaries and listings with treatment arm labeled as "Arm A" and "Arm B" will be presented. The contents of these reports are outlined in Section 5.3 of the DMC charter.

Statistical analyses will be carried out in SAS (Statistical Analysis System, SAS Institute, North Carolina, USA), unless otherwise indicated.

7.2 Study Conduct

7.2.1 General Study Information

The following summary table will be presented on the All Randomized population: the potential duration of time on study, defined as the time between randomization date and cutoff date (defined by last patient last visit date), will be summarized descriptively (median, min, max, etc.) in months for all randomized participants.

A by-participant listing of batch numbers for all treated participants will also be provided.

7.2.2 Accrual

The following summary tables will be presented on the All Enrolled population:

- Number (%) of participants enrolled, randomized, and treated by country, and investigational site.
- Number (%) enrolled and randomized by month.

Approved v2.0 930199387 2.0

A by-participant listing of participants accrued by country, and investigational site will also be provided.

7.2.3 Protocol Deviations

A listing of Important Protocol Deviations will be provided.

7.2.4 Relevant Protocol Deviations

The Relevant Protocol Deviations will be summarized and listed on all randomized participants: by treatment group as randomized and overall.

At Entrance:

- No histologically documented melanoma stage IIIA/B/C/D or IV for non-mucosal patients or stage III or IVA/B/C for mucosal patients.
- Documented/confirmed disease at baseline.
- Participants with baseline ECOG performance status > 1.
- The last intervention demonstrating that the participant is free of disease is more than 90 days prior to randomization (+7 day window).
- Interferon use within 6 weeks of randomization or any prior systemic anti-cancer therapy for melanoma.
- Participants with ocular melanoma except conjunctival melanoma.

On-study:

- Participants receiving systemic anti-cancer therapy (chemotherapy, immunotherapy, standard or investigational agents for treatment of cancer) while on study therapy.
- Participants treated differently than as randomized (participants who received the wrong treatment, excluding the never treated).

7.3 Study Population

Analyses in this section will be tabulated for all randomized participants by treatment group as randomized, unless otherwise specified.

7.3.1 Participant Disposition

The total number of participants enrolled (randomized or not randomized) will be presented along with the reason for not being randomized. This analysis will be performed on the All Enrolled population only.

Number of participants randomized but not treated along with the reason for not being treated will be tabulated by treatment group as randomized and overall.

Number of participants who discontinued study treatment along with corresponding reason will be tabulated by treatment group as treated. Reason for discontinuation will be derived from participant status CRF page. This analysis will be performed only on the All Treated participants population.

Approved v2.0 930199387 2.0

A summary table will be provided for number of participants unblinded and also number of participants unblinded prior to recurrence by treatment arms.

A by-participant listing for all treated participants will be provided showing the participant's off treatment date and whether the participant continues in the treatment period/study along with the reason for going off treatment period/study. A by-participant listing for all enrolled participants will also be provided, showing whether the participant was randomized/treated along with the reason for not being randomized/treated.

A listing of randomization scheme presenting randomized treatment group and as treated treatment group will be provided for all randomized participants.

7.3.2 Demographics and Other Baseline Disease Characteristics

The following demographic and baseline disease characteristics will be summarized and listed by treatment group as randomized and overall:

- Age (continuous)
- Age categorization (≥ 12 and <18, ≥ 18 and < 65, ≥ 65 and < 75, ≥ 75 and < 85, ≥ 65, ≥ 75, ≥ 85)
- Sex (Male vs. Female)
- Race (as categorized: White/Black or African American/Asian (includes Indian, Chinese, Japanese, Korean, "Asian Indian", Malay, American Indian or Alaska Native)/Other (other, Native Hawaiian or Other Pacific Islander source: eCRF))
- Ethnicity (Hispanic/Latino vs. Not Hispanic/Latino : source: eCRF)
- AJCC v8 stage at study entry (Source: eCRF) using the following categories: Stages IIIA/IIIB, Stage IIIC, Stages IIID/IV (including all participants with mucosal melanoma, Stage III, Stage IVA, Stage IVB, and Stage IVC)
- AJCC v8 stage at study entry (Source: eCRF):
  - Non-mucosal participants: Stage IIIA, Stage IIIB, Stage IIIC, Stages IIID, Stage IV
  - Mucosal participants: Stage III, Stage IVA, Stage IVB, and Stage IVC
- Melanoma Subtype at study entry (cutaneous non-acral; cutaneous acral; mucosal; unknown primary; other)
- Baseline Metastasis Stage (Source: eCRF)
  - Non-mucosal participants : M0, M1a, M1b, M1c, M1d
  - Mucosal participants : M0, M1
- Baseline PD-L1 Status (≥ 1%; <1%; indeterminate/unevaluable; not reported) (Source: clinical database)
- Baseline LAG-3 Status (≥ 1%; <1%; indeterminate/unevaluable; not reported) (Source: clinical database)
- Baseline BRAF status (mutant; wild type; Not Reported)
- ECOG (0; 1, > 1, Not Reported)

Approved v2.0 930199387 2.0

- Geographic Region, as per stratification groups and key individual countries/region as subcategories underneath (Source: eCRF):
  - USA/Canada/Australia,
    - ♦ USA/Canada
    - ♦ Australia
  - Europe,
  - Rest of the World
    - ♦ Latin America
    - ♦ China
- Baseline LDH level (≤ ULN, > ULN)

Note: If for a Patient, derived age comes up as <18, additional check will be added to see if they have an assent date (that's signed by the minor) and if so, then the age can be <18. The parent signs the consent form in this case, the minor signs the assent form. If there is no assent date then age should be re-derived to 18.

7.3.3 Medical History

A by-participant listing of general medical history for all randomized participants will be provided.

7.3.4 Prior Melanoma Directed Therapy Agents

The following will be summarized by treatment as randomized and overall:

- Prior surgery (Yes, No)
- Prior radiotherapy (Yes, No)
- Prior systemic cancer therapy (Yes, No)

Prior systemic cancer therapy, prior surgery, and prior radiotherapy related to cancer will be listed by participant.

7.3.5 Physical Examinations

Participants with abnormal baseline physical examination will be listed by participant.

7.3.6 Discrepancies Between IRT and CRF Information

Summary tables (cross-tabulations) of stratification factors for all randomized participants by treatment group will be provided to show any discrepancies between what was reported through IRT vs. CRF data or clinical database (baseline).

- AJCC v8 Stage (IRT vs. CRF data)
- Geographic Region (IRT vs. CRF Data)

Approved v2.0 930199387 2.0

7.4 Extent of Exposure

Listings will include all available exposure data. Analyses will be performed by treatment group "as treated" in all treated participants, unless otherwise specified.

7.4.1 Administration of Study Therapy

The following parameters defined in Table 7.4.1-1 and Table 7.4.1-2 will be summarized (descriptive statistics) by treatment group (nivo + rela FDC vs nivo):

- Number of doses received
- Cumulative dose (mg)
- Relative dose intensity (%) which is the actual received drug relative to the planned received drug, using the following categories:
  - < 50%; 50 - < 70%; 70 - < 90%; 90 - < 110%; ≥ 110%

Duration of study therapy will be summarized (descriptive statistics) by treatment group.In addition, time to treatment discontinuation will be summarized and presented by treatment group using a Kaplan-Meier curve whereby the last dose date will be the event date for those participants who are off study therapy. Median duration of study therapy and associated 95% CI will be estimated by the Kaplan-Meier methodology. Participants who are still on study therapy will be censored on their last dose date.

For nivo + rela FDC, time to treatment discontinuation is defined as time from the first dosing date of any drug component until the last dosing date of any drug component.

A by-participant listing of dosing of study medication (record of study medication, infusion details, and dose changes) will be also provided.

Table 7.4.1-1: Administration of Study Therapy: Nivo + Rela FDC Arm

	**Adults, and Adolescents ≥ 40 kg**	**Adolescents < 40 kg**
**Dosing schedule per protocol**	**Nivolumab 480 mg IV, Relatlimab 160 mg IV, Q4W**	**Nivolumab 6 mg/kg IV, Relatlimab 2 mg/kg IV, Q4W**
Dose	*Dose (mg) is defined as: the nominal dose (mg) * total volume infused (mL) /total volume prepared (mL)*<br>*nominal dose = 640 mg*	*Dose (mg/kg) is defined as: the minimum of [vial strength (mg/mL)*total volume infused (mL)] /most recent weight (kg) and nominal dose (mg/kg) * total volume infused (mL) /total volume prepared (mL)*<br>*Vial strength = 16 mg/mL*<br>*Dose (mg) is defined as dose (mg/kg) * most recent weight (kg)*
Cumulative Dose	*Cum dose (mg) is sum of the doses (mg) administered to a participant during the treatment period.*	*Cum dose (mg) is sum of the doses (mg) administered to a participant during the treatment period.*
Relative Dose Intensity (%)	*Cum dose (mg) / [(Last dose date - Start dose date + 28) x 640 / 28] x 100*	*Cum dose (mg/kg) / [(Last dose date - Start dose date + 28) x 8 / 28] x 100*

Table 7.4.1-1: Administration of Study Therapy: Nivo + Rela FDC Arm

	Adults, and Adolescents ≥ 40 kg	Adolescents < 40 kg
Duration of Study Therapy	*Last dose date - Start dose date +1*	*Last dose date - Start dose date +1*

Table 7.4.1-2: Administration of Study Therapy: Nivolumab Arm

	Adults, and Adolescents ≥ 40 kg	Adolescents < 40 kg
**Dosing schedule per protocol**	**Nivolumab 480 mg IV, Q4W**	**Nivolumab 6 mg/kg IV, Q4W**
Dose	*Dose (mg) is defined as: the nominal dose (mg) * total volume infused (mL) /total volume prepared (mL)*<br>*nominal dose = 480 mg*	*Dose (mg/kg) is defined as: the minimum of [vial strength (mg/mL)*total volume infused (mL)] /most recent weight (kg) and nominal dose (mg/kg) * total volume infused (mL) /total volume prepared (mL)*<br>*Vial strength = 10 mg/mL*<br>*Dose (mg) is defined as dose (mg/kg) * most recent weight (kg)*
Cumulative Dose	*Cum dose (mg) is sum of the doses (mg) administered to a participant during the treatment period.*	*Cum dose (mg) is sum of the doses (mg) administered to a participant during the treatment period.*
Relative Dose Intensity (%)	*Cum dose (mg) / [(Last dose date - Start dose date + 28) x 480 / 28] x 100*	*Cum dose (mg/kg) / [(Last dose date - Start dose date + 28) x 6 / 28] x 100*
Duration of Study Therapy	*Last dose date - Start dose date +1*	*Last dose date - Start dose date +1*

7.4.2 Modifications of Study Therapy

7.4.2.1 Dose Delays

Each nivolumab or nivo + rela FDC infusion may be delayed. A dose will be considered as actually delayed if the delay is exceeding 3 days (i.e., greater than or equal to 4 days from scheduled dosing date) for nivolumab or nivo + rela FDC. All study drugs must be delayed until treatment can resume. Reason for dose delay will be retrieved from CRF dosing pages.

The following parameters will be summarized by treatment group:

- Number of participants with at least one dose delayed, the number of dose delays per participant, the reason for dose delay and the length of dose delay.

7.4.2.2 Infusion Interruptions and Rate Changes

Each nivolumab or nivo + rela FDC infusion can be interrupted and/or the IV infusion rate can be reduced. This information will be retrieved from CRF dosing pages.

Approved v2.0 930199387 2.0

The following parameters will be summarized by treatment group:

- Number of participants with at least one dose infusion interruption, the reason for interruption, and the number of infusion interruptions per participant.
- Number of participants with at least one IV infusion rate reduction, the reason for reduction and the number of infusions with IV rate reduction per participant.

7.4.2.3 Dose Escalations

Dose escalations (within participant) are not permitted for either nivolumab or nivo + rela FDC.

7.4.2.4 Dose Reductions

Dose reductions (within participant) are not permitted for either nivolumab or nivo + rela FDC.

7.4.2.5 Dose Omissions

Dose omissions are not permitted for either nivolumab or nivo + rela FDC.

7.4.3 Concomitant Medications

Concomitant medications, defined as medications other than study medications which are taken at any time on-treatment (i.e., on or after the first day of study therapy and within 135 days following the last dose of study therapy), will be coded using the UMC WHO Drug Global Dictionary and the most recent version of the dictionary at the time of the database lock will be used.

The following summary table will be provided:

- Concomitant medications (participants with any concomitant medication, participants by medication class and generic term)

7.4.3.1 Immune Modulating Medication

Immune modulating medications are medications entered on an immune modulating medication form or available from the most current pre-defined list of immune modulating medications. This list is revisited whenever UMC WHO releases a new version and updated accordingly. The list of anatomic class, therapeutic class and generic name used for the selection at the time of the database lock will be provided.

The percentage of participants who received an immune modulating medication indicated for:

- management of adverse event
- premedication
- other use
- any use
- management of drug-related select adverse event (any grade, grade 3-5) by select AE category/ subcategory (EU/ROW Submissions)
- management of IMAEs (any grade, grade 3-5) by IMAE category (US Submission) will be reported separately for each treatment group (percentages of treated participants by medication class and generic term).

For each category/subcategory of drug-related select AEs (any grade, grade 3-5) and IMAEs (any grade, grade 3-5), the following will be reported for each treatment group:

- The total immune modulating medication treatment duration (excluding overlaps), duration of high dose of corticosteroid, initial dose of corticosteroid, and tapering duration (summary statistics)

Duration represents the total duration the participant received the medication of interest. If the participant took the medication periodically, then duration is the sum of all the lengths of use within the analysis. Initial dose represents the dose of the medication of interest received at the start of the event. In the case multiple medications started on the same date, the highest equivalent dose is chosen and converted to mg/kg by dividing by the participant's recent weight.

These analyses, except the ones related to IMAEs will be conducted using the 30-day safety window. The analyses related to IMAEs will be conducted using the 135-day safety window.

7.4.3.2 Subsequent Cancer Therapy

Subsequent therapy is defined as any systemic therapy, surgery, or radiotherapy for the cancer under study with a start date on or after the date of first dose of study drug (randomization date if participant was never treated).

- Number and percentage of participants receiving subsequent cancer therapies will be summarized for all randomized participants. Categories include:
  - Subsequent systemic therapy will be re-grouped by clinical team prior to database lock in the Data Presentation Plan (DPP).
  - Subsequent surgery for treatment of tumors
  - Subsequent radiotherapy for treatment of tumors

A by-participant listing of subsequent cancer therapy will also be produced for all randomized participants.

7.5 Efficacy

Analyses in this section will be tabulated for all randomized participants by treatment group as randomized, unless otherwise specified.

Unless stated otherwise, whenever a stratified analysis is specified, the following stratifications factors (recorded at randomization as per IRT) will be used:

- AJCC v8: Stage IIIA/IIIB, Stage IIIC, Stages IIID/IV (including all participants with mucosal melanoma, Stage III, Stage IVA, Stage IVB, and Stage IVC)
- Geographic Regions: USA/Canada/Australia, Europe, ROW

Anticipating the study population may not be equally distributed across levels in a given stratification factor, it is likely that the number of participants in some strata may be small. The stratum size with less than 30 participants would likely cause unreliable estimates in the stratified analyses[8,9,10,11].

Approved v2.0 930199387 2.0

Therefore, the following rule will be used when applicable and evaluated when the study is fully enrolled (at the earliest) and prior to unblinding (at the latest).

If the number of participants in at least one stratum is less than 20 across all randomized participants, the planned stratified analyses will be modified per the following steps.

- The marginal distribution of each level of a given stratification factor will be examined.
- The stratification factor with the lowest prevalence in a level will be excluded from the planned stratified analyses.
- After the above step, if there is still at least one stratum with less than 20 participants, the above steps will be repeated for the rest of stratification factors until all the stratum size is at least 20.

For assessing the key secondary objectives of this study, a hierarchical testing procedure[12] will be used so that the overall experiment-wise Type I error rate is two-sided 0.05.

Confidence intervals (CI) for primary and key secondary endpoint analyses included in hierarchy will be based on nominal significance level adjusted for primary endpoints and interim analyses to preserve overall type one error rate.

Alpha (α) for the CI will be the same as nominal significance level for hypothesis testing. CIs for other endpoints will be at the two-sided 95% level. All p-values reported will be two-sided. P-values will be rounded to the fourth decimal place. Point estimates and confidence bounds for efficacy variables will be rounded to the second decimal place.

7.5.1 Analysis of Recurrence-Free Survival (RFS)

The primary objective of the study is to compare the recurrence-free survival between treatment groups in all randomized participants.RFS determined by investigator will be compared between the treatment groups via stratified log-rank test among all randomized participants at a two-sided adjusted α level. The stratification factors will be AJCC v8 stage and Geographic Regions as recorded in IRT. The two-sided log-rank p-value will be reported.

Two interim analyses for superiority are planned. An O'Brien and Fleming type α-spending function will be employed to determine the nominal significance levels for the interim and final analyses

The estimate of the RFS hazard ratio between treatment groups will be calculated using a stratified Cox proportional hazards model, with treatment as the single covariate, stratified by above stratifications factors. Ties will be handled using the exact method. A two-sided 95% CI for the hazard ratio will also be presented.

The RFS distribution for each treatment group will be estimated using the KM product limit method and will be displayed graphically. A two-sided 95% CI for median RFS in each treatment group will be computed via the log-log transformation method. RFS rates at fixed time points (e.g., 6, 12, 18, 24 and 36 months, and yearly depending on the minimum follow-up) will be presented along with their associated 95% CIs. These estimates will be derived from the Kaplan Meier estimate and corresponding CIs will be derived based on Greenwood's[13] formula for variance derivation and the log-log transformation applied on the survivor function[14].

Approved v2.0 930199387 2.0

The source of RFS events will be summarized:

- documented recurrence
  - local
  - regional
    - ♦ in transit metastasis
    - ♦ regional node
  - distant metastasis
  - new primary invasive melanoma (excluding in situ melanoma)
- death (any cause)

The status of participants who are censored in the RFS KM analysis will be tabulated using following categories:

- Censored on randomization date
  - no baseline disease assessment
  - disease at baseline
  - no on-study disease assessment and no recurrence/death
- Censored on date of last disease assessment on-study
  - still on treatment
  - in follow-up
  - off study
    - lost to follow-up
    - participant withdrew consent
    - other

7.5.1.1 Supplemental Analysis of RFS

The main estimand analysis of RFS will be repeated based on supplemental estimand RFS analyses, censoring for subsequent therapy and diagnosis of second non-melanoma cancer. Under this strategy, RFS will be censored as described in Section 4.4.1.

In addition, to examine the assumption of proportional hazards in the Cox regression model, in addition to treatment, a time-dependent variable defined by treatment by time interaction will be added into the model. This treatment by time interaction is defined as the product of the treatment allocation and the non-missing binary time-dependent variable indicating if the RFS time is greater than or equal to 6 months. A two-sided Wald Chi-square p-value of less than 0.1, along with corresponding hazard plots, may indicate a potential nonconstant treatment effect. In that case, additional exploratory analyses may be performed.

A by-participant listing will be presented including treatment group, randomization date, first and last dose date, RFS duration and reason of event/censored for both main and supplemental

estimand definitions and associated date (on both primary and secondary definition) and subsequent therapy date.

Supplemental analyses of RFS will only be performed if the RFS primary analysis reaches statistical significance.

7.5.1.2 Sensitivity Analyses of RFS

Sensitivity analyses of RFS will also be performed:

1) RFS stratified analysis using stratification factors as obtained from the baseline CRF pages (instead of IRT). This analysis will be performed only if at least one stratification factor at randomization (as per IRT) and baseline are not concordant for at least 10% of all randomized participants.
2) RFS analysis using a 2-sided, un-stratified log-rank test and an un-stratified Cox proportional hazards model with treatment as the single covariate will be conducted.
3) RFS stratified analysis accounting for two or more consecutive missing scans. This analysis will be performed only if at least 10% of RFS events have two or more consecutive missing scans. A participant is considered to have two or more missing tumor assessments if the elapsed time between the RFS event and the last assessment prior to the event is greater than the time interval of two scheduled assessments. In case a subject has two or more consecutively missing disease assessments, the subject will be censored at the last evaluable disease assessment prior to the missing assessments. The main estimand of RFS will be used in this analysis.

Sensitivity analyses of RFS will only be performed if the RFS primary analysis reaches statistical significance.

7.5.1.3 Subset Analyses of RFS

To assess consistency of treatment effects in different subsets, a forest plot of the RFS un-stratified hazard ratio (and 95% CI) will be produced for the following subgroups. If a subset category has less than 20 participants per treatment group, HR will not be computed/displayed.

- Age categorization (≥ 18 to < 65, ≥ 65 to < 75, ≥ 65, ≥ 75)
- Sex (Male vs. Female)
- Race (as categorized: White/Black or African American/Asian (includes Indian, Chinese, Japanese, Korean, Asian Indian ,Malay , American Indian or Alaska Native) /Other (includes other, Native Hawaiian or Other Pacific Islander) source: eCRF)
- AJCC v8 stage at study entry (Source: eCRF): Stages IIIA/IIIB, Stage IIIC, Stages IIID/IV (Stage IV includes all participants with mucosal melanoma)
- Melanoma Subtype at study entry (cutaneous non-acral, cutaneous acral, mucosal, unknown primary, and other)
- Geographic Region (USA/Canada/Australia, Europe, Rest of World) as per eCRF
- Baseline PD-L1 Status (≥ 1%; <1%; indeterminate/unevaluable/not reported) (Source: clinical database)

Approved v2.0 930199387 2.0

- Baseline LAG-3 Status (≥ 1%; <1%; indeterminate/unevaluable/not reported) (Source: clinical database)
- BRAF (mutant/wild type/not reported) (source: eCRF)
- Baseline ECOG Performance Status (0, 1, >1, Not Reported)
- Baseline LDH level (≤ ULN, > ULN)

Number of events, median RFS along with 95% CI and RFS rates at 12 months along with 95% CI will be displayed for each treatment group.

7.5.1.4 Participant Follow-up for RFS

The currentness of follow-up for RFS, defined as the time between last disease assessment or randomization date (if the participant did not have on-study disease assessments) and data cut-off date, will be summarized by treatment group for all randomized participants. Participants who have an RFS event before data cut-off date will automatically have zero value for currentness of follow-up. Participants who have an RFS event and participants with last disease assessment date on data cut-off date will have zero value for currentness of follow-up. The currentness of follow-up will be categorized into the following categories: 0, >0 - < 3 months, 3 - < 6 months, 6 - < 9 months, 9 - < 12 months and ≥ 12 months.

7.5.2 Analysis of Overall Survival

Key secondary objective of the study is to compare the overall survival between treatment groups in all randomized participants.

Overall survival will be compared between the treatment groups at the interim and final analyses, using stratified log-rank test if primary RFS analysis is significant as per hierarchical testing strategy. The stratification factors will be AJCC v8 stage and Geographic Regions as recorded in IRT.

An O'Brien and Fleming[15] type α-spending function[16] will be employed to determine the nominal significance levels for the interim and final analyses. The hazard ratio between the treatment groups will be presented along with 100*(1- α)% CI (adjusted for interim) and 95% CI using a stratified Cox proportional hazards regression model. In addition, two-sided p-value will also be reported for the analysis of OS.

The OS distribution will be conducted using the KM techniques. A two-sided 95% CI for median OS in each treatment group will be computed via the log-log transformation method. OS rates at fixed time points (e.g. 6, 12, 18, 24, 30, 36 months and yearly depending on the minimum follow-up) will be presented along with their associated 95% CIs. These estimates will be derived from the Kaplan Meier estimate and corresponding CIs will be derived based on Greenwood formula for variance derivation and on log-log transformation applied on the survivor function.

The status of participants who are censored in the OS KM analysis will be tabulated for each treatment group using the following categories:

- On-study

Approved v2.0 930199387 2.0

  - on-treatment
  - in follow-up
- Off-study
  - lost to follow-up
  - withdrew consent
  - other

A by-participant listing will be presented including treatment group, randomization date, first and last dose date, whether the participant died, and if censored, the reason, event/censored date, and OS duration.

7.5.2.1 Supplemental Analyses of Overall Survival

To examine the assumption of proportional hazards in the Cox regression model, in addition to treatment, a time-dependent variable defined by treatment by time interaction will be added into the model. This treatment by time interaction is defined as the product of the treatment allocation and the non-missing binary time-dependent variable indicating if the OS time is greater than or equal to 6 months. A two-sided Wald Chi-square p-value of less than 0.1, along with corresponding hazard plots, may indicate a potential nonconstant treatment effect. In that case, additional exploratory analyses may be performed.

7.5.2.2 Sensitivity Analyses of Overall Survival

Sensitivity analyses of OS will only be performed if the OS primary analysis reaches statistical significance.

1) OS stratified analysis using stratification factors as obtained from the baseline CRF pages (instead of IRT). This analysis will be performed only if at least one stratification factor at randomization (as per IRT) and baseline are not concordant for at least 10% of all randomized participants.
2) OS analysis using a 2-sided, un-stratified log-rank test and an un-stratified Cox proportional hazards model with treatment as the single covariate will be conducted.

7.5.2.3 Subset Analyses of Overall Survival

To assess consistency of treatment effects in different subsets, a forest plot of the OS un-stratified hazard ratio (and 95% CI) will be produced for the following subgroups: Age categorization (≥ 18 to < 65, ≥ 65 to < 75, ≥ 65, ≥ 75)

- Sex (Male vs. Female)
- Race (as categorized: White/Black or African American/Asian (includes Indian, Chinese, Japanese, Korean, Asian Indian ,Malay , American Indian or Alaska Native) /Other (includes other, Native Hawaiian or Other Pacific Islander) source: eCRF)
- AJCC v8 stage at study entry (Source: eCRF): Stages IIIA/IIIB, Stage IIIC, Stages IIID/IV (Stage IV includes all participants with mucosal melanoma)
- Melanoma Subtype at study entry (cutaneous non-acral, cutaneous acral, mucosal, unknown primary, and other)

- Geographic Region (USA/Canada/Australia, Europe, Rest of World) as per eCRF
- Baseline PD-L1 Status (≥ 1%; <1%; indeterminate/unevaluable/not reported) (Source: clinical database)
- Baseline LAG-3 Status (≥ 1%; <1%; indeterminate/unevaluable/not reported) (Source: clinical database)
- BRAF (mutant/wild type/not reported) (source: eCRF)
- Baseline ECOG Performance Status (0, 1, >1, Not Reported)
- Baseline LDH level (≤ ULN, > ULN)

If a subset category has less than 20 participants per treatment group, HR will not be computed/displayed. Number of events, median OS along with 95% CI and OS rates at 12 months along with 95% CI will be displayed for each treatment group.

7.5.2.4 Current Status of OS Follow-up

The currentness of follow-up for survival, defined as the time between last OS contact (i.e., last known alive date or death date) and cutoff date, will be summarized descriptively (median, min, max, etc.) in months for all randomized participants. Participants who died, participants who have discontinued from the study, and participants with last known alive date on or after data cut-off date will have zero value for currentness of follow-up.

Minimum follow-up of OS for all randomized participants, defined as the time from the last participant's randomization date to cutoff date, will be displayed in months.

The currentness of follow-up will be categorized into the following categories: 0, >0 - < 3 months, 3 - < 6 months, 6 - < 9 months, 9 - < 12 months and ≥ 12 months.

In addition, the extent of follow-up, defined as the time between randomization and last OS contact (i.e., last known alive date or death date) will be summarized descriptively (median, min, max, etc.) in months for all randomized participants.

A by-participant listing will also be produced to accompany the participant time from last disease assessment date and last known alive date to cutoff date.

7.5.3 Analysis of Distant Metastasis-Free Survival (DMFS)

DMFS analyses will be performed in all randomized participants. Analysis results should be considered descriptive.

The main estimand analysis for DMFS will be conducted using a stratified Cox proportional hazards model, with treatment as the single covariate. The estimate of the DMFS hazard ratio, of BMS-986213 to nivolumab, will be calculated A two-sided 95% CI for the hazard ratio will also be presented.

The DMFS distribution for each treatment group will be estimated using Kaplan-Meier techniques. Median DMFS along with 95% CI will be constructed based on a log-log transformed CI for the survivor function. Rates at fixed time points (e.g., 6, 12, 18, 24, 30, 36, 42, 48, ... months, depending on the minimum follow-up) will be derived from the Kaplan Meier estimate and

Approved v2.0 930199387 2.0

corresponding confidence interval will be derived based on Greenwood formula for variance derivation and on log-log transformation applied on the survivor function.

The source of DMFS event will be summarized:

- distant metastasis
- death (any cause)

The status of participants who are censored in the DMFS KM analysis will be tabulated using following categories:

- Censored on randomization date
  - no baseline disease assessment
  - no on-study disease assessment with either no distant metastasis/death
  - disease at baseline
- Censored on date of last disease assessment on-study
  - still on treatment
  - in follow-up
  - off study
    - lost to follow-up
    - participant withdrew consent
    - other

7.5.4 Analysis of Progression-free Survival Through Next-line Therapy (PFS2)

One of the objectives of the study is to compare PFS2 between treatment groups in all randomized participants.

The estimate of the PFS2 hazard ratio by treatment group will be calculated using a stratified Cox proportional hazards model, with treatment as the single covariate. A two-sided 95% CI for the hazard ratio will also be presented.

The PFS2 distribution for each treatment group will be estimated using Kaplan-Meier techniques. Median PFS along with 95% CI will be constructed based on a log-log transformed CI for the survivor function. Rates at fixed time points (e.g. 6, 12, 18, 24, 30, 36 months, and yearly depending on the minimum follow-up) will be derived from the Kaplan Meier estimate and corresponding confidence interval will be derived based on Greenwood formula for variance derivation and on log-log transformation applied on the survivor function.

The source of PFS2 event will be summarized:

- recurrence / disease progression on next line therapy

Approved v2.0 930199387 2.0

- death (any cause)

The status of participants who are censored in the PFS2 KM analysis will be tabulated using following categories:

- Censored at the date of diagnosis of second non-melanoma primary cancer
- Censored on last known alive date
- Censored at randomization

A by-participant listing will be presented including treatment group, randomization date, first and last dose date, PFS2 status (event/censor) and associated date and reason, PFS2 duration, next line therapy date and second next line therapy date.

7.5.5 Interim Analyses

7.5.5.1 Interim Analyses of RFS

Two interim analyses for efficacy (RFS) will be performed based on number of events as detailed below.

The first interim analysis for RFS (RFS IA#1) will be performed when approximately 309 RFS events have occurred (~75% information fraction). At that time, a minimum follow-up of 14 months is expected for all randomized participants (ie, approximately 28 months from first participant randomized in the study). The significance boundaries at the interim analyses will be based on an O'Brien and Fleming alpha spending function. With an interim RFS analysis at 309 RFS events, the nominal significance level would be 0.020 (2-sided) and an observed HR of 0.765 or less would result in a statistically significant improvement, with power of 47%.

If RFS IA#1 is not statistically significant, the second interim analysis for RFS (RFS IA#2) will be performed when approximately 361 RFS events have occurred (~88% information fraction). At that time, a minimum follow-up of 24 months is expected for all randomized participants (ie, approximately 38 months from first participant randomized in the study). With an interim RFS analysis at 361 RFS events, the nominal significance level would be 0.028 (2-sided) and an observed HR of 0.793 or less would result in a statistically significant improvement at the interim analysis with cumulative power of 75%. A decision may be taken by BMS not to perform RFS IA#2 if, at the time the expected number of events for RFS IA#2 is reached, the predicted time frame to reach RFS FA number of events (410 events) is < 6 months. The DMC will be notified in the event that this decision is made. Alpha spending will be adjusted appropriately to not include RFS IA#2 if this is not performed. Please see Section 5.1 for specific details.

7.5.5.2 Interim Analysis of OS

Assuming the RFS result is statistically significant, there will be an interim analysis of OS performed by the DMC at the time of RFS statistical significance (either at RFS IA#1, RFS IA#2, or RFS FA). If RFS is statistically significant at one of the interims (RFS IA#1, RFS IA#2) but OS is not statistically significant, an additional interim OS analysis will be performed by the DMC at

Approved v2.0 930199387 2.0

approximately 52 months from the randomization of the first participant (~59% information fraction, 183 OS events out of 309). If this OS IA is not statistically significant, another OS IA will be performed at approximately 71 months from the randomization of the first participant (~81% information fraction, 250 OS events out of 309). Please see Section 5.2 for specific details.

7.5.5.3 Interim Analyses of RFS and OS: Additional Details

These interim analyses for RFS and OS will allow for early formal testing for superiority, and the boundaries for declaring superiority will be derived based on the actual number of events using Lan-DeMets spending function with O'Brien and Fleming type of boundary based on the information fraction observed.

7.6 Safety

7.6.1 General Methods for Adverse Events

Drug-related AEs are those events with relationship to study drug "Related", as recorded on the CRF. If the relationship to study drug is missing, the AE will be considered as drug-related.

Serious adverse events consist of AEs deemed serious by the Investigator and flagged accordingly in the CRF and clinical database.

Adverse events leading to study drug discontinuation are AEs with action taken regarding study drugs = "Drug Withdrawn".

Adverse events leading to dose delay are AEs with action taken regarding study drugs = "Dose Delayed".

Adverse events will be coded using the Medical Dictionary for Regulatory Activities (MedDRA), and the most recent version of the dictionary at the time of the database lock will be used. Adverse event results will be graded for severity using the NCI Common Terminology Criteria for Adverse Events (CTCAE) and the version of the criteria specified in the protocol will be used.

Unless otherwise specified, the AE summary tables will be restricted to on-treatment events regardless of the causality.

Analyses in this section will be tabulated for all treated participants by treatment group as treated, unless otherwise specified.

7.6.2 Deaths

Deaths will be summarized by treatment group:

- All deaths, reasons for death.
- Deaths within 30 days of last dose received, reasons for death.
- Deaths within 135 days of last dose received, reasons for death.
- Overall summary of AEs leading to death within 135 days of last dose received
- Overall summary of drug-related AEs leading to death within 135 days of last dose received
- Overall summary of SAEs leading to death within 135 days of last dose received

Approved v2.0 930199387 2.0

- Overall summary of AEs leading to death within 135 days of last dose excluding preferred terms associated with malignant neoplasm progression

A by-participant listing of deaths will be provided for the All Enrolled population. A listing of all preferred terms defining malignant neoplasm progression will be included.

7.6.3 Serious Adverse Events

Serious adverse events will be summarized by treatment group:

- Overall summary of SAEs by worst CTC grade (any grade, grade 3-4, grade 5) presented by SOC/PT.
- Overall summary of drug-related SAEs by worst CTC grade (any grade, grade 3-4, grade 5) presented by SOC/PT.

All analyses will be conducted using the 30-days safety window.
A by-participant SAE listing will be provided for all enrolled participants population.

7.6.4 Adverse Events Leading to Discontinuation of Study Therapy

AEs leading to discontinuation will be summarized by treatment group:

- Overall summary of AEs leading to discontinuation by worst CTC grade (any grade, grade 3-4, grade 5) presented by SOC/PT.
- Overall summary of drug-related AEs leading to discontinuation by worst CTC grade (any grade, grade 3-4, grade 5) presented by SOC/PT.

The analyses will be conducted using the 30-days safety window.

A by-participant AEs leading to discontinuation listing will be provided.

7.6.5 Adverse Events Leading to Dose Modification

AEs leading to dose delay will be summarized by treatment group:

- Overall summary of AEs leading to dose delay/reduction by worst CTC grade (any grade, grade 3-4, grade 5) presented by SOC/PT.
- Overall summary of drug-related AEs leading to dose delay/reduction by worst CTC grade (any grade, grade 3-4, grade 5) presented by SOC/PT.

The analysis will be conducted using the 30-days safety window.

A by-participant AEs leading to dose delay listing will be provided.

7.6.6 Adverse Events

Adverse events will be summarized by treatment group.

The following analyses will be conducted using the 30-days safety window only:

Approved v2.0 930199387 2.0

- Overall summary of any AEs by worst CTC grade (1, 2, 3, 4, 5, not reported, total) presented by SOC/PT.
- Overall summary of any AEs presented by worst CTC grade (any grade, grade 3-4, grade 5) by SOC/PT. This table will be restricted to events with an incidence greater or equal to 5% in any treatment group.
- Overall summary of any non-serious AEs presented by SOC/PT. This table will be restricted to events with an incidence greater or equal to 5% in any treatment group.
- Overall summary of any AEs that required immune modulating medication by worst CTC grade (any grade, grade 3-4, grade 5) presented by SOC/PT.
- Overall summary of drug-related AEs by worst CTC grade (1, 2, 3, 4, 5, not reported, total) presented by SOC/PT.The following analyses will be conducted using the 30 days safety window and repeated using the 135 days safety window:
- Overall summary of drug-related AEs by worst CTC grade (any grade, grade 3-4, grade 5) presented by SOC/PT.

A by-participant AE listing will be provided. A by-participant listing of any AE requiring immune modulating medications will also be provided.

7.6.7 Select Adverse Events (EU/ROW Submissions)

7.6.7.1 General Methods for Select Adverse Events

The select Adverse Events (select AEs) consist of a list of preferred terms grouped by specific category. AEs that may differ from or be more severe than AEs caused by non-immunotherapies and AEs whose early recognition and management may mitigate severe toxicity are included as select AEs. Categories of select AEs may include subcategories.

The list of MedDRA preferred terms used to identify select adverse events is revisited biannually for MedDRA versioning and updated accordingly. The preferred terms used for the selection at the time of the database lock will be provided by categories/subcategories.

In addition to the frequency and worst severity of select AEs, time-to onset, time-to resolution, and time-to resolution where immune modulating medication was initiated will be analyzed for each specific category/subcategory of drug-related select AEs when applicable.

Further details on the definitions time-to onset and time-to resolution are described in APPENDIX 1.

Unless otherwise specified, analyses will be performed by select AE category. Analyses will also be repeated by subcategory of endocrine events.

7.6.7.2 Incidence of Select AE

Select AEs will be summarized by treatment group for each category/subcategory.

The following analyses will be conducted using the 30 day safety window only:

- Overall summaries of any (endocrine and separately non-endocrine) select AEs by worst CTC grade (any grade, grade 3-4, grade 5) presented by Category or Subcategory/PT.

Approved v2.0 930199387 2.0

- Overall summaries of any (endocrine and separately non-endocrine) select AEs leading to discontinuation by worst CTC grade (any grade, grade 3-4, grade 5) presented by Category or Subcategory /PT.
- Overall summaries of any (endocrine and separately non-endocrine) drug-related select AEs by worst CTC grade (any grade, grade 3-4, grade 5) presented by Category or Subcategory/PT.
- Overall summaries of any (endocrine and separately non-endocrine) drug-related select AEs leading to discontinuation by worst CTC grade (any grade, grade 3-4, grade 5) presented by Category or Subcategory /PT.
- Overall summaries of any (endocrine and separately non-endocrine) serious select AEs by worst CTC grade (any grade, grade 3-4, grade 5) presented by Category or Subcategory/PT.
- Overall summaries of any (endocrine and separately non-endocrine) serious select AEs leading to discontinuation by worst CTC grade (any grade, grade 3-4, grade 5) presented by Category or Subcategory /PT.
- Overall summaries of any (endocrine and separately non-endocrine) drug-related serious select AEs by worst CTC grade (any grade, grade 3-4, grade 5) presented by Category or Subcategory/PT.
-
- A by-participant select AE listing will be provided.

7.6.7.3 Time-to Onset of Select AE

Time-to onset of the following specific events will be summarized separately for each category/subcategory.

Time-to onset analyses are restricted to treated participants who experienced at least one (and endocrine) drug-related select AE in the category/subcategory. The analyses will be conducted using the 30-days safety window.

Additional details regarding the time-to onset definition are described in time-to onset definition subsection of APPENDIX 1.

7.6.7.4 Time-to Resolution of Select AE

Time-to resolution of the following specific events will be summarized separately for each category/subcategory.

- Time-to resolution of any (and endocrine) drug-related select AE (any grade, grade 3-5) by treatment group
- Time-to resolution of any (and endocrine) drug-related select AE (any grade, grade 3-5) where immune modulating medication was initiated, by treatment group

Time-to resolution where immune modulating medication was initiated analyses are restricted to treated participants who experienced the specific events and who received immune modulating medication during the longest select AE.

The analyses will be conducted using the 30 day safety window.

The following summary statistics will be reported: percentage of participants with resolution of the longest select AE, median time-to resolution along with 95% CI (derived from Kaplan-Meier estimation) and ranges.

See time-to resolution definition subsection of APPENDIX 1 for additional details.

7.6.8 Immune-Mediated Adverse Events (US Submission)

7.6.8.1 General Methods for Immune-Mediated Adverse Events

In order to further characterize AEs of special clinical interest, analysis of immune-mediated AEs (IMAE) will be conducted. IMAEs are specific events (or groups of PTs describing specific events) that include pneumonitis, diarrhea/colitis, hepatitis, nephritis/renal dysfunction, rash, endocrine (adrenal insufficiency, hypothyroidism/thyroiditis, hypothyroidism, thyroiditis, hyperthyroidism, diabetes mellitus, and hypophysitis), and other specific events, considered as potential immune-mediated events by investigator that meet the definition summarized below:

- those occurring within 135 days of the last dose
- regardless of causality
- treated with immune-modulating medication OR endocrine AEs (of note, endocrine AEs such as adrenal insufficiency, hypothyroidism/thyroiditis, hypothyroidism, thyroiditis, hyperthyroidism, diabetes mellitus, and hypophysitis are considered IMAEs regardless of immune-modulating medication use, since endocrine drug reactions are often managed without immune-modulating medication)
- with no clear alternate etiology based on investigator assessment, or with an immune-mediated component

The list of MedDRA preferred terms used to identify IMAEs is revisited biannually for MedDRA versioning and updated accordingly. The preferred terms used for the selection at the time of the database lock by categories will be provided.

For the purposes of IMAE analysis, the following definitions will be used:

- Participants who were re-challenged/re-initiated with nivolumab or nivo+rela FDC are defined as receiving the next active study medication after onset of the IMAE that led to dose delay (re-initiation date) and after:
  - IMAE improvement or resolution, for the specific IMAE preferred term that led to dose delay
  - IMAE resolution, for any other preferred term in the same IMAE class
- Participants who have had a positive re-challenge (or recurrence of IMAE), defined as either:
  - For the specific IMAE preferred term that led to dose delay, either:
    - ♦ Worsening in grade on or after re-initiation of nivolumab or nivo+rela FDC
    - ♦ If resolved, re-occurrence of the preferred term at any severity grade on or after re-initiation of nivolumab or nivo+rela FDC
  - For any other preferred term in the same IMAE class:
    - ♦ Any occurrence of new event(s) within the same IMAE class on or after re-initiation of nivolumab or nivo+rela FDC

Approved v2.0 930199387 2.0

IMAEs will be summarized by treatment group for each immune-mediated category / PT using the 135 days safety window:

- Overall summary of non-endocrine IMAEs by worst CTC grade (any grade, grade 3-4, grade 5) where immune modulating medication was initiated presented by Category / PT.
- Overall summary of endocrine IMAEs by worst CTC grade (any grade, grade 3-4, grade 5) presented by Category / PT.
- Overall summary of non-endocrine IMAEs leading to discontinuation by worst CTC grade (any grade, grade 3-4, grade 5) where immune modulating medication was initiated presented by Category / PT.
- Overall summary of endocrine IMAEs leading to discontinuation by worst CTC grade (any grade, grade 3-4, grade 5) presented by Category / PT.
- Overall summary of non-endocrine IMAEs leading to dose delay or reduction by worst CTC grade (any grade, grade 3-4, grade 5) where immune modulating medication was initiated presented by Category / PT
- Overall summary of endocrine IMAEs leading to dose delay or reduction by worst CTC grade (any grade, grade 3-4, grade 5) presented by Category / PT.
- Summaries of time-to onset and time-to resolution of non-endocrine IMAEs where immune modulating medication was initiated presented by Category.
- Summaries of time-to onset and time-to resolution of endocrine IMAEs presented by Category.
- A by-participant listing of IMAEs will be provided. By-participant listings of time-to resolution for longest IMAEs cluster (any grade and grade 3-5 in separate summaries) will also be provided.

A by-participant listing of AEs considered as immune-mediated events per investigator but not qualified for IMAEs definition will also be provided.

In addition, for all treated participants who experienced at least one IMAE, the following data presentation will be provided by IMAE category using the 135-days safety window:

- Summary of participants with an IMAE leading to dose delay with the dose delay confirmed, as defined in Section 7.4.2.1
- Summary of participants who were re-challenged/re-initiated with nivolumab or nivo + rela FDC
- Summary of participants who have had a positive re-challenge (or recurrence of IMAE)

7.6.9 Other Events of Special Interest

Other events of special interest (OEOSI) consist of a list of preferred terms grouped by specific category. The list of MedDRA preferred terms used to identify OEOSI is revisited biannually for MedDRA versioning and updated accordingly. The preferred terms used for the selection at the time of the database lock by categories will be provided.

OEOSI will be summarized by treatment group for each category.

The following analyses will be conducted using the 135-days safety window:

Approved v2.0 930199387 2.0

- Overall summary of OEOSI by worst CTC grade (any grade, grade 3-4, grade 5) presented by Category / PT
- Overall summary of drug-related OEOSI by worst CTC grade (any grade, grade 3-4, grade 5) presented by Category / PT

A by-participant listing of OEOSI will be provided.

7.6.10 Multiple Events

Analyses that take into account the multiple occurrences of a given adverse event will be conducted. To prepare these analyses, the CRF data will be processed according to the BMS Global Standards Specifications in order to collapse adverse event records into unique records based on the preferred term. These data will be presented as the rate per 100 person-years of exposure. These analyses will take into account all on-treatment events (allowing more than 1 event per participant) and the total exposure time. The person-year exposure will be computed as the sum over the participants' exposure expressed in years where the exposure time is defined as

- (Date of last dose of study treatment - date of first dose of study treatment + 31)/365.25, for participant who are off study treatment and were followed for at least 30 days after last dose of study treatment.
- (Last known alive date - date of first dose of study treatment +1)/365.25, for participants who are still on-treatment or who are off study treatment and were followed less than 30 days after last dose of study treatment.

The following summary tables will be provided using a 30-days window:

- A table showing the total number and rate (exposure adjusted) of occurrences for all AEs.
- A table showing the total number and rate (exposure adjusted) of occurrences for AEs occurring in at least 5% of participants in any treatment group.

In addition, the rate (exposure adjusted for different time intervals) and its 95% CI evaluated will be displayed graphically for each treatment group. The 95% CI of the rate per 100 person-year of exposure will be derived using the normal approximation and variance estimation proposed in Cook and Lawless[17]. This analysis will be limited to the rate of all AEs and all drug-related AEs.

A listing displaying the unique instances of all AEs, i.e., after duplicates have been eliminated and overlapping and contiguous occurrences of the same event (i.e. same PT) have been collapsed will be provided. No formal comparisons will be made between treatment groups.

7.6.11 Laboratory Parameters

7.6.11.1 General Methods for Laboratory Tests

Clinical laboratory parameters (hematology, serum chemistry, and electrolytes) will be evaluated.

Laboratory tests will be graded using the NCI Common Terminology Criteria.

Clinical laboratory data will be first analyzed using International System of Units (SI).

Approved v2.0 930199387 2.0

Analyses will be repeated using US conventional units.

The analysis population for each laboratory test is restricted to treated participants who underwent that laboratory test. Analyses will be presented by grade (0, 1, 2, 3, 4).

- Laboratory analyses may be repeated by grade groups (grades 1-4, grade 3-4) for labeling purposes and will be indicated in the DPP if applicable.

A by-participant listing of differences in categorization of SI and US laboratory test results will be provided.

7.6.11.2 Hematology

The following will be summarized by treatment group as worst CTC grade on-treatment per participant and as shift table of worst on-treatment CTC grade compared to baseline CTC grade per participant: hemoglobin (HB), platelets, white blood counts (WBC), absolute neutrophils count (ANC) and lymphocyte count (LYMPH).

The analyses will be conducted using the 30-day safety window.

A by-participant listing of these laboratory parameters will be provided.

7.6.11.3 Serum Chemistry

The following will be summarized by treatment group as worst CTC grade on-treatment per participant and as shift table of worst on-treatment CTC grade compared to baseline CTC grade per participant: ALT, AST, alkaline phosphatase (ALP), total bilirubin and creatinine.

The analyses will be conducted using the 30-days safety window.

A by-participant listing of these laboratory parameters will be provided.

7.6.11.4 Electrolytes

The following will be summarized by treatment group as worst CTC grade on-treatment per participant and as shift table of worst on-treatment CTC grade compared to baseline CTC grade per participant: sodium (high and low), potassium (high and low), calcium (high and low), and Glucose Serum (fasting hyperglycemia and hypoglycemia regardless of fasting status).

The analyses will be conducted using the 30-days safety window.

A by-participant listing of these laboratory parameters will be provided.

7.6.11.5 Additional Analyses

In addition, further analyses on specific laboratory parameters will be performed by treatment group:

Abnormal Hepatic Function Test

The number of participants with the following laboratory abnormalities from on-treatment evaluations will be summarized by treatment group:

- ALT or AST > 3 x ULN, > 5 x ULN, > 10 x ULN and > 20 x ULN

Approved v2.0 930199387 2.0

- Total bilirubin > 2 x ULN
- ALP > 1.5 x ULN
- Concurrent (within 1 day) ALT or AST > 3 x ULN and total bilirubin > 1.5 x ULN
- Concurrent (within 30 days) ALT or AST > 3 x ULN and total bilirubin > 1.5 x ULN
- Concurrent (within 1 day) ALT or AST > 3 x ULN and total bilirubin > 2 x ULN
- Concurrent (within 30 days) ALT or AST > 3 x ULN and total bilirubin > 2 x ULN

The analyses will be conducted using the 30-days safety window.

A by-participant listing of these specific abnormalities will be provided.

Abnormal Thyroid Function Test

The number of participants with the following laboratory abnormalities from on-treatment evaluations will be summarized by treatment group:

- TSH value > ULN and
  - with baseline TSH value ≤ ULN
  - with at least one FT3/FT4 test value < LLN within 2-week window after the abnormal TSH test
  - with all FT3/FT4 test values ≥ LLN within 2-week window after the abnormal TSH test
  - with FT3/FT4 missing within 2-week window after the abnormal TSH test.
- TSH < LLN and
  - with baseline TSH value ≥ LLN
  - with at least one FT3/FT4 test value > ULN within 2-week window after the abnormal TSH test
  - with all FT3/FT4 test values ≤ ULN within 2-week window after the abnormal TSH test
  - with FT3/FT4 missing within 2-week window after the abnormal TSH test

The analyses will be conducted using the 30-days safety window. A subject data listing of TSH values will be provided.

7.6.12 Vital Signs

Vital signs collected on the CRF will be provided in listing.

7.6.13 Electrocardiogram

All of the available ECG parameter values collected at baseline, from each participant will be included in the ECG data set. All recorded ECG parameter values will be included in the data listings.

Baseline values are defined as the last recorded values prior to the first dosing. Although individual values for QT, QTcB, and QTcF will be presented in the data listings, only QTcF will be analyzed and discussed in the report. For other ECG parameters such as heart rate (HR), QT, QRS, and PR,

Approved v2.0 930199387 2.0

summary measures at baseline (n, mean, standard deviation, median, minimum, and maximum) will be provided.

Listing:

- A by-participant listing of all ECG measures
- A listing of abnormal ECG interpretations

7.6.14 Physical Measurements

Physical measurements will be listed by participant.

7.6.15 Non-Protocol Medical Procedures

Non-protocol medical procedures (Diagnostic Procedures & Medical Treatment Procedures) will be listed by participant.

7.6.16 Pregnancy

A by-participant listing of pregnancy tests results will be provided for randomized female participants.

7.6.17 Adverse Events By Subgroup

Overall summary of any AEs and drug-related AEs by worst CTC grade (any grade, grade 3-4, grade 5) for each treatment group for the following subgroups:

- Sex (Male vs. Female)
- Race (as categorized: White/Black or African American/Asian (includes Indian, Chinese, Japanese, Korean, Asian Indian, Malay, American Indian or Alaska Native) /Other (includes other, Native Hawaiian or Other Pacific Islander) source: eCRF)
- Age ($\geq$ 18 - < 65 vs. $\geq$ 65)
- Region (USA/Canada/Australia, Europe, ROW)

These analyses will be conducted using the 30-days safety window only.

7.6.18 Analysis of SARS-CoV-2

Sensitivity analyses of RFS and OS due to COVID-19 will be detailed in an exploratory SAP. These analyses will not be included in the CSR.

The potential association between exploratory measurements of SARS-CoV-2 serology (anti-SARS-CoV-2 total or IgG), from serum samples collected at baseline and selected endpoints related to safety, efficacy, and/or biomarkers will be analyzed in an exploratory SAP and will not be included in the CSR.

7.7 Pharmacokinetics

7.7.1 Pharmacokinetic Concentrations

Summary:

Summary statistics will be provided for the following pharmacokinetic serum concentrations by treatment, cycle, study day and time for based on the relatlimab and Nivolumab PK Participants.

Approved v2.0 930199387 2.0

- Serum concentrations of relatlimab
- Serum concentrations of nivolumab

Listing:

- relatlimab and nivolumab serum concentrations

These data may also be pooled with other datasets for population PK analysis, which will be presented in a separate report.

7.7.2 Ctrough and Ceoi

Ctrough and Ceoi of relatlimab and nivolumab will be listed and summarized by treatment, cycle and study day. The geometric means of Ctrough vs. cycle and day will be plotted using the PK Population.

The concentration vs time data obtained in this study may be combined with data from other studies in the clinical development program to develop a population PK model. This model will be used to evaluate the effects of intrinsic and extrinsic covariates on the PK of relatlimab. Model determined exposures will be used for exposure-response analyses of selected efficacy and safety end points. Results of population PK and exposure-response analyses will be reported separately.

7.8 Biomarkers

Analyses for PD-L1 and LAG-3 are described below. The analyses specified in the sections below will be performed for all randomized participants when comparing treatment arms, whereas they will be performed on biomarker quantifiable subjects when comparing PD-L1 (respectively LAG-3) subcategories within each treatment arm.

7.8.1 Analysis of PD-L1 Expression

Analyses of PD-L1 expression are descriptive in nature and intended to examine the distribution of PD-L1 expression and assess the association between PD-L1 expression and safety/efficacy measures.

PD-L1 expression will be analyzed both as continuous and as categorical endpoint. Categorical PD-L1 levels are defined as follows:

- PD-L1 expression ≥ 1%
- PD-L1 expression < 1%

PD-L1 indeterminate/unevaluable/not reported will not be included for within treatment arm comparisons.

Additional cut off values may also be explored in another analysis plan.

The following analyses will be conducted. Unless otherwise specified, each analysis will be performed for both PD-L1 approaches. Analyses involving treatment groups comparison will be

Approved v2.0 930199387 2.0

performed for the comparison of Arm B vs Arm A. Unless otherwise specified, baseline PD-L1 will be considered in the analyses.

- Descriptive statistics of PD-L1 expression:
  - Listing of all PD-L1 data, all randomized participants
  - Summary of tumor specimen acquisition and characteristics, all randomized participants
  - Summary statistics of PD-L1 expression (i.e., PD-L1 continuous endpoint) by treatment groups and overall.
  - Box plot of PD-L1 expression by treatment group and overall
  - Cumulative distribution plot of PD-L1 expression versus population percentile by treatment group and overall
  - Waterfall plots of individual PD-L1 expression by treatment group
- Analyses for the RFS endpoint
  - For each of categorical PD-L1 level (>=1% vs <1%):
    - RFS function for each treatment group will be estimated using the Kaplan-Meier product limit method and will be displayed graphically.
      - The first KM plot will display the comparison of PD-L1 positive participants versus PD-L1 negative participants overlaid (1 graph by treatment arm).
      - The second KM plot will display the comparison between the two treatment arms overlaid (1 graph by level of PD-L1, including a category for PD-L1 indeterminate/unevaluable/not reported).

      A two-sided 95% CI for median RFS in each treatment group will be computed via the log-log transformation method.
  - A Cox proportional hazards regression model will be fitted for RFS with treatment, categorical PD-L1 (≥ 1% vs. < 1%), and treatment by categorical PD-L1 interaction. Although the study is not designed to have appropriate power to formally test the interaction of the model, an interaction test at significance level of 0.2 will warrant further exploration and the following statistics will be reported:
    - Interaction p-value
    - Hazard ratio of treatment vs. control and its associated 95% CI will be reported for each of the categorical PD-L1 level ((≥ 1% vs. < 1% vs. indeterminate/unevaluable/not reported).
    - Hazard ratio of categorical PD-L1 (≥ 1% vs. < 1%) and its associated 95% CI will be reported for each treatment group.
- Same analyses will be repeated for OS and DMFS endpoints

7.8.2 Analysis of LAG-3 Expression

All these analyses performed above for PD-L1 will be repeated on LAG-3 expression. Cut-off value used for LAG-3 expression will be 1%.

Approved v2.0 930199387 2.0

7.9 Immunogenicity Analysis

Further details on immunogenicity background and rationale, definitions, are described in APPENDIX 3.

Population for Analyses

Analysis of immunogenicity data will be based on ADA evaluable participants defined as all treated participants with baseline and at least 1 post-baseline immunogenicity assessment. Analysis dataset and data listing will include all available ADA samples. However, participant-level ADA status will be defined based on only adequate samples (e.g., excluding 1-hour post-infusion samples when clearly indicated).

Definitions

Sample ADA Status**:**

- Baseline ADA-positive sample: ADA is detected in the last sample before initiation of treatment
- Baseline ADA-negative sample: ADA is not detected in the last sample before initiation of treatment
- ADA-positive sample: After initiation of treatment, (1) an ADA detected (positive seroconversion) sample in a participant for whom ADA is not detected at baseline, or (2) an ADA detected sample with ADA titer to be at least 4-fold or greater (≥) than baseline positive titer
- ADA-negative sample: After initiation of treatment, ADA not positive sample relative to baseline

Next, using the sample ADA status, participant ADA status is defined as follows: Participant ADA Status:

- Baseline ADA-positive participant: A participant with baseline ADA-positive sample
- **ADA-positive participant:** A participant with at least one ADA positive-sample relative to baseline at any time after initiation of treatment

1) Persistent Positive (PP): ADA-positive sample at 2 or more consecutive timepoints, where the first and last ADA-positive samples are at least 16 weeks apart
2) Not PP-Last Sample Positive: Not persistent positive with ADA-positive sample at the last sampling timepoint
3) Other Positive: Not persistent positive but some ADA-positive samples with the last sample being negative
4) Neutralizing Positive: At least one ADA-positive sample with neutralizing antibodies detected

- ADA-**negative participant**: A participant with no ADA-positive sample after the initiation of treatment.

(Note: 16 weeks was chosen based on a long half-life of IgG4.)

Table:

The number (%) of participants with the following anti-drug responses will be reported by dose, if applicable, and overall.

- Baseline ADA-positive
- ADA-positive
  - Persistent Positive (PP)
  - Not PP - Last Sample Positive
  - Other Positive
- ADA-positive with Neutralizing Positive
- ADA-negative

Listing:

All collected immunogenicity samples will be listed with flags indicating baseline-positive sample, ADA-positive sample or ADA-negative sample, together with the associated drug concentration.

Clinical Implications:

Clinical implications of positive ADA may be explored by a comparison of ADA-positive participants to ADA-negative participants. Effect of immunogenicity on clearance of study drugs may be explored by comparison of clearance estimates (determined by PPK analysis), if appropriate. Effect of immunogenicity on safety will be explored by examining the frequency and type of AEs of special interest such as hypersensitivity/infusion reaction. Summary tables for incidence of each preferred terms and overall as a category of AEs will be provided, if the number of participants is of sufficient size (e.g., at least 10 participants). Otherwise, individual participant's safety profile will be examined and described based on a listing. Association between trough concentrations of study drugs and ADA assessments may be explored, as needed. Assessments of effect of ADA on efficacy will be explored.

7.10 Clinical Outcomes Assessments

Unless otherwise stated, the analysis of EORTC QLQ-C30 and FACIT-GP5 will be performed on all randomized adult participants and the EQ-5D-5L will be performed on all randomized participants.

- Analysis datasets and data listings will include all available COA assessments. However, COA analyses will be performed based on valid assessments only.

7.10.1 EORTC QLQ-C30

For EORTC QLQ-C30, all scales and single items are scored on categorical scales and linearly transformed to 0-to-100 scales with higher scores for a functional scale representing higher or healthier levels of functioning, higher scores for the GHS/QOL scale representing higher levels of GHS/QOL and higher scores for a symptom scale or item representing higher level of symptoms

Approved v2.0 930199387 2.0

or problems. A score difference of 10 is used as an estimate of a clinically minimum important difference (MID) for the scales of the EORTC QLQ-C30[18].

The following descriptive analyses will be conducted by treatment group, for each timepoint at which the EORTC QLQ-C30 questionnaire was assessed (including baseline, on-study, and follow-up timepoints):

- EORTC QLQ-C30 questionnaire available data rate where the numerator is the number of participants submitting a valid EORTC QLQ-C30 questionnaire assessment at the designated time point and the denominator is a fixed denominator defined as the number of all randomized participants ≥ 18 years of age (i.e., all participants who consented and were eligible to participate in the EORTC QLQ-C30 data collection at baseline).
- EORTC QLQ-C30 questionnaire completion rate, where the numerator is the number of participants submitting a valid EORTC QLQ-C30 questionnaire assessment at the designated time point and the denominator is a variable denominator defined as the number of participants expected to have a EORTC QLQ-C30 assessment at the designated time point. The number of participants expected to have a EORTC QLQ-C30 assessment at the designated timepoint is defined as follows: any participant ≥ 18 years of age is expected to have a completed EORTC QLQ-C30 assessment if the nominal date for that visit is before both the end of study/data cut-off date and before the last known alive date (i.e., the number of participants randomized minus participants who died, or discontinued from the study due to any reason at or before that visit).
- Mean score and mean change from baseline in EORTC QLQ-C30 GHS/QOL, functional scales and symptom scales, and other individual items will be summarized using descriptive statistics (N, mean with SD and 95% CI, median, first and third quartiles, minimum, maximum) for all scales at each assessment time point (post-baseline assessment timepoints up to Follow-Up Visit 2) by treatment group. Descriptive statistics of the EORTC QLQ-C30 scores at baseline will also be summarized by treatment group.
  - A line graph summarizing the mean changes from baseline will be produced.

7.10.2 EuroQoL EQ-5D-5L

For EQ-5D-5L, higher scores represent better HRQoL; therefore, an increase in score reflects improvement while a decrease in score reflects worsening in HRQoL. A change from baseline of 7 is considered the MID for the EQ-5D-5L VAS[19]. There is no MID available for the EQ-5D-5L utility index.The following descriptive analyses will be conducted by treatment group, for each timepoint at which the EQ-5D-5L questionnaire was assessed (including baseline, on-study, follow-up, and survival follow-up timepoints):

- EQ-5D-5L questionnaire available data rate where the numerator is the number of participants submitting a valid EQ-5D-5L questionnaire assessment at the designated time point and the denominator is a fixed denominator defined as the number of randomized participants (i.e., all participants who consented and were eligible to participate in the EQ-5D-5L data collection at baseline).
- EQ-5D-5L questionnaire completion rate, where the numerator is the number of participants submitting a valid EQ-5D-5L questionnaire assessment at the designated time point and the

Approved v2.0 930199387 2.0

denominator is a variable denominator defined as the number of participants expected to have a EQ-5D-5L assessment at the designated time point. The number of participants expected to have an ED-5D-5L assessment at the designated timepoint is defined as follows: any participant is expected to have a completed EQ-5D-5L assessment if the nominal date for that visit is before both the end of study/data cut-off date and before the last known alive date (i.e., the number of participants randomized minus participants who died, or terminated the study due to any reason at or before that visit).

- A by-participant listing of the level of problems in each dimension, corresponding to EQ-5D-5L health state (i.e., 5-digit vector), EQ-5D-5L utility index score, and EQ-5D-5L VAS score will be provided.
- Proportion of participants reporting problems for the 5 EQ-5D-5L dimensions at each assessment time point will be summarized by level of problem and by treatment group. Percentages will be based on number of participants assessed at assessment time point.
- For the remapped EQ-5D-5L utility index based on EEPRU model and VAS scores, separately:
  - Mean score and mean change from baseline at each assessment time point will be summarized by treatment group using descriptive statistics (N, mean with SD and 95% CI, median, first and third quartiles, minimum, maximum). Descriptive statistics of the EQ-5D-5L scores at baseline will also be summarized by treatment group.
  - A line graph summarizing the mean changes from baseline will be produced.

7.10.3 FACIT-GP5

Unless otherwise specified, the analysis of FACIT-GP5 will be performed in all randomized participants. The following descriptive analyses will be conducted.

- FACIT-GP5 available data rate where the numerator is the number of participants submitting a valid FACIT-GP5 questionnaire assessment at the designated time point and the denominator is a fixed denominator defined as the number of randomized participants ≥ 18 years of age (i.e., all participants who consented and were eligible to participate in the FACIT-GP5 data collection at baseline).
- FACIT-GP5 questionnaire completion rate, where the numerator is the number of participants submitting a valid FACIT-GP5 questionnaire assessment at the designated time point and the denominator is a variable denominator defined as the number of participants expected to have a FACIT-GP5 assessment at the designated time point. The number of participants expected to have a FACIT-GP5 assessment at the designated timepoint is defined as follows: any participant ≥ 18 years of age is expected to have a completed FACIT-GP5 assessment if the nominal date for that visit is before both the end of study/data cut-off date and before the last known alive date (i.e., the number of participants randomized minus participants who died, or discontinued from the study due to any reason at or before that visit).
- Number and proportion of participants endorsing each response option at each assessment timepoint by treatment group. Percentages will be based on number of participants assessed at assessment time point. A stacked bar chart of responses at each timepoint by treatment group will be created.

Approved v2.0 930199387 2.0

7.11 COVID-19 Related Analyses

7.11.1 COVID-19 Related Disposition Events

- Number of participants enrolled but not randomized due to COVID-19 along with the specific reason for not being randomized due to COVID-19 will be summarized.
- Number of participants randomized but not treated due to COVID-19 along with the specific reason for not being treated due to COVID-19 will be tabulated by treatment group as randomized.
- Number of participants who discontinued study treatment due to COVID-19, and discontinued from study due to COVID-19, along with corresponding reason will be tabulated by treatment group as treated.
- Disposition events due to COVID-19 will also be indicated in the by participant disposition listings.

7.11.2 COVID-19 Related Dose Modifications

- Dose modifications (e.g., delay, discontinuation, etc.) due to COVID-19 will be summarized similarly as the summaries specified in Section 7.4.2.
- Dose modifications due to COVID-19 will also be indicated in the by- participant listing of dosing.

7.11.3 COVID-19 Related Adverse Events

COVID-19 related adverse events is defined by the SMQ of “COVID-19 (SMQ)” with “narrow scope”. COVID-19 related adverse events will be presented by SOC/PT, and summarized by treatment group as treated. A listing of COVID-19 related adverse events will also be provided.

8 CONVENTIONS

All conventions for imputing partial dates for analyses requiring dates will follow the BMS Global Standards Specifications and/or IO Core Standard Specifications, where applicable. Additional conventions are specified below. Further details will be documented in the study-specific data specifications.

- For missing and partial adverse event resolution dates, imputation will be performed as follows:
  - If only the day of the month is missing, the last day of the month will be used to replace the missing day. If the imputed date is after the death date or the last known alive date, then the latest known alive date or death date is considered as the resolution date.
  - If the day and month are missing or a date is completely missing, it will be considered as missing.
- For surgery, radiotherapy, and systemic cancer therapy, date imputation rules will be specified in the analysis dataset specifications.
- For death dates, the following conventions will be used for imputing partial dates:

Approved v2.0 930199387 2.0

- If only the day of the month is missing, the 1st of the month will be used to replace the missing day. The imputed date will be compared to the last known alive date and the maximum will be considered as the death date.
- If the month or the year is missing, the death date will be imputed as the last known alive date.
- If the date is completely missing but there is any evidence of death, the death date will be imputed as the last known date alive.

- For date of recurrence after start of study therapy, the following conventions will be used for imputing partial dates:
  - If only the day of the month is missing, the 1st of the month will be used to replace the missing day. In case of the date of death is present and complete, the imputed recurrence date will be compared to the date of death. The minimum of the imputed recurrence date and date of death will be considered as the date of recurrence.
  - If the day and month are missing or a date is completely missing, it will be considered as missing.
- For date of recurrence to prior therapies, the following conventions will be used for imputing partial dates:
  - If only the day of the month is missing, the 1st of the month will be used to replace the missing day.
  - If the day and month are missing or a date is completely missing, it will be considered as missing.
- For other partial/missing dates, the following conventions were used:
  - If only the day of the month is missing, the 15th of the month will be used to replace the missing day.
  - If both the day and the month are missing, “July 1” will be used to replace the missing information.
  - If a date is completely missing, it will be considered as missing.

The following conversion factors will be used to convert days to months or years:

1 month = 30.4375 days and 1 year = 365.25 days.

Duration (e.g., time-to onset, time-to resolution) will be calculated as follows:

Duration = (Last date - first date + 1)

Last known alive date will be defined based on all appropriate dates collected on the CRF.

All statistical analyses will be carried out using SAS (Statistical Analysis System software, SAS Institute, North Carolina, USA) unless otherwise noted.

8.1.1 Pharmacokinetic Summaries

<u>Handling of Non-Quantifiable Concentrations5</u>

Approved v2.0 930199387 2.0

For the summaries of matrix concentration-time data, concentrations that are less than the lower limit of quantification (LLOQ) should be displayed as "< LLOQ" in the listings and be treated as missing in summary tables and plots.

Summary statistics for Ctrough concentrations will be calculated by imputing values less than LLOQ as ½ * LLOQ. This imputation is done for Ctrough concentrations because it is treated like a PK parameter; the imputation is not done for Day 1 pre-dose concentrations. Individual Ctrough listings will display these concentrations as "< LLOQ."

All available matrix concentration-time data will be included in the PK data set and listed accordingly.

9 CONTENT OF REPORTS

All analyses described in this SAP will be included in the Clinical Study Report(s) except where otherwise noted. Refer to the Data Presentation Plan for mock-ups of all tables and listings.

9.1 Within-Trial Analyses Performed To Date

None of the efficacy endpoints have been analyzed yet and no alpha has been spent yet, since the first Interim Analysis has not occurred at this stage. Safety endpoints have been analyzed during periodic DMC meetings. Only the SDAC Reporting statistician and the DMC have been unblinded at this stage.

Approved v2.0 930199387 2.0

10 DOCUMENT HISTORY

Table 10-1: Document History

Version Number	Author(s)	Description
1.0	[redacted]	Original Issue
2.0	[redacted]	• Update of CSR SAP throughout the document to reflect the protocol amendment 02 and 03.<br>• Addition of Table 2.4-1 to summarize which endpoints have been downgraded as exploratory endpoints.<br>• Implementation of comments from the core/senior development team.<br>• Section 4:<br>o RFS and OS censoring rules for the main and supplemental estimands have been clarified. Estimands have been updated as well according to the protocol. Supplemental analysis of RFS and OS have been clarified. Supplemental estimand of DMFS has been removed, as it's no longer a key secondary endpoint.<br>o PFS2 definition and censoring rules have been updated.<br>o Time To Next Treatment (TTNT) has been added, as it's included in protocol amendment #02. Duration of next line therapy, TTNT, TFI and FFR have been moved to an exploratory SAP.<br>o Time windows of COA have been updated.<br>o Time to confirmed deterioration in HRQoL has been removed, as it's no longer included in the protocol.<br>• Section 6:<br>o Baseline definition for efficacy has been corrected.<br>o Addition of global study population.<br>o PRO analysis population is no longer needed and has been removed.<br>o PK evaluable population removed as this is a duplicate from PK population<br>o Biomarker populations have been clarified.<br>• Section 7.2:<br>o A listing of important protocol deviations will be prepared by GBDS.<br>o Relevant PDs have been updated to align with the DMC SAP.<br>• Section 7.3:<br>o Baseline characteristics have been updated to align with the DMC SAP.<br>• Section 7.5<br>o Only the 95% CI of HR will be presented, not the one with adjusted alpha level.<br>o Sensitivity analysis #3 of RFS has been removed. Sensitivity analysis #5 of RFS will be considered in an exploratory SAP. Sensitivity analysis #4 of OS will be considered in an

Table 10-1: Document History

Version Number	Author(s)	Description
		exploratory SAP.<br>o Subset analysis of RFS, OS have been updated, to align with DMC SAP.<br>o Supplemental, sensitivity and subset analyses of DMFS have been removed, as it's no longer a key secondary endpoint. Log-rank test of DMFS endpoint has been removed, considering this endpoint is not included in the multiple strategy testing.<br>o Extent to follow-up has been added in Section 7.5.2.3.<br>o Categories for the currentness of follow-up of RFS and OS have been updated.<br>o PFS2 analysis has been updated to align with the definition from Section 4.<br>o Timing of Interim Analyses in Section 7.5.7 have been corrected.<br>• Section 7.6:<br>o SARS-CoV-2 exploratory endpoint has been added.<br>o Laboratory section has been updated to include a listing of TSH levels<br>• Section 7.8:<br>o Biomarker forest plots have been removed, no longer needed. KM plots by PD-L1 have been updated and clarified.<br>o Biomarker analysis has been added for DMFS.<br>• Section 7.10:<br>o The status of participants who are censored in TCD KM analysis has been removed in QoL section, no longer needed.<br>• Section 7.11:<br>o The sentence stating "the SARS COV-2 related analysis will not be reported in the CSR" has been deleted.<br>• Section 9.1 from IO Core SAP v2.0 has been added.<br>• Minor typographical corrections throughout the document.

Approved v2.0 930199387 2.0

11 REFERENCES

1 Aaronson N.K., Ahmedzai S., et al. The European Organisation for Research and Treatment of Cancer QLQ-30: a quality-of-life instrument for use in international clinical trials in oncology. J Natl Cancer Inst, 85: 365-376, 1993.

2 Fayers PM, Aaronson NK, Bjordal K, Groenvold M, Curran D, Bottomley A. The EORTC QLQ-C30 Scoring Manual. 3rd ed: Brussels: European Organisation for Research and Treatment of Cancer; 2001.

3 Hernandez-Alava M and Pudney S. Econometric modelling of multiple self-reports of health states: The switch from EQ-5D-3L to EQ-5D-5L in evaluating drug therapies for rheumatoid arthritis. J Health Econ. 2017 Sep;55:139-152. doi: 10.1016/j.jhealeco.2017.06.013. Epub 2017 Jul 4.

4 Hernandez-Alava M, Wailoo A, Pudney S. Methods for Mapping Between the EQ-5D-5L and the 3L for Technology Appraisal. Report by the Decision Support Unit. 11 July 2017. Health Economics and Decision Science, School of Health and Related Research, University of Sheffield, UK.

5 The EuroQol Group: EuroQol: A new facility for the measurement of health-related quality of life—The EuroQol Group. Health Policy 16:199-208, 1990.

6 Brookmeyer R. and Crowley J. A confidence interval for the median survival time. Biometrics 38:29-41, 1982.

7 Klein, J. P. and Moeschberger, M. L. (1997), Survival Analysis: Techniques for Censored and Truncated Data, New York: Springer-Verlag.

8 Schoenfeld, David A., and Anastasios A. Tsiatis. "A modified log rank test for highly stratified data." Biometrika 74.1 (1987): 167-175.

9 Kernan, Walter N., et al. "Stratified randomization for clinical trials." Journal of clinical epidemiology 52.1 (1999): 19-26.

10 De Stavola, B. L., and D. R. Cox. "On the consequences of overstratification." Biometrika 95.4 (2008): 992-996.

11 Feng, Changyong, Hongyue Wang, and Xin M. Tu. "Power loss of stratified log-rank test in homogeneous samples." Journal of Quality and Reliability Engineering 2010 (2010).

12 Glimm E, Maurer W, Bretz F. Hierarchical testing of multiple endpoints in group-sequential trials. Statistics in Medicine. 2010;29:219-228.

13 Greenwood, M. The errors of sampling of the survivorship tables, Reports on Public Health and Statistical Subjects, 33, Appendix 1, HMSO, London, 1926.

Approved v2.0 930199387 2.0

14 Kalbfleisch, J. D. and Prentice, R. L. (1980), The Statistical Analysis of Failure Time Data, New York: John Wiley & Sons.

15 O'Brien PC, Fleming TR. A multiple testing procedure for clinical trials. Biometrics, 35, 549-556 (1979).

16 DeMets DL, Lan KK. Interim analysis: the alpha spending function approach. Stat Med. 1994 Jul 15-30; 13(13-14):1341-52.

17 The Statistical Analysis of Recurrent Events. Cook and Lawless, 2007 Springer.

18 Osoba D, Rodrigues G, Myles et al. Interpreting the significance of changes in health-related quality of life scores. J Clin Oncol 1998;16:139-44.

19 Pickard AS, Neary MP, Cella D. Estimation of minimally important differences in EQ-5D utility and VAS scores in cancer. Health Qual Life Outcomes 2007;5:70.

Approved v2.0 930199387 2.0

Approved v2.0 930199387 2.0

APPENDIX 1 TIME-TO ONSET AND TIME-TO RESOLUTION DEFINITION AND CONVENTIONS FOR SELECT ADVERSE EVENTS, IMMUNE-MEDIATED ADVERSE EVENTS AND EVENTS OF SPECIAL INTEREST

Time-to onset definition

Time-to onset of AE (any grade) for a specific category is defined as the time between the day of the first dose of study treatment and the onset date of the earliest AE (of any grade) in this category.

The time-to onset of AE (grade 3-5) for a specific category is defined similarly with an onset date corresponding to a grade 3-5 AE.

Time-to onset of drug-related AE (any grade or grade 3-5) for a specific category is defined similarly but restricted to drug-related AE.

Time-to onset for a specific subcategory is defined similarly but restricted to event of this subcategory.

Time-to resolution definition

In order to derive the time-to resolution, overlapping or contiguous AEs within a specific category or subcategory will be collapsed into what will be termed "clustered" AEs. For example, if a participant (without pre-treatment AE) experienced an AE from 1st to 5th January, another AE (with different PT but within same category) from 6th to 11th January and same AE from 10th to 12th January, these will be collapsed into one clustered AE from 1st to 12th January. Table 1 is summarizing key derivation steps for each type of clustered AEs.

Time-to resolution of AE (any grade) for a specific category is defined as the longest time from onset to complete resolution or improvement to the grade at baseline among all clustered AEs experienced by the participant in this category per adverse event criteria category. Events which worsened into grade 5 events (death) or have a resolution date equal to the date of death are considered unresolved. If a clustered AE is considered as unresolved, the resolution date will be censored to the last known alive date. Improvement to the grade at baseline implies that all different events in the clustered adverse event should at least have improved to the corresponding (i.e. with same preferred term) baseline grade. This measure is defined only for participants who experienced at least one AE in the specific category.

The time-to resolution of AE (grade 3-5) for a specific category is defined similarly with an onset date corresponding to a grade 3-5 AE.

Time-to resolution of drug-related AE (any grade or grade 3-5) for a specific category is defined similarly but restricted to drug-related AE.

The time-to resolution of AE (any grade or grade 3-5, drug-related or all) where immune modulating medication was initiated is defined similarly.

Time-to resolution for a specific subcategory is defined similarly but restricted to event of this subcategory.

Table 1: Derivation of clustered AE

Type of clustered AE	Derivation
Any grade	Collapse any on-treatment AE from the same category
Drug-related of any grade	Collapse any on-treatment drug-related AE from the same category
Grade 3-5	Collapse any on-treatment AE from the same category. Resolution will be based on the onset date of the earliest grade 3-5 records (if no grade 3-5 record, clustered AE is excluded)
Drug-related of Grade 3-5	Collapse any on-treatment drug-related AE from the same category Resolution will be based on the onset date of the earliest grade 3-5 record (if no Grade 3-5 record, clustered AE is excluded)

The algorithm for collapsing adverse event records is using the following conventions:

For each participant and specified category, the corresponding adverse event records will be collapsed when:

1) Multiple adverse event records have the same onset date.
2) The onset date of an event record is either the same day or 1 day later than the resolution date of a preceding event record (contiguous events).
3) The onset date of an event record is after the onset date and prior to or on the resolution date of a preceding event record (overlapping events).

Approved v2.0 930199387 2.0

APPENDIX 2 BIOMARKER ENDPOINTS

PD-L1 Tumor Cell (PD-L1 TC)

PD-L1 TC protein expression is quantified by immunohistochemistry (IHC) on fresh/frozen/ formalin-fixed paraffin embedded (FFPE) tumor biopsy samples with adjacent normal tissue collected prior to randomization/initial dose first treatment visit. The validated assay kit, Dako PD-L1 IHC pharmDx™ Kit (SK005), employing the 28-8 antibody, is used to stain sample slides. Stained slides are analyzed and scored by Dako-trained pathologist(s) at LabCorp for PD-L1 partial or complete linear circumferential tumor cell plasma membrane staining, expressed on a scale from 0%-100%. Tested samples lacking scores are confirmed unevaluable or indeterminate. For the purposes of the statistical analysis, the PD-L1 TC endpoint will use only quantifiable samples, excluding indeterminate and not evaluable.

- Indeterminate: Tumor cell membrane staining hampered for reasons attributed to the biology of the tumor tissue sample and not because of improper sample preparation or handling.
- Not evaluable: Tumor tissue sample was not optimally collected or prepared and PD-L1 expression is neither quantifiable nor indeterminate. Not evaluable can be determined from H&E process before the tumor biopsy specimen is sent for PD-L1 evaluation or from the H&E process during PD-L1 evaluation.

LAG-3 Immune Cell

LAG-3 expression is determined with a commercially available reagent (mouse monoclonal antibody 17B4) using analytically validated assay on fresh/frozen/formalin-fixed paraffin embedded (FFPE) tumor biopsy samples with adjacent normal tissue collected prior to randomization/initial dose first treatment visit. Positive staining is defined as cytoplasmic and/or partial or complete plasma membrane staining of any intensity of cells morphologically resembling lymphocytes relative to all cells within the overall tumor region (including tumor cells, macrophages/histiocytes, lymphocytes, plasma cells, myeloid cells, dendritic cells, giant cells and stromal cells). Stained slides are analyzed and scored by trained pathologist(s) at LabCorp, expressed on a scale from 0%-100%. Tested samples lacking scores are confirmed. For the purposes of the statistical analysis, the LAG-3 endpoint will use only quantifiable samples, excluding not evaluable.

- Not evaluable: Tumor tissue sample was not optimally collected or prepared and LAG-3 expression is neither quantifiable nor indeterminate. Not evaluable can be determined from H&E process before the tumor biopsy specimen is sent for LAG-3 evaluation or from the H&E process during LAG-3 evaluation.

Approved v2.0 930199387 2.0

APPENDIX 3 IMMUNOGENICITY ANALYSIS: BACKGROUND AND RATIONALE

The following summary is from the FDA Guidance for Industry Immunogenicity Assessment for Therapeutic Protein Products and White Paper on Assessment and Reporting of the Clinical Immunogenicity of Therapeutic Proteins and Peptides – Harmonized Terminology and Tactical Recommendations by Shankar et al. The program-level definitions of sample- and participant-level ADA status are based on recommendation from the BMS Immunogenicity Council.

Immune responses to therapeutic protein products may pose problems for both patient safety and product efficacy. Immunologically based adverse events, such as anaphylaxis and infusion reactions, have caused termination of the development of therapeutic protein products or limited the use of otherwise effective therapies. Unwanted immune responses to therapeutic proteins may also neutralize the biological activity of therapeutic proteins and may result in adverse events not only by inhibiting the efficacy of the therapeutic protein product, but by cross-reacting to an endogenous protein counterpart, if present. Because most of the adverse effects resulting from elicitation of an immune response to a therapeutic protein product appear to be mediated by humoral mechanisms, circulating antibody has been the chief criterion for defining an immune response to this class of products.

ADA is defined as biologic drug-reactive antibody, including pre-existing host antibodies that are cross-reactive with the administered biologic drug (baseline ADA). Titer is a quasiquantitative expression of the level of ADA in a sample. By employing a serial dilution-based test method, titer is defined as the reciprocal of the highest dilution of the sample (e.g., dilution of 1/100 = titer of 100). The ADA is also tested, via a cell-based biologic assay or a non cell-based competitive ligand-binding assay for a subpopulation of ADA known as neutralizing antibodies (NAb), which inhibits or reduces the pharmacological activity of the biologic drug molecule regardless of its in vivo clinical relevance. Non-neutralizing ADA (non-NAb) is ADA that binds to the biologic drug molecule but does not inhibits its pharmacological activity.

ADA should be tested using sensitive and valid methods and employing an appropriate strategy for elucidating immunogenicity. Detection of ADA is typically performed in three tiers (screening, confirmatory, and titer) using statistically determined cutpoints and samples testing positive in the ADA assay are analyzed for neutralizing activity, especially in late-stage clinical studies. "Detection" of ADA implies that drug-specific ADA was confirmed. The 'drug tolerance" of an assay (highest drug concentration that does not interfere in the ADA detection method) is not an absolute value and differs between individuals due to the varying avidities of ADA immune responses. An ADA sampling strategy of collecting samples at times when the least drug concentration is anticipated (trough concentrations) can increase the likelihood of accurate ADA detection.

It is useful to present ADA results from clinical studies as (a) characteristics of the ADA immune response, (b) relationship of ADA with pharmacokinetics (PK) and, when relevant, pharmacodynamics (PD) biomarkers, and (c) relationship of ADA with clinical safety and efficacy.

Approved v2.0 930199387 2.0

Clinical consequences of ADA can range from no apparent clinical effect to lack of efficacy (primary treatment failure), loss of efficacy (secondary treatment failure) or heightened effect due to altered exposure to the biologic drug, adverse drug reactions (administration-related systemic or site reactions), and severe adverse drug reactions (anaphylaxis and unique clinical problems associated with cross-reactivity and neutralization of endogenous molecules). Thus it becomes important to examine any associations between ADA or any of its attributes with the various clinical sequelae. The presence of ADA may or may not preclude the administration of drug to ADA-positive participants because the outcome is dependent upon the magnitude of the impact of ADA on PK and PD. Hence, the relationship of ADA with PK/PD is an important additional consideration, but does not necessarily result in a clinically impactful consequence per se.

Immunogenicity Endpoints

A fundamental metric that informs clinical immunogenicity interpretation is the incidence of ADA in a study or across comparable studies. ADA incidence is defined as the proportion of the study population found to have seroconverted or boosted their pre-existing ADA during the study period.

Terms and Definitions

Validated ADA test methods enable characterization of samples into ADA-positive vs. ADA-negative. To classify the ADA status of a participant using data from an in vitro test method, each sample from the participant is categorized based on the following definitions:

Sample ADA Status**:**

- Baseline ADA-positive sample: ADA is detected in the last sample before initiation of treatment
- Baseline ADA-negative sample: ADA is not detected in the last sample before initiation of treatment
- ADA-positive sample: After initiation of treatment, (1) an ADA detected (positive seroconversion) sample in a participant for whom ADA is not detected at baseline, or (2) an ADA detected sample with ADA titer to be at least 4-fold or greater (≥) than baseline positive titer
- ADA-negative sample: After initiation of treatment, ADA not positive sample relative to baseline

Next, using the sample ADA status, participant ADA status is defined as follows: Participant ADA Status:

- Baseline ADA-positive participant: A participant with baseline ADA-positive sample
- **ADA-positive participant**: A participant with at least one ADA positive-sample relative to baseline at any time after initiation of treatment

1) *Persistent Positive (PP)*: ADA-positive sample at 2 or more consecutive timepoints, where the first and last ADA-positive samples are at least 16 weeks apart
2) *Not PP-Last Sample Positive*: Not persistent positive with ADA-positive sample at the last sampling timepoint

3) *Other Positive*: Not persistent positive but some ADA-positive samples with the last sample being negative
4) *Neutralizing Positive*: At least one ADA-positive sample with neutralizing antibodies detected

- **ADA-negative participant:** A participant with no ADA-positive sample after the initiation of treatment.

  (Note: 16 weeks was chosen based on a long half-life of IgG4.)

Population for Analyses

Analysis of immunogenicity data will be based on ADA evaluable participants defined as all treated participants with baseline and at least 1 post-baseline immunogenicity assessment. Analysis dataset and data listing will include all available ADA samples. However, participant-level ADA status will be defined based on only adequate samples (e.g., excluding 1-hour post-infusion samples when clearly indicated).

Approved v2.0 930199387 2.0
