# Supplementary material for: Adjuvant nivolumab and relatlimab in stage III/IV melanoma: the randomized phase 3 RELATIVITY-098 trial
Source: Nat Med. 2025 Oct 18;31(12):4301–9. doi: 10.1038/s41591-025-04032-8 (PMC12705465; doi:10.1038/s41591-025-04032-8)
Supplement: Supplementary file 2 — Reporting Summary [file 41591_2025_4032_MOESM2_ESM.pdf]

nature portfolio

Corresponding author(s): Georgina V Long

Last updated by author(s): Sep 24, 2025

Reporting Summary

Nature Portfolio wishes to improve the reproducibility of the work that we publish. This form provides structure for consistency and transparency in reporting. For further information on Nature Portfolio policies, see our Editorial Policies and the Editorial Policy Checklist.

Statistics

For all statistical analyses, confirm that the following items are present in the figure legend, table legend, main text, or Methods section.

n/a	Confirmed	
☐	☒	The exact sample size (*n*) for each experimental group/condition, given as a discrete number and unit of measurement
☐	☒	A statement on whether measurements were taken from distinct samples or whether the same sample was measured repeatedly
☐	☒	The statistical test(s) used AND whether they are one- or two-sided<br>*Only common tests should be described solely by name; describe more complex techniques in the Methods section.*
☐	☒	A description of all covariates tested
☐	☒	A description of any assumptions or corrections, such as tests of normality and adjustment for multiple comparisons
☐	☒	A full description of the statistical parameters including central tendency (e.g. means) or other basic estimates (e.g. regression coefficient) AND variation (e.g. standard deviation) or associated estimates of uncertainty (e.g. confidence intervals)
☐	☒	For null hypothesis testing, the test statistic (e.g. *F*, *t*, *r*) with confidence intervals, effect sizes, degrees of freedom and *P* value noted<br>*Give P values as exact values whenever suitable.*
☒	☐	For Bayesian analysis, information on the choice of priors and Markov chain Monte Carlo settings
☒	☐	For hierarchical and complex designs, identification of the appropriate level for tests and full reporting of outcomes
☐	☒	Estimates of effect sizes (e.g. Cohen's *d*, Pearson's *r*), indicating how they were calculated

Our web collection on statistics for biologists contains articles on many of the points above.

Software and code

Policy information about availability of computer code

Data collection	Medidata Classic Rave (version 2025.1; New York, NY, USA).
Data analysis	All clinical analyses were performed using Statistical Analysis System software (SAS Institute, North Carolina, USA). All biomarker statistical analyses were performed using R version 4.3.1.

For manuscripts utilizing custom algorithms or software that are central to the research but not yet described in published literature, software must be made available to editors and reviewers. We strongly encourage code deposition in a community repository (e.g. GitHub). See the Nature Portfolio guidelines for submitting code & software for further information.

Data

Policy information about availability of data

All manuscripts must include a data availability statement. This statement should provide the following information, where applicable:

- Accession codes, unique identifiers, or web links for publicly available datasets
- A description of any restrictions on data availability
- For clinical datasets or third party data, please ensure that the statement adheres to our policy

Data availability: Bristol Myers Squibb will honor legitimate requests for our clinical trial data from qualified researchers with a clearly defined scientific objective. We share data from Phase II-IV interventional clinical trials completed on or after January 1, 2008 and evaluate medicines and indications approved in the US, EU, and other designated markets. Data shared may include non-identifiable patient-level and study-level clinical trial data, full clinical study reports and protocols.

Sharing is subject to protection of patient privacy and respect for the patient's informed consent, and publication of the primary results in peer-reviewed journals. Bristol Myers Squibb reserves the right to update and change criteria at any time. Other criteria may apply, for details please visit Bristol Myers Squibb at https://www.bms.com/researchers-and-partners/independent-research/data-sharing-request-process.html. The option to submit data requests as well as review criteria for data requests are available at https://vivli.org/ourmember/bristol-myers-squibb/. The study protocol of RELATIVITY-098 is provided in the Supplementary Information.

Research involving human participants, their data, or biological material

Policy information about studies with human participants or human data. See also policy information about sex, gender (identity/presentation), and sexual orientation and race, ethnicity and racism.

Reporting on sex and gender	Both male and female patients were eligible for enrollment. Sex was self-reported. The number of male (n = 642) and female (n = 451) patients randomized in this study is reported in Table 1. Evaluations of recurrence-free survival in several prespecified subgroups, including subgroups defined by sex, are reported in Extended data Fig. 1.
Reporting on race, ethnicity, or other socially relevant groupings	There are no analyses based on race or ethnicity reported in this manuscript.
Population characteristics	Baseline patient demographics (including age, sex, geographic region) and disease characteristics for patients randomized in this study are reported in Table 1.
Recruitment	From October 2021 to November 2022, a total of 1093 patients were randomly assigned to receive nivolumab plus relatlimab (547 patients) or nivolumab (546 patients) (Fig. 1) at 163 hospitals and cancer centers in 24 countries worldwide (site list is provided in the supplement). Patients were recruited by investigators through enrollment based on prespecified inclusion/exclusion criteria. Eligibility criteria and screening procedures minimized the potential of self-selection bias.
Ethics oversight	The protocol and amendments for this trial were reviewed by the institutional review board or independent ethics committee for each trial site and all patients provided written informed consent before enrollment. The trial was conducted in accordance with the International Council for Harmonisation Good Clinical Practice Guidelines.

Note that full information on the approval of the study protocol must also be provided in the manuscript.

Field-specific reporting

Please select the one below that is the best fit for your research. If you are not sure, read the appropriate sections before making your selection.

☒ Life sciences ☐ Behavioural & social sciences ☐ Ecological, evolutionary & environmental sciences

For a reference copy of the document with all sections, see nature.com/documents/nr-reporting-summary-flat.pdf

Life sciences study design

All studies must disclose on these points even when the disclosure is negative.

Sample size	An approximate sample size of 1050 patients was planned to achieve the required 410 RFS events and show a significant difference in investigator-assessed RFS with a two-sided alpha of 0.05 using a stratified log-rank test with at least 90% statistical power when the average hazard ratio of nivolumab and relatlimab fixed-dose combination versus nivolumab was 0.72 and an assumed cure rate of 0.52 in the nivolumab arm. The actual events were 427, with a critical HR of 0.815 and cumulative power of 91.5%. A total of 1093 patients were randomly assigned to receive nivolumab plus relatlimab (547 patients) or nivolumab (546 patients).
Data exclusions	No data were excluded from the reported clinical analyses. Biomarker analyses were performed on biomarker-evaluable samples.
Replication	Attempts of replication were not performed given that RELATIVITY-098 was a clinical trial.
Randomization	Randomization was carried out via permutated blocks within each stratum, defined by a combination of geographic region and AJCC v8 stage. Patients were stratified according to AJCC-8 stage (IIIA/IIIB or IIIC or IIID/IV [including mucosal melanoma]), and geographic region (USA/Canada/Australia or Europe or rest of the world).
Blinding	This was a double-blind study. Access to treatment codes was restricted for all participants, as well as site and Sponsor personnel, prior to the primary database lock, with exceptions as follows: in the event of a medical emergency or pregnancy involving an individual participant, where knowledge of the investigational product was essential for participant management, the blind for that participant was broken by the investigator. The participant's safety took priority over any other considerations when determining if treatment assignment was to be unblinded.

Reporting for specific materials, systems and methods

We require information from authors about some types of materials, experimental systems and methods used in many studies. Here, indicate whether each material, system or method listed is relevant to your study. If you are not sure if a list item applies to your research, read the appropriate section before selecting a response.

Materials & experimental systems

n/a	Involved in the study
☐	☒ Antibodies
☒	☐ Eukaryotic cell lines
☒	☐ Palaeontology and archaeology
☒	☐ Animals and other organisms
☐	☒ Clinical data
☒	☐ Dual use research of concern
☒	☐ Plants

Methods

n/a	Involved in the study
☒	☐ ChIP-seq
☐	☒ Flow cytometry
☒	☐ MRI-based neuroimaging

Antibodies

Antibodies used

Nivolumab, an anti-PD-1 monoclonal antibody, and relatlimab anti-LAG-3 antibody, were administered as the experimental treatment in this study. Nivolumab and relatlimab administered as part of this study was provided by the study's sponsor (Bristol Myers Squibb).

For the IHC biomarker analyses, the following antibodies were used: PD-L1 immunohistochemistry assay (Dako PD-L1 IHC 28-8 pharmDx assay; Agilent Technologies, Inc.), LAG-3 (17B4) mouse monoclonal antibody, CD8 (mouse clone C8/144B) antibody

For biomarker analyses using CODEX/phenocycler, the following antibodies were employed:
Detection of E-cadherin was performed using the 4250021 (Akoya Inventoried) antibody.
Detection of CD45RO was performed using the 4250023 (Akoya Inventoried) antibody.
Detection of CD8 was performed using the 4250012 (Akoya Inventoried) antibody.
Detection of Ki67 was performed using the 4250019 (Akoya Inventoried) antibody.
Detection of CD163 was performed using the 2450079 (Akoya Inventoried) antibody.
Detection of CD4 was performed using the 4550112 (Akoya Inventoried) antibody.
Detection of CD68 was performed using the 4550113 (Akoya Inventoried) antibody.
Detection of CD45 was performed using the 4550121 (Akoya Inventoried) antibody.
Detection of FOXP3 was performed using the 4550071 (Akoya Inventoried) antibody.
Detection of HLA-DR was performed using the 4550118 (Akoya Inventoried) antibody.
Detection of PCNA was performed using the 4550124 (Akoya Inventoried) antibody.
Detection of Collagen IV was performed using the 4550122 (Akoya Inventoried) antibody.
Detection of PD-L1 was performed using the 4550072 (Akoya Inventoried) antibody.
Detection of CD3e was performed using the 4550119 (Akoya Inventoried) antibody.
Detection of PD-1 was performed using the 4550038 (Akoya Inventoried) antibody.
Detection of LAG3 was performed using the 17b4 antibody.
Detection of CD20 was performed using the 4450018 (Akoya Inventoried) antibody.
Detection of PanCK was performed using the 4450020 (Akoya Inventoried) antibody.
Detection of SOX10 was performed using the 5H7L26 antibody.

Validation

Adjuvant nivolumab plus relatlimab for patients with resected stage IIIB/C/D or stage IV melanoma was evaluated vs nivolumab in this study as part of Bristol Myers Squibb's clinical study program.

The majority of the *Akoya Inventoried antibodies were validated by Akoya described on the Akoya website.
Clones chosen by the investigators were well-known and conjugated by Akoya. All antibodies were tested and the staining was validated with Pathologist.

Clinical data

Policy information about clinical studies
All manuscripts should comply with the ICMJE guidelines for publication of clinical research and a completed CONSORT checklist must be included with all submissions.

Clinical trial registration

NCT05002569

Study protocol

The study protocol is provided in the Supplementary Information.

Data collection

From October 2021 to November 2022, a total of 1093 patients were randomly assigned to rreceive 480 mg of nivolumab plus 160 mg of relatlimab as FDC or 480 mg of nivolumab administered via intravenous infusion every 4 weeks for a maximum of 1 year (or ≤ 13 doses) or until recurrence of disease, unacceptable adverse events, or withdrawal of consent at 163 sites in 24 countries worldwide. At the December 16, 2024 clinical cutoff date, patients had a minimum follow-up (time from last patient randomized to the cutoff date) of 23.4 months and a median follow-up (median time between randomization and the last known alive date) of 26.7 months.

Outcomes

The primary endpoint was investigator-assessed RFS, with computed tomography scans performed every 12 weeks for 2 years and

Outcomes

then twice a year; patients who did not undergo complete lymph node dissection required ultrasound surveillance. Overall survival was the key secondary endpoint to be hierarchically tested if the primary endpoint was met. Other secondary endpoints included investigator-assessed DMFS, PFS through next-line therapy, and safety. The severity of adverse events was graded according to the National Cancer Institute Common Terminology Criteria for Adverse Events version 5.0. Included treatment-related adverse events (TRAES) are those reported between the first dose and 30 days after the last dose. In addition, immune-mediated AEs and AEs of special interest reported between the first dose and 135 days after the last dose are reported per category.

Biomarker analyses were exploratory and were performed on patient blood samples from RELATIVITY-098 and RELATIVITY-047 (NCT03470922) at baseline and on treatment to assess expression or expansion of biomarker-specific immune cell populations and soluble proteins. Biomarker analyses were performed on patient tumor samples from RELATIVITY-098 at baseline and at recurrence and RELATIVITY-047 (NCT03470922) at baseline to assess expression of proteins in the tumor microenvironment.

Flow Cytometry

Plots

Confirm that:

☐ The axis labels state the marker and fluorochrome used (e.g. CD4-FITC).

☐ The axis scales are clearly visible. Include numbers along axes only for bottom left plot of group (a 'group' is an analysis of identical markers).

☐ All plots are contour plots with outliers or pseudocolor plots.

☒ A numerical value for number of cells or percentage (with statistics) is provided.

Methodology

Sample preparation

Whole blood collected on Cyto-Chex BCT tubes

Instrument

5-laser Cytek Aurora spectral cytometer

Software

OMIQ

Cell population abundance

The parent populations are CD4 and CD8 cells which are highly abundant in the total white cell population. Activation markers were analyzed on a subset of these cells that are LAG3+ or PD1+ populations which can range from 1-20% of the parent cells in normal circumstances.

Gating strategy

The original files were gated by time. Non-debris cells were selected by FSC and SSC. Doublets were excluded. Dead cells were excluded. Leukocytes were selected by SSC vs. CD45. From leukocytes, T cells were selected by CD3 positivity. T cells were subseted to CD4 or CD8 positive. CD4 and CD8 cells were subseted based on LAG3 or PD1 positivity. Activation markers (i.e Ki67) were measured from these cell subsets as a percent of parent.

☐ Tick this box to confirm that a figure exemplifying the gating strategy is provided in the Supplementary Information.
